# Photocatalytic Activation of Fluoroform for Radical Trifluoromethylation

**DOI:** 10.1021/jacs.6c10939

**Published:** 2026-08-04

**Authors:** Shun Zhou, Yan Song, Yu Harabuchi, Wataru Kanna, Hitomi Katsuyama, Simon J. Cooper, Kosaku Tanaka, Tsuyoshi Mita, Satoshi Maeda, Hiroki Hayashi

**Affiliations:** † Institute for Chemical Reaction Design and Discovery (WPI-ICReDD), 12965 Hokkaido University, Kita 21, Nishi 10, Kita-ku, Sapporo, Hokkaido 001-0021, Japan; ‡ JST, ERATO Maeda Artificial Intelligence in Chemical Reaction Design and Discovery Project, Hokkaido University, Kita 10, Nishi 8, Kita-ku, Sapporo, Hokkaido 060-0810, Japan; § Department of Chemistry, Faculty of Science, 12810 Hokkaido University, Kita 10, Nishi 8, Kita-ku, Sapporo, Hokkaido 060-0810, Japan

## Abstract

Fluoroform is an ideal trifluoromethyl source in synthetic chemistry due to its low cost, low toxicity, and high potential for greenhouse-gas valorization. However, the use of fluoroform has been largely limited to the generation of trifluoromethyl anions or metal-bound species; direct access to the trifluoromethyl radical remains elusive, despite the broad applicability of radical trifluoromethylation for preparing medicinally important compounds. Here, we report a method for radical trifluoromethylation using fluoroform by leveraging the reactivity of ketones in combination with a Brønsted base under photoirradiation. The ketone-mediated photochemical activation provides access to the trifluoromethyl radical from fluoroform via a photoexcited alkoxide intermediate without exogenous redox reagents, enabling radical trifluoromethylation of alkenes and arenes under mild conditions. The developed system is amenable to a photocatalytic protocol, late-stage trifluoromethylation of complex molecules, and upgrading bulk chemicals into high-value trifluoromethylated compounds.

## Graphical abstract

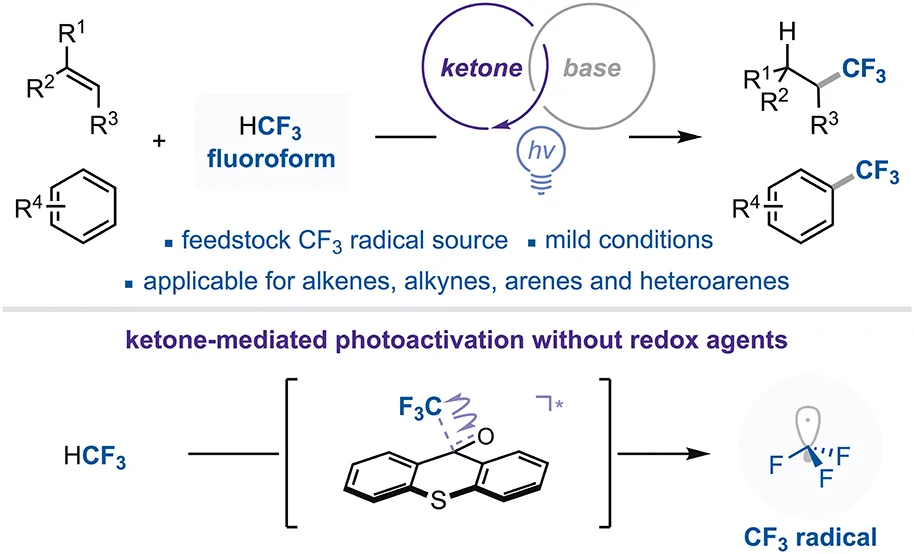

## Introduction

The conversion of inexpensive, abundant feedstocks into value-added compounds offers a path to advance sustainability goals in an economically viable manner. Gaseous light alkanes are ideally suited for direct deployment as alkyl group feedstocks in alkylation reactions, enabling cost- and atom-economical processes and obviating the need for bespoke alkylating agents.^1^ State-of-the-art technologies enable the activation of certain gaseous molecules to generate alkyl radical species that can be used as reactive intermediates to forge new chemical bonds under mild conditions and enhance molecular complexity. Even the chemically unreactive methane can be forced to undergo homolytic C–H bond cleavage to generate methyl radicals for the radical methylation of alkenes and aromatic compounds (Figure 1a).^2,3^ In this context, the analogous fluoroalkylation would be particularly valuable, as the incorporation of fluorine atoms into organic compounds is an effective strategy for the modulation of pharmacological properties in drug discovery, by altering factors such as metabolic stability, lipophilicity, and protein-binding affinity.^4,5^ Fluoroform, a gaseous light fluoroalkane and the simplest trifluoromethyl-containing compound,^6^ is generated in substantial quantities as a waste byproduct of Teflon manufacturing. Its chemical inertness leads to its accumulation in the atmosphere, where it acts as a greenhouse gas with a global warming potential approximately 14,800 times higher than that of CO_2_. Although regulations on fluoroform emissions have been tightened,^7,8^ global emissions are still increasing.^9^ Thus, its synthetic application in feedstock valorization would be highly compelling.

**Figure 1:**
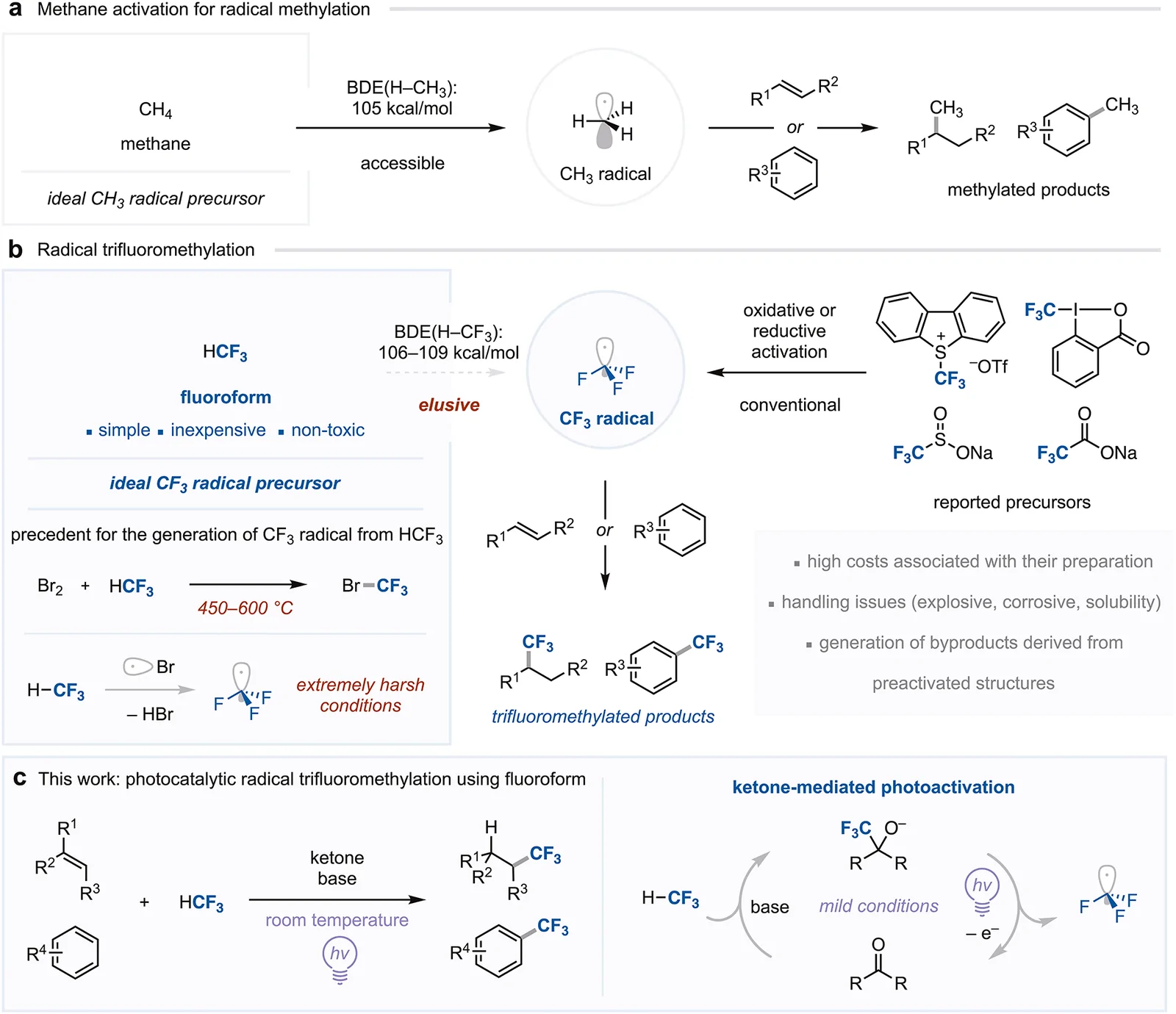
Background and challenges in the radical trifluoromethylation using fluoroform. (a) Radical methylation using methane. (b) Elusive radical trifluoromethylation using fluoroform and conventional precursors. (c) Photocatalytic activation of fluoroform to generate trifluoromethyl radicals (*this work*).

The trifluoromethyl group has primarily been employed in medicinal chemistry, and numerous pharmaceuticals feature trifluoromethyl units.^10,11^ Since fluoroform is abundant, relatively inexpensive, and of relatively low toxicity to humans, methods based on this ideal reagent for the trifluoromethylation of organic compounds have been studied over the last three decades.^12^ Pioneering studies have focused on the deprotonation of fluoroform to generate the trifluoromethyl anion for use in nucleophilic trifluoromethylation, as its proton is relatively acidic (p*K*a ≈ 27 in H_2_O)^13^ due to the electron-withdrawing effect of the fluorine atoms.^14–16^ In contrast, radical trifluoromethylation for the direct introduction of the trifluoromethyl group into unsaturated functionalities, bypassing the need to preactivate substrates, would be highly advantageous. However, no examples of the direct generation of trifluoromethyl radicals from fluoroform under mild conditions have been reported so far, which significantly limits its synthetic utility (Figure 1b).^17^

The generation of trifluoromethyl radicals has required activated precursor molecules, such as the Togni, Umemoto, or Langlois reagents.^18^ Recent progress has enabled the use of trifluoroacetic acid as a simpler precursor for the generation of trifluoromethyl radicals via oxidative decarboxylation.^19,20^ Gaseous bromotrifluoromethane and iodotrifluoromethane have also been employed as sources of trifluoromethyl radicals.^17^ However, the aforementioned trifluoromethyl radical precursors are associated with various drawbacks, including high cost, poor solubility, corrosivity, potential explosivity, and the formation of unwanted byproducts. We wondered whether fluoroform could be directly used as the trifluoromethyl radical precursor and thus enable ideal radical trifluoromethylation. While the activation of the C–H bond of fluoroform via hydrogen atom transfer (HAT) would appear to be a feasible strategy, its bond-dissociation energy (106–109 kcal/mol) is higher than the C–H bonds of methane (105 kcal/mol),^17,21,22^ and it is significantly more electrophilic than alkanes, making this approach less viable. To the best of our knowledge, the sole approach reported to date is the bromination of fluoroform with molecular bromine via HAT by bromine radicals; however, this method requires extremely harsh conditions (450–600 °C)^23,24^ that are rarely applicable in modern organic synthesis. Stepwise approaches also have been developed to generate trifluoromethyl radicals via the preparation and isolation of precursor metal complexes from fluoroform and stoichiometric amounts of metal salts, followed by their oxidative or photochemical activation.^25–28^ The resulting trifluoromethylation products are inevitably accompanied by stoichiometric amounts of byproducts derived from the metal precursors and/or oxidants. Thus, the direct and catalytic generation of trifluoromethyl radicals from fluoroform under mild conditions remains a long-standing unmet goal in the realm of radical trifluoromethylation methodology.

To address this challenge, we hypothesized that trifluoromethyl radicals could be generated from fluoroform by combining conventional deprotonation with photoexcitation, instead of direct HAT. Here, we report a method to generate trifluoromethyl radicals from fluoroform by the combination of a Brønsted base and a ketone mediator under photoirradiation. This process enables the direct radical trifluoromethylation of alkenes, alkynes, and aromatic compounds at room temperature (Figure 1c). The radical forms by deprotonation of fluoroform, addition of the trifluoromethyl anion to a carbonyl group, and the photoexcitation of the resulting alkoxide, to release the trifluoromethyl radical. This previously untapped activation mode is ultimately applicable to ketone-catalyzed radical trifluoromethylation using fluoroform as the trifluoromethyl radical precursor without the need for redox reagents. Mechanistic studies support the ketone-enabled radical-based mechanism as outlined above.

## Results and Discussion

### Methodology Development

We envisioned a multistep activation process involving the addition of the trifluoromethyl anion generated from fluoroform to a mediator molecule, followed by photoexcitation of the intermediate to generate the trifluoromethyl radical. We speculated that carbonyl compounds could serve as suitable mediators to trap the carbanions and form stable alkoxide intermediates (Figure 2a). To investigate whether the generation of radicals from the excited-state alkoxide could occur via homolytic C–C cleavage, we performed density functional theory (DFT) calculations (Figure S1). The computational results indicated that an alkoxide derived from thioxanthone (TX) and the trifluoromethyl anion could undergo C–C cleavage with an accessible energy barrier from its T_1_ excited state (10.7 kcal/mol) (Figure 2b). Molecular orbital and NBO spin-density analyses indicated that the T_1_ excited state contained significant radical character on the one of the arene rings, with maximum spin densities at the carbon atoms *ipso* and *para* to the sulfur atom.^29^ The resulting out-of-plane structure may contribute to a relatively low energy barrier for homolytic cleavage.

**Figure 2:**
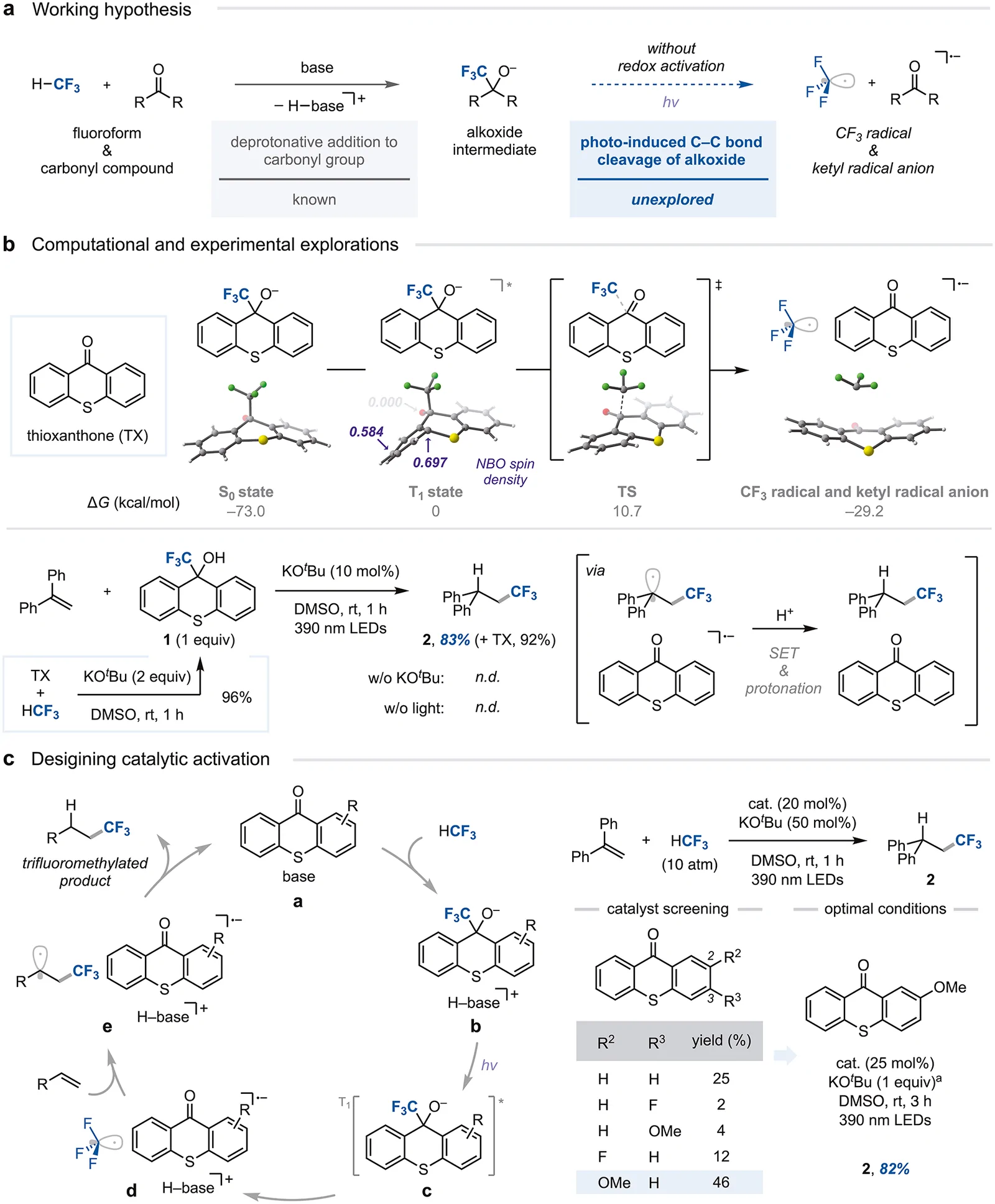
Design and development of the generation of trifluoromethyl radicals from fluoroform. (a) Working hypothesis for the generation of radicals through the deprotonation and photoexcitation of an alkoxide intermediate. (b) Computational and experimental explorations of the generation of trifluoromethyl radicals from the alkoxide derived from thioxanthone (TX). Computations were performed at the (U)­ωB97X-D/6-311+G­(2d,p)/CPCM­(DMSO)//(U)­ωB97X-D/6-31+G­(d)/CPCM­(DMSO) level of theory. (c) Design of the catalytic mechanism. Yields were determined by ^19^F NMR analysis; ^a^KO*^t^* Bu was added stepwise in portions of 0.5, 0.25, and 0.25 equiv; for details, see the Supporting Information.

This promising computational result prompted us to experimentally examine the feasibility of generating trifluoromethyl radicals this way. Alcohol intermediate **1** was readily accessed via a simple base-mediated deprotonative addition of fluoroform to TX. The hydrotrifluoromethylation of 1,1-diphenylethylene was examined as a model reaction. Based on UV–vis experiments (*vide infra*), which indicated that the proposed alkoxide absorbs in the purple region, the reaction was conducted under irradiation from purple LEDs (390 nm). Indeed, trifluoromethylated product **2** was obtained in 83% yield with a catalytic amount of KO^*t*^Bu, with excellent recovery (92%) of the mediator (TX). In the absence of the light source or KO^*t*^Bu, no trifluoromethylated product was formed. These control experiments indicate that the trifluoromethyl radical is generated from the alkoxide form of **1** by photoexcitation, as suggested by our computations, followed by addition of the radical to produce the benzylic alkyl radical intermediate. The cogenerated TX ketyl radical anion (*E*_1/2_^red^ = −1.62 V vs SCE)^30^ can reduce the alkyl radical intermediate (*E*_1/2_^red^ = −1.47 V vs SCE)^31^ via single electron transfer (SET), followed by protonation to afford **2** and TX.

The recovery of TX from these reactions inspired us to design a photocatalytic radical trifluoromethylation of alkenes using fluoroform with TX as a catalyst (Figure 2c). In this process, the catalyst TX and base (**a**) would deprotonate fluoroform to generate the alkoxide intermediate (**b**). Photoexcitation would give the triplet excited state (**c**) of the alkoxide intermediate, which would readily generate both the trifluoromethyl radical and the ketyl radical anion (**d**). The trifluoromethyl radical would then add to the alkene to form alkyl radical intermediate (**e**). Subsequent reduction by the ketyl radical anion and protonation would deliver the desired trifluoromethylated product and regenerate the catalyst TX and base (**a**). To develop a trifluoromethylation system based on this mechanism, we evaluated various TX derivatives as potential catalysts for the model hydrotrifluoromethylation. Under a high-pressure atmosphere to maintain a sufficient concentration of fluoroform, the desired trifluoromethylated product (**2**) was obtained in 25% yield using 20 mol % of TX. Screening a variety of TX derivatives with differing substitution patterns revealed that introducing either electron-donating or -withdrawing groups at the C2 position led to a higher yield of the trifluoromethylated product than that at the C3 position. The yield of the reaction with 2-methoxy-thioxanthone (2-OMe-TX) as catalyst was the highest yield (46%) among those with the catalysts tested. Further optimization of the reaction conditions, including a slight increase in catalyst loading to 25 mol % and the stepwise addition of KO^*t*^Bu, led to a photocatalytic system that delivered **2** in 82% yield (see Section 4 in the Supporting Information for details).

### Scope of the Radical Trifluoromethylation Using Fluoroform

With the optimized photoactivation system in hand, we investigated the scope of the radical trifluoromethylation using fluoroform. As a complement to the developed catalytic high-pressure system, the trifluoromethylation under ambient pressure using commercially available TX and stoichiometric conditions is also synthetically practical (Table S2). Therefore, the substrate scope was examined under both stoichiometric and catalytic conditions in order to assess the versatility of the developed trifluoromethylation protocol. Diarylethylene substrates underwent the trifluoromethylation to furnish products **2–4** in synthetically useful yields (Figure 3a). Notably, the catalytic method afforded **3** in 75% yield on the gram scale, demonstrating the practical utility and scalability of this method. The reaction of an unsymmetric diarylethylene containing a pyridine unit provided **5** in a similar manner. The trifluoromethylation protocol was successfully applied to styrene derivatives containing diverse functional groups, including halogens, alkyl groups, ethers, and amides, affording products **6–20** in moderate to high yields. While **17** could not be obtained from 4-vinylbenzoic acid in its free acid form, the corresponding potassium salt could be effectively used in the present radical trifluoromethylation system, affording **17** in up to 50% yield. A series of pyridine-containing compounds can also serve as substrates for trifluoromethylation, providing **21–23** in up to 80% yield. The developed trifluoromethylation was also compatible with a sterically demanding trisubstituted alkene substrate, leading to **24**. In addition to styrene-type substrates, α,β-unsaturated esters and amides afforded the corresponding products (**25** and **26**) in moderate-to-good yield (61% and 45%, respectively), although the catalytic conditions are not suitable for these alkenes due to their instability under the applied reaction conditions. Furthermore, the system is applicable to the radical trifluoromethylation of alkynes, affording vinyl trifluoromethylated products **27** and **28**. The challenging dearomative hydrotrifluoromethylation of an *N*-Boc indole was accomplished with the current reaction system to furnish trifluoromethylated indoline **29**. Finally, the developed system was applied to the radical trifluoromethylation of various bioactive compounds and their derivatives, including methyl isoeugenol, threonine, estrone, fenofibrate, and bexarotene. These reactions smoothly afforded trifluoromethylated products **30–34**, showcasing the broad applicability and effectiveness of the method, as well as its valuable applicability to the late-stage functionalization of complex molecules.

**Figure 3:**
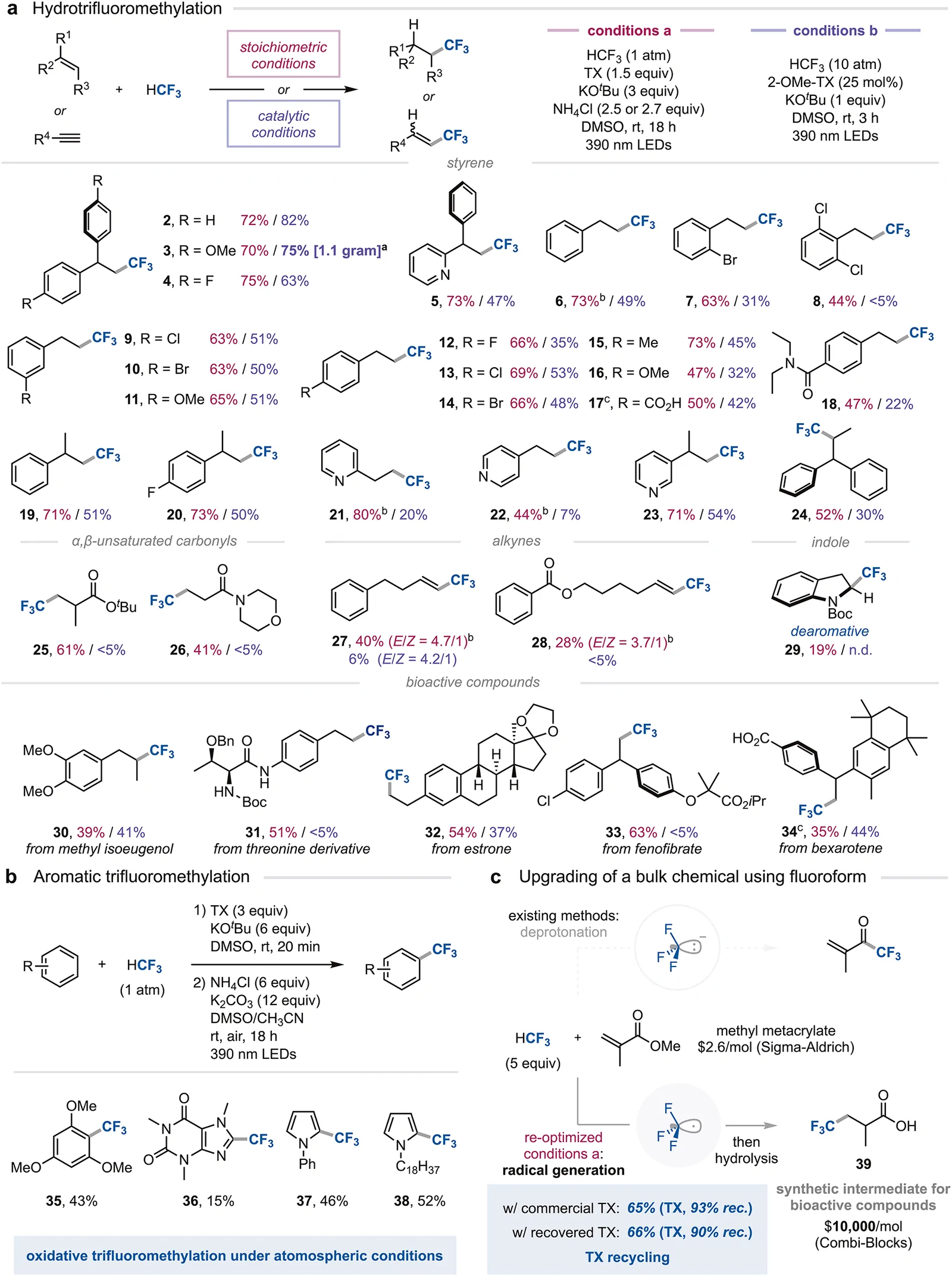
Radical trifluoromethylation using fluoroform. (a) Scope of the hydrotrifluoromethylation of alkenes and alkynes. For the stoichiometric conditions, isolated product yields are reported; for the catalytic conditions, yields were determined by ^19^F NMR analysis; ^a^isolated yields are shown; ^b^yields and/or *E*/*Z* ratios were determined by ^19^F NMR analysis. ^c^The potassium salts of carboxylic acid substrates were used for the reaction. (b) Application to the oxidative trifluoromethylation of aromatic compounds; isolated yields are shown. (c) Demonstration of the upgrading of a bulk chemical using the trifluoromethylation methods developed in this study; isolated yields are shown.

We envisioned that the developed system could also be applicable to the direct radical trifluoromethylation of aromatic compounds using fluoroform (Figure 3b).^32,33^ To achieve this transformation, an oxidant is required for the rearomatization step. To this end, we developed a radical trifluoromethylation protocol in which **1** was generated *in situ* and the photoreaction was conducted after replacing fluoroform with ambient air. Gratifyingly, this system enabled the oxidative trifluoromethylation using fluoroform for a series of aromatic compounds, including heteroaromatics such as caffeine and pyrroles, to afford trifluoromethylated arenes **35–38**.

We found that the TX used as the mediator or catalyst in the trifluoromethylation can be recovered in significant quantities. This inspired us to develop a mediator-recycling protocol for the trifluoromethylation (Figure 3c). Specifically, we targeted the conversion of methyl methacrylate (MMA),^34^ a bulk chemical (e.g., Sigma-Aldrich, $2.6/mol) used in a wide range of applications. We hypothesized that the hydrotrifluoromethylation of MMA followed by a simple hydrolysis step could afford the corresponding trifluoromethylated product (**39**), which is a high-value carboxylic acid (e.g., Combi-Blocks, $10,000/mol) that has been used as a synthetic intermediate for an anticancer agent,^35,36^ a γ-secretase inhibitor,^37,38^ and a leukotriene antagonist,^39,40^ together with the recovery of TX for recycling. Notably, while the previously reported fluoroform activation strategy can only generate the trifluoromethyl anion, which undergoes nucleophilic addition to the carbonyl group of MMA to afford the corresponding vinyl ketone,^41^ our newly developed radical generation system successfully delivered trifluoromethylated product **39** in a synthetically useful yield (∼65%) with nearly quantitative TX recovery. It is worth noting that the reaction proceeded with a limited amount of fluoroform (5 equiv. relative to MMA) without requiring the supply of an excess via a balloon or high-pressure reactor, and that the recovered TX exhibited an activity identical to that observed in the initial run. These results represent a practical synthetic application that enables the upgrading of bulk chemicals and fluoroform into value-added compounds via radical trifluoromethylation.

### Mechanistic Studies

We conducted mechanistic experiments to investigate the developed radical trifluoromethylation process. To examine the potential role of TX as a triplet energy-transfer agent,^42^ we monitored the formation of hydrotrifluoromethylation product **2** using one equivalent of the fluoroform-TX adduct **1** in the presence and absence of additional TX (Figure 4a). NMR experiments indicated that the rate of trifluoromethylation decreases significantly when an additional 0.1 equiv of TX are added, suggesting that TX is unlikely to function as a triplet energy-transfer agent in the photoreaction process. To determine which reaction intermediate absorbs light, we performed a UV–vis analysis of solutions of the reaction components in DMSO (Figure 4b). The solution of **1** showed no strong absorbance in the 350–500 nm range, while a solution of KO^*t*^Bu alone exhibited absorbance in the visible to UV region, which is characteristic of the dimsyl anion formed upon deprotonation of DMSO.^43,44^ The mixture of **1** and KO^*t*^Bu produced a distinct new absorbance band at 350–400 nm. The mixed solution was analyzed via high-resolution mass spectrometry (electrospray ionization), which revealed the formation of the corresponding alkoxide from **1**, together with dimeric and trimeric potassium alkoxide species (for details, see the Supporting Information, Section 9). These results suggest that the *in situ* generated alkoxide of **1** is likely the intermediate that undergoes photoexcitation. Furthermore, we conducted control experiments to investigate the intermediacy of radical species formed during the reactions. Radical-trapping experiments using **1** and 2,2,6,6-tetramethylpiperidine-1-oxyl (TEMPO) in the presence of KO^*t*^Bu afforded trifluoromethylated product **40**, implying that the trifluoromethyl radical is generated from the alkoxide of **1** (Figure 4c). The reaction with 1-hexene under otherwise identical conditions that provide **2** (Figure 2b) did not yield the hydrotrifluoromethylation product but instead produced numerous unidentified side-products (Figure 4d). One of these products was isolated and identified as alkene-insertion product **41**, which suggests that a secondary alkyl radical intermediate is generated via the addition of a trifluoromethyl radical to the alkene moiety. Subsequent radical–radical coupling between this intermediate and the **TX** radical anion occurs, rather than an SET mechanism leading to hydrotrifluoromethylation (**e** to **a** in Figure 2c), likely due to the highly negative reduction potential of alkyl radicals compared to the TX radical anion.^45^ A computational analysis indicated that **2** and **41** are both formed through alkyl radical intermediate **I**, which is generated after the addition of the trifluoromethyl radical to the alkene (Figure 4e). According to the analysis, the benzylic radical intermediate derived from 1,1-diphenylethylene **Ia** undergoes a facile SET process with the TX radical anion **TX** ^**•–**^ through the minimum energy crossing point (MECP) with a low barrier (Δ*G* ^‡^ = 0.5 kcal/mol) to form the corresponding benzylic anion **IIa**. The subsequent nucleophilic addition of **IIa** to **TX** to afford the coupled intermediate **IIIa** would be unfavorable due to the endergonic nature of this process, with the free energy increasing from −9.6 to 0.4 kcal/mol. Thus, subsequent protonation of **IIa** takes place to give **2** together with the recovery of **TX**. In stark contrast, the analysis showed that alkyl radical intermediate **Ib**, derived from 1-hexene, undergoes C–C bond formation with **TX** ^**•–**^ through a MECP (Δ*G* ^‡^ = 7.3 kcal/mol), leading to intermediate **IIIb** (Δ*G* = −26.4 kcal/mol), rather than participating in a single electron-transfer event to form the energetically unfavorable carbanion intermediate **IIb** (Δ*G* = 4.0 kcal/mol). Subsequent protonation of **IIIb** affords the experimentally observed product **41**. These calculations thus support the proposed mechanism involving radical intermediates and are consistent with the effective regeneration of TX during the reaction.

**Figure 4:**
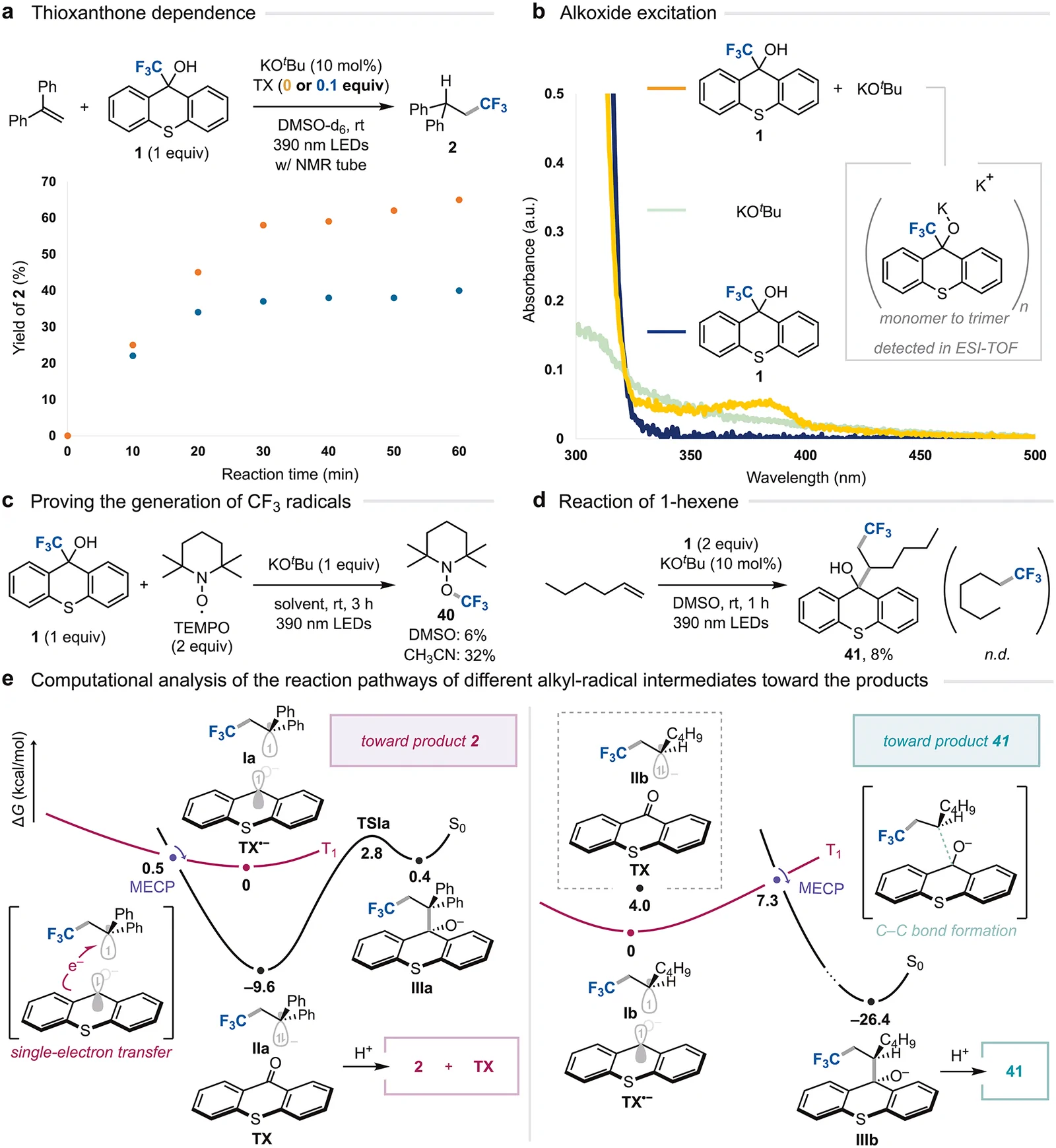
Mechanistic studies. (a) Hydrotrifluoromethylation to 2 in the presence or absence of TX. (b) UV–vis spectra of solutions of 1, KO*^t^* Bu, and 1 + KO*^t^* Bu in DMSO. (c) Control experiments involving the trapping of trifluoromethyl radicals with TEMPO. (d) Reaction of 1-hexene to probe the intermediacy of the alkyl radical and ketyl radical anion. (e) Computational reaction pathways from radical intermediates; computations were performed at the (U)­ωB97X-D/6-311+G­(2d,p)/CPCM­(DMSO)//(U)­ωB97X-D/6-31+G­(d)/CPCM­(DMSO) level of theory; for details, see the Supporting Information.

## Conclusions

The developed system enables the direct use of fluoroform in radical trifluoromethylation. Trifluoromethyl radicals can be generated from fluoroform under mild conditions and subsequently be incorporated into various unsaturated molecules. The photocatalytic ability of ketones was exploited to develop a catalytic radical trifluoromethylation process employing fluoroform with excellent atom economy. The use of fluoroform is becoming increasingly important, not only for synthetic purposes, but also for the preparation of radiolabeled compounds.^46^ This work broadens access to high-value trifluoromethylated compounds from fluoroform, thereby advancing an economically and environmentally attractive strategy for the sustainable valorization of an industrial waste product.

## Supplementary Material



## Notes

Details regarding experimental procedures, materials, calculations, and spectroscopic data (PDF)

## ||

Research Center for Materials Science and Department of Chemistry, Graduate School of Science, Nagoya University, Furo-cho, Chikusa-ku, Nagoya 464-8602, Japan
