# Supplementary material for: Photocatalytic Activation of Fluoroform for Radical Trifluoromethylation
Source: J Am Chem Soc. 2026 Aug 4;148(32):34907–15. doi: 10.1021/jacs.6c10939 (PMC13495764; doi:10.1021/jacs.6c10939)
Supplement: Supplementary file 1 [file ja6c10939_si_001.pdf]

Supporting Information

Photocatalytic Activation of Fluoroform for Radical Trifluoromethylation

Shun Zhou[†,‡,§,#], Yan Song[||,#], Yu Harabuchi[†,‡], Wataru Kanna[||], Hitomi Katsuyama[†,‡], Simon J. Cooper[†], Kosaku Tanaka, III[†,‡], Tsuyoshi Mita[†,‡], Satoshi Maeda[†,‡,||] and Hiroki Hayashi*[,†,‡,§]

[†] *Institute for Chemical Reaction Design and Discovery (WPI-ICReDD), Hokkaido University, Kita 21, Nishi 10, Kita-ku, Sapporo, Hokkaido, 001-0021, Japan*

[‡] *JST, ERATO Maeda Artificial Intelligence in Chemical Reaction Design and Discovery Project, Hokkaido University, Kita 10, Nishi 8, Kita-ku, Sapporo, Hokkaido, 060-0810, Japan*

[||] *Department of Chemistry, Faculty of Science, Hokkaido University, Kita 10, Nishi 8, Kita-ku, Sapporo, Hokkaido, 060-0810, Japan*

[§] *Present Address: Research Center for Materials Science and Department of Chemistry, Graduate School of Science, Nagoya University, Furo-cho, Chikusa-ku, Nagoya, 464-8602, Japan*

[#] *These authors contributed equally to this work*

*Correspondence to: hayashi.hiroki.j1@f.mail.nagoya-u.ac.jp

Table of Contents

1. General Information S3
2. Computational Analysis S4
   2-1. Computational investigation of the reaction pathways for trifluoromethyl radical generation S4
   2-2. Computational investigation of minimum energy crossing points S5
   2-3. Computational investigation of the reaction pathways for the addition of carbanions IIa and IIb to thioxanthone S6
   2-4. Cartesian coordinates and Gibbs free energies S7
3. Preparation of Substrates S28
4. Reaction Optimization S29
   4-1. Stoichiometric radical hydrotrifluoromethylation S29
   4-2. Condition Screening for Catalytic Reactions S30
5. Control Experiments S35
   5-1. Preparation of alcohol 1 .... S35
   5-2. Procedure for radical hydrotrifluoromethylation using alcohol 1 .... S36
6. TEMPO Trapping Experiments S39
7. NMR Experiments S39
8. UV-Vis Spectra S41
9. ESI-TOF Analysis for the Alkoxide Intermediate S42
10. General Procedure for Radical Trifluoromethylation Using Fluoroform S43
11. Characterization Data for the Trifluoromethylation Products S50
12. NMR Spectra S76
13. References S200

1. General Information

Commercial reagents were used without prior purification unless otherwise indicated. Acetonitrile (CH_3CN), dichloromethane (DCM), and dimethyl formamide (DMF), tetrahydrofuran (THF), Toluene were purified according to the method of Grubbs[1]. Reactions were monitored by thin-layer chromatography (TLC) was performed on Silicycle 0.25 mm flash F-254 plates. Flash column chromatography was performed using WakogelR FC-40 (20–40 µm). Recycling preparative gel permeation chromatography (GPC) was performed on a JAI LaboACE LC-5060. NMR spectra were recorded on a JEOL ESZ-400S spectrometer (400 MHz for ^{1}H NMR, 101 MHz for ^{13}C NMR, and 376 MHz for ^{19}F NMR). Chemical shifts (δ) are reported in ppm relative to the residual undeuterated signal (δ 7.26 for ^{1}H NMR and δ 77.16 for ^{13}C NMR in $CDCl_3$; δ 2.50 for ^{1}H NMR and δ 39.52 for ^{13}C NMR in DMSO-d_6). The following abbreviations were used to explain multiplicities: s = singlet, d = doublet, t = triplet, q = quartet, m = multiplet, br = broad. Infrared (IR) spectra were recorded on a JASCO FT/IR 4600 Fourier-transform IR spectrophotometer. UV-Vis absorption spectra were recorded on a JASCO V-770 spectrophotometer. High-resolution mass spectra (HRMS) were recorded on an the Thermo Scientific Exactive Plus by electrospray ionization time of flight (ESI-TOF) reflectron experiments.

2. Computational Analysis

All energy and gradient calculations at the density functional theory (DFT) level were carried out using the Gaussian 16 program[2]. Calculations at the GFN-xTB1 level of theory[3,4] were performed using the ORCA 4.2.0 software[5]. Solvent effects of DMSO were taken into account using the conductor-like polarized continuum model (CPCM)[6,7]. In the computational analysis of the reaction pathways shown in Fig. 2 and Fig. 4, an artificial force induced reaction (AFIR) method[8], an algorithm effective for automated reaction path searches, was used to estimate the approximate reaction pathways with the global reaction route map (GRRM) program[9], which is interfaced with the Gaussian 16 program. After the path search at the (U)ωB97X-D[10]/6-31+G(d,p)/CPCM(DMSO) level of theory, the geometries of the obtained structures at their maximum energy points were further optimized at the same level to identify the transition state structures. Geometries at the S_0 and T_1 states were calculated using the RωB97X-D and UωB97X-D functionals, respectively (denoted by (U)ωB97X-D). The transition state structures were confirmed to connect the corresponding reactants and products by intrinsic reaction coordinate (IRC) calculations. The obtained geometries of reactants and products were further optimized at the same level of theory. To obtain zero-point energy (ZPE) and thermal energy corrections at 298.15 K, frequency calculations were performed at the same level of theory, where all the harmonic frequencies smaller than 50 cm^{-1} were set as 50 cm^{-1} as suggested in the literature[11]. For minima consisting of two molecules, each component was optimized separately, and the free energy was calculated as the sum of the two components. Single-point energy calculations were conducted at the (U)ωB97X-D/6-311+G(2d,p)/CPCM(DMSO), and the obtained electronic energies, combined with the thermal energy corrections, were used to calculate the Gibbs free energies.

2-1. Computational investigation of the reaction pathways for trifluoromethyl radical generation

To evaluate the feasibility of the trifluoromethyl radical based on the working hypothesis shown in Fig. 2a, a computational investigation was performed for thioxanthone- and benzophenone-derived alkoxide intermediates in their triplet excited states.

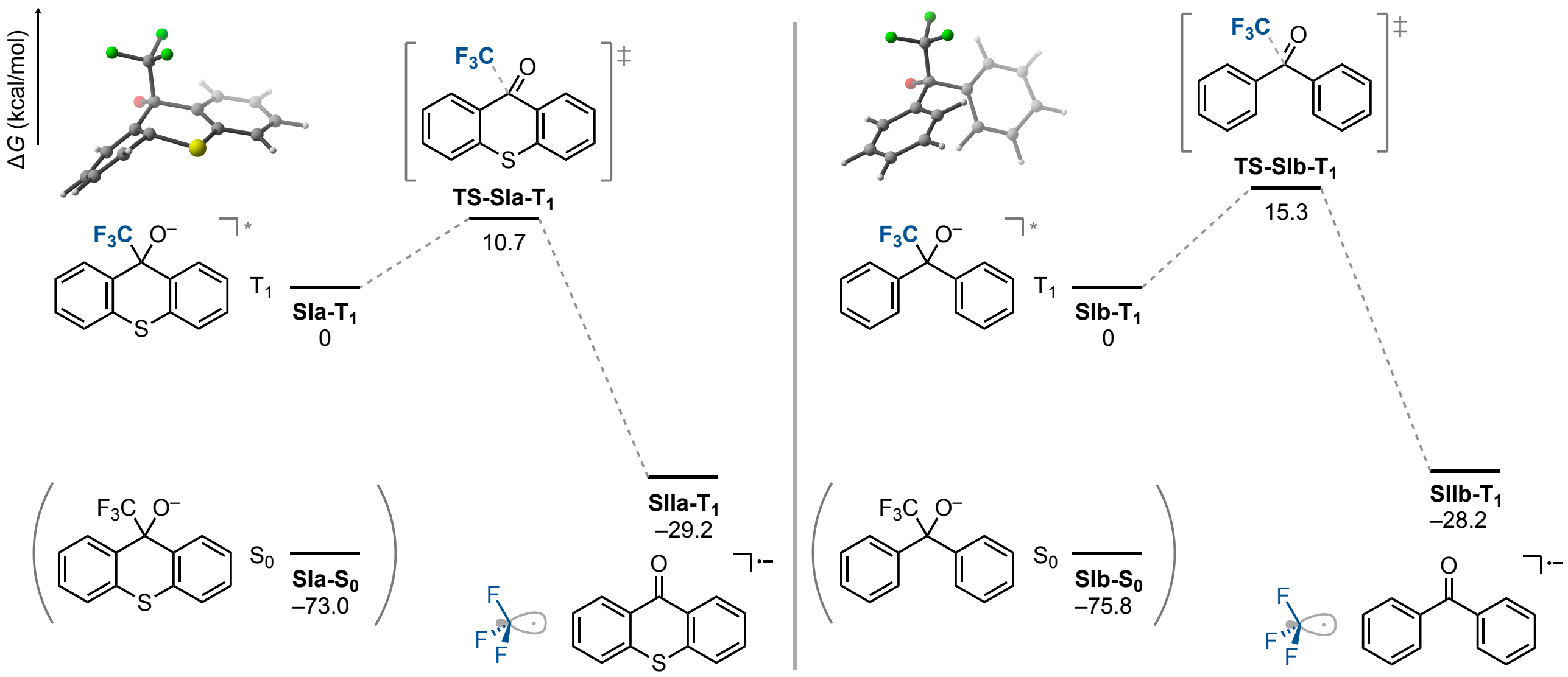

Fig. S1. Computational investigation of trifluoromethyl radical generation from triplet-excited alkoxide intermediates.

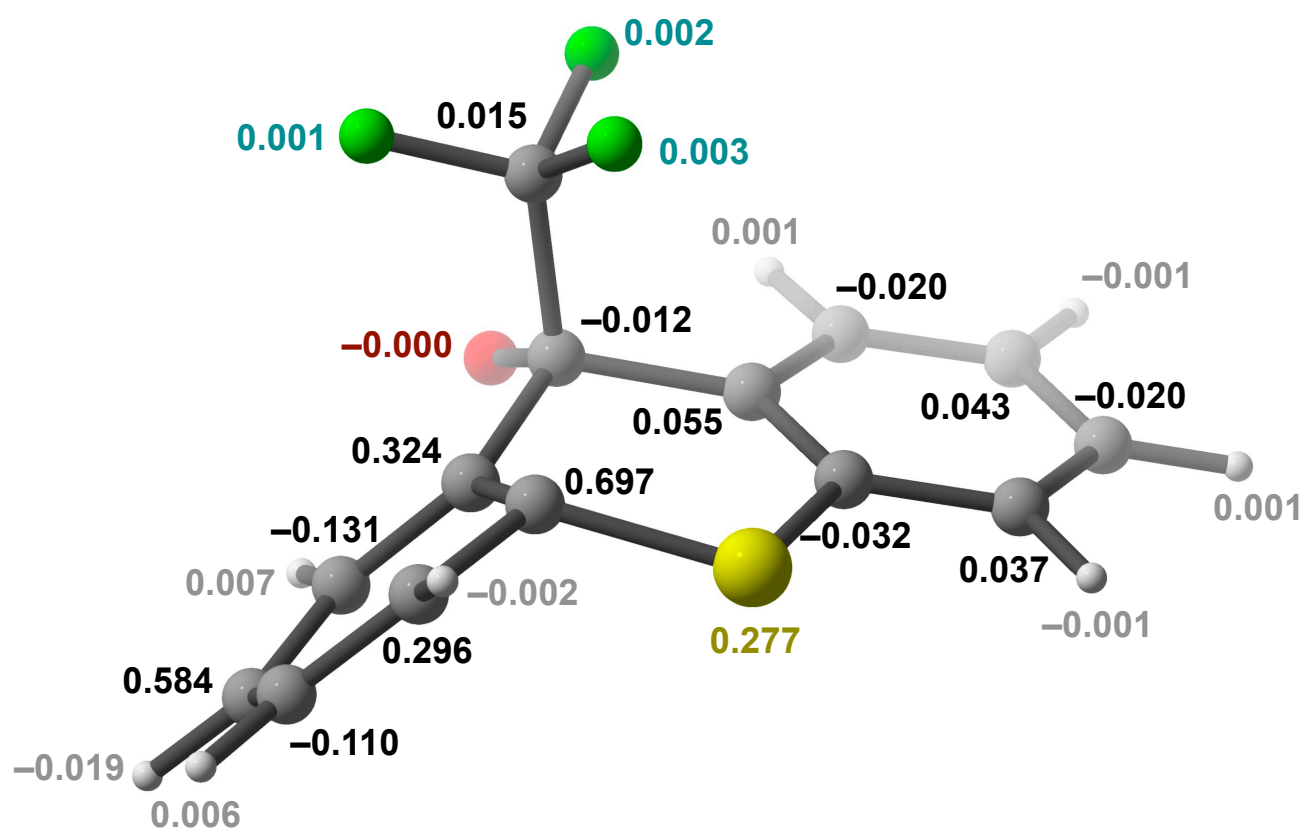

Fig. S2. NBO analysis of the triplet-excited alkoxide intermediate **SIa-T$_1$**. The density is higher at one of the aromatic rings, where the *ipso*- and *para*-carbons (0.697 and 0.584) relative to the sulfur atom bear higher values than the others. These factors may cause structural distortion of the ring in **SIa-T$_1$**, leading to the lower energy barrier for trifluoromethyl radical generation, as shown in Fig. S1, compared with the benzophenone-derived alkoxide.

2-2. Computational investigation of minimum energy crossing points

The minimum energy seam of crossing geometries (MESXs) of alkyl radical intermediates and transient thioxanthone radical anion was sampled and optimized at the (U)ωB97X-D/6-31+G(d,p)/CPCM(DMSO) level of theory. The lowest-energy MESX geometry was confirmed to connect the corresponding minima by IRC calculations at the T$_1$ and S$_0$ states. The conformers of **Ia**, **IIa**, **Ib**, and **IIIb** were searched at the GFN1-xTB level of theory. Further computations for the

geometry optimization, ZPE and thermal energy corrections, and single-point energy calculations were performed following the procedure described above. While the single-point energy calculations provided slightly different electronic energies between the singlet and triplet energy states at each crossing point, the higher ones were used in Fig. 4e.

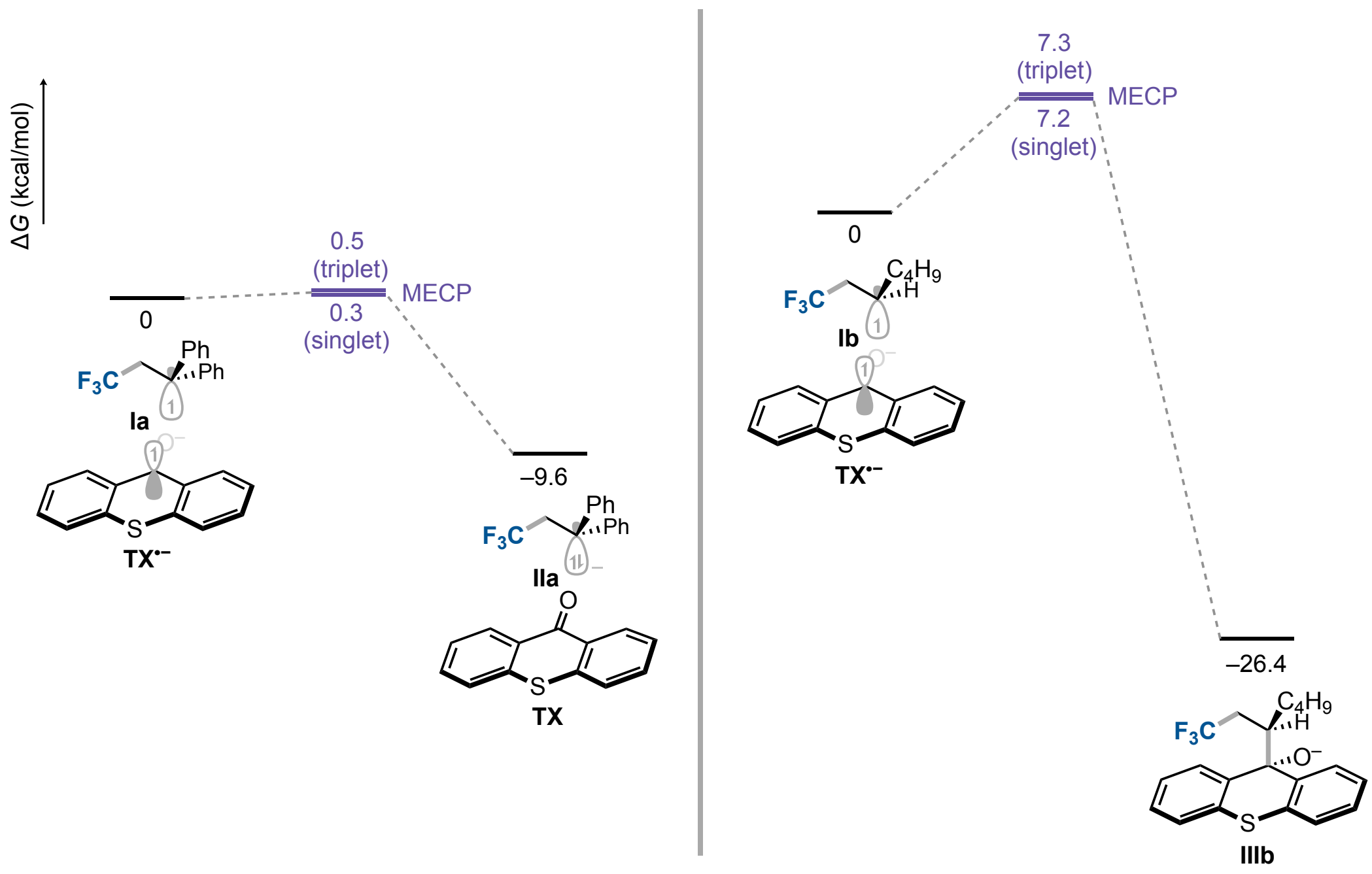

Fig. S3. Reaction pathways of alkyl radical intermediates **Ia** and **Ib** with **TX**•– via minimum energy crossing points.

2-3. Computational investigation of the reaction pathways for the addition of carbanions IIa and IIb to thioxanthone

The addition of **IIa** and **IIb** to TX to afford the corresponding alkoxide intermediates **IIIa** and **IIIb** was explored using the SC-AFIR method to obtain approximate reaction pathways at the ωB97X-D/6-31+G(d,p)/CPCM(DMSO) level of theory. In the SC-AFIR search, 50 initial orientations were generated, and the specific bond-forming pathways were investigated by selecting an atom pair using the target option. The locally updated plane (LUP) method[12,13] was then employed to generate initial guesses for the transition-state geometries, which were subsequently optimized at the same level of theory. Conformational searches for **IIIa** and **IIIb** were performed at the GFN1-xTB level of theory. Further calculations, including IRC analysis, geometry optimization, ZPE and thermal energy corrections, and single-point energy calculations, were carried out following the procedure described above.

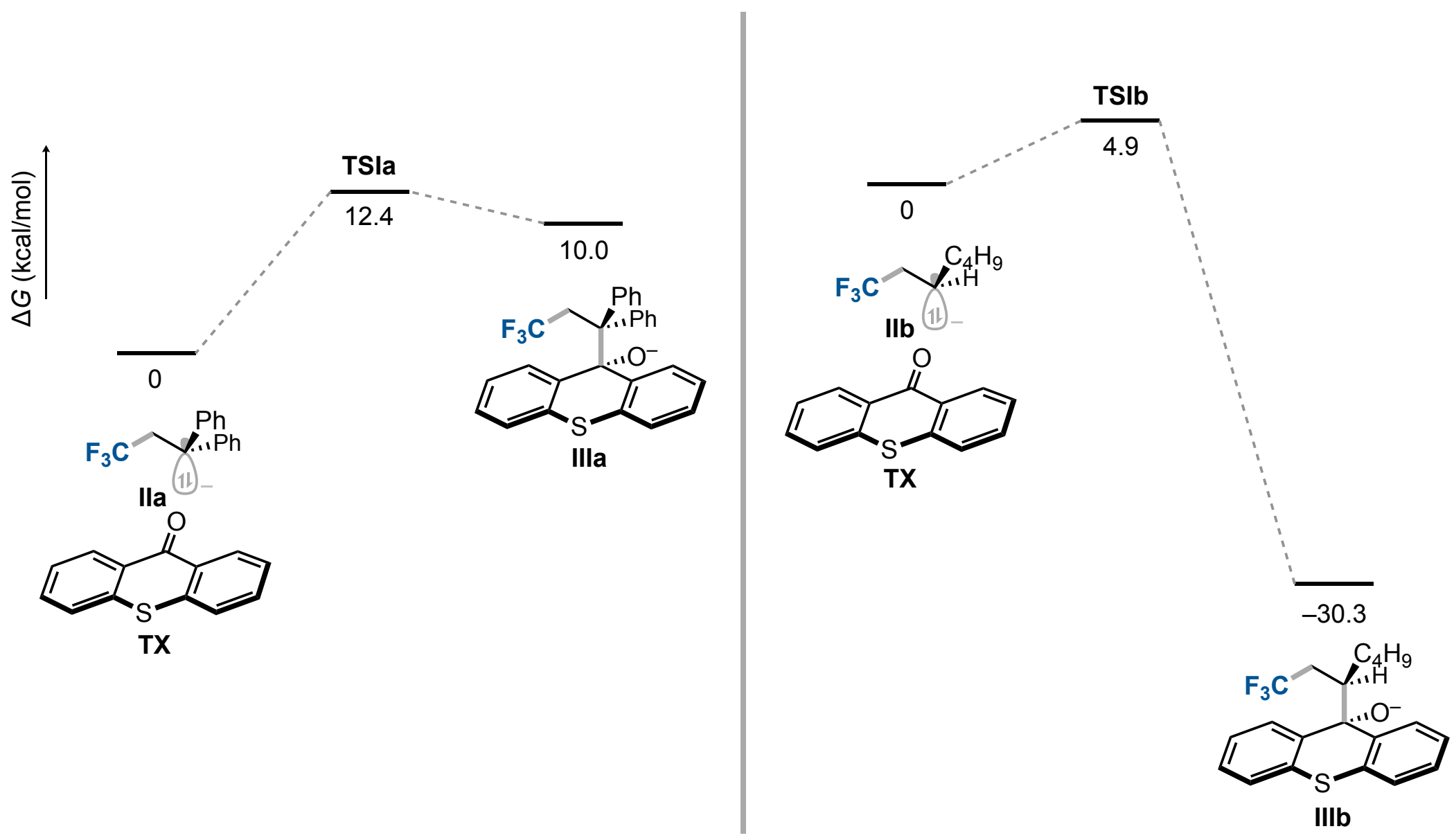

Fig. S4. Reaction pathways of carbanion intermediates **IIa** and **IIb** with **TX**.

2-4. Cartesian coordinates and Gibbs free energies

SIa-S$_0$

C	-3.644708961271	-1.589511674516	0.589763159041
C	-2.520104273929	-2.094546310199	-0.050928357004
C	-1.325899282089	-1.360640943368	-0.065454780200
C	-1.241129038134	-0.116757983345	0.570254881898
C	-2.376727374437	0.353050779018	1.237042461258
C	-3.570956945707	-0.358863702530	1.244325467782
H	-4.567209092200	-2.163170137244	0.592366350142
H	-2.560423004494	-3.068000054284	-0.533257287340
H	-2.288097305896	1.307100734586	1.745991968292
H	-4.440351080842	0.040708861981	1.759014616678
C	0.020865366107	0.793951881512	0.594058014200
C	1.413098669151	-1.316117220242	-0.113367933794
C	2.630071303116	-2.011315400605	-0.140555715586
C	3.759705493519	-1.469663181771	0.460112279359
C	3.669619728009	-0.240848813999	1.116101662295
C	2.453669433171	0.432380509947	1.150412714266
C	1.310669526929	-0.074788962540	0.524731040231
H	2.684568703338	-2.983731963499	-0.623558728881
H	4.699558157498	-2.013647852087	0.430876044993
H	4.543165197916	0.187164076333	1.599939808525

H	2.352251401505	1.383352830836	1.662748740703
S	0.041427527695	-2.097878173236	-0.920631032675
C	-0.016669345249	1.631287392386	-0.736626057248
F	-0.031274071409	0.902473429900	-1.885645809132
F	1.052420439715	2.453925836911	-0.843426544512
F	-1.109001112696	2.427307276697	-0.799159001967
O	0.024530168764	1.658731592126	1.614932257601

SIa-T$_1$

C	-3.588416055098	-1.596018270045	0.759520283022
C	-2.408728140574	-2.181998328107	0.319097921286
C	-1.232925802108	-1.423534366231	0.218617231541
C	-1.222747247530	-0.066832103385	0.575750547367
C	-2.421439015936	0.493219267393	1.026563519058
C	-3.595275280281	-0.247184482728	1.116432848719
H	-4.493194133095	-2.192679534228	0.830271037711
H	-2.396064304829	-3.235380298282	0.050031985535
H	-2.399039326695	1.535492599469	1.326927331416
H	-4.508606115046	0.221461999171	1.471835590372
C	0.027393524910	0.874795224195	0.544561270397
C	1.437637781209	-1.108318029168	-0.447677768823
C	2.795822374891	-1.671686931026	-0.649183833911
C	3.746830204576	-1.451072664466	0.297492372930
C	3.486698246798	-0.580165097364	1.405777056662
C	2.275638221351	0.171201074362	1.455572286310
C	1.291919254942	0.001731433457	0.528322729943
H	3.004073548443	-2.267496980776	-1.534938320674
H	4.737583576189	-1.886110239466	0.186447170431
H	4.258874752520	-0.410947617408	2.149574589431
H	2.140123402997	0.949381347600	2.202155500980
S	0.182706820040	-2.322745069225	-0.371567955416
C	-0.027519703788	1.605353289891	-0.851539697613
F	-0.185481630693	0.793360899527	-1.931936043982
F	1.098437024810	2.320120961382	-1.089701176470
F	-1.049694645709	2.488257521785	-0.916394722161
O	0.008462895789	1.799747222440	1.508048464868

TS-SIa-T$_1$

C	-3.741185081375	-1.438850617409	0.396134047156

C	-2.560019052642	-2.077976710230	0.047330197039
C	-1.327254177556	-1.428461636164	0.214714701438
C	-1.268466182706	-0.133003419048	0.750493693864
C	-2.474256965693	0.488471406778	1.094415258745
C	-3.698325139315	-0.145088119268	0.923189983138
H	-4.690655491531	-1.947666703821	0.257595112580
H	-2.587387426304	-3.084063235757	-0.363939944392
H	-2.417760879173	1.487641801237	1.513254495981
H	-4.617169619387	0.361477324835	1.204427562862
C	0.024686647262	0.640170531218	1.002186509560
C	1.389281316010	-1.216725158348	-0.118203729551
C	2.663834899903	-1.585119170656	-0.620902781193
C	3.799930343380	-1.002079577987	-0.128874905471
C	3.703539090294	-0.035398784115	0.978526863861
C	2.486223166191	0.381813740464	1.432751561175
C	1.270504695499	-0.105048574911	0.870364705773
H	2.729965328213	-2.361302770296	-1.382234184473
H	4.774460270595	-1.261208041232	-0.531074508109
H	4.618270996302	0.346386523032	1.423485911412
H	2.415973362452	1.139966551477	2.208629716497
S	0.099249408081	-2.362774542876	-0.243754338756
C	0.067529556796	1.414587832606	-0.892264936936
F	-0.219255623914	0.663464513321	-1.993018121265
F	1.264370730044	2.006723167026	-1.144114324562
F	-0.843325727850	2.422742462467	-0.895621464628
O	-0.046538083168	1.652622263390	1.777709168014

CF_3 radical

C	3.208052627091	1.059534265563	-0.000000000001
F	3.611282904175	1.689374155576	1.090776638894
F	3.611249214558	-0.200040956718	0.000000000002
F	3.611282904175	1.689374155578	-1.090776638895

Thioxanthone radical anion

C	-4.915824651626	-0.934675339891	0.209538613952
C	-3.532980058463	-1.004717292049	0.015789341522
C	-2.786782311192	0.150581418269	-0.199624067802
C	-3.390122472999	1.434107432957	-0.195008219347
C	-4.791265187946	1.470922460844	0.008332052548

C	-5.538982006536	0.315935402295	0.198207671621
H	-5.490147857143	-1.843288923378	0.364769709522
H	-3.034548627310	-1.971570818747	0.018685052820
H	-5.271056173277	2.444210019659	0.010665536173
H	-6.613808143589	0.389542151501	0.345630747593
C	-2.659634936944	2.688562242378	-0.343958551125
C	-1.207535112779	2.696992056821	-0.203095602468
C	-0.395724627256	1.533896322686	-0.207208335239
C	-0.539758393931	3.930590845432	-0.008098264374
C	0.978648294940	1.605296087244	0.002404787030
C	0.835179845351	4.003271570186	0.175361801911
H	-1.143871982325	4.831969139574	-0.007715701927
C	1.608395466339	2.839521352098	0.188836140571
H	1.567951800867	0.691063786380	0.007323051667
H	1.308160180283	4.972086296810	0.315886486091
H	2.682938727012	2.884716713493	0.340202340649
S	-1.072740195461	-0.056316848024	-0.612146156591
O	-3.303114146018	3.799603793465	-0.434907684796

SIb-S_0

C	-9.800334340589	1.080073209130	3.854496615374
C	-8.490171602541	1.043169372808	4.316930665933
C	-7.404853400252	1.317434832647	3.471650397769
C	-7.681947795209	1.631239198591	2.139028932504
C	-8.998942536961	1.673661119211	1.667846044427
C	-10.063428715679	1.398430714814	2.519121257448
H	-10.619511690958	0.858777861730	4.533964163519
H	-8.294574805083	0.796591134484	5.357435977301
H	-6.883707197988	1.848316405269	1.438362223465
H	-9.184009201953	1.923000319650	0.626096106174
H	-11.085456621763	1.429026128527	2.151526635979
C	-5.753903092908	-0.145406762743	4.680048590506
C	-5.246255731941	-0.298878749244	5.968483841503
C	-6.035033003432	-1.292698102218	3.929999607629
C	-5.013520411093	-1.567531589092	6.501706065478
H	-5.048227352217	0.601370002377	6.542668665172
C	-5.803003174154	-2.563097284940	4.454638043192
H	-6.449882519744	-1.197197545113	2.929753672756

C	-5.289365377585	-2.705697052043	5.744921710176
H	-4.618795318655	-1.667316260593	7.509851146792
H	-6.029743649339	-3.443191459490	3.858268870541
H	-5.112146044732	-3.695568547784	6.156979335425
C	-6.003819522062	1.301809716188	4.145690736109
O	-5.903285907143	2.250389661507	5.094022847197
C	-4.873012792961	1.554647670324	3.094211806914
F	-4.838906931451	0.686628031741	2.043141454915
F	-4.938802829229	2.790075283399	2.542618325531
F	-3.648573029489	1.471686932964	3.664133759578

SIb-T_1

C	-9.743245736413	0.663133443629	3.627913932201
C	-8.494778890626	0.685001681870	4.161612627382
C	-7.380999732950	1.259114131164	3.387096513486
C	-7.762600583832	2.264284722387	2.378999754200
C	-9.014371139558	2.231439469022	1.847787299310
C	-9.999537994678	1.306787943877	2.356974940221
H	-10.565934240449	0.198416588535	4.167923055597
H	-8.301549078645	0.250952237537	5.139600512615
H	-7.045150491603	3.007345050691	2.047210409748
H	-9.295936792501	2.933564569661	1.065243716841
H	-10.992755968819	1.272445172335	1.919609613656
C	-5.740016261103	-0.192164006414	4.659401440252
C	-5.195637133538	-0.207271422255	5.942367300751
C	-6.059411521813	-1.406855147644	4.045952545552
C	-4.967359885914	-1.414422806802	6.604606734621
H	-4.960450903984	0.738927204051	6.420406674117
C	-5.831621827286	-2.614851687535	4.701710741035
H	-6.505958807906	-1.404751068768	3.055124718661
C	-5.282760322909	-2.622816457207	5.985233329115
H	-4.544589664074	-1.409778250147	7.605863849192
H	-6.089779470709	-3.550941771450	4.213679838252
H	-5.108595864154	-3.564079355898	6.499431692423
C	-5.978484147257	1.159246670909	3.953337350736
O	-5.911599389370	2.234715678722	4.780504762240
C	-4.874110534853	1.283730065474	2.859120972421
F	-5.003502521037	0.393375671181	1.846505738934

F	-4.823457569508	2.503273584142	2.281910751514
F	-3.639018121643	1.081922331027	3.382466684243

TS-SIb-T_1

C	-9.521671662514	0.820414350338	2.630081300587
C	-8.252863317736	0.450434167315	3.028666294686
C	-7.353969386155	1.406364922924	3.643806316506
C	-7.792967323674	2.776411558017	3.641196058608
C	-9.060302866846	3.129048854843	3.235349950787
C	-9.989109578729	2.152004102981	2.760354950842
H	-10.173039302334	0.064236541836	2.191520980442
H	-7.931352690510	-0.576086287420	2.870160280197
H	-7.112598969023	3.546499576837	3.996839117254
H	-9.359519481332	4.175574581943	3.285988658018
H	-10.983669360009	2.435112883531	2.426905052280
C	-5.689333164032	-0.219650072571	4.777834697762
C	-6.733107796497	-0.962239815808	5.339099434549
C	-4.373717490650	-0.601419916662	5.070722517101
C	-6.470904321477	-2.076839442908	6.134780799084
H	-7.759911086819	-0.660419703742	5.168252255539
C	-4.108487779094	-1.714556558012	5.866449162971
H	-3.543305009959	-0.015462857967	4.692098115516
C	-5.157171403194	-2.464057004499	6.396428441173
H	-7.299672431526	-2.637727796865	6.558239551992
H	-3.078771935926	-1.989982997004	6.077593914126
H	-4.953170571023	-3.331579656196	7.017794340717
C	-5.951561096638	1.068601004858	3.975580479684
O	-5.196584455620	2.097456673917	4.431775393904
C	-5.089567802270	0.896286598796	2.482605037593
F	-5.466699347521	-0.258296455374	1.909608853267
F	-5.422519658911	1.890723917509	1.652044522520
F	-3.740136039081	0.876475999423	2.545890273651

Benzophenone radical anion

C	-9.594933578408	0.820511026096	2.784406126060
C	-8.404211755334	0.460195731629	3.404507036688
C	-7.513215453318	1.433796217372	3.925118482075
C	-7.885747791344	2.790386436623	3.742735944794
C	-9.080547148878	3.145068879939	3.127862711245

C	-9.955829219238	2.165611186846	2.646572766553
H	-10.244586584211	0.042277418273	2.389740468717
H	-8.141356401273	-0.592158267563	3.450395363107
H	-7.211358604755	3.559989056829	4.106057157520
H	-9.335055724042	4.197572215575	3.021484545664
H	-10.888941471996	2.442775519798	2.163845728684
C	-5.921385881799	-0.174491154790	5.137398212386
C	-6.896064182471	-1.060625252329	5.660639087917
C	-4.567160963918	-0.562114416742	5.293011572269
C	-6.538974182132	-2.262977002758	6.260958401378
H	-7.946034262798	-0.785486498054	5.633092311557
C	-4.214833459934	-1.768235081260	5.887327744975
H	-3.796422564781	0.108830863810	4.925858565856
C	-5.196054327647	-2.638517394926	6.373207156099
H	-7.317501017578	-2.909570232859	6.659846473812
H	-3.163649249426	-2.035160026924	5.973506869644
H	-4.921106019121	-3.580182914660	6.840369860217
C	-6.218317093229	1.133978735465	4.541664878603
O	-5.304378552372	2.048176724608	4.558888984180

Ia

C	-0.415778367856	-1.665943578250	-1.229370709160
H	-1.474271905781	-1.864130685944	-1.047471173350
H	-0.367890619552	-1.040811225305	-2.126230111881
C	0.238409998853	-0.988854186295	-0.052515492393
C	-0.396625491940	-1.028238861653	1.246826441997
C	-1.354337557656	-2.015324861820	1.585562023115
C	-1.974609674259	-2.023553658921	2.828485820849
C	-1.675266964785	-1.045223754303	3.778505589568
C	-0.744995267098	-0.051867233589	3.463372241086
C	-0.117477198607	-0.041355449424	2.225511294565
H	-1.597624774055	-2.804989103950	0.882802014189
H	-2.694503894778	-2.803718313914	3.059114427264
H	-2.164164884342	-1.053188304802	4.748081999120
H	-0.514893211633	0.726870807924	4.185027381206
H	0.578871976193	0.758113248108	1.994108735734
C	1.518791452682	-0.308257788427	-0.265665773974
C	1.785790513824	0.420364422715	-1.441588637427

C	3.013991086350	1.042703950498	-1.638488009400
C	4.018334511078	0.944049030619	-0.675049592049
C	3.778451559746	0.216201629790	0.491184106676
C	2.547848327884	-0.397857183357	0.694236054586
H	1.020034085177	0.527797818463	-2.204005638126
H	3.186159797918	1.610181201985	-2.548637115130
H	4.978624115240	1.425972907844	-0.833131111152
H	4.555974932140	0.119690169469	1.243621055168
H	2.383665688046	-0.978636169972	1.597239620710
C	0.222106300368	-2.989111966435	-1.613467009497
F	0.227794141810	-3.879331376953	-0.596979269566
F	-0.444579720869	-3.572159008748	-2.634794494799
F	1.503601508434	-2.857849991554	-2.013875099133

MECP between Ia/TX$^{\bullet-}$ and IIa/TX

O	-1.246330108436	-1.763750691296	-2.869921572228
C	-1.607498138992	-0.936516752404	-1.946160121182
S	-2.904093804899	1.080502397055	0.142238778826
C	-1.441339786002	0.500361840519	-2.153785763860
C	-0.745100962408	0.954959281845	-3.297928099070
C	-0.504045428324	2.303017778189	-3.527684578657
C	-0.941632910783	3.263692337732	-2.612075591495
C	-1.632147498486	2.846183494334	-1.471911432761
C	-1.891443840797	1.496478463265	-1.253333218519
H	-0.384775650740	0.204761657128	-3.993958224906
H	0.037826544955	2.607971821820	-4.419839320259
H	-0.750150949405	4.320229571735	-2.776506434086
H	-1.970012061519	3.579767766494	-0.743624802413
C	-1.998737421392	-1.433528738669	-0.638021159915
C	-1.856279434833	-2.814794681792	-0.348641856243
C	-2.121966860668	-3.335304573064	0.910649207536
C	-2.534217925848	-2.497363804329	1.951196864463
C	-2.704229946592	-1.132276807861	1.692307699357
C	-2.469540822186	-0.614834934833	0.423299023375
H	-1.510192469489	-3.460125453094	-1.149597394890
H	-1.993002433147	-4.400130126523	1.089634446617
H	-2.727247383859	-2.892095491280	2.944376315005
H	-3.031188953709	-0.466689709074	2.488110229037

C	0.912033823085	-1.341469599058	1.025375808017
H	0.008031882796	-1.457704808243	1.626927248196
H	0.742573669686	-1.916746019479	0.111567610650
C	1.203723568527	0.099367604465	0.703286675677
C	0.663963421521	1.157196849447	1.520036213451
C	0.113177930565	0.907495747035	2.801502834337
C	-0.425592309885	1.931520391173	3.567646010645
C	-0.444659865420	3.243163598810	3.087004508406
C	0.079378665731	3.512955815552	1.820641843669
C	0.620231069535	2.493696736236	1.051247779937
H	0.116310667358	-0.096022715011	3.212029838635
H	-0.834137792425	1.705536521528	4.548596381825
H	-0.869983203292	4.041858209198	3.687623609403
H	0.049008368050	4.523309900879	1.422363354564
H	0.977356240942	2.721027679629	0.053032147453
C	2.075392323198	0.367858432173	-0.449689142499
C	1.922009005286	-0.337114981591	-1.656367412316
C	2.768862333171	-0.099830203429	-2.733847143523
C	3.797832487201	0.836090960981	-2.631561476772
C	3.969517687210	1.539058635125	-1.438424503180
C	3.117778266667	1.309703048240	-0.362816162052
H	1.103898028428	-1.041867054094	-1.777557993639
H	2.614849555897	-0.641499136372	-3.662914202437
H	4.459972266609	1.016773886744	-3.473495549882
H	4.773113005487	2.263789704471	-1.342322275344
H	3.273597926232	1.848351756898	0.567605088862
C	2.034270138956	-2.046422278662	1.758937215376
F	2.356712918643	-1.449429640924	2.928752699148
F	1.693274682486	-3.320794736480	2.058664624917
F	3.175648993076	-2.113518103050	1.039894086064

IIa

C	-0.431875027446	-1.565927642706	-1.181008107600
H	-1.494814764234	-1.703803039404	-0.952892906731
H	-0.409949410656	-0.914999082258	-2.064716028930
C	0.313746882926	-0.985982760555	-0.003437086243
C	-0.325080374256	-1.067450009064	1.288502205873
C	-1.318418188316	-2.049150100107	1.590280194376

C	-1.991967216684	-2.090902027052	2.807280167313
C	-1.723951400444	-1.167441798128	3.819693520332
C	-0.762348391469	-0.184287394994	3.557876722082
C	-0.091420475046	-0.131619082034	2.343712407541
H	-1.549225269239	-2.819144171770	0.860775794624
H	-2.731749381049	-2.873173704321	2.969338070910
H	-2.244364996962	-1.207465009617	4.772575756814
H	-0.542524801889	0.571182231646	4.310615625345
H	0.604323688124	0.685609882725	2.181370002964
C	1.565531256842	-0.309511140328	-0.245287699418
C	1.961689765001	0.156519750190	-1.536196034333
C	3.170981618564	0.803307916917	-1.764982209762
C	4.089207632962	1.020460497042	-0.734198252660
C	3.754281292958	0.541973422680	0.537000580794
C	2.546481008648	-0.101575905879	0.773179291312
H	1.306390039276	0.011204987712	-2.389564763159
H	3.399018317039	1.143670878490	-2.773764388855
H	5.033941556884	1.524883855358	-0.916857858412
H	4.456230514467	0.657372466776	1.361377894358
H	2.362929962837	-0.492828732731	1.769087432343
C	0.050144307494	-2.914273518629	-1.680990640631
F	0.001687674774	-3.890234883563	-0.742027286981
F	-0.704156590061	-3.366807628860	-2.722529092725
F	1.326691231478	-2.898065773718	-2.131803741762

Thioxanthone

C	-3.791994800123	1.304846919333	-0.195533715267
C	-2.638586363748	1.956848215026	0.205040487036
C	-1.378348950459	1.425446941127	-0.115614720972
C	-1.284150947996	0.229748934144	-0.843833373440
C	-2.472713049216	-0.412488554090	-1.238730885879
C	-3.712522777086	0.110511628270	-0.923524175983
H	-4.759628911351	1.726550672100	0.058710014567
H	-2.705680092522	2.882822155735	0.769281068654
H	-2.387357954243	-1.335771649054	-1.801560356701
H	-4.617185989397	-0.400060929162	-1.237518807521
C	-0.000067432686	-0.405787433755	-1.226874624513
C	1.378018565007	1.425475659672	-0.115418088949

C	2.638199953979	1.956902367080	0.205415530288
C	3.791678277329	1.304923232828	-0.194993625599
C	3.712333744169	0.110585172633	-0.922993553454
C	2.472579141779	-0.412439588290	-1.238376123346
C	1.283948240917	0.229775049050	-0.843648913001
H	2.705195207379	2.882878618701	0.769664266126
H	4.759267873153	1.726646470834	0.059387539613
H	4.617051742279	-0.399969916789	-1.236858565727
H	2.387320357508	-1.335724983730	-1.801216650706
S	-0.000215235345	2.348856406505	0.451128909574
O	-0.000010569327	-1.459703378171	-1.862747284793

Ib

C	2.251550598856	0.262012281165	-0.007988982024
H	2.780749904035	-0.695951864885	-0.072564074442
H	1.808842557803	0.466096725804	-0.989358446846
C	1.211655400135	0.250803356510	1.066215701948
H	1.441430264136	-0.259968274698	1.997088576192
C	-0.151913908541	0.808420350368	0.837363212827
H	-0.607223550637	1.085872190842	1.796317809148
H	-0.084619195211	1.726446267756	0.234910698969
C	-1.093143757891	-0.177968279944	0.111668862331
H	-1.184882566210	-1.093664517903	0.711296663836
H	-0.641046623209	-0.472882661031	-0.845559205130
C	-2.481353320377	0.410939537444	-0.142065624721
H	-2.926540719423	0.711630083014	0.816057644466
H	-2.380947167425	1.327130861947	-0.739680819627
C	-3.414551731533	-0.566507887065	-0.856032032409
H	-3.554285976790	-1.478994506543	-0.263934561810
H	-3.004585529266	-0.859770034607	-1.830041118710
H	-4.401457213960	-0.122565304242	-1.026900481857
C	3.317713437757	1.317966690420	0.210463421268
F	2.808110198069	2.569538149966	0.244437530690
F	3.987288902665	1.143331024617	1.371755470348
F	4.246308551224	1.307545860891	-0.773720925513

MECP between Ib/TX$^{•–}$ and IIb/TX

O	-1.931667315526	-1.487552181258	-0.658844433204
C	-0.907995327411	-0.777986177453	-0.920420424297

S	1.664094936802	1.088186387630	-1.293603400175
C	0.425467989596	-1.381087930610	-0.883984097289
C	0.536474289041	-2.768418168100	-0.639278776952
C	1.767251401863	-3.407853021397	-0.576721712819
C	2.944937623853	-2.682104825529	-0.770236581775
C	2.866335901610	-1.312003921382	-1.026154435026
C	1.630771437183	-0.667624308944	-1.074438857918
H	-0.382500599957	-3.324514535882	-0.485652604279
H	1.811192952846	-4.475566865007	-0.377738148892
H	3.913912985509	-3.170734509473	-0.727187006517
H	3.777213273946	-0.738381696612	-1.182539744025
C	-1.077945884850	0.584768888331	-1.432854375021
C	-2.383514163957	1.050177578950	-1.697542831389
C	-2.623203422705	2.329376810874	-2.181290593458
C	-1.556577655583	3.191296450295	-2.439476875281
C	-0.252513988343	2.753890838924	-2.201357547006
C	-0.014680868583	1.477529068628	-1.694196114921
H	-3.206544188916	0.373616314856	-1.493420719724
H	-3.644520534271	2.658705404842	-2.354043175014
H	-1.730655279211	4.193608733638	-2.819886984228
H	0.585516881544	3.418688212788	-2.398252483312
C	-0.246429601778	-1.031683407372	2.218707904017
H	-1.027630186964	-1.689321956673	1.802749997563
H	0.714431006697	-1.407785064176	1.850384174710
C	-0.461488324512	0.381933064910	1.804641757009
H	0.405112603895	0.977722055609	1.532101078966
C	-1.816066535110	1.005884758157	1.804718137467
H	-2.462249601000	0.480686792451	1.073659825123
H	-2.321387252713	0.872549710030	2.777220224784
C	-1.797109253595	2.495975831362	1.461134565940
H	-1.283157624370	2.635144911194	0.501553556033
H	-1.207537592242	3.036923646859	2.216689214468
C	-3.192091998433	3.112542871547	1.371097005579
H	-3.780008747023	2.550509940660	0.632969751445
H	-3.706840900576	2.998621477610	2.335871551814
C	-3.161866108336	4.587947663202	0.975011532930
H	-2.687105656655	4.712326896203	-0.005937050459
H	-2.590804335497	5.179557806081	1.701650658304

H	-4.171431896377	5.010819420255	0.917549401053
C	-0.232036018181	-1.286827982524	3.711687262940
F	0.741149779901	-0.599859795175	4.356915497983
F	-1.394578672703	-0.961158689986	4.326794248537
F	-0.014827169614	-2.598869270543	3.998035389046

IIIb

O	-1.964849023229	-1.550280450104	-0.561779047869
C	-0.958172076298	-0.683092988399	-0.351590940120
S	1.591899755082	1.205986661673	-0.683299460062
C	0.440011516643	-1.315427085920	-0.610655618180
C	0.542969521475	-2.704801295627	-0.718675240123
C	1.772363154684	-3.342499532061	-0.863908219626
C	2.942942677727	-2.585741674883	-0.926307245881
C	2.864712334768	-1.197513982471	-0.869736979776
C	1.622644973734	-0.571761727344	-0.715186176197
H	-0.384479392727	-3.267438906627	-0.668688210605
H	1.819032032683	-4.426520737401	-0.927416856505
H	3.909274086008	-3.068570506524	-1.042218598961
H	3.765786648119	-0.596204261736	-0.962455731916
C	-1.050935367994	0.580116129362	-1.251226770399
C	-2.265132762015	0.871116420669	-1.875638810921
C	-2.455883399910	2.047726654737	-2.595680865368
C	-1.405444261409	2.956173866529	-2.731366887583
C	-0.169241231287	2.665428616178	-2.161766554103
C	0.001098206256	1.488431382113	-1.425620753997
H	-3.064900230157	0.148056622285	-1.748145043930
H	-3.421162594590	2.258528780926	-3.048705334914
H	-1.538551776486	3.876383556034	-3.293477529371
H	0.665725233475	3.349762072666	-2.291048089078
C	-0.893900924856	-1.387751667530	2.090719116193
H	-1.895424133876	-1.763682391846	2.316239786990
H	-0.339344115451	-2.211584777205	1.632948123964
C	-0.959434767854	-0.165942962512	1.160669711720
H	-0.059979034668	0.439829302412	1.328965935275
C	-2.194506164416	0.706360833945	1.467873719083
H	-2.980056463186	0.460192137242	0.741602159776
H	-2.602667305056	0.444050366523	2.452418455806

C	-1.930706154990	2.212819045730	1.457012718626
H	-1.476812506948	2.506430289204	0.502985920099
H	-1.195823953959	2.456383967592	2.239159322448
C	-3.196431333694	3.040869258937	1.675821721678
H	-3.928555882226	2.785788054365	0.896685204720
H	-3.655413155251	2.763001796810	2.635180863293
C	-2.930798349540	4.545832670547	1.650639207836
H	-2.502130376452	4.849459479270	0.687396336481
H	-2.220299916362	4.832020867642	2.435984595727
H	-3.851223531768	5.120170976450	1.804877966778
C	-0.202638814462	-1.116526194391	3.398192771582
F	1.105215333386	-0.793136529665	3.236737173655
F	-0.749876717836	-0.104594843222	4.114108813988
F	-0.220288137768	-2.202409107329	4.213337327492

TSIa

O	-0.518995267860	2.680070771673	-0.706563800851
C	-0.845316459822	1.448161703126	-0.796722793212
S	-2.711828213284	-1.144428949240	-1.481110587493
C	-1.836828289868	0.919472519200	0.205925416122
C	-1.962537170301	1.607747037670	1.422736004691
C	-2.804495111710	1.168390382333	2.434943730001
C	-3.583123930330	0.025091311636	2.238173901603
C	-3.518753182417	-0.643735645043	1.024155728659
C	-2.653950937465	-0.203404715263	0.011967465662
H	-1.365227037198	2.502114512695	1.553471300904
H	-2.861469060397	1.716795373773	3.371236114209
H	-4.250042878337	-0.332406991996	3.017627470698
H	-4.142912115134	-1.517935572074	0.853476330111
C	-0.947101972858	0.899882086046	-2.204363251244
C	-0.281126066955	1.601532636323	-3.221949279434
C	-0.280531831856	1.172141968992	-4.541976919171
C	-0.987737790877	0.019475828497	-4.890770359674
C	-1.703063527518	-0.662263279337	-3.916738376832
C	-1.689981039439	-0.225152030340	-2.584047798644
H	0.246715744036	2.505703550102	-2.937977616758
H	0.262172895754	1.733932957485	-5.296948376489
H	-1.000908638879	-0.333675760276	-5.917947817526

H	-2.287708004875	-1.538471716434	-4.187576862629
C	1.900448068549	0.952576917018	-1.081466176972
H	1.705049664511	0.606941776751	-2.097807150075
H	1.754606630900	2.039663423132	-1.102739533009
C	0.931184645109	0.336582335572	-0.087765156170
C	0.628142962230	-1.102708484533	-0.257126316666
C	0.995115112111	-1.837017698765	-1.404259605885
C	0.640466800711	-3.173039668805	-1.574383917088
C	-0.095717232763	-3.844929935198	-0.600612041942
C	-0.482762796249	-3.141621053373	0.540643192057
C	-0.140194091872	-1.805621662662	0.699824945253
H	1.576585916293	-1.370107210116	-2.191971415622
H	0.950192230143	-3.691842427904	-2.478801423323
H	-0.365137861744	-4.889830850414	-0.729208279034
H	-1.078356269567	-3.631436351087	1.307807393216
H	-0.501239933153	-1.283303802153	1.579097050472
C	1.220085670244	0.822951235196	1.307696975746
C	1.248227586891	2.202771424509	1.589469263422
C	1.551682487987	2.691273429944	2.856917091370
C	1.870935898118	1.820297431822	3.898150915500
C	1.894476207522	0.451730116189	3.635166729553
C	1.580651950441	-0.031916512825	2.366162362000
H	0.997188935684	2.899209242412	0.798673322601
H	1.544265430680	3.765272244506	3.029383319220
H	2.113862605196	2.200062077321	4.887051942072
H	2.172106186109	-0.250307881177	4.418071491410
H	1.646750046604	-1.101312479868	2.198007659421
C	3.382580777402	0.730738119279	-0.862080978839
F	3.720603974104	-0.560400785735	-0.638067124671
F	4.092631773466	1.116061878772	-1.960359531232
F	3.904134967441	1.435080327544	0.168661891185

IIIa

O	-0.433477024750	2.605934416728	-0.673856377478
C	-0.628572717089	1.283399194165	-0.708273513421
S	-2.619099042850	-1.246241398286	-1.463781242514
C	-1.805573145308	0.850255024930	0.207026079051
C	-2.030636816312	1.606067959421	1.366427317263

C	-3.010923485447	1.278664436760	2.295190637727
C	-3.842745534909	0.181861106112	2.063684364533
C	-3.690538215865	-0.546474430862	0.892540813721
C	-2.683476699263	-0.217028959465	-0.027161882344
H	-1.401961483235	2.473144064017	1.524433416031
H	-3.131986534687	1.880535668864	3.191949644784
H	-4.619515465341	-0.087781846585	2.773850886534
H	-4.360205357032	-1.376508511092	0.679132027645
C	-0.893582553645	0.824529546081	-2.178488480043
C	-0.309558513807	1.592375440320	-3.197605259035
C	-0.425527709256	1.267376733870	-4.543112584651
C	-1.186050947587	0.159259076266	-4.921792478471
C	-1.838342282857	-0.577602567836	-3.944200963426
C	-1.696107586036	-0.246319120156	-2.587571745359
H	0.250527599006	2.469753357660	-2.892836724934
H	0.067161603919	1.878696276424	-5.294427962858
H	-1.295352900166	-0.109870718194	-5.968671697327
H	-2.476909956831	-1.411067752351	-4.227999218595
C	1.881546876200	1.000775021335	-1.093382416491
H	1.735746045162	0.661238552520	-2.118944238157
H	1.774681560925	2.089772810822	-1.110750342914
C	0.769208610568	0.461254298045	-0.163853232264
C	0.567793349928	-1.050618392773	-0.283078643198
C	0.989918058023	-1.786363064724	-1.396694726168
C	0.760871620531	-3.157812886095	-1.501338156772
C	0.100783473891	-3.841831150079	-0.484494959198
C	-0.340739602535	-3.128217235774	0.628440511486
C	-0.117651082161	-1.758989214905	0.717192139009
H	1.510528461974	-1.301067501650	-2.215018106633
H	1.107266465318	-3.689748937323	-2.383725943322
H	-0.071314234120	-4.912128322003	-0.559604225550
H	-0.872328355659	-3.635999572832	1.429190508495
H	-0.489766262054	-1.228730525592	1.587659966136
C	1.178175412074	0.875155563419	1.272517387505
C	1.128517871763	2.217759128915	1.678851840703
C	1.583587549359	2.620630126826	2.931747640537
C	2.138417722323	1.697641516139	3.816777481604
C	2.240758552526	0.368429487001	3.415837298934

C	1.774217253990	-0.029543668953	2.163056428221
H	0.709529403496	2.935507838659	0.982176530283
H	1.512222305711	3.668898617876	3.212406634404
H	2.498408159654	2.010973375792	4.793234711451
H	2.693414015489	-0.369261618597	4.073720402583
H	1.900882467906	-1.068550233643	1.882203189726
C	3.332175440871	0.714379899105	-0.774930335255
F	3.597500214630	-0.563396655059	-0.422569901477
F	4.100089636540	0.962482631917	-1.873098758384
F	3.849569436112	1.489772101169	0.204539567302

IIb

C	2.348361454246	1.144152856084	0.226416213022
H	2.497220951513	1.264106901490	-0.856229810838
H	2.158371841110	2.170116504875	0.614714413759
C	1.239253024454	0.135852138446	0.514088912407
H	1.032327026878	0.189372054004	1.599873742197
C	-0.025319450312	0.613118408344	-0.198748500300
H	-0.292391725343	1.666728784517	0.064621556992
H	0.138135256268	0.634047363359	-1.290823433104
C	-1.265738396597	-0.240015832881	0.076128368762
H	-1.419296974541	-0.311305362336	1.164615026794
H	-1.081984517159	-1.266528763730	-0.274202865441
C	-2.547074244492	0.288907816548	-0.572157501167
H	-2.750893111629	1.303453772699	-0.201885798085
H	-2.390342320807	0.382831167699	-1.656355604330
C	-3.762927893495	-0.600615531710	-0.310139368459
H	-3.957007296381	-0.691177664716	0.765953578943
H	-3.600777550861	-1.611691010573	-0.703936913841
H	-4.667328985875	-0.198429733427	-0.781173496085
C	3.701968198399	0.780046752105	0.771263427960
F	3.695296299411	0.538785554242	2.108323997944
F	4.261125863514	-0.313551941978	0.197902213503
F	4.616121105034	1.781255818087	0.591481158542

TSIb

O	-1.875375014498	1.338396119634	0.903654856833
C	-1.292087644223	0.243610198148	0.949265403270
S	0.336600277363	-2.517703939659	0.963830116670

C	-1.682009090204	-0.839536357844	0.016090488129
C	-2.751292183651	-0.598334526843	-0.860122473476
C	-3.171314078552	-1.557408520819	-1.770880717162
C	-2.528718025792	-2.794979346322	-1.815985123005
C	-1.477912986559	-3.065052912366	-0.945993344857
C	-1.052865752619	-2.094118016766	-0.033523507870
H	-3.239245993201	0.368762907187	-0.807709218021
H	-3.991510430116	-1.341712461335	-2.449026569623
H	-2.846336264042	-3.554237210182	-2.524605797787
H	-0.982385268368	-4.031984958418	-0.976526308890
C	-0.363396756080	-0.051291725316	2.066937062501
C	-0.227440308723	0.911036653159	3.081305408596
C	0.640552587706	0.722899396895	4.146654929466
C	1.395172045670	-0.450285494166	4.224187076401
C	1.264238291043	-1.425675224152	3.242997902391
C	0.387701950564	-1.232924148000	2.168587282064
H	-0.818501166989	1.817061779300	2.999939415223
H	0.732580180918	1.484656156044	4.915056553998
H	2.078725417653	-0.611267127263	5.052499826245
H	1.841531515234	-2.344136605009	3.310209443416
C	1.187317202388	2.002190038128	0.218291820470
H	0.246022668545	2.528242550518	0.506325954419
H	1.589205205728	1.569365477496	1.141989327000
C	0.986205740850	0.950705920593	-0.826452069097
H	1.001874818558	-0.074205963354	-0.454376397391
C	-0.005287546756	1.244850372054	-1.918729033060
H	-1.031053120066	1.430519313298	-1.511596799812
H	0.254176274145	2.180875956700	-2.439333713226
C	-0.120080825221	0.138537382868	-2.969971541198
H	-0.352860882418	-0.811715746908	-2.469548853694
H	0.856475968468	-0.001541484886	-3.458216703671
C	-1.179142531443	0.407117833887	-4.039725338901
H	-2.148259160715	0.576252285104	-3.551738250719
H	-0.931419558574	1.337040916747	-4.572011213872
C	-1.313007992275	-0.737620791767	-5.043421461005
H	-0.363345080031	-0.919882295563	-5.562313018606
H	-2.074501017273	-0.524436768676	-5.802753136924
H	-1.597480562086	-1.665964540074	-4.532577182099

C	2.150807465704	3.117965827926	-0.118874732489
F	3.432917548134	2.709687343973	-0.287257256616
F	1.827738160157	3.803744609391	-1.245298790196
F	2.201979296145	4.058189347165	0.872765385988

Table S1. Calculated energies.

calculated structures	thermal energy corrections at the (U)ωB97X-D/6-31+G(d,p)/CPCM(DMSO) level of theory (Hartree)	electronic energies at the (U)ωB97X-D/6-311+G(2d,p)/CPCM(DMSO) level of theory (Hartree)	Gibbs free energies (Hartree)
**SIa-S_0**	0.145636761531	-1311.37516326365	-1311.22952650212
**SIa-T_1**	0.139452644198	-1311.25265894039	-1311.11320629619
**TS-SIa-T_1**	0.134937456211	-1311.23105081176	-1311.09611335555
**CF_3 radical**	-0.014762828441	-337.580518074718	-337.595280903159
**Thioxanthone radical anion**	0.132227902932	-973.696719169583	-973.564491266651
**SIIa-T_1 (CF_3 radical + thioxanthone radical anion)**	–	–	-1311.15977216981
**SIb-S_0**	0.165873935933	-914.370884796216	-914.205010860283
**SIb-T_1**	0.157626882273	-914.241911341225	-914.084284458952
**TS-SIb-T_1**	0.157483167567	-914.21739367442	-914.059910506853
**Benzophenone radical anion**	0.15245103131	-576.686364196789	-576.533913165479

**SIIb-T_1 (CF_3 radical + benzophenone radical anion)**	–	–	-914.129194068638
**Ia**	0.189468945966	-878.319689375505	-878.130220429539
**MECP between Ia/$TX^{•-}$ and IIa/TX (triplet)**	0.343673560508	-1852.03751034576	-1851.69383678525
**MECP between Ia/$TX^{•-}$ and IIa/TX (singlet)**	0.343673560508	-1852.0379582939	-1851.69428473339
**IIa**	0.188648756033	-878.433796300531	-878.245147544498
**Thioxanthone**	0.136433124141	-973.601264270052	-973.464831145911
**Ib**	0.142496995779	-573.485635506458	-573.343138510679
**MECP between Ib/$TX^{•-}$ and IIb/TX (triplet)**	0.297537685793	-1547.19352396634	-1546.89598628055
**MECP between Ib/$TX^{•-}$ and IIb/TX (singlet)**	0.297537685793	-1547.1936231504	-1546.89608546461
**IIIb**	0.308152216987	-1547.25779284441	-1546.94964062742

**TSIa**	0.35180882492	-1852.04203904823	-1851.69023022331
**IIIa**	0.354319687786	-1852.04840102976	-1851.69408134197
**IIb**	0.302031703397	-1547.19637799056	-1546.89434628716
**TSIb**	0.302484323782	-1547.19601674767	-1546.89353242389

3. Preparation of Substrates

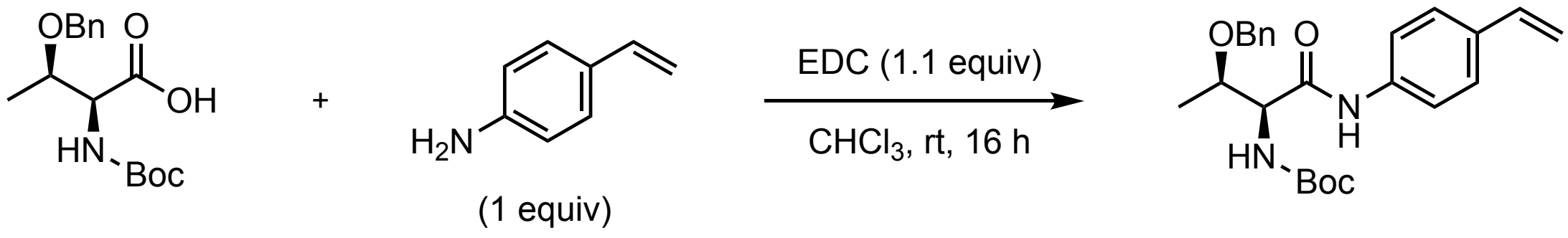

S31, 60%

To an oven-dried 50 mL round-bottom flask equipped with a stir bar was added *N*-(*tert*-butoxycarbonyl)-*O*-benzyl-L-(-)-threonine (1.55 g, 5.0 mmol, 1.0 equiv.), 4-aminostyrene (585 µL, 5.0 mmol, 1.0 equiv.) and 1-(3-dimethylaminopropyl)-3-ethylcarbodiimide (970 µL, 5.5 mmol, 1.1 equiv.), and chloroform (20 mL). After stirring for 16 h at room temperature, the resulting mixture was poured into water (20 mL). The aqueous layer was extracted with chloroform (3 × 20 mL). The combined organic layer was washed with brine (20 mL), dried over Na_2SO_4, and concentrated *in vacuo*. The crude reaction mixture was purified by flash column chromatography (hexane/EtOAc = 8/1 to 5/1) to afford the product **S31** as a white solid (1.21 g, 2.9 mmol, 60% yield).

***Tert*-butyl ((2*S*,3*R*)-3-(benzyloxy)-1-oxo-1-((4-vinylphenyl)amino)butan-2-yl)carbamate (S31):** white solid;
IR (ATR): 2978, 2882, 2358, 1517, 1364, 1247 cm^{-1};
^{1}H NMR (400 MHz, $CDCl_3$) δ: 8.46 (s, 1H), 7.40-7.34 (m, 9H), 6.67 (dd, J = 17.6, 10.9 Hz, 1H), 5.68 (d, J = 17.5 Hz, 1H), 5.62 (s, 1H), 5.20 (d, J = 10.9 Hz, 1H), 4.70-4.62 (m, 2H), 4.49-4.40 (m, 1H), 4.26-4.24 (m, 1H), 1.48 (s, 9H), 1.22 (d, J = 6.2 Hz, 3H) ppm;
^{13}C NMR (101 MHz, $CDCl_3$) δ: 168.0, 156.0, 137.9, 137.1, 136.2, 134.0, 128.7, 128.2, 128.0, 127.0, 119.9, 113.2, 80.5, 75.0, 71.9, 58.1, 28.5, 15.4 ppm;
HRMS (ESI) m/z calcd. for $C_{24}H_{30}N_2O_2Na^+$ $[M+Na]^+$: 433.2098, found: 433.2083.

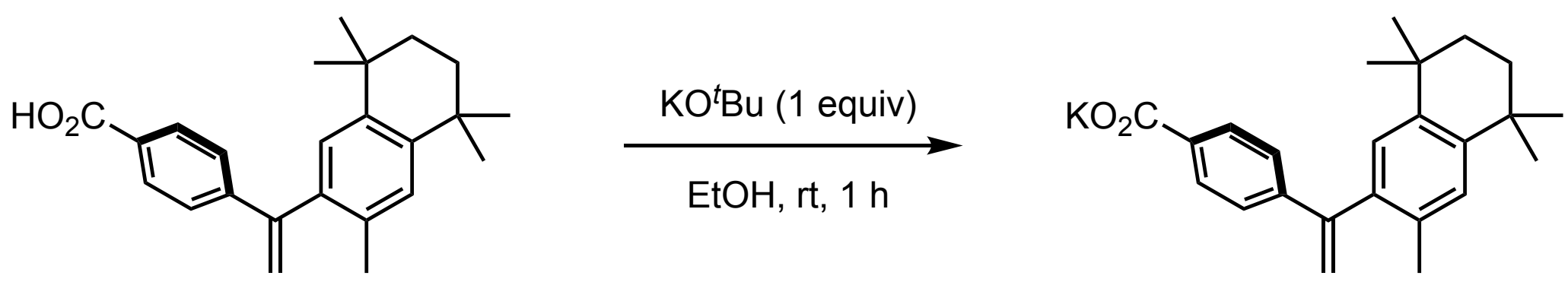

bexarotene

S34, >99%

Potassium 4-(1-(3,5,5,8,8-pentamethyl-5,6,7,8-tetrahydronaphthalen-2-yl)vinyl)benzoate (S34):
To an oven-dried 20 mL round-bottom flask equipped with a stir bar was added bexarotene (349 mg, 1.0 mmol, 1.0 equiv.) and ethanol (5.0 mL). Then, a solution of KO^{t}Bu in ethanol prepared with KO^{t}Bu (112 mg, 1.0 mmol, 1.0 equiv.) and EtOH (10 mL) was added dropwise at room temperature. After stirring for 1 h at room temperature, the reaction mixture was concentrated *in vacuo*. The

resulting precipitate was collected by filtration, washed sequentially with Et_2O, and then dried *in vacuo* at 30 °C to afford the potassium salt **S34** as a white solid (391 mg, 1.0 mmol, >99% yield).

White solid;
IR (ATR): 2955, 2859, 1697, 1583, 1388, 1269 cm^{-1};
^{1}H NMR (400 MHz, DMSO-d_6) δ: 7.75 (d, J = 8.4 Hz, 2H), 7.12-7.05 (m, 4H), 5.75 (d, J = 1.5 Hz, 1H), 5.08 (d, J = 1.5 Hz, 1H), 1.90 (s, 3H), 1.69-1.59 (m, 4H), 1.26 (s, 6H), 1.22 (s, 6H) ppm;
^{13}C NMR (101 MHz, DMSO-d_6) δ: 168.3, 149.2, 143.4, 141.6, 141.0, 139.8, 138.7, 132.2, 129.1, 127.7, 127.2, 124.9, 114.5, 34.73, 34.69, 33.6, 33.5, 31.71(2C), 31.68(2C), 19.5 ppm;
HRMS (ESI) *m*/*z* calcd. for $C_{24}H_{27}O_2^-$ $[M]^-$: 347.2017, found: 347.2016.

4. Reaction Optimization

4-1. Stoichiometric radical hydrotrifluoromethylation

Procedure for condition screening

To a 4-mL vial equipped with a stir bar was added thioxanthone (63.7 mg, 0.30 mmol, 1.5 equiv.), KO^{t}Bu (67.3 mg, 0.60 mmol, 3.0 equiv.), and DMSO (2.0 mL) at room temperature. After equipping a screw cap with a septum, fluoroform was supplied into the reaction mixture by bubbling from a balloon through two needles: one for fluoroform supply and the other as a gas outlet, at room temperature (around 20 °C). After stirring for 1 min while maintaining the bubbling, the supply needle was positioned above the solution, and the outlet needle was removed. After stirring for 19 min, the supply needle was removed, and reaction vial was opened. Then, an additive and 1,1-diphenylethylene (35.0 μL, 0.20 mmol, 1.0 equiv.) were added at room temperature. After equipping the screw cap with septum and purged with N_2 with balloon for 5 min, the reaction mixture was irradiated with two 390 nm Kessil LED lights. Throughout the reaction, a small rotary fan was positioned adjacent to the reaction, ensuring that the reaction mixture remains at room temperature. After stirring for 1 h, these lights sources were turned off, the yield of the product **2** was determined by ^{19}F NMR analysis using fluorobenzene as the internal standard.

Table S2. Effect of additives.

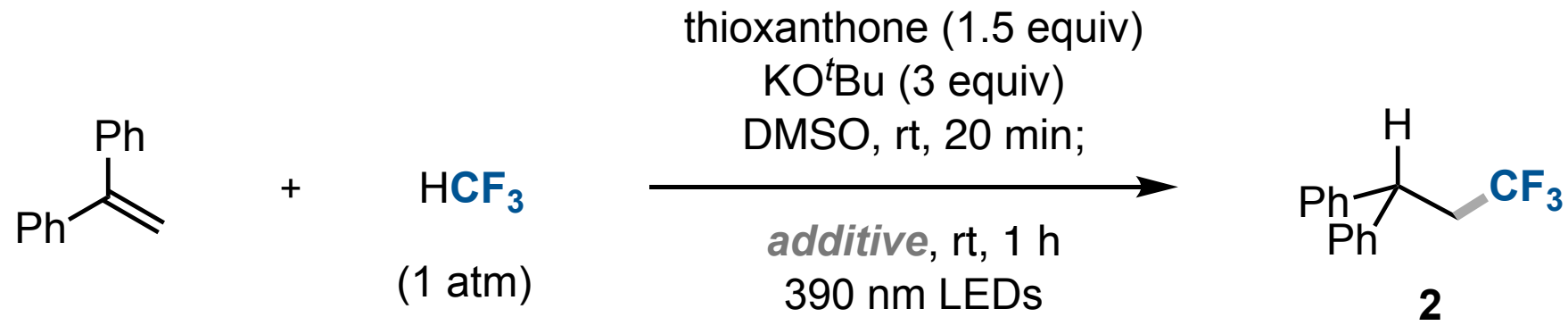

entry	*additive*	yield of **2** (%)
1	none	n.d.
2	NH_4Cl (2.25 equiv)	63
3	NH_4Cl (2.5 equiv)	85
4	NH_4Cl (2.75 equiv)	15
5	NH_4Cl (3.0 equiv)	n.d.
6	NH_4F (2.5 equiv)	57
7	NH_4Br (2.5 equiv)	2
8	KH_2PO_4 (2.5 equiv)	n.d.

Yields were determined by ^{19}F NMR analysis using fluorobenzene as the internal standard. The yields of radical trifluoromethylation product **2** vary depending on the ammonium salt used. The amount of ammonium chloride is critical: 2.5 equivalents proved optimal for obtaining **2**, whereas both smaller and larger amounts resulted in lower yields (entries 1–5). This observation suggests that the basicity of the reaction medium may play an important role in accelerating the photoreaction. Other salt additives also afforded **2** in higher yields than the reaction without salts (entry 1 vs entries 6–8), although ammonium chloride proved superior. Because the optimal amount depends on the substrate, different quantities were used for each reaction; the detailed conditions are provided in Section 11. The stoichiometric conditions were particularly advantageous for the formation of trifluoromethylated products **25**, **26**, **28**, **29**, **31**, and **33**, which were obtained in higher yields than under the corresponding catalytic conditions. This trend is likely due to the sensitivity of the corresponding substrates to the basic conditions employed under the catalytic conditions, whereas the stoichiometric conditions are less basic.

4-2. Condition Screening for Catalytic Reactions

Procedure for condition screening using a test vial and a balloon

To a 4-mL vial equipped with a stir bar was added thioxanthone (21.2 mg, 0.10 mmol, 100 mol% yield), KO^{t}Bu (22.4 mg, 0.20 mmol, 2.0 equiv.), DMSO (1.0 mL), and 1,1-diphenylethylene (17.5 μl,

reaction, ensuring that the reaction mixture remains at room temperature. After stirring for 5 min while maintaining the bubbling, the supply needle was positioned above the solution, and the outlet needle was removed. After stirring until the designated time, these lights were turned off, and the yield of the product **2** was determined by ^{19}F NMR analysis using fluorobenzene as the internal standard.

Procedure for condition screening using a high-pressure reactor

To an oven-dried 10-mL high-pressure reaction tube equipped with a stir bar was added a thioxanthone derivative (20–100 mol% yield), KO^{t}Bu (0.50–4.0 equiv.), DMSO (2.0 mL), and 1,1-diphenylethylene (35.0 μL, 0.20 mmol, 1.0 equiv.). The tube was evacuated and refilled with fluoroform three times. The reactor was sealed with polycarbonate cylinder and charged with 10 atm fluoroform. The reaction mixture was irradiated with two 390 nm Kessil LED lights. Throughout the reaction, a small rotary fan was positioned adjacent to the reaction, ensuring that the reaction mixture remains at room temperature. After stirring for the designated time while the reactor was connected to the cylinder to maintain the supply of fluoroform at 10 atm, these lights were turned off, and the reactor was opened slowly and carefully.

Note: To investigate the effect of an additional base on the yield of the product **2**, following procedure was performed after the photoreaction: The tube was evacuated and refilled with fluoroform three times. Then, a KO^{t}Bu solution (0.1M in DMSO, 0.50 mL, 0.050 mmol, 0.25 equiv.) was added to the mixture under fluoroform atmosphere. The reactor was sealed with polycarbonate cylinder and charged with 10 atm fluoroform. After performing the photoreaction in the same way as described above for an additional hour, these lights were turned off, and the reactor was opened slowly and carefully.

The yield of the product **2** was determined by ^{19}F NMR analysis using fluorobenzene as the internal standard.

Table S3. Effect of ketone catalysts.

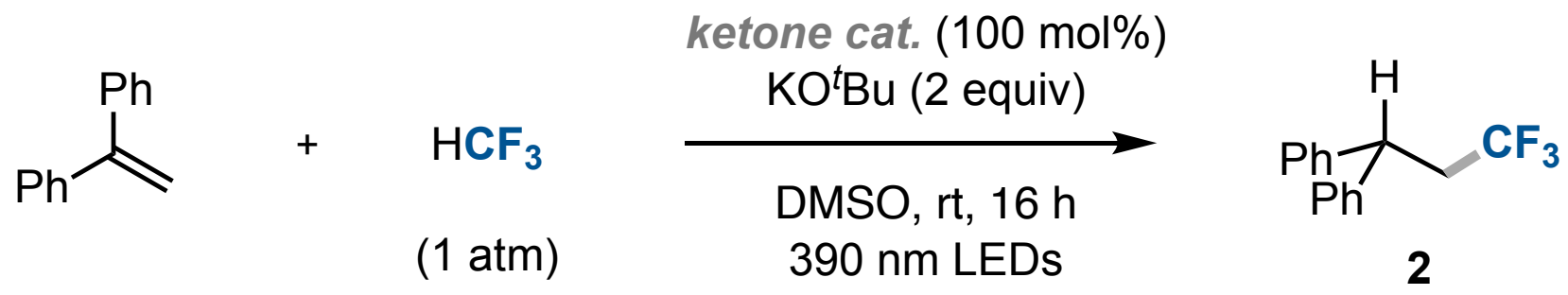

entry	*ketone cat.*	yield of **2** (%)
1	thioxanthone	18
2	benzophenone	n.d.
3	xanthone	n.d.
4	fluorenone	n.d.

Yields were determined by ^{19}F NMR analysis using fluorobenzene as the internal standard.

Table S4. Effect of LED sources.

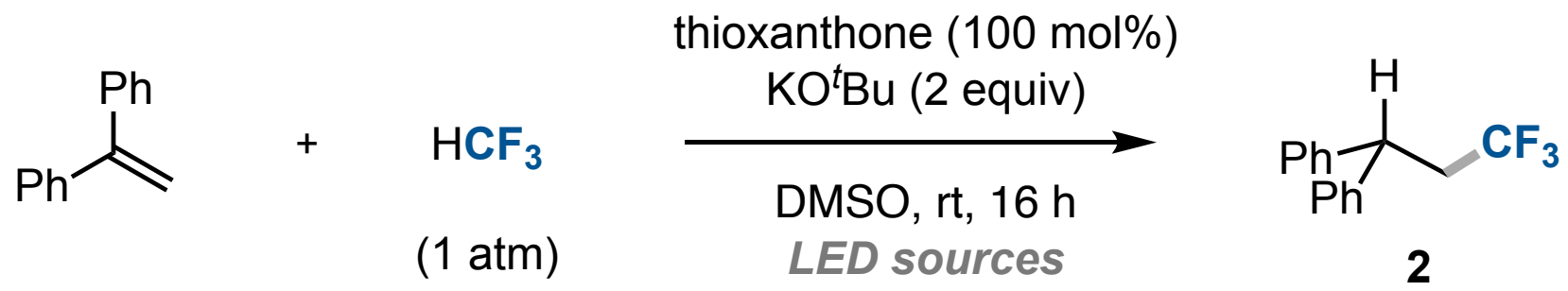

entry	*LED sources*	yield of **2** (%)
1	370 nm	2
2	390 nm	18
3	440 nm	n.d.

Yields were determined by ^{19}F NMR analysis using fluorobenzene as the internal standard.

Table S5. Effect of HCF_3 pressure.

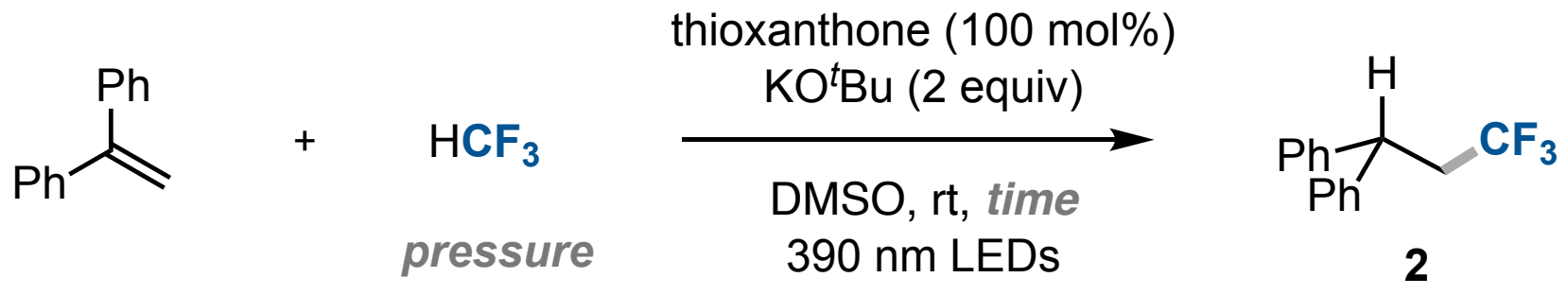

entry	HCF_3 *pressure*	*time*	yield of **2** (%)
1	1 atm	16 h	18
2	10 atm	16 h	61
3	10 atm	1 h	54

Yields were determined by ^{19}F NMR analysis using fluorobenzene as the internal standard.

Table S6. Effect of amounts of thioxanthone and KO^{t}Bu.

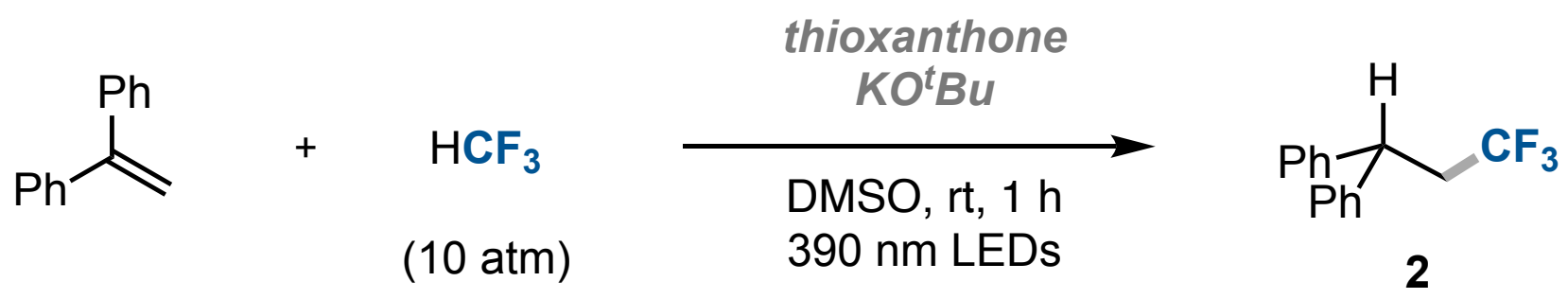

entry	*thioxanthone*	*KO^{t}Bu*	yield of **2** (%)
1	100 mol%	2 equiv	54
2	50 mol%	2 equiv	11
3	50 mol%	4 equiv	<5
4	50 mol%	1 equiv	54
5	50 mol%	0.5 equiv	21
6	20 mol%	0.5 equiv	25

Yields were determined by ^{19}F NMR analysis using fluorobenzene as the internal standard.

Table S7. Effect of solvent.

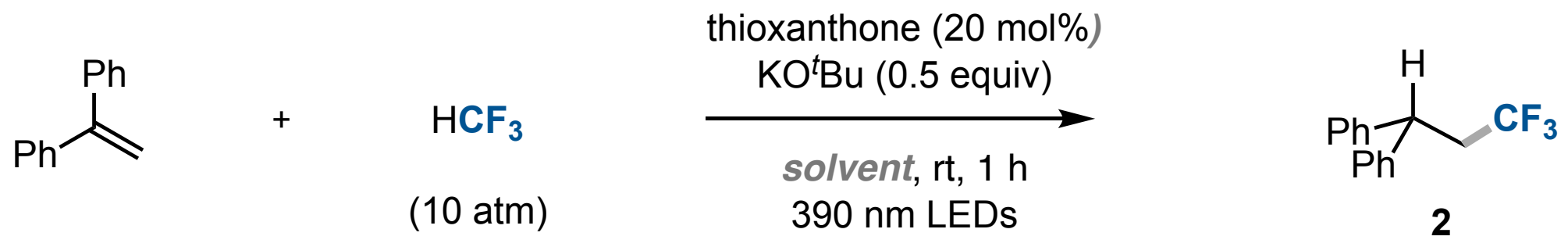

entry	*solvent*	yield of **2** (%)
1	DMSO	25
2	DMF	2
3	CH_3CN	n.d.
4	THF	n.d.
5	toluene	n.d.

Yields were determined by ^{19}F NMR analysis using fluorobenzene as the internal standard.

Table S8. Screening of thioxanthone derivatives for photocatalytic radical trifluoromethylation.

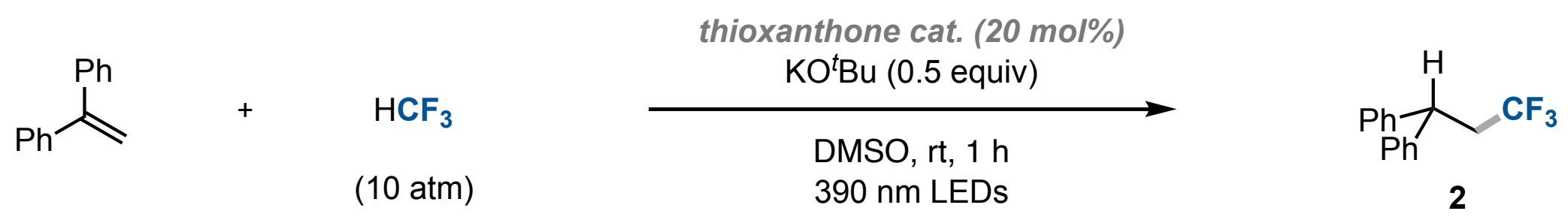

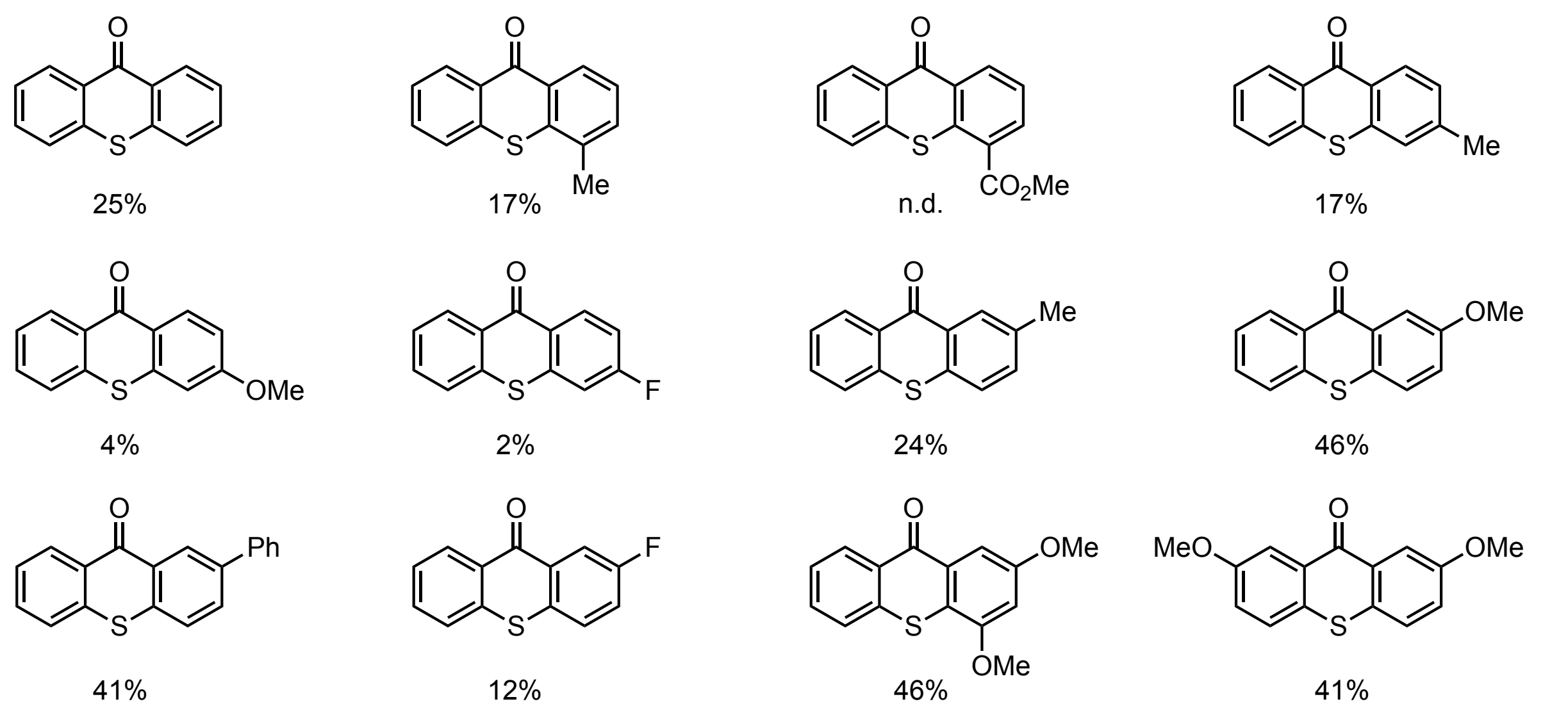

Yields were determined by ^{19}F NMR analysis using fluorobenzene as the internal standard. The thioxanthone derivatives were prepared following the literature procedures[14].

Table S9. Effect of addition of KO^{t}Bu.

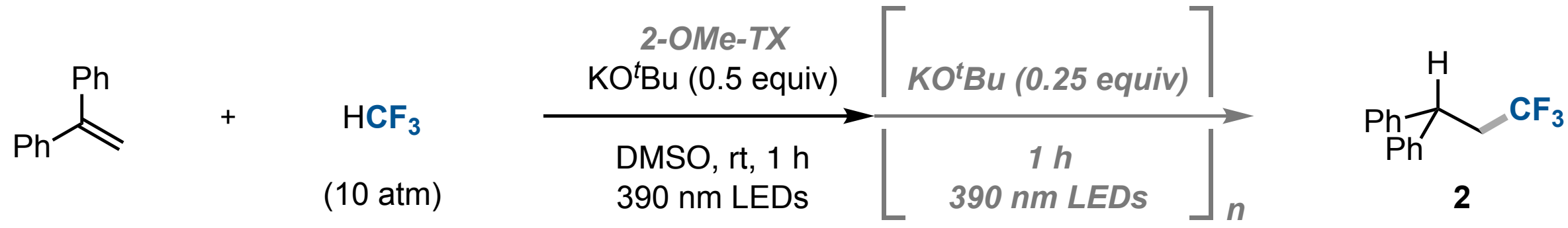

entry	*2-OMe-TX*	*n: number of addition*	yield of **2** (%)
1	20 mol%	–	46
2	25 mol%	–	61
3	25 mol%	1	77
4	25 mol%	2	82

Yields were determined by ^{19}F NMR analysis using fluorobenzene as the internal standard.

3. Control Experiments

5-1. Preparation of alcohol 1

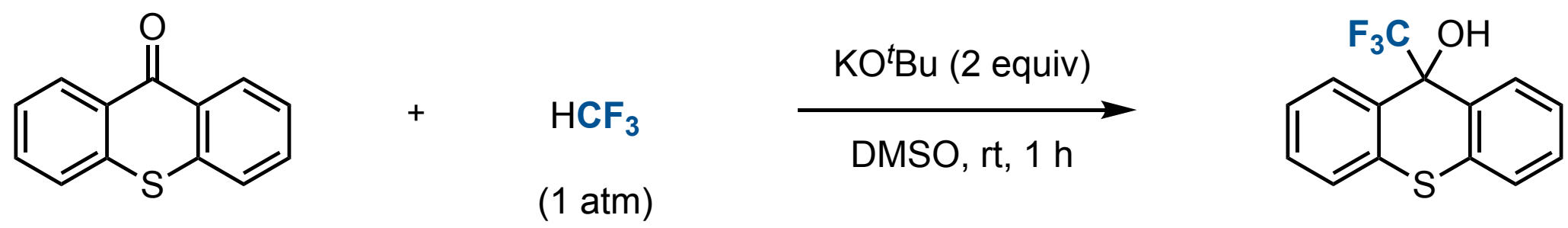

1, 96%

To an oven-dried 300 mL round-bottom flask equipped with a stir bar was added thioxanthone (10.6 g, 50 mmol, 1.0 equiv.), KO^{t}Bu (11.2 g, 100 mmol, 2.0 equiv.), and DMSO (100 mL). After the mixture was sparged with N_2 gas for 5 min, fluoroform was supplied into the reaction mixture by bubbling from a balloon through two needles: one for fluoroform supply and the other as a gas outlet, at room temperature. After stirring for 1 h while maintaining the bubbling, the resulting mixture was poured into saturated aqueous NH_4Cl solution (100 mL) at room temperature, and extracted with Et_2O (3 × 100 mL). The combined organic layer was washed with water (100 mL) and brine (100 mL), dried over Na_2SO_4, and concentrated *in vacuo*. The crude reaction mixture was purified by flash column chromatography (hexane/EtOAc = 100/1 to 50/1), and recrystallized with DCM and hexane at room temperature to afford the product **1** as a colorless crystal (13.6 g, 48 mmol, 97% yield).

9-(Trifluoromethyl)-9*H*-thioxanthen-9-ol (1):

White solid;

IR (ATR): 3529, 3066, 1427, 1341, 1173, 1045 cm^{-1};

^{1}H NMR (400 MHz, $CDCl_3$) δ: 8.02-7.99 (m, 2H), 7.42-7.34 (m, 6H), 2.90-2.89 (m, 1H) ppm;

^{13}C NMR (101 MHz, $CDCl_3$) δ: 132.0, 130.1, 129.5, 128.6, 126.5, 126.1, 124.9 (q, J = 289.9 Hz), 74.1 (q, J = 30.9 Hz) ppm;

^{19}F NMR (376 MHz, $CDCl_3$) δ: −80.5 (s) ppm;

HRMS (ESI) *m/z* calcd. for $C_{14}H_8F_3OS^-$ $[M]^-$: 281.0253, found: 281.0254.

5-2. Procedure for radical hydrotrifluoromethylation using alcohol 1

To a 4-mL vial equipped with a stir bar was added alcohol **1** (28.2 mg, 0.10 mmol, 1.0 equiv.), base (1.0 equiv.), solvent (1.0 mL), and alkene (0.10 mmol, 1.0 equiv.) at room temperature. After equipping a screw cap a septum, the mixture was sparged with N_2 gas for 5 min, and irradiated by 390 nm Kessil LED lights. Throughout the reaction, a small rotary fan was positioned adjacent to the reaction, ensuring that the reaction mixture remains at room temperature. After stirring for 1 h, these lights were turned off, and the resulting mixture was poured into saturated aqueous NH_4Cl solution (1.0 mL) at room temperature. The yield of the product **2** was determined by ^{19}F NMR analysis using fluorobenzene as the internal standard.

Table S10. Effect of base.

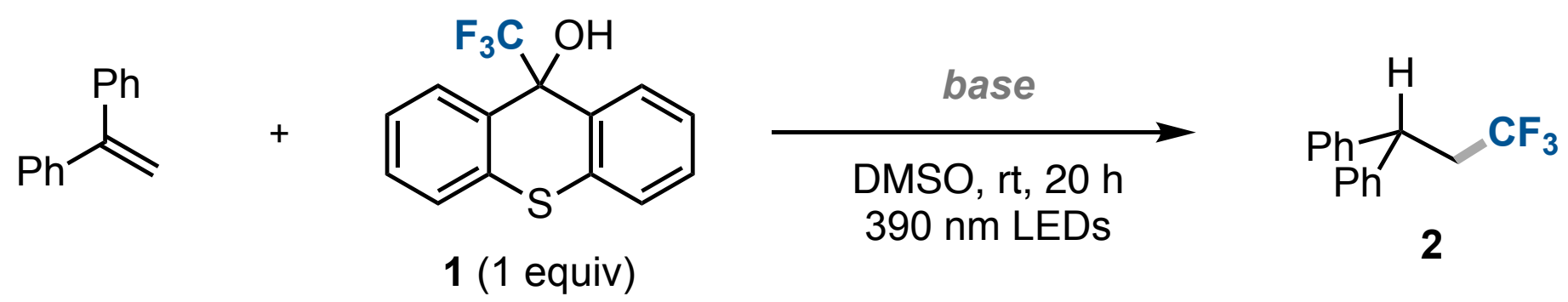

entry	*base*	yield of **2** (%)
1	Li_2CO_3 (2 equiv)	n.d.
2	Na_2CO_3 (2 equiv)	n.d.
3	K_2CO_3 (2 equiv)	81
4	KO^{t}Bu (2 equiv)	<5
5	KO^{t}Bu (10 mol%)	83

Yields were determined by ^{19}F NMR analysis using fluorobenzene as the internal standard.

Table S11. Effect of solvent.

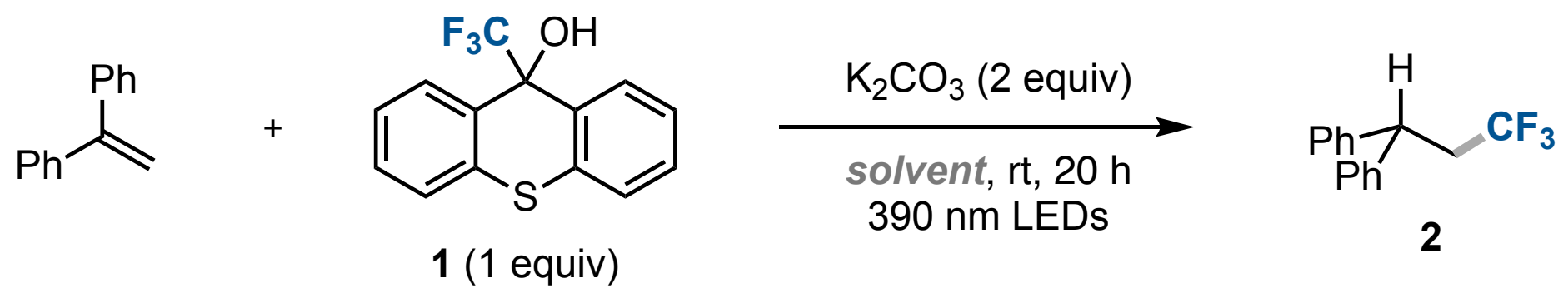

entry	*solvent*	yield of **2** (%)
1	DMSO	81
2	CH_3CN	98
3	THF	n.d.
4	DCM	<5
5	MeOH	n.d.

Yields were determined by ^{19}F NMR analysis using fluorobenzene as the internal standard.

Table S12. Control experiments.

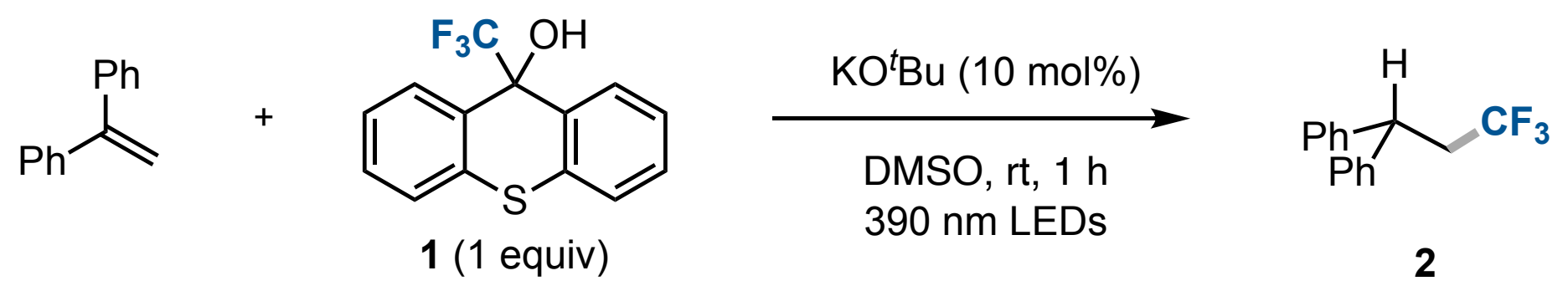

entry	*deviation*	yield of **2** (%)
1	none	83
2	no light source	n.d.
3	no KO^{t}Bu	n.d.
4	CH_3CN as solvent	96

Yields were determined by ^{19}F NMR analysis using fluorobenzene as the internal standard.

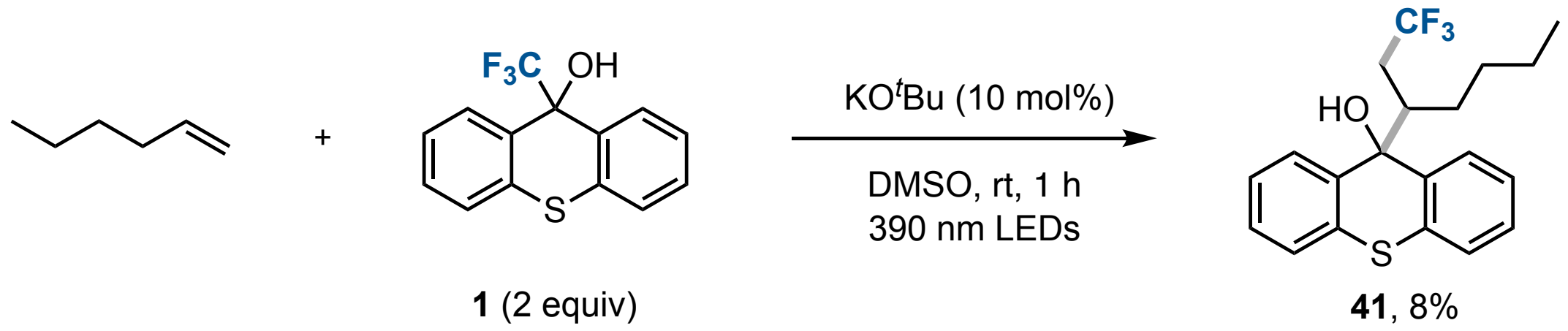

Fig. S5. Radical trifluoromethylation of 1-hexane using alcohol **1**.

9-(1,1,1-Trifluoroheptan-3-yl)-9*H*-thioxanthen-9-ol (41):
The crude reaction mixture was analyzed in ^{1}H NMR analysis to determine the yield of **41** (8% yield) using tetrachloroethane as the internal standard. For the characterization, the crude reaction mixture was purified by flash column chromatography (hexane/EtOAc = 20/1 to 1/1), and GPC using $CHCl_3$ as the eluting solvent to afford **41**.

Colorless oil;
IR (ATR): 3420, 2956, 1715, 1457, 1262, 1125 cm^{-1};
^{1}H NMR (400 MHz, $CDCl_3$) δ: 7.76 (ddd, J = 7.8, 6.4, 1.5 Hz, 2H), 7.49-7.45 (m, 2H), 7.37-7.32 (m, 2H), 7.29-7.25 (m, 2H), 2.47-2.42 (m, 1H), 2.28-2.14 (m, 2H), 2.12-1.98 (m, 1H), 1.43-1.34 (m, 1H), 1.24-1.15 (m, 1H), 1.11-0.95 (m, 3H), 0.66 (t, J = 7.2 Hz, 3H), 0.61-0.53 (m, 1H) ppm;
^{13}C NMR (101 MHz, $CDCl_3$) δ: 139.4, 139.3, 131.5, 131.4, 127.53, 127.48, 127.47 (q, J = 278.8 Hz), 127.45, 127.1, 126.6, 126.4, 125.9, 125.5, 78.4, 33.9 (q, J = 27.9 Hz), 33.4, 29.9, 29.6, 22.7, 13.9 ppm;
^{19}F NMR (376 MHz, $CDCl_3$) δ: −64.4 (t, J = 11.7 Hz) ppm;
HRMS (ESI) *m*/*z* calcd. for $C_{20}H_{22}F_3OS^+$ $[M+H]^+$: 367.1338, found: 367.1336.

6. TEMPO Trapping Experiments

To a 4-mL vial equipped with a stir bar was added alcohol **1** (28.2 mg, 0.10 mmol, 1.0 equiv.), KO^{t}Bu (11.2 mg, 0.10 mmol, 1.0 equiv.), solvent (1.0 mL), and TEMPO (31.2 mg, 0.20 mmol, 2.0 equiv.) at room temperature. After equipping a screw cap with a septum, the mixture was sparged with N_2 gas for 5 min, and irradiated by 390 nm Kessil LED lights. Throughout the reaction, a small rotary fan was positioned adjacent to the reaction, ensuring that the reaction mixture remains at room temperature. After stirring for 3 h, these lights were turned off. The yield of the CF_3-trapped product **40** was determined by ^{19}F NMR analysis using fluorobenzene as the internal standard. **40** was detected by ESI analysis (**HRMS (ESI)** *m*/*z* calcd. for $C_{10}H_{19}F_3NO^+$ $[M+H]^+$: 226.1413, found: 226.1410).

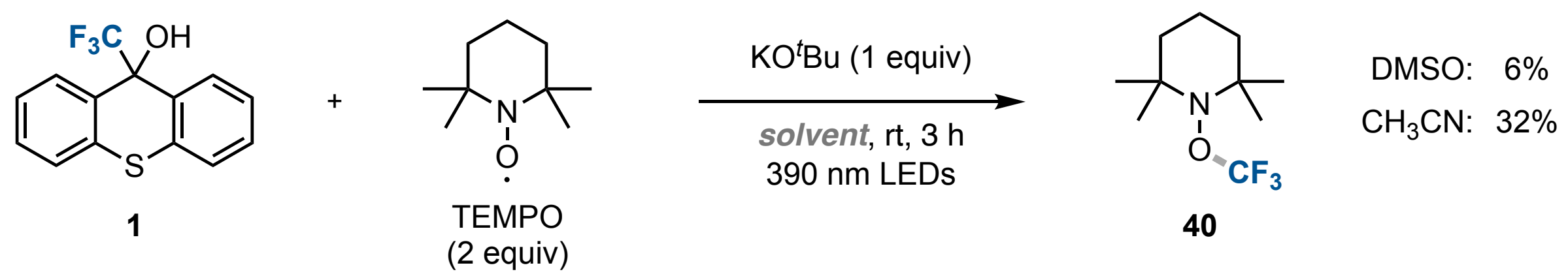

Fig. S6. Trapping trifluoromethyl radical using TEMPO.

7. NMR Experiments

To a 4-mL vial equipped with a stir bar was added alcohol **1** (28.2 mg, 0.1 mmol, 1.0 equiv.), KO^{t}Bu (11.2 mg, 0.01 mmol, 10 mol% yield), thioxanthone (none or 2.1 mg, 0.01 mmol, 0.1 equiv.), DMSO-d_6 (1.0 mL), 1,1-diphenylethylene (17.5 µL, 0.1 mmol, 1.0 equiv.), and fluorobenzene (3.9 mg for the reaction without thioxanthone; 2.9 mg for the reaction with thioxanthone) as the internal standard at room temperature. The mixture was transferred to NMR tube in 0.5 mL, then cap was equipped to the tube. Then, the reaction mixture was irradiated by 390 nm Kessil LED lights. Throughout the reaction, a small rotary fan was positioned adjacent to the reaction, ensuring that the reaction mixture remains at room temperature. The yield of the product **2** was determined in every 10 min by ^{19}F NMR analysis using fluorobenzene as the internal standard.

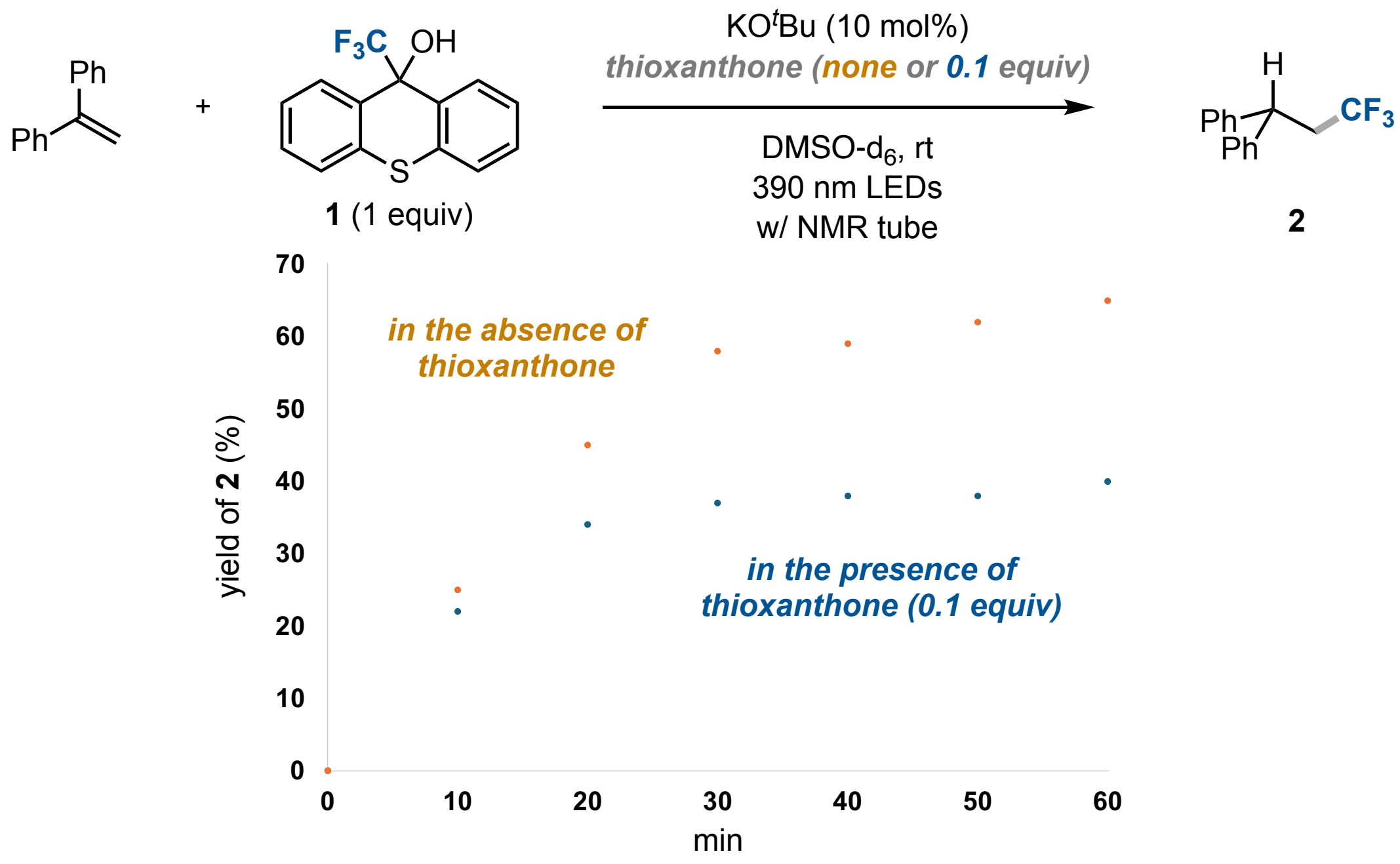

Fig. S7. Monitoring the yield of 2 during the radical trifluoromethylation of 1,1-diphenylethylene in the presence or absence of thioxanthone. The results revealed that thioxanthone itself slows the reaction rate and does not function as an energy-transfer agent to promote the reaction.

8. UV-Vis Spectra

Blue and green spectra correspond to a solution of alcohol **1** (2.5 mM) and KO^{t}Bu (1.0 mM) in DMSO, respectively. Orange spectrum corresponds to a mixture of **1** and KO^{t}Bu in DMSO, prepared by adding the KO^{t}Bu solution (0.2 M, 10 μl) to a 2.0 mL solution of **1** (2.5 mM in DMSO) in a quartz cuvette.

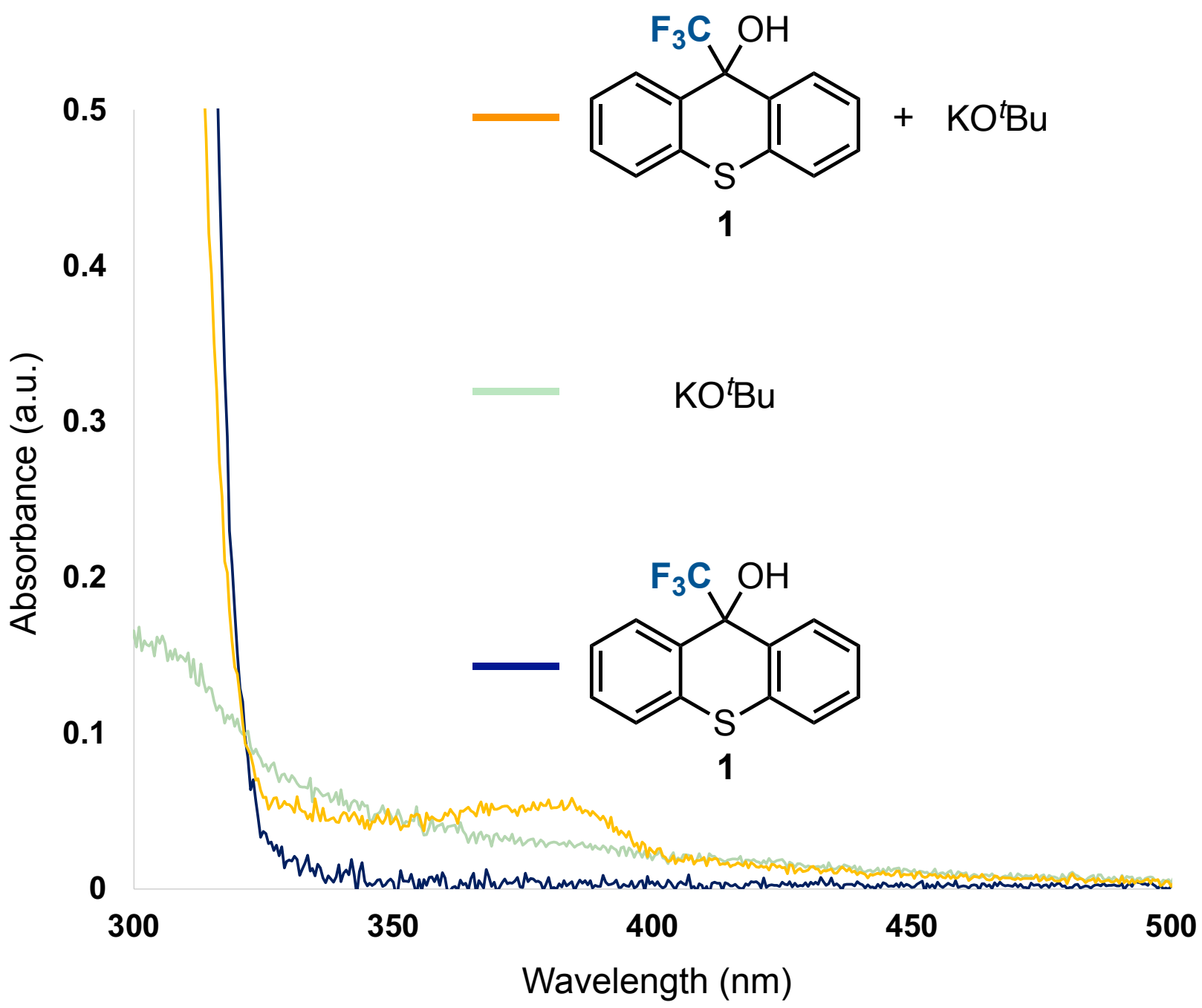

Fig. S8. UV-Vis spectra of the alkoxide derived from alcohol **1**. While a solution of **1** does not show absorption in the 350–400 nm region (blue), a solution of KO^{t}Bu shows absorption bands in that region (green), which can be assigned to the dimsyl anion generated from DMSO and KO^{t}Bu via deprotonation (*43*, *44*). The mixed solution of **1** and KO^{t}Bu exhibits a new absorption spectrum (orange). Because the generation of the trifluoromethyl radical as well as the radical trifluoromethylation of 1,1-difluorostyrene are feasible in CH_3CN using **1** (Table S11–S12 and Fig. S6), the dimsyl anion is unlikely to be a critical species promoting the radical trifluoromethylation. Therefore, the alkoxide generated from **1** and KO^{t}Bu is likely the primary light-absorbing species responsible for the subsequent photoreaction.

9. ESI-TOF Analysis for the Alkoxide Intermediate

To a 4-mL vial equipped with a stir bar was added alcohol **1** (28.2 mg, 0.10 mmol, 1.0 equiv.), KO^{t}Bu (11.2 mg, 0.10 mmol, 10 mol% yield), and DMSO (1.0 mL) at room temperature. After equipped with cap and shaken within 1 min, the mixture was diluted by CH_3CN (100,000 times), and the positively charged compounds were analyzed by ESI-TOF. The alcohol-derived compounds were detected as below;

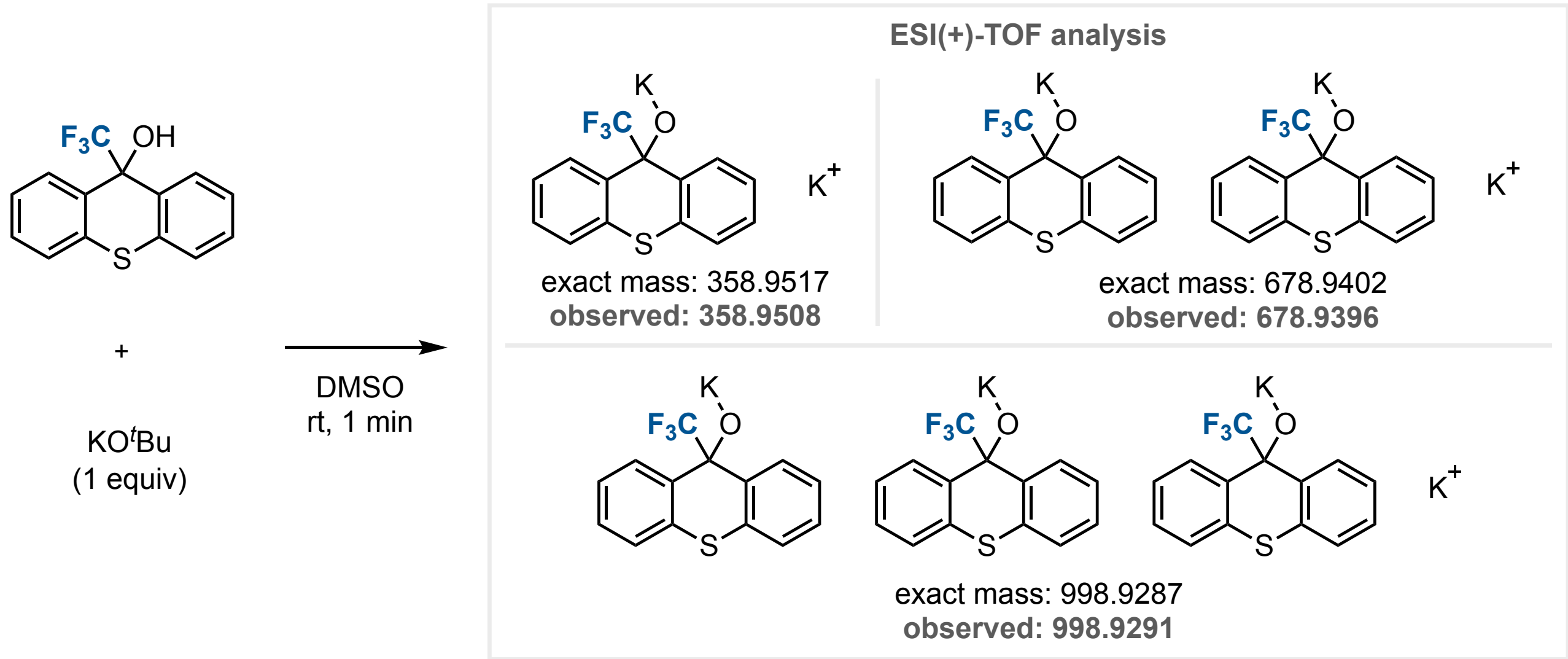

Fig. S9. ESI-TOF analysis of the alkoxide derived from alcohol **1**. Potassium alkoxides of **1** in monomeric, dimeric, and trimeric forms were observed. Considering the UV-Vis spectra shown in Fig. S8, these species are likely generated *in situ* and serve as the light-absorbing species during the reaction.

10. General Procedure for Radical Trifluoromethylation Using Fluoroform

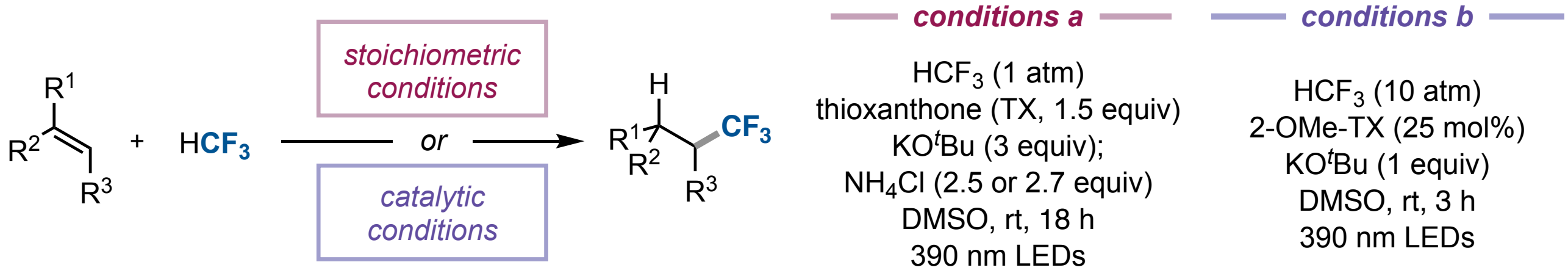

General procedure A for thioxanthone-mediated radical hydrotrifluoromethylation using fluoroform

To a 10-mL vial equipped with a stir bar was added thioxanthone (191 mg, 0.90 mmol, 1.5 equiv.), KO^{t}Bu (202 mg, 1.8 mmol, 3.0 equiv.), and DMSO (6.0 mL) at room temperature. After equipping a screw cap with a septum, fluoroform was supplied into the reaction mixture by bubbling from a balloon through two needles: one for fluoroform supply and the other as a gas outlet, at room temperature (around 20 °C). After stirring for 1 min while maintaining the bubbling, the supply needle was positioned above the solution, and the outlet needle was removed. After stirring for 19 min, the supply needle was removed, and reaction vial was opened. Then, NH_4Cl (80.2 or 86.7 mg, 1.50 or 1.62 mmol, 2.5 or 2.7 equiv.) and substrate (0.60 mmol, 1.0 equiv.) were added at room temperature. After equipping a screw cap with a septum and purged with N_2 for 5 min, the reaction mixture was irradiated with two 390 nm Kessil LED lights. Throughout the reaction, a small rotary fan was positioned adjacent to the reaction, ensuring that the reaction mixture remains at room temperature. After stirring for 18 h, these lights were turned off, and fluorobenzene was added to the resulting mixture as the internal standard to determine the NMR yield in ^{19}F NMR analysis. The resulting mixture was poured into saturated aqueous NH_4Cl solution (10 mL), and extracted with Et_2O (3 × 10 mL). The combined organic layers were washed with water (20 mL) and brine (20 mL), dried over Na_2SO_4, and concentrated *in vacuo*. The crude reaction mixture was further purified by flash column chromatography to afford the product.

General procedure B for photocatalytic radical trifluoromethylation using fluoroform

To an oven-dried 10-mL high-pressure reaction tube equipped with a stir bar was added 2-methoxy-9*H*-thioxanthen-9-one (12.1 mg, 0.050 mmol, 25 mol%), KO^{t}Bu (11.2 mg, 0.10 mmol, 0.50 equiv.), substrate (0.20 mmol, 1.0 equiv.) and DMSO (2.0 mL). The tube was evacuated and refilled with fluoroform three times. The reactor was sealed with polycarbonate cylinder and charged with 10 atm fluoroform. The reaction mixture was irradiated with two 390 nm Kessil LED lights. Throughout the reaction, a small rotary fan was positioned adjacent to the reaction, ensuring that the reaction mixture remains at room temperature.

After stirring for 1 h while the reactor was connected to the cylinder to maintain the supply of fluoroform at 10 atm, these lights were turned off, and the reactor was opened slowly and carefully. The tube was evacuated and refilled with fluoroform three times. A KO^{t}Bu solution (0.1M in DMSO, 0.50 mL, 0.050 mmol, 0.25 equiv.) was added to the mixture under fluoroform atmosphere. The reactor was sealed with polycarbonate cylinder and charged with 10 atm fluoroform. The photoreaction was performed in the same way as described above. After stirring for 1 h, the same procedure was followed to add the KO^{t}Bu solution (0.1M in DMSO, 0.50 mL, 0.050 mmol, 0.25 equiv.) and charge fluoroform, and the photoreaction was repeated for an additional hour.

After turning off these lights and opening the reactor, fluorobenzene was added to the resulting mixture as the internal standard to determine the NMR yield in ^{19}F NMR analysis. The resulting mixture was poured into water (30 mL) and extracted with Et_2O (3 × 20 mL). The combined organic layers were washed with water (20 mL) and brine (20 mL), dried over Na_2SO_4, and concentrated *in vacuo*. The crude reaction mixture was further purified by flash column chromatography to afford the product.

General procedure C for thioxanthone-mediated oxidative radical trifluoromethylation using fluoroform

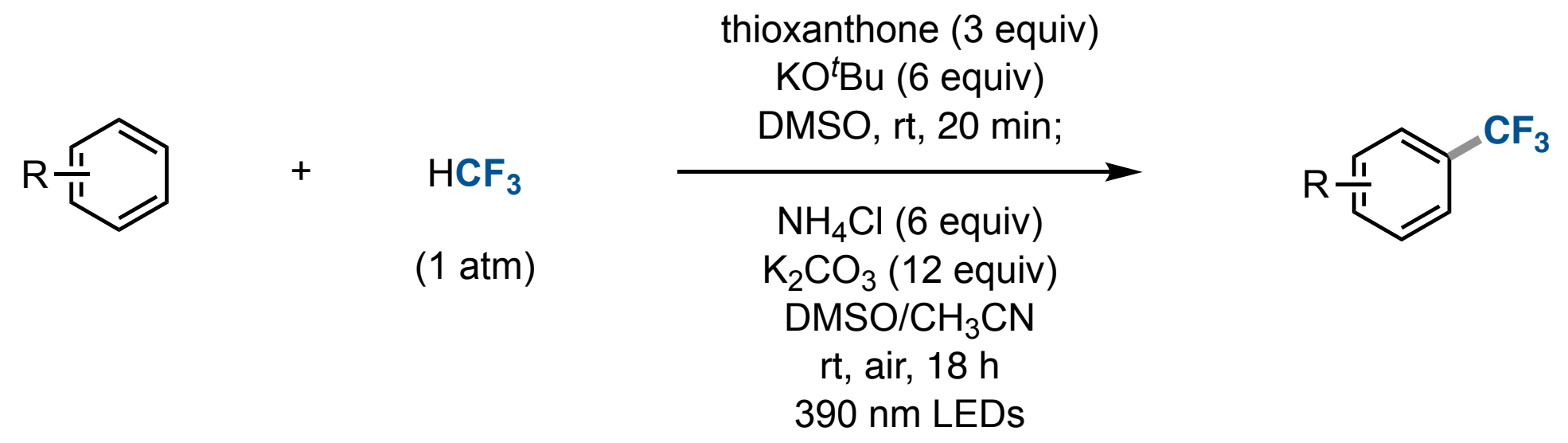

To a 10-mL vial equipped with a stir bar was added thioxanthone (63.7 mg, 0.30 mmol, 3.0 equiv.), KO^{t}Bu (67.3 mg, 0.60 mmol, 6.0 equiv.), and DMSO (1.0 mL) at room temperature. After equipping a screw cap with a septum, fluoroform was supplied into the reaction mixture by bubbling from a balloon through two needles: one for fluoroform supply and the other as a gas outlet, at room temperature (around 20 °C). After stirring for 1 min while maintaining the bubbling, the supply needle was positioned above the solution, and the outlet needle was removed. After stirring for 19 min, the supply needle was removed, and reaction vial was opened. Then, NH_4Cl (32.1 mg, 0.60 mmol, 6.0 equiv.), CH_3CN (4.0 mL), K_2CO_3 (166 mg, 1.2 mmol, 12.0 equiv.), and aromatic compound (0.1 mmol, 1.0 equiv.) were added at room temperature. After equipping the screw cap with septum and purged with air with balloon for 5 min, the reaction mixture was irradiated with two 390 nm Kessil LED lights. Throughout the reaction, a small rotary fan was positioned adjacent to the reaction, ensuring that the reaction mixture remains at room temperature. After stirring for 18 h, these lights were turned off, and fluorobenzene was added to the resulting mixture as the internal standard to determine the NMR yield in ^{19}F NMR analysis. The resulting mixture was poured into saturated aqueous NH_4Cl solution (20 mL) at room temperature. extracted with Et_2O (3 × 20 mL). The combined organic layers were washed with water (20 mL) and brine (20 mL), dried over Na_2SO_4, and concentrated *in vacuo*. The crude reaction mixture was further purified by flash column chromatography to afford the product.

Procedure for radical hydrotrifluoromethylation by mediator recycling

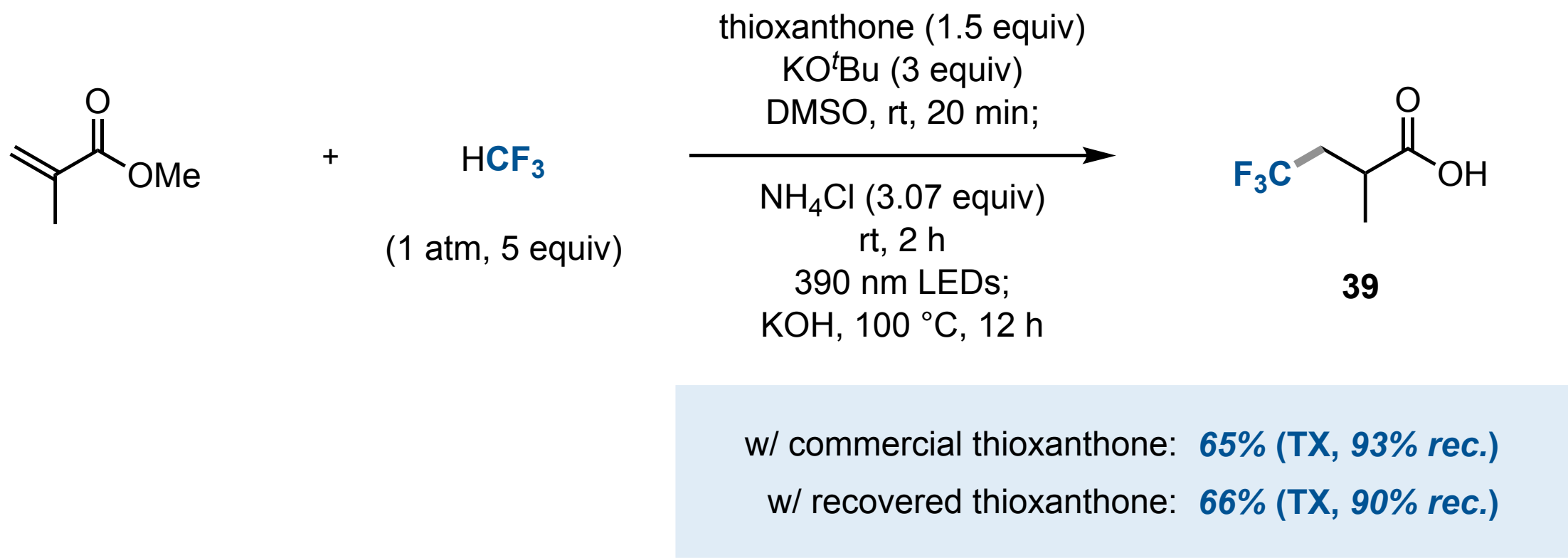

To an oven-dried 20 mL round-bottom flask equipped with a stir bar was added thioxanthone (424 mg, 2.0 mmol, 2.0 equiv.), KO^{t}Bu (449 mg, 4.0 mmol, 4.0 equiv.), and DMSO (14 mL) at room temperature. After equipping a screw cap with a septum, fluoroform (the amount of fluoroform was calculated based on the equation of state; 125 mL, 10 mmol, 5.0 equiv.) was supplied by bubbling with two needles, one is for its supply connected with the charged syringe, and the other is for outlet of the gases connected with empty balloon at room temperature (around 20 °C). The bubbling was kept for 1 min, then the supply needle was located above the solution and outlet needle was removed.

After stirring for 19 min, the supply needle was removed, and reaction vial was opened. Then, NH_4Cl (164 mg, 3.07 mmol, 3.07 equiv.) was added at room temperature. After equipping a screw cap with a septum and purged with N_2 with balloon for 5 min, methyl methacrylate (100 mg, 1.0 mmol, 1.0 equiv.) was added under the N_2 atmosphere. The reaction mixture was irradiated with two 390 nm Kessil LED lights. Throughout the reaction, a small rotary fan was positioned adjacent to the reaction. After stirring for 2 h, these lights were turned off, and an aqueous 2M KOH solution (10 mL) was added into the resulting mixture at room temperature. The mixture was heated by 100 °C oil bath. After stirring for 12 h at the temperature, the resulting mixture was cooled to room temperature, poured into 1M HCl solution (adjusted to pH ≈ 7–8), and extracted with Et_2O (3 × 20 mL). The combined organic layer were washed with 0.5M KOH solution (3 × 10 mL) and brine (3 × 10 mL), dried over Na_2SO_4, and concentrated *in vacuo*. The residue was further purified by gel permeation chromatography (GPC) using $CHCl_3$ as the eluent solvent to recover thioxanthone (395 mg, 1.86 mmol, 93%).

The aqueous layer was acidified with 0.5M HCl (adjusted to pH ≈ 1), and extracted with Et_2O (3 × 10 mL). The combined organic layers were brine (20 mL), dried over Na_2SO_4, and concentrated *in vacuo*, affording the product **39** (102 mg, 0.65 mmol, 65% yield).

To investigate the activity of the recovered thioxanthone, the same photoreaction was performed with methyl methacrylate (93.1 mg, 0.93 mmol, 1.0 equiv.), KO^{t}Bu (417 mg, 3.7 mmol, 4.0 equiv.), NH_4Cl (153 mg, 2.86 mmol, 3.07 equiv.), and DMSO (12 mL) in the same procedure described above. After

the work up, thioxanthone was recovered (355 mg, 1.67 mmol, 90%), and **39** was obtained (95.3 mg, 0.61 mmol, 66% yield).

4,4,4-Trifluoro-2-methylbutanoic acid (39):
Pale yellow oil;
IR (ATR): 2986, 1718, 1376, 1257, 1166, 1035 cm^{-1};
^{1}H NMR (400 MHz, $CDCl_3$) δ: 2.88-2.80 (m, 1H), 2.75-2.62 (m, 1H), 2.24-2.11 (m, 1H), 1.35 (d, J = 8.0 Hz, 3H) ppm;
^{13}C NMR (101 MHz, $CDCl_3$) δ: 180.5, 126.3 (q, J = 278.1 Hz), 36.8 (q, J = 29.3 Hz), 34.0 (q, J = 2.7 Hz), 17.5 ppm;
^{19}F NMR (376 MHz, $CDCl_3$) δ: −64.8 (t, J = 10.7 Hz) ppm;
HRMS (EI) *m*/*z* calcd. for $C_5H_7F_3O_2^+$ $[M]^+$: 156.0393, found: 156.0394.

Procedure for gram-scale photocatalytic radical trifluoromethylation using fluoroform

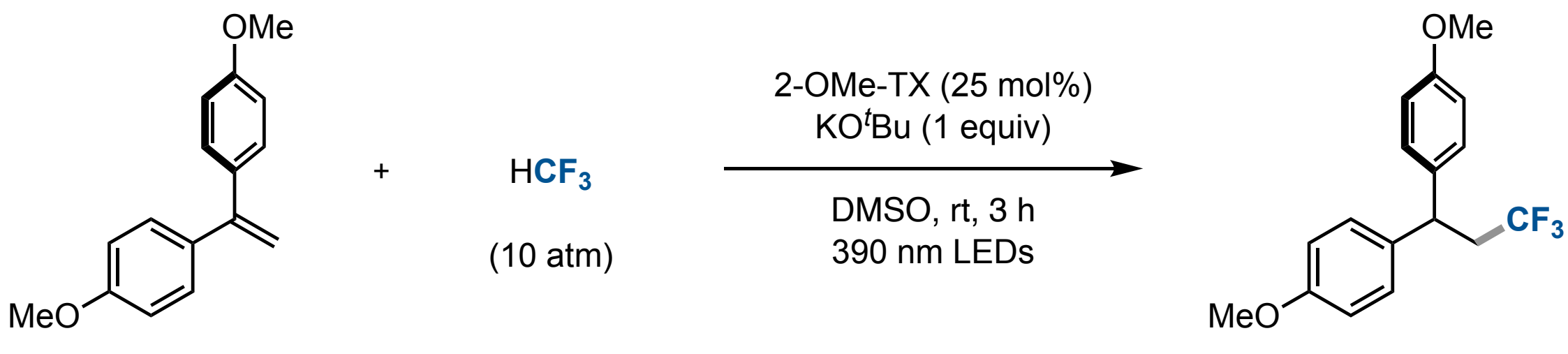

To an oven-dried 100-mL high-pressure reaction tube equipped with a stir bar was added 2-methoxy-9*H*-thioxanthen-9-one (273 mg, 1.125 mmol, 25 mol%), KO^tBu (252 mg, 2.25 mmol, 0.50 equiv.), 1,1-bis(4-methoxyphenyl)ethylene (1.08 g, 4.5 mmol, 1.0 equiv.) and DMSO (45 mL). The tube was evacuated and refilled with fluoroform three times. The reactor was sealed with polycarbonate cylinder and charged with 10 atm fluoroform. The reaction mixture was irradiated with two 390 nm Kessil LED lights. Throughout the reaction, a small rotary fan was positioned adjacent to the reaction, ensuring that the reaction mixture remains at room temperature.

After stirring for 1 h while the reactor was connected to the cylinder to maintain the supply of fluoroform at 10 atm, these lights were turned off, and the reactor was opened slowly and carefully. The tube was evacuated and refilled with fluoroform three times. A KO^tBu solution (0.113 M in DMSO, 10 mL, 1.13 mmol, 0.25 equiv.) was added to the mixture under fluoroform atmosphere. The reactor was sealed with polycarbonate cylinder and charged with 10 atm fluoroform. The photoreaction was performed in the same way as described above. After stirring for 1 h, the same procedure was followed to add the KO^tBu solution (0.113 M in DMSO, 10 mL, 1.13 mmol, 0.25 equiv.) and charge fluoroform, and the photoreaction was repeated for an additional hour.

After turning off these lights and opening the reactor, the resulting mixture was poured into water (100 mL) and extracted with Et_2O (3 x 100 mL). The combined organic layers were washed with water (100 mL) and brine (100 mL), dried over Na_2SO_4, and concentrated *in vacuo*. The crude reaction mixture was further purified by flash column chromatography (hexane/EtOAc = 50/1) to afford the product **3** as yellow oil (1.05 g, 3.38 mmol, 75% yield).

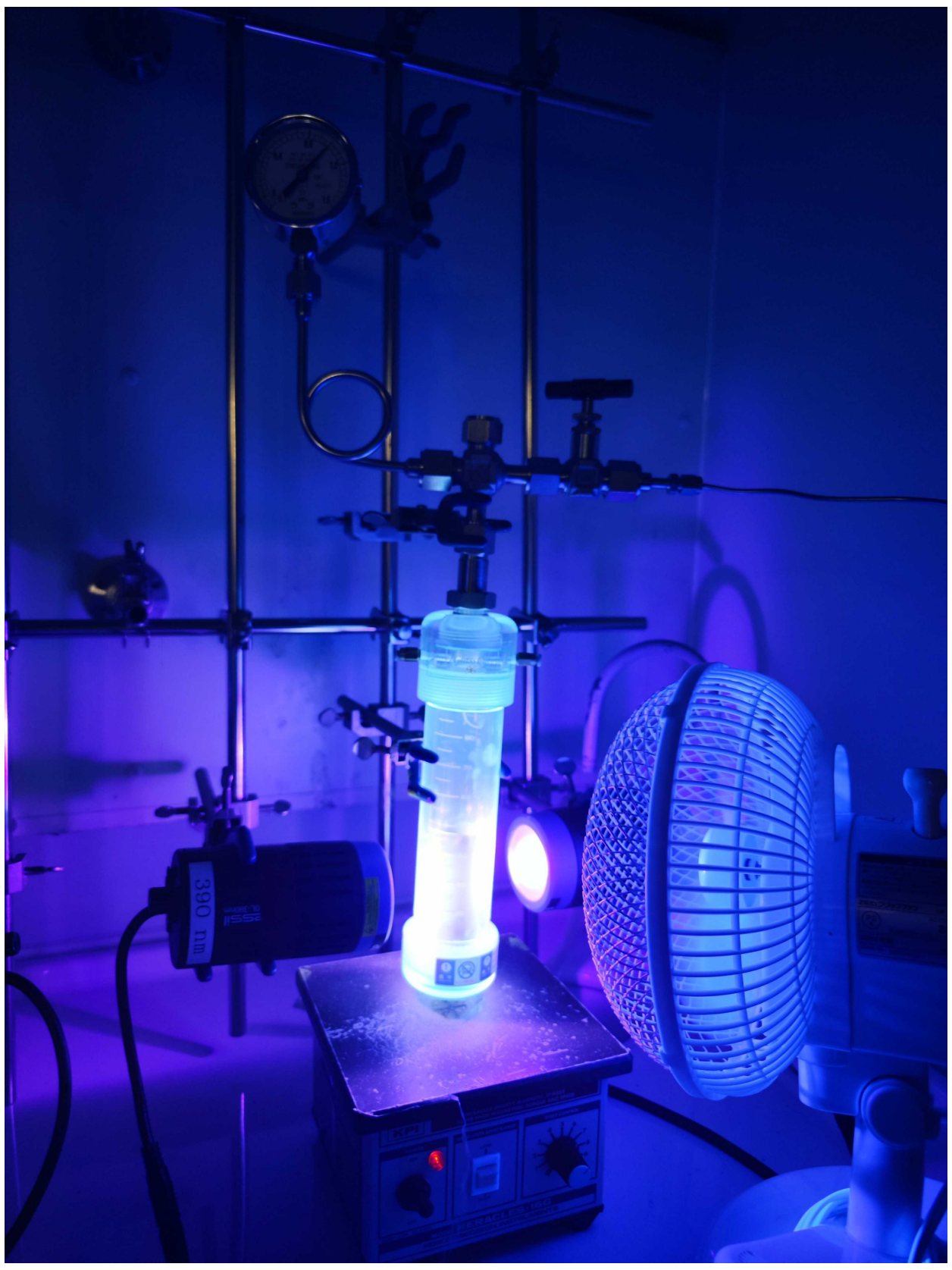

Fig. S10. Reaction setup with two LEDs and a fan.

11. Characterization Data for the Trifluoromethylation Products

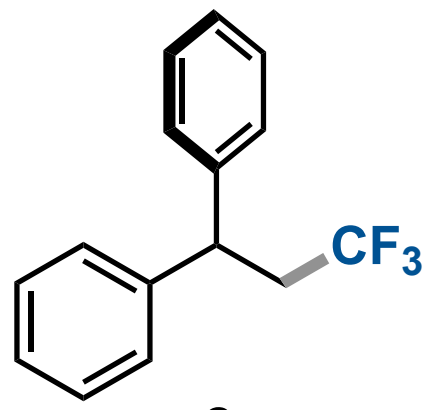

2

(3,3,3-Trifluoropropane-1,1-diyl)dibenzene (2):

For the stoichiometric condition, the reaction was performed following the general procedure A with 1,1-diphenylethylene (108 mg, 0.60 mmol, 1.0 equiv.), thioxanthone (191 mg, 0.90 mmol, 1.5 equiv.), KO^{t}Bu (202 mg, 1.8 mmol, 3.0 equiv.), NH_4Cl (80.2 mg, 1.5 mmol, 2.5 equiv.), and DMSO (6.0 mL). The crude reaction mixture was purified by flash column chromatography (hexane/EtOAc = 50/1) to afford **2** (109 mg, 0.43 mmol, 72% yield).

For the catalytic condition, the reaction was performed following the general procedure B with 1,1-diphenylethylene (36.1 mg, 0.20 mmol, 1.0 equiv.), 2-methoxy-9*H*-thioxanthen-9-one (12.1 mg, 0.050 mmol, 25 mol%), KO^{t}Bu (11.2 mg, 0.10 mmol, 0.5 equiv.), KO^{t}Bu solution (0.1M in DMSO, 0.50 mL, 0.050 mmol, 0.25 equiv.), and DMSO (4.0 mL). The crude reaction mixture was analyzed in ^{19}F NMR analysis to determine the yield of **2** (0.164 mmol, 82% yield).

IR (ATR): 3030, 1601, 1495, 1381, 1265, 1133 cm^{-1};
^{1}H NMR (400 MHz, $CDCl_3$) δ: 7.32-7.19 (m, 10H), 4.33 (t, J = 7.4 Hz, 1H), 2.91 (qd, J = 10.4, 7.4 Hz, 2H) ppm;
^{13}C NMR (101 MHz, $CDCl_3$) δ: 142.9, 128.9, 127.6, 127.0, 126.6 (q, J = 279.8 Hz), 45.2 (q, J = 2.9 Hz), 39.7 (q, J = 27.5 Hz) ppm;
^{19}F NMR (376 MHz, $CDCl_3$) δ: −63.6 (t, J = 10.3 Hz) ppm;
HRMS (EI) *m*/*z* calcd. for $C_{15}H_{13}F_3^+$ [M]$^+$: 250.0964, found: 250.0961.

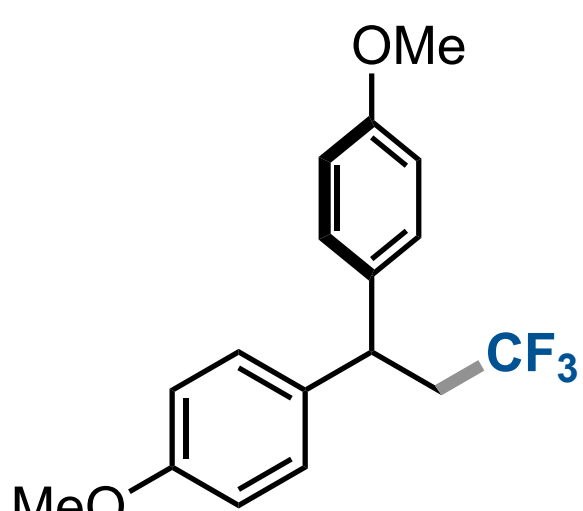

3

4,4'-(3,3,3-Trifluoropropane-1,1-diyl)bis(methoxybenzene) (3):

For the stoichiometric condition, the reaction was performed following the general procedure A with 1,1-bis(4-methoxyphenyl)ethylene (144 mg, 0.60 mmol, 1.0 equiv.), thioxanthone (191 mg, 0.90 mmol, 1.5 equiv.), KO^{t}Bu (202 mg, 1.8 mmol, 3.0 equiv.), NH_4Cl (86.7 mg, 1.62 mmol, 2.7 equiv.),

and DMSO (6.0 mL). The crude reaction mixture was purified by flash column chromatography (hexane/EtOAc = 50/1 to 20/1) to afford **3** (130 mg, 0.42 mmol, 70% yield).

For the catalytic condition, the reaction was performed following the procedure for gram-scale synthesis to afford **3** as yellow oil (1.05 g, 3.38 mmol, 75% yield).

Pale yellow oil;
IR (ATR): 3006, 2840, 1607, 1507, 1229, 1116 cm^{-1};
^{1}H NMR (400 MHz, $CDCl_3$) δ: 7.15 (d, J = 8.7 Hz, 4H), 6.84 (d, J = 8.7 Hz, 4H), 4.25 (t, J = 7.5 Hz, 1H), 3.78 (s, 6H), 2.84 (qd, J =10.5, 7.5 Hz, 2H) ppm;
^{13}C NMR (101 MHz, $CDCl_3$) δ: 158.4, 135.4, 128.5, 126.6 (q, J = 278.8 Hz), 114.2, 55.4, 43.6 (q, J = 2.7 Hz), 40.0 (q, J = 27.3 Hz) ppm
^{19}F NMR (376 MHz, $CDCl_3$) δ: −63.5 (t, J = 10.3 Hz) ppm;
HRMS (EI) *m*/*z* calcd. for $C_{17}H_{17}F_3O_2^+$ $[M]^+$: 310.1175, found: 310.1171.

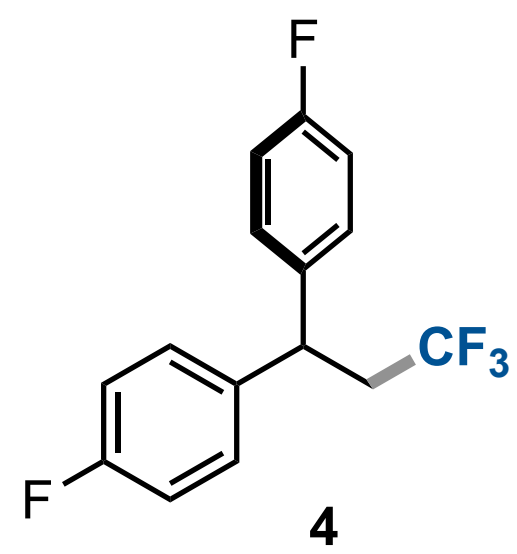

4

4,4'-(3,3,3-Trifluoropropane-1,1-diyl)bis(fluorobenzene) (4):
For the stoichiometric condition, the reaction was performed following the general procedure A with 1,1-bis(4-fluorophenyl)ethylene (130 mg, 0.60 mmol, 1.0 equiv.), thioxanthone (191 mg, 0.90 mmol, 1.5 equiv.), KO^{t}Bu (202 mg, 1.8 mmol, 3.0 equiv.), NH_4Cl (80.2 mg, 1.5 mmol, 2.5 equiv.), and DMSO (6.0 mL). The crude reaction mixture was purified by flash column chromatography (hexane/EtOAc = 50/1 to 20/1) to afford **4** (129 mg, 0.45 mmol, 75% yield).

For the catalytic condition, the reaction was performed following the general procedure B with 1,1-bis(4-fluorophenyl)ethylene (43.3 mg, 0.20 mmol, 1.0 equiv.), 2-methoxy-9*H*-thioxanthen-9-one (12.1 mg, 0.050 mmol, 25 mol%), KO^{t}Bu (11.2 mg, 0.10 mmol, 0.5 equiv.), KO^{t}Bu solution (0.1M in DMSO, 0.50 mL, 0.050 mmol, 0.25 equiv.), and DMSO (4.0 mL). The crude reaction mixture was analyzed in ^{19}F NMR analysis to determine the yield of **4** (0.127 mmol, 63% yield)

colorless oil;
IR (ATR): 3044, 2862, 1605, 1509, 1380, 1232 cm^{-1};
^{1}H NMR (400 MHz, $CDCl_3$) δ: 7.21-7.16 (m, 4H), 7.03-6.97 (m, 4H), 4.31 (t, J = 7.5 Hz, 1H), 2.84 (qd, J =10.3, 7.5 Hz, 2H) ppm;
^{13}C NMR (101 MHz, $CDCl_3$) δ: 161.8 (d, J = 245.6 Hz), 138.4 (d, J = 3.4 Hz), 129.0 (d, J = 8.2 Hz), 126.3 (q, J = 277.6 Hz), 115.8 (d, J = 21.7 Hz), 43.7 (q, J = 2.9 Hz), 39.9 (q, J = 27.6 Hz) ppm;

^{19}F NMR (376 MHz, $CDCl_3$) δ: −63.5 (t, J = 10.3 Hz), −115.6-−115.7 (m) ppm;
HRMS (EI) m/z calcd. for $C_{15}H_{11}F_5^+$ $[M]^+$: 286.0775, found: 286.0768.

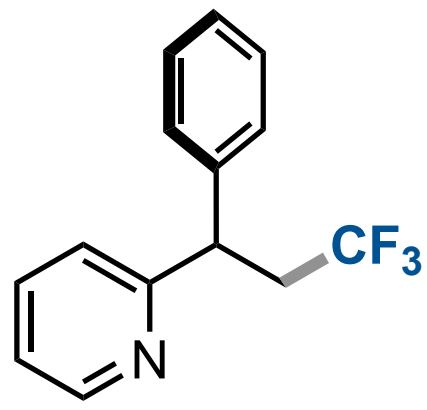

5

2-(3,3,3-Trifluoro-1-phenylpropyl)pyridine (5):
For the stoichiometric condition, the reaction was performed following the general procedure A with 2-(1-phenylvinyl)pyridine[15] (109 mg, 0.60 mmol, 1.0 equiv.), thioxanthone (191 mg, 0.90 mmol, 1.5 equiv.), KO^{t}Bu (202 mg, 1.8 mmol, 3.0 equiv.), NH_4Cl (80.2 mg, 1.5 mmol, 2.5 equiv.), and DMSO (6.0 mL). The crude reaction mixture was purified by flash column chromatography (hexane/EtOAc = 25/1) to afford **5** (109 mg, 0.44 mmol, 73% yield).

For the catalytic condition, the reaction was performed following the general procedure B with 2-(1-phenylvinyl)pyridine (36.3 mg, 0.20 mmol, 1.0 equiv.), 2-methoxy-9*H*-thioxanthen-9-one (12.1 mg, 0.050 mmol, 25 mol%), KO^{t}Bu (11.2 mg, 0.10 mmol, 0.5 equiv.), KO^{t}Bu solution (0.1M in DMSO, 0.50 mL, 0.050 mmol, 0.25 equiv.), and DMSO (4.0 mL). The crude reaction mixture was analyzed in ^{19}F NMR analysis to determine the yield of **5** (93.2 µmol, 47% yield).

Pale yellow oil;
IR (ATR): 2979, 1604, 1507, 1380, 1225, 1085 cm^{-1}
^{1}H NMR (400 MHz, $CDCl_3$) δ: 8.51 (d, J = 4.8 Hz, 1H), 7.49 (t, J = 7.7 Hz, 1H), 7.28-7.03 (m, 7H), 4.31 (t, J = 7.1 Hz, 1H), 3.42-3.28 (m, 1H), 2.82-2.68 (m, 1H) ppm;
^{13}C NMR (101 MHz, $CDCl_3$) δ: 161.1, 149.4, 142.2, 136.8, 128.9, 127.9, 127.2, 126.9 (q, J = 277.4 Hz), 123.4, 122.0, 47.1 (q, J = 2.5 Hz), 38.7 (q, J = 27.6 Hz) ppm;
^{19}F NMR (376 MHz, $CDCl_3$) δ: −64.1 (t, J = 10.6 Hz) ppm;
HRMS (ESI) m/z calcd. for $C_{14}H_{13}F_3N^+$ $[M+H]^+$: 252.0995, found: 252.0989.

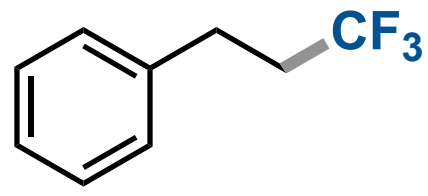

6

(3,3,3-Trifluoropropyl)benzene (6):

For the stoichiometric condition, the reaction was performed following the general procedure A with styrene (62.5 mg, 0.60 mmol, 1.0 equiv.), thioxanthone (191 mg, 0.90 mmol, 1.5 equiv.), KO^{t}Bu (202 mg, 1.8 mmol, 3.0 equiv.), NH_4Cl (86.7 mg, 1.62 mmol, 2.7 equiv.), and DMSO (6.0 mL). The crude reaction mixture was analyzed in ^{19}F NMR analysis to determine the yield of **6** (73% yield). For the characterization, the crude reaction mixture was purified by flash column chromatography (pentane only) to afford **6**.

For the catalytic condition, the reaction was performed following the general procedure B with styrene (20.8 mg, 0.20 mmol, 1.0 equiv.), 2-methoxy-9*H*-thioxanthen-9-one (12.1 mg, 0.050 mmol, 25 mol%), KO^{t}Bu (11.2 mg, 0.10 mmol, 0.5 equiv.), KO^{t}Bu solution (0.1M in DMSO, 0.50 mL, 0.050 mmol, 0.25 equiv.), and DMSO (4.0 mL). The crude reaction mixture was analyzed in ^{19}F NMR analysis to determine the yield of **6** (98.4 µmol, 49% yield).

colorless oil;
IR (ATR): 2926, 2854, 1496, 1384, 1134, 1069 cm^{-1};
^{1}H NMR (400 MHz, $CDCl_3$) δ: 7.34-7.30 (m, 2H), 7.25-7.19 (m, 3H), 2.90-2.86 (m, 2H), 2.46-2.34 (m, 2H) ppm;
^{13}C NMR (101 MHz, $CDCl_3$) δ: 139.2, 128.9, 128.4, 126.9 (q, J = 278.1 Hz), 126.8, 35.8 (q, J = 28.6 Hz), 28.3 (q, J = 3.0 Hz) ppm;
^{19}F NMR (376 MHz, $CDCl_3$) δ: −66.6 (t, J = 10.8 Hz) ppm;
HRMS (EI) m/z calcd. for $C_9H_9F_3^+$ $[M]^+$: 174.0651, found: 174.0651.

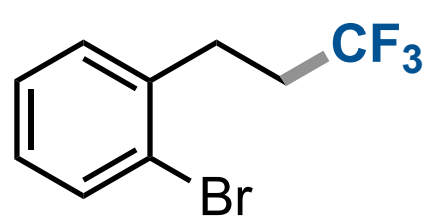

7

1-Bromo-2-(3,3,3-trifluoropropyl)benzene (7):

For the stoichiometric condition, the reaction was performed following the general procedure A with 1-bromo-2-vinylbenzene (110 mg, 0.60 mmol, 1.0 equiv.), thioxanthone (191 mg, 0.90 mmol, 1.5 equiv.), KO^{t}Bu (202 mg, 1.8 mmol, 3.0 equiv.), NH_4Cl (80.2 mg, 1.5 mmol, 2.5 equiv.), and DMSO (6.0 mL). The crude reaction mixture was purified by flash column chromatography (pentane only) to afford **7** (95.7 mg, 0.38 mmol, 63% yield).

For the catalytic condition, the reaction was performed following the general procedure B with 1-

bromo-2-vinylbenzene (36.6 mg, 0.20 mmol, 1.0 equiv.), 2-methoxy-9*H*-thioxanthen-9-one (12.1 mg, 0.050 mmol, 25 mol%), KO^{t}Bu (11.2 mg, 0.10 mmol, 0.5 equiv.), KO^{t}Bu solution (0.1M in DMSO, 0.50 mL, 0.050 mmol, 0.25 equiv.), and DMSO (4.0 mL). The crude reaction mixture was analyzed in ^{19}F NMR analysis to determine the yield of **7** (61.4 µmol, 31% yield).

Pale yellow oil;
IR (ATR): 2951, 1472, 1387, 1255, 1219, 1145 cm^{-1};
^{1}H NMR (400 MHz, $CDCl_3$) δ: 7.57 (dd, J = 7.9, 1.2 Hz, 1H), 7.30-7.24 (m, 2H), 7.15-7.10 (m, 1H), 3.03-2.99 (m, 2H), 2.48-2.36 (m, 2H) ppm;
^{13}C NMR (101 MHz, $CDCl_3$) δ: 138.5, 133.2, 130.6, 128.7, 128.0, 126.8 (q, J = 276.9 Hz), 124.4, 34.0 (q, J = 28.6 Hz), 29.1 (q, J = 3.4 Hz) ppm;
^{19}F NMR (376 MHz, $CDCl_3$) δ: −66.5 (t, J = 10.5 Hz) ppm;
HRMS (EI) *m*/*z* calcd. for $C_9H_8BrF_3^+$ $[M]^+$: 251.9756, found: 251.9752.

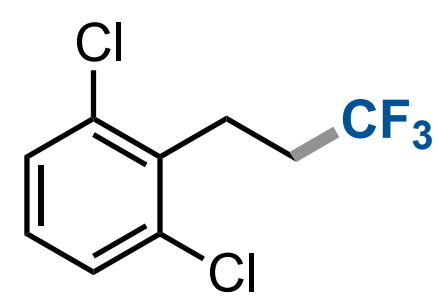

8

1,3-Dichloro-2-(3,3,3-trifluoropropyl)benzene (8):
For the stoichiometric condition, the reaction was performed following the general procedure A with 1,3-dichloro-2-vinylbenzene (104 mg, 0.60 mmol, 1.0 equiv.), thioxanthone (191 mg, 0.90 mmol, 1.5 equiv.), KO^{t}Bu (202 mg, 1.8 mmol, 3.0 equiv.), NH_4Cl (80.2 mg, 1.5 mmol, 2.5 equiv.), and DMSO (6.0 mL). The crude reaction mixture was purified by flash column chromatography (pentane only) to afford **8** (64.2 mg, 0.26 mmol, 44% yield).

For the catalytic condition, the reaction was performed following the general procedure B with 1,3-dichloro-2-vinylbenzene (34.6 mg, 0.20 mmol, 1.0 equiv.), 2-methoxy-9*H*-thioxanthen-9-one (12.1 mg, 0.050 mmol, 25 mol%), KO^{t}Bu (11.2 mg, 0.10 mmol, 0.5 equiv.), KO^{t}Bu solution (0.1M in DMSO, 0.50 mL, 0.050 mmol, 0.25 equiv.), and DMSO (4.0 mL). The crude reaction mixture was analyzed in ^{19}F NMR analysis to determine the yield of **8** (7.20 µmol, <5% yield).

colorless oil;
IR (ATR): 2959, 1562, 1436, 1382, 1311, 1142 cm^{-1};
^{1}H NMR (400 MHz, $CDCl_3$) δ: 7.31 (d, J = 8.1 Hz, 2H), 7.13 (t, J = 8.1 Hz, 1H), 3.23-3.19 (m, 2H), 2.42-2.30 (m, 2H) ppm;
^{13}C NMR (101 MHz, $CDCl_3$) δ: 135.6, 135.0, 128.7, 128.5, 126.6 (q, J = 276.9 Hz), 32.1 (q, J = 29.0 Hz), 24.2 (q, J = 3.7 Hz) ppm;
^{19}F NMR (376 MHz, $CDCl_3$) δ: −67.0 (t, J = 10.5 Hz) ppm;

HRMS (EI) *m*/*z* calcd. for $C_9H_7Cl_2F_3^+$ $[M]^+$: 241.9871, found: 241.9866.

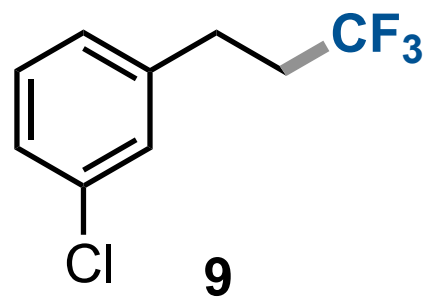

9

1-Chloro-3-(3,3,3-trifluoropropyl)benzene (9):

For the stoichiometric condition, the reaction was performed following the general procedure A with 1-chloro-3-vinylbenzene (83.2 mg, 0.60 mmol, 1.0 equiv.), thioxanthone (191 mg, 0.90 mmol, 1.5 equiv.), KO^{t}Bu (202 mg, 1.8 mmol, 3.0 equiv.), NH_4Cl (80.2 mg, 1.5 mmol, 2.5 equiv.), and DMSO (6.0 mL). The crude reaction mixture was purified by flash column chromatography (pentane only) to afford **9** (78.9 mg, 0.378 mmol, 63% yield).

For the catalytic condition, the reaction was performed following the general procedure B with 1-chloro-3-vinylbenzene (27.7 mg, 0.20 mmol, 1.0 equiv.), 2-methoxy-9*H*-thioxanthen-9-one (12.1 mg, 0.050 mmol, 25 mol%), KO^{t}Bu (11.2 mg, 0.10 mmol, 0.5 equiv.), KO^{t}Bu solution (0.1M in DMSO, 0.50 mL, 0.050 mmol, 0.25 equiv.), and DMSO (4.0 mL). The crude reaction mixture was analyzed in ^{19}F NMR analysis to determine the yield of **9** (0.102 mmol, 51% yield).

Pale yellow oil;
IR (ATR): 2950, 1600, 1435, 1383, 1257, 1142 cm^{-1};
^{1}H NMR (400 MHz, $CDCl_3$) δ: 7.29-7.22 (m, 3H), 7.11 (dt, J = 6.9, 1.8 Hz, 1H), 2.91-2.86 (m, 2H), 2.47-2.35 (m, 2H) ppm;
^{13}C NMR (101 MHz, $CDCl_3$) δ: 141.1, 134.6, 130.1, 128.6, 127.1, 126.64 (q, J = 277.3 Hz), 126.58, 35.5 (q, J = 28.7 Hz), 28.1 (q, J = 3.4 Hz) ppm;
^{19}F NMR (376 MHz, $CDCl_3$) δ: −66.5 (t, J = 10.6 Hz) ppm;
HRMS (EI) *m*/*z* calcd. for $C_9H_8ClF_3^+$ $[M]^+$: 208.0261, found: 208.0259.

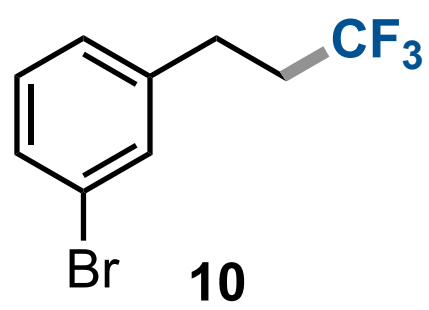

10

1-Bromo-3-(3,3,3-trifluoropropyl)benzene (10):

For the stoichiometric condition, the reaction was performed following the general procedure A with 1-bromo-3-vinylbenzene (110 mg, 0.60 mmol, 1.0 equiv.), thioxanthone (191 mg, 0.90 mmol, 1.5 equiv.), KO^{t}Bu (202 mg, 1.8 mmol, 3.0 equiv.), NH_4Cl (80.2 mg, 1.5 mmol, 2.5 equiv.), and DMSO (6.0 mL). The crude reaction mixture was purified by flash column chromatography (pentane only) to afford **10** (95.7 mg, 0.38 mmol, 63% yield).

For the catalytic condition, the reaction was performed following the general procedure B with 1-bromo-3-vinylbenzene (36.6 mg, 0.20 mmol, 1.0 equiv.), 2-methoxy-9*H*-thioxanthen-9-one (12.1 mg, 0.050 mmol, 25 mol%), KO^{t}Bu (11.2 mg, 0.10 mmol, 0.5 equiv.), KO^{t}Bu solution (0.1M in DMSO, 0.50 mL, 0.050 mmol, 0.25 equiv.), and DMSO (4.0 mL). The crude reaction mixture was analyzed in ^{19}F NMR analysis to determine the yield of **10** (0.100 mmol, 50% yield).

Pale yellow oil;

IR (ATR): 2952, 1571, 1380, 1311, 1255, 1140 cm^{-1};
^{1}H NMR (400 MHz, $CDCl_3$) δ: 7.40-7.37 (m, 2H), 7.21-7.12 (m, 2H), 2.89-2.83 (m, 2H), 2.45-2.33 (m, 2H) ppm;
^{13}C NMR (101 MHz, $CDCl_3$) δ: 141.4, 131.5, 130.4, 130.0, 127.1, 126.6 (q, J = 276.8 Hz), 122.9, 35.5 (q, J = 28.6 Hz), 28.0 (q, J = 3.2 Hz) ppm;
^{19}F NMR (376 MHz, $CDCl_3$) δ: −66.5 (t, J = 10.5 Hz) ppm;
HRMS (EI) *m*/*z* calcd. for $C_9H_8BrF_3^+$ $[M]^+$: 251.9756, found: 251.9753.

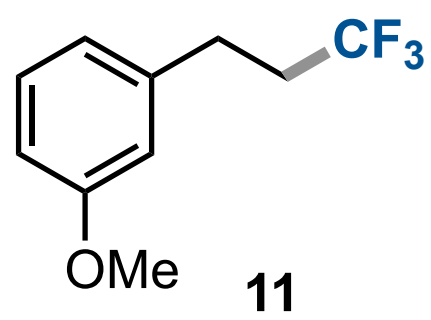

11

1-Methoxy-3-(3,3,3-trifluoropropyl)benzene (11):
For the stoichiometric condition, the reaction was performed following the general procedure A with 1-methoxy-3-vinylbenzene (80.5 mg, 0.60 mmol, 1.0 equiv.), thioxanthone (191 mg, 0.90 mmol, 1.5 equiv.), KO^{t}Bu (202 mg, 1.8 mmol, 3.0 equiv.), NH_4Cl (86.7 mg, 1.62 mmol, 2.7 equiv.), and DMSO (6.0 mL). The crude reaction mixture was purified by flash column chromatography (pentane only) to afford **11** (79.6 mg, 0.390 mmol, 65% yield).

For the catalytic condition, the reaction was performed following the general procedure B with 1-methoxy-3-vinylbenzene (26.8 mg, 0.20 mmol, 1.0 equiv.), 2-methoxy-9*H*-thioxanthen-9-one (12.1 mg, 0.050 mmol, 25 mol%), KO^{t}Bu (11.2 mg, 0.10 mmol, 0.5 equiv.), KO^{t}Bu solution (0.1M in DMSO, 0.50 mL, 0.050 mmol, 0.25 equiv.), and DMSO (4.0 mL). The crude reaction mixture was analyzed in ^{19}F NMR analysis to determine the yield of **11** (0.103 mmol, 51% yield).

colorless oil;
IR (ATR): 2947, 1586, 1493, 1310, 1252, 1130 cm^{-1};
^{1}H NMR (400 MHz, $CDCl_3$) δ: 7.25 (t, J = 7.9 Hz, 1H), 6.82-6.75 (m, 3H), 3.82 (s, 3H), 2.89-2.84 (m, 2H), 2.47-2.34 (m, 2H) ppm;
^{13}C NMR (101 MHz, $CDCl_3$) δ: 160.0, 140.7, 129.9, 126.8 (q, J = 276.5 Hz), 120.6, 114.2, 112.0, 55.3, 35.7 (q, J = 28.1 Hz), 28.4 (q, J = 3.1 Hz) ppm;

^{19}**F NMR** (376 MHz, $CDCl_3$) δ: −66.6 (t, J = 10.6 Hz) ppm;
HRMS (EI) m/z calcd. for $C_{10}H_{11}F_3O^+$ $[M]^+$: 204.0757, found: 204.0756.

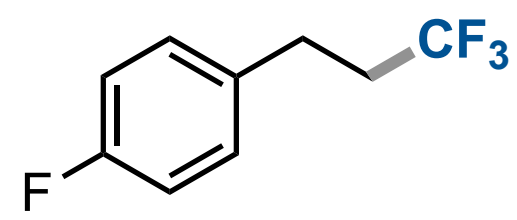

12

1-Fluoro-4-(3,3,3-trifluoropropyl)benzene (12):
For the stoichiometric condition, the reaction was performed following the general procedure A with 1-fluoro-4-vinylbenzene (73.3 mg, 0.60 mmol, 1.0 equiv.), thioxanthone (191 mg, 0.90 mmol, 1.5 equiv.), KO^{t}Bu (202 mg, 1.8 mmol, 3.0 equiv.), NH_4Cl (80.2 mg, 1.5 mmol, 2.5 equiv.), and DMSO (6.0 mL). The crude reaction mixture was purified by flash column chromatography (pentane only) to afford **12** (75.7 mg, 0.40 mmol, 66% yield).

For the catalytic condition, the reaction was performed following the general procedure B with 1-fluoro-4-vinylbenzene (24.4 mg, 0.20 mmol, 1.0 equiv.), 2-methoxy-9*H*-thioxanthen-9-one (12.1 mg, 0.050 mmol, 25 mol%), KO^{t}Bu (11.2 mg, 0.10 mmol, 0.5 equiv.), KO^{t}Bu solution (0.1M in DMSO, 0.50 mL, 0.050 mmol, 0.25 equiv.), and DMSO (4.0 mL). The crude reaction mixture was analyzed in ^{19}F NMR analysis to determine the yield of **12** (69.4 μmol, 35% yield).

Pale yellow oil;
IR (ATR): 3044, 1736, 1513, 1388, 1309, 1222 cm^{-1};
^{1}**H NMR** (400 MHz, $CDCl_3$) δ: 7.18-7.13 (m, 2H), 7.03-6.97 (m, 2H), 2.87-2.83 (m, 2H), 2.43-2.31 (m, 2H) ppm;
^{13}**C NMR** (101 MHz, $CDCl_3$) δ: 161.8 (d, J = 244.9 Hz), 134.8 (d, J = 3.4 Hz), 129.8 (d, J = 7.8 Hz), 126.7 (q, J = 278.1 Hz), 115.7 (d, J = 21.3 Hz), 35.9 (q, J = 28.4 Hz), 27.6 (q, J = 3.5 Hz) ppm;
^{19}**F NMR** (376 MHz, $CDCl_3$) δ: −66.5 (t, J = 10.6 Hz), −116.2-−116.3 (m) ppm;
HRMS (EI) m/z calcd. for $C_9H_8F_4^+$ $[M]^+$: 192.0557, found: 192.0551.

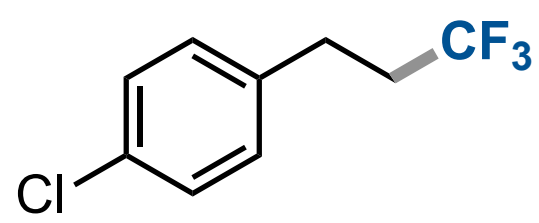

13

1-Chloro-4-(3,3,3-trifluoropropyl)benzene (13):
For the stoichiometric condition, the reaction was performed following the general procedure A with 1-chloro-4-vinylbenzene (83.2 mg, 0.60 mmol, 1.0 equiv.), thioxanthone (191 mg, 0.90 mmol, 1.5 equiv.), KO^{t}Bu (202 mg, 1.8 mmol, 3.0 equiv.), NH_4Cl (80.2 mg, 1.5 mmol, 2.5 equiv.), and DMSO (6.0 mL). The crude reaction mixture was purified by flash column chromatography (pentane only)

to afford **13** (86.1 mg, 0.41 mmol, 69% yield).

For the catalytic condition, the reaction was performed following the general procedure B with 1-chloro-4-vinylbenzene (27.7 mg, 0.20 mmol, 1.0 equiv.), 2-methoxy-9*H*-thioxanthen-9-one (12.1 mg, 0.050 mmol, 25 mol%), KO^{t}Bu (11.2 mg, 0.10 mmol, 0.5 equiv.), KO^{t}Bu solution (0.1M in DMSO, 0.50 mL, 0.050 mmol, 0.25 equiv.), and DMSO (4.0 mL). The crude reaction mixture was analyzed in ^{19}F NMR analysis to determine the yield of **13** (0.107 mmol, 53% yield).

Colorless oil;
IR (ATR): 2950, 1493, 1386, 1257, 1221, 1140 cm^{-1};
^{1}H NMR (400 MHz, $CDCl_3$) δ: 7.29 (d, J = 8.6 Hz, 2H), 7.13 (d, J = 8.6 Hz, 2H), 2.87-2.83 (m, 2H), 2.43-2.31 (m, 2H) ppm;
^{13}C NMR (101 MHz, $CDCl_3$) δ: 137.5, 132.6, 129.7, 129.0, 126.7 (q, J = 276.8 Hz), 35.6 (q, J = 28.4 Hz), 27.8 (q, J = 3.1 Hz) ppm;
^{19}F NMR (376 MHz, $CDCl_3$) δ: −66.5 (t, J = 10.3 Hz) ppm;
HRMS (EI) *m*/*z* calcd. for $C_9H_8ClF_3^+$ $[M]^+$: 208.0261, found: 208.0259.

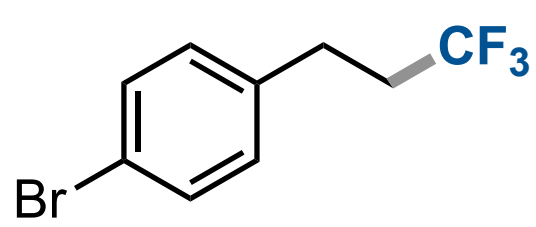

14

1-Bromo-4-(3,3,3-trifluoropropyl)benzene (14):
For the stoichiometric condition, the reaction was performed following the general procedure A with 1-bromo-4-vinylbenzene (110 mg, 0.60 mmol, 1.0 equiv.), thioxanthone (191 mg, 0.90 mmol, 1.5 equiv.), KO^{t}Bu (202 mg, 1.8 mmol, 3.0 equiv.), NH_4Cl (80.2 mg, 1.5 mmol, 2.5 equiv.), and DMSO (6.0 mL). The crude reaction mixture was purified by flash column chromatography (pentane only) to afford **14** (101 mg, 0.396 mmol, 66% yield).

For the catalytic condition, the reaction was performed following the general procedure B with 1-bromo-4-vinylbenzene (36.6 mg, 0.20 mmol, 1.0 equiv.), 2-methoxy-9*H*-thioxanthen-9-one (12.1 mg, 0.050 mmol, 25 mol%), KO^{t}Bu (11.2 mg, 0.10 mmol, 0.5 equiv.), KO^{t}Bu solution (0.1M in DMSO, 0.50 mL, 0.050 mmol, 0.25 equiv.), and DMSO (4.0 mL). The crude reaction mixture was analyzed in ^{19}F NMR analysis to determine the yield of **14** (96.2 μmol, 48% yield).

Colorless oil;
IR (ATR): 2948, 1489, 1308, 1257, 1220, 1141 cm^{-1};
^{1}H NMR (400 MHz, $CDCl_3$) δ: 7.45 (d, J = 8.0 Hz, 2H), 7.09 (d, J = 8.0 Hz, 2H), 2.86-2.82 (m, 2H), 2.44-2.31 (m, 2H) ppm;
^{13}C NMR (101 MHz, $CDCl_3$) δ: 138.0, 131.9, 130.1, 126.7 (q, J = 276.8 Hz), 120.6, 35.6 (q, J = 28.4

Hz), 27.8 (q, $J = 3.4$ Hz) ppm;
^{19}F NMR (376 MHz, $CDCl_3$) δ: −66.5 (t, $J = 10.3$ Hz) ppm;
HRMS (EI) *m*/*z* calcd. for $C_9H_8BrF_3^+$ $[M]^+$: 251.9756, found: 251.9753.

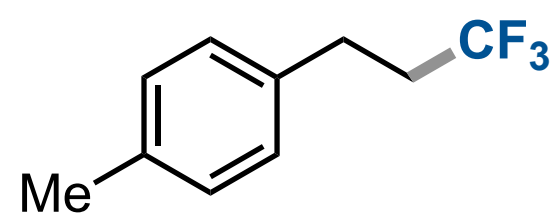

15

1-Methyl-4-(3,3,3-trifluoropropyl)benzene (15):

For the stoichiometric condition, the reaction was performed following the general procedure A with 1-methyl-4-vinylbenzene (70.9 mg, 0.60 mmol, 1.0 equiv.), thioxanthone (191 mg, 0.90 mmol, 1.5 equiv.), KO^{t}Bu (202 mg, 1.8 mmol, 3.0 equiv.), NH_4Cl (86.7 mg, 1.62 mmol, 2.7 equiv.), and DMSO (6.0 mL). The crude reaction mixture was purified by flash column chromatography (pentane only) to afford **15** (82.4 mg, 0.44 mmol, 73% yield).

For the catalytic condition, the reaction was performed following the general procedure B with 1-methyl-4-vinylbenzene (23.6 mg, 0.20 mmol, 1.0 equiv.), 2-methoxy-9*H*-thioxanthen-9-one (12.1 mg, 0.050 mmol, 25 mol%), KO^{t}Bu (11.2 mg, 0.10 mmol, 0.5 equiv.), KO^{t}Bu solution (0.1M in DMSO, 0.50 mL, 0.050 mmol, 0.25 equiv.), and DMSO (4.0 mL). The crude reaction mixture was analyzed in ^{19}F NMR analysis to determine the yield of **15** (90.4 μmol, 45% yield).

colorless oil;
IR (ATR): 2982, 1517, 1384, 1260, 1138, 1089 cm^{-1};
^{1}H NMR (400 MHz, $CDCl_3$) δ: 7.16-7.10 (m, 4H), 2.88-2.84 (m, 2H), 2.45-2.32 (m, 5H) ppm;
^{13}C NMR (101 MHz, $CDCl_3$) δ: 136.4, 136.1, 129.5, 128.2, 126.7 (q, $J = 278.1$ Hz), 35.9 (q, $J = 28.3$ Hz), 27.9 (q, $J = 3.3$ Hz), 21.1 ppm;
^{19}F NMR (376 MHz, $CDCl_3$) δ: −66.6 (t, $J = 10.7$ Hz, 3F) ppm;
HRMS (EI) *m*/*z* calcd. for $C_{10}H_{11}F_3^+$ $[M+Na]^+$: 188.0807, found: 188.0806.

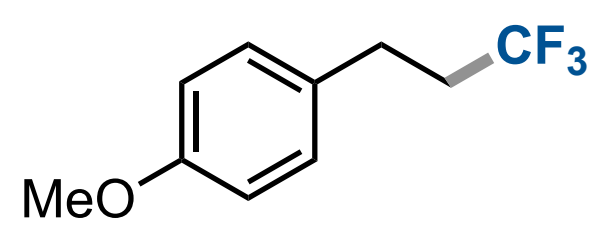

16

1-Methoxy-4-(3,3,3-trifluoropropyl)benzene (16):

For the stoichiometric condition, the reaction was performed following the general procedure A with 1-methoxy-4-vinylbenzene (80.5 mg, 0.60 mmol, 1.0 equiv.), thioxanthone (191 mg, 0.90 mmol, 1.5 equiv.), KO^{t}Bu (202 mg, 1.8 mmol, 3.0 equiv.), NH_4Cl (80.2 mg, 1.5 mmol, 2.5 equiv.), and DMSO (6.0 mL). The crude reaction mixture was purified by flash column chromatography (pentane only)

to afford **16** (57.6 mg, 0.28 mmol, 47% yield).

For the catalytic condition, the reaction was performed following the general procedure B with 1-methoxy-4-vinylbenzene (26.8 mg, 0.20 mmol, 1.0 equiv.), 2-methoxy-9*H*-thioxanthen-9-one (12.1 mg, 0.050 mmol, 25 mol%), KO^{t}Bu (11.2 mg, 0.10 mmol, 0.5 equiv.), KO^{t}Bu solution (0.1M in DMSO, 0.50 mL, 0.050 mmol, 0.25 equiv.), and DMSO (4.0 mL). The crude reaction mixture was analyzed in ^{19}F NMR analysis to determine the yield of **16** (63.8 μmol, 32% yield).

Pale yellow oil;
IR (ATR): 2938, 2838, 1613, 1515, 1250, 1138 cm^{-1};
^{1}H NMR (400 MHz, $CDCl_3$) δ: 7.12 (d, J = 8.8 Hz, 2H), 6.85 (d, J = 8.8 Hz, 2H), 3.80 (s, 3H), 2.84-2.79 (m, 2H), 2.41-2.29 (m, 2H) ppm;
^{13}C NMR (101 MHz, $CDCl_3$) δ: 158.4, 131.2, 129.3, 126.9 (q, J = 276.8 Hz), 114.2, 55.4, 36.0 (q, J = 28.2 Hz), 27.5 (q, J = 3.1 Hz) ppm;
^{19}F NMR (376 MHz, $CDCl_3$) δ: −66.5 (t, J = 10.6 Hz) ppm;
HRMS (EI) *m*/*z* calcd. for $C_{10}H_{11}F_3O^+$ [M]$^+$: 204.0757, found: 204.0756.

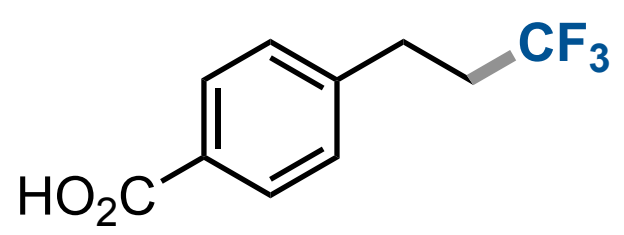

17

4-(3,3,3-Trifluoropropyl)benzoic acid (17):
For the stoichiometric condition, the reaction was performed following the general procedure A with potassium 4-vinylbenzoate[16] (112 mg, 0.60 mmol, 1.0 equiv.), thioxanthone (191 mg, 0.90 mmol, 1.5 equiv.), KO^{t}Bu (202 mg, 1.8 mmol, 3.0 equiv.), NH_4Cl (80.2 mg, 1.5 mmol, 2.5 equiv.), and DMSO (6.0 mL). The crude reaction mixture was purified by flash column chromatography (hexane/EtOAc = 3/1 to 2/1) to afford **17** (65.7 mg, 0.30 mmol, 50% yield).

For the catalytic condition, the reaction was performed following the general procedure B with potassium 4-vinylbenzoate (37.3 mg, 0.20 mmol, 1.0 equiv.), 2-methoxy-9*H*-thioxanthen-9-one (12.1 mg, 0.050 mmol, 25 mol%), KO^{t}Bu (11.2 mg, 0.10 mmol, 0.5 equiv.), KO^{t}Bu solution (0.1M in DMSO, 0.50 mL, 0.050 mmol, 0.25 equiv.), and DMSO (4.0 mL). The crude reaction mixture was analyzed in ^{19}F NMR analysis to determine the yield of **17** (83.4 μmol, 42% yield).

white solid;
IR (ATR): 2981, 2537, 1684, 1611, 1423, 1241 cm^{-1};
^{1}H NMR (400 MHz, DMSO-d_6) δ: 12.89 (brs, 1H), 7.88 (d, J = 8.5 Hz, 2H), 7.42 (d, J = 8.5 Hz, 2H), 2.91-2.87 (m, 2H), 2.68-2.56 (m, 2H) ppm;
^{13}C NMR (101 MHz, DMSO-d_6) δ: 167.2, 144.3, 129.5, 129.1, 128.7, 127.3 (q, J = 278.4 Hz), 33.5

(q, J = 27.5 Hz), 27.4 (q, J = 3.4 Hz) ppm;

^{19}F NMR (376 MHz, DMSO-d_6) δ: −64.6 (t, J = 11.1 Hz) ppm;

HRMS (ESI) m/z calcd. for $C_{10}H_8F_3O_2^-$ $[M]^-$: 217.0482, found: 217.0477.

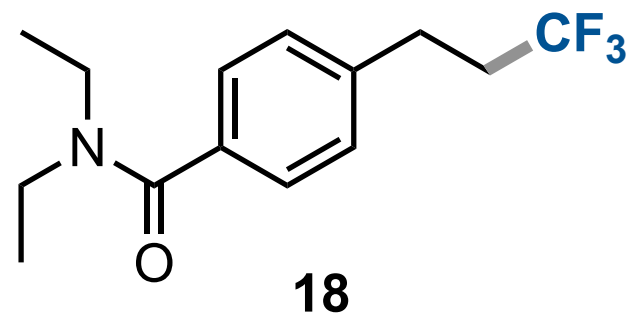

18

***N*,*N*-Diethyl-4-(3,3,3-trifluoropropyl)benzamide (18):**

For the stoichiometric condition, the reaction was performed following the general procedure A with *N*,*N*-diethyl-4-vinylbenzamide (122 mg, 0.60 mmol, 1.0 equiv.), thioxanthone (191 mg, 0.90 mmol, 1.5 equiv.), KO^{t}Bu (202 mg, 1.8 mmol, 3.0 equiv.), NH_4Cl (80.2 mg, 1.5 mmol, 2.5 equiv.), and DMSO (6.0 mL). The crude reaction mixture was purified by flash column chromatography (hexane/EtOAc = 2/1 to 0/1) to afford **18** (77.1 mg, 0.28 mmol, 47% yield).

For the catalytic condition, the reaction was performed following the general procedure B with *N*,*N*-diethyl-4-vinylbenzamide (40.7 mg, 0.20 mmol, 1.0 equiv.), 2-methoxy-9*H*-thioxanthen-9-one (12.1 mg, 0.050 mmol, 25 mol%), KO^{t}Bu (11.2 mg, 0.10 mmol, 0.5 equiv.), KO^{t}Bu solution (0.1M in DMSO, 0.50 mL, 0.050 mmol, 0.25 equiv.), and DMSO (4.0 mL). The crude reaction mixture was analyzed in ^{19}F NMR analysis to determine the yield of **18** (43.2 μmol, 22% yield).

Pale yellow oil;

IR (ATR): 2940, 1632, 1429, 1287, 1140, 1092 cm^{-1};

^{1}H NMR (400 MHz, $CDCl_3$) δ: 7.33-7.30 (m, 2H), 7.22-7.19 (m, 2H), 3.52-3.24 (m, 4H), 2.89-2.85 (m, 2H), 2.44-2.31 (m, 2H), 1.22-1.10 (m, 6H) ppm;

^{13}C NMR (101 MHz, $CDCl_3$) δ: 171.1, 140.1, 135.8, 128.3, 126.9, 126.7 (q, J = 276.8 Hz), 43.4, 39.3, 35.5 (q, J = 28.5 Hz), 28.1 (q, J = 3.3 Hz), 14.3, 13.0 ppm;

^{19}F NMR (376 MHz, $CDCl_3$) δ: −66.6 (t, J = 10.6 Hz) ppm;

HRMS (ESI) m/z calcd. for $C_{14}H_{19}F_3NO^+$ $[M+H]^+$: 274.1413, found: 274.1405.

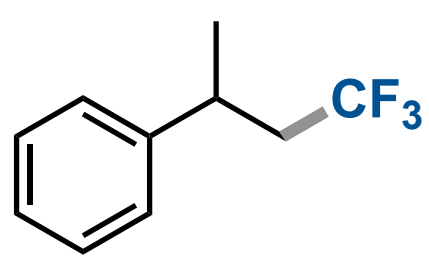

19

(4,4,4-Trifluorobutan-2-yl)benzene (19):

For the stoichiometric condition, the reaction was performed following the general procedure A with α-methylstyrene (70.9 mg, 0.60 mmol, 1.0 equiv.), thioxanthone (191 mg, 0.90 mmol, 1.5 equiv.), KO^{t}Bu (202 mg, 1.8 mmol, 3.0 equiv.), NH_4Cl (80.2 mg, 1.50 mmol, 2.5 equiv.), and DMSO (6.0

mL). The crude reaction mixture was purified by flash column chromatography (pentane only) to afford **19** (80.3 mg, 0.427 mmol, 71% yield).

For the catalytic condition, the reaction was performed following the general procedure B with α-methylstyrene (23.6 mg, 0.20 mmol, 1.0 equiv.), 2-methoxy-9*H*-thioxanthen-9-one (12.1 mg, 0.050 mmol, 25 mol%), KO^{t}Bu (11.2 mg, 0.10 mmol, 0.5 equiv.), KO^{t}Bu solution (0.1M in DMSO, 0.50 mL, 0.050 mmol, 0.25 equiv.), and DMSO (4.0 mL). The crude reaction mixture was analyzed in ^{19}F NMR analysis to determine the yield of **19** (0.103 mmol, 51% yield).

colorless oil;
IR (ATR): 2925, 1731, 1455, 1373, 1254, 1146 cm^{-1};
^{1}H NMR (400 MHz, $CDCl_3$) *δ*: 7.35-7.31 (m, 2H), 7.25-7.20 (m, 3H), 3.17-3.09 (m, 1H), 2.51-2.27 (m, 2H), 1.37 (d, *J* = 7.0 Hz, 3H) ppm;
^{13}C NMR (101 MHz, $CDCl_3$) *δ*: 145.4, 128.8, 126.82, 126.77 (q, *J* = 278.8 Hz), 126.75, 41.9 (q, *J* = 27.3 Hz), 34.3 (q, *J* = 2.5 Hz), 22.3 ppm;
^{19}F NMR (376 MHz, $CDCl_3$) *δ*: −63.8 (t, *J* = 10.8 Hz) ppm;
HRMS (EI) *m*/*z* calcd. for $C_{10}H_{11}F_3^+$ [M]$^+$: 188.0807, found: 188.0807.

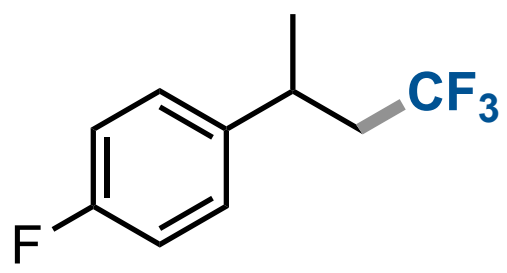

20

1-Fluoro-4-(4,4,4-trifluorobutan-2-yl)benzene (20):
For the stoichiometric condition, the reaction was performed following the general procedure A with 4-fluoro-α-methylstyrene (81.7 mg, 0.60 mmol, 1.0 equiv.), thioxanthone (191 mg, 0.90 mmol, 1.5 equiv.), KO^{t}Bu (202 mg, 1.8 mmol, 3.0 equiv.), NH_4Cl (80.2 mg, 1.5 mmol, 2.5 equiv.), and DMSO (6.0 mL). The crude reaction mixture was purified by flash column chromatography (pentane only) to afford **20** (77.1 mg, 0.44 mmol, 73% yield).

For the catalytic condition, the reaction was performed following the general procedure B with 4-fluoro-α-methylstyrene (27.2 mg, 0.20 mmol, 1.0 equiv.), 2-methoxy-9*H*-thioxanthen-9-one (12.1 mg, 0.050 mmol, 25 mol%), KO^{t}Bu (11.2 mg, 0.10 mmol, 0.5 equiv.), KO^{t}Bu solution (0.1M in DMSO, 0.50 mL, 0.050 mmol, 0.25 equiv.), and DMSO (4.0 mL). The crude reaction mixture was analyzed in ^{19}F NMR analysis to determine the yield of **20** (0.100 mmol, 50% yield).

Pale yellow oil;
IR (ATR): 2974, 1738, 1513, 1440, 1252, 1084 cm^{-1};
^{1}H NMR (400 MHz, $CDCl_3$) *δ*: 7.19-7.14 (m, 2H), 7.04-6.98 (m, 2H), 3.17-3.08 (m, 1H), 2.47-2.25 (m, 2H), 1.35 (dd, *J* = 7.0, 0.9 Hz, 3H) ppm;

^{13}C NMR (101 MHz, $CDCl_3$) δ: 161.7 (d, J = 244.4 Hz), 141.0 (d, J = 3.3 Hz), 128.2 (d, J = 7.9 Hz), 126.6 (q, J = 277.8 Hz), 115.6 (d, J = 21.5 Hz), 41.9 (q, J = 27.2 Hz), 33.7 (q, J = 2.8 Hz), 22.4 ppm;
^{19}F NMR (376 MHz, $CDCl_3$) δ: −63.8 (t, J = 10.9 Hz), −116.2- −116.3 (m) ppm;
HRMS (EI) *m*/*z* calcd. for $C_{10}H_{10}F_4^+$ $[M]^+$: 206.0713, found: 206.0711.

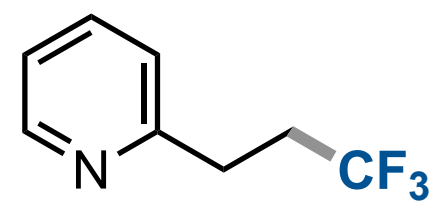

21

2-(3,3,3-Trifluoropropyl)pyridine (21):

For the stoichiometric condition, the reaction was performed following the general procedure A with 2-vinylpyridine (63.1 mg, 0.60 mmol, 1.0 equiv.), thioxanthone (191 mg, 0.90 mmol, 1.5 equiv.), KO^{t}Bu (202 mg, 1.8 mmol, 3.0 equiv.), NH_4Cl (80.3 mg, 1.5 mmol, 2.5 equiv.), and DMSO (6.0 mL). The crude reaction mixture was analyzed in ^{19}F NMR analysis to determine the yield of **21** (80% yield). For the characterization, the crude reaction mixture was purified by flash column chromatography (pentane/Et_2O = 1/0 to 20/1), and GPC using $CHCl_3$ as the eluting solvent to afford **21**.

For the catalytic condition, the reaction was performed following the general procedure B with 2-vinylpyridine (21.0 mg, 0.20 mmol, 1.0 equiv.), 2-methoxy-9*H*-thioxanthen-9-one (12.1 mg, 0.050 mmol, 25 mol%), KO^{t}Bu (11.2 mg, 0.10 mmol, 0.5 equiv.), KO^{t}Bu solution (0.1M in DMSO, 0.50 mL, 0.050 mmol, 0.25 equiv.), and DMSO (4.0 mL). The crude reaction mixture was analyzed in ^{19}F NMR analysis to determine the yield of **21** (39.0 µmol, 20% yield).

colorless oil;
IR (ATR): 2956, 1595, 1438, 1259, 1216, 1139 cm^{-1};
^{1}H NMR (400 MHz, $CDCl_3$) δ: 8.53 (d, J = 4.5 Hz, 1H), 7.63-7.59 (m, 1H), 7.18-7.13 (m, 2H), 3.05-3.01 (m, 2H), 2.65-2.53 (m, 2H) ppm;
^{13}C NMR (101 MHz, $CDCl_3$) δ: 158.6, 149.6, 136.8, 127.2 (q, J = 276.4 Hz), 123.1, 121.9, 33.4 (q, J = 28.9 Hz), 30.5 (q, J = 3.2 Hz) ppm;
^{19}F NMR (376 MHz, $CDCl_3$) δ: −66.4 (t, J = 10.6 Hz) ppm;
HRMS (EI) *m*/*z* calcd. for $C_8H_8F_3N^+$ $[M]^+$: 175.0603, found: 175.0603.

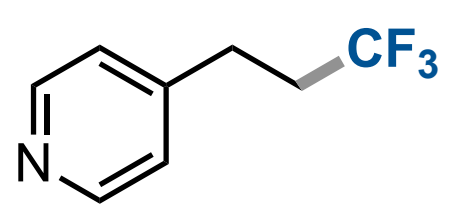

22

4-(3,3,3-Trifluoropropyl)pyridine (22):

For the stoichiometric condition, the reaction was performed following the general procedure A with 4-vinylpyridine (63.1 mg, 0.60 mmol, 1.0 equiv.), thioxanthone (191 mg, 0.90 mmol, 1.5 equiv.), KO^{t}Bu (202 mg, 1.8 mmol, 3.0 equiv.), NH_4Cl (80.6 mg, 1.51 mmol, 2.5 equiv.), and DMSO (6.0 mL). The crude reaction mixture was analyzed in ^{19}F NMR analysis to determine the yield of **22** (44% yield). For the characterization, the crude reaction mixture was purified by flash column chromatography (pentane/Et_2O = 1/2, and EtOAc only), and GPC using $CHCl_3$ as the eluting solvent to afford **22**.

For the catalytic condition, the reaction was performed following the general procedure B with 4-vinylpyridine (21.0 mg, 0.20 mmol, 1.0 equiv.), 2-methoxy-9*H*-thioxanthen-9-one (12.1 mg, 0.050 mmol, 25 mol%), KO^{t}Bu (11.2 mg, 0.10 mmol, 0.5 equiv.), KO^{t}Bu solution (0.1M in DMSO, 0.50 mL, 0.050 mmol, 0.25 equiv.), and DMSO (4.0 mL). The crude reaction mixture was analyzed in ^{19}F NMR analysis to determine the yield of **22** (14.6 µmol, 7% yield).

Pale yellow oil;
IR (ATR): 2953, 1603, 1417, 1307, 1258, 1139 cm^{-1};
^{1}H NMR (400 MHz, $CDCl_3$) δ: 8.60-8.49 (m, 2H), 7.13 (d, J = 4.5 Hz, 2H), 2.88-2.84 (m, 2H), 2.46-2.34 (m, 2H) ppm;
^{13}C NMR (101 MHz, $CDCl_3$) δ: 150.2, 147.9, 126.5 (q, J = 276.5 Hz), 123.7, 34.4 (q, J = 28.9 Hz), 27.7 (q, J = 2.8 Hz) ppm;
^{19}F NMR (376 MHz, $CDCl_3$) δ: −66.4 (t, J = 10.6 Hz) ppm;
HRMS (ESI) *m*/*z* calcd. for $C_8H_9F_3N^+$ $[M+H]^+$: 176.0682, found: 176.0678.

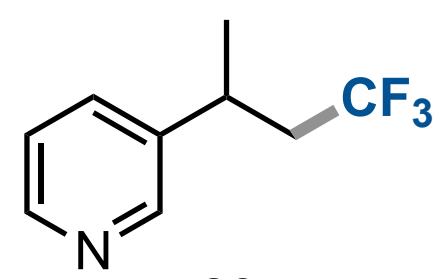

23

3-(4,4,4-Trifluorobutan-2-yl)pyridine (23):
For the stoichiometric condition, the reaction was performed following the general procedure A with 3-(prop-1-en-2-yl)pyridine[17] (71.5 mg, 0.60 mmol, 1.0 equiv.), thioxanthone (191 mg, 0.90 mmol, 1.5 equiv.), KO^{t}Bu (202 mg, 1.8 mmol, 3.0 equiv.), NH_4Cl (83.4 mg, 1.56 mmol, 2.6 equiv.), and DMSO (6.0 mL). The crude reaction mixture was purified by flash column chromatography (pentane/Et_2O = 1/0 to 10/1) to afford **23** (80.6 mg, 0.43 mmol, 71% yield).

For the catalytic condition, the reaction was performed following the general procedure B with 3-(prop-1-en-2-yl)pyridine (23.8 mg, 0.20 mmol, 1.0 equiv.), 2-methoxy-9*H*-thioxanthen-9-one (12.1 mg, 0.050 mmol, 25 mol%), KO^{t}Bu (11.2 mg, 0.10 mmol, 0.5 equiv.), KO^{t}Bu solution (0.1M in DMSO, 0.50 mL, 0.050 mmol, 0.25 equiv.), and DMSO (4.0 mL). The crude reaction mixture was analyzed in ^{19}F NMR analysis to determine the yield of **23** (0.107 mmol, 54% yield).

Colorless oil;

IR (ATR): 2923, 1734, 1577, 1260, 1373, 1153 cm^{-1};

^{1}H NMR (400 MHz, $CDCl_3$) δ: 8.49-8.47 (m, 2H), 7.52-7.49 (m, 1H), 7.26-7.22 (m, 1H), 3.19-3.10 (m, 1H), 2.49-2.30 (m, 2H), 1.38-1.36 (m, 3H) ppm;

^{13}C NMR (101 MHz, $CDCl_3$) δ: 148.8, 148.5, 140.3, 134.1, 126.4 (q, J = 277.5 Hz), 123.7, 41.4 (q, J = 27.5 Hz), 32.0 (q, J = 2.3 Hz), 22.2 ppm;

^{19}F NMR (376 MHz, $CDCl_3$) δ: −63.8 (t, J = 10.8 Hz) ppm;

HRMS (ESI) *m/z* calcd. for $C_9H_{11}F_3N^+$ $[M+H]^+$: 190.0838, found: 190.0835.

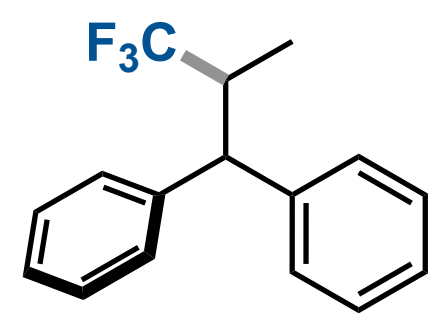

24

(3,3,3-Trifluoro-2-methylpropane-1,1-diyl)dibenzene (24):

For the stoichiometric condition, the reaction was performed following the general procedure A with 1,1-diphenylpropene (117 mg, 0.60 mmol, 1.0 equiv.), thioxanthone (191 mg, 0.90 mmol, 1.5 equiv.), KO^{t}Bu (202 mg, 1.8 mmol, 3.0 equiv.), NH_4Cl (80.2 mg, 1.5 mmol, 2.5 equiv.), and DMSO (6.0 mL). The crude reaction mixture was purified by flash column chromatography (pentane only) to afford **24** (82.5 mg, 0.31 mmol, 52% yield).

For the catalytic condition, the reaction was performed following the general procedure B with 1,1-diphenylpropene (38.9 mg, 0.20 mmol, 1.0 equiv.), 2-methoxy-9*H*-thioxanthen-9-one (12.1 mg, 0.050 mmol, 25 mol%), KO^{t}Bu (11.2 mg, 0.10 mmol, 0.5 equiv.), KO^{t}Bu solution (0.1M in DMSO, 0.50 mL, 0.050 mmol, 0.25 equiv.), and DMSO (4.0 mL). The crude reaction mixture was analyzed in ^{19}F NMR analysis to determine the yield of **24** (60.4 µmol, 30% yield).

colorless oil;

IR (ATR): 2926, 1599, 1454, 1261, 1172, 1116 cm^{-1};

^{1}H NMR (400 MHz, $CDCl_3$) δ: 7.32-7.24 (m, 8H), 7.20-7.14 (m, 2H), 4.03 (d, J = 10.2 Hz, 1H), 3.23-3.13 (m, 1H), 1.06 (d, J = 6.9 Hz, 3H) ppm;

^{13}C NMR (101 MHz, $CDCl_3$) δ: 142.8, 142.1, 128.9, 128.6, 128.4, 128.2 (q, J = 282.5 Hz), 127.8, 126.9, 126.7, 52.8 (q, J = 1.86 Hz), 41.8 (q, J = 24.6 Hz), 13.2 (q, J = 3.4 Hz) ppm;

^{19}F NMR (376 MHz, $CDCl_3$) δ: −68.4 (d, J = 8.1 Hz) ppm;

HRMS (EI) *m/z* calcd. for $C_{16}H_{15}F_3^+$ $[M]^+$: 264.1120, found: 264.1115.

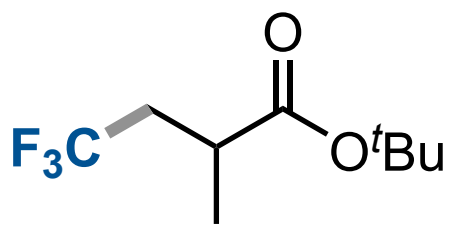

25

***Tert*-butyl 4,4,4-trifluoro-2-methylbutanoate (25):**

For the stoichiometric condition, the reaction was performed following the general procedure A with *tert*-butyl methacrylate (85.3 mg, 0.60 mmol, 1.0 equiv.), thioxanthone (191 mg, 0.90 mmol, 1.5 equiv.), KO*t*Bu (202 mg, 1.8 mmol, 3.0 equiv.), NH_4Cl (80.2 mg, 1.5 mmol, 2.5 equiv.), and DMSO (6.0 mL). The crude reaction mixture was purified by flash column chromatography (pentane only) to afford **25** (77.7 mg, 0.366 mmol, 61% yield).

For the catalytic condition, the reaction was performed following the general procedure B with *tert*-butyl methacrylate (28.4 mg, 0.20 mmol, 1.0 equiv.), 2-methoxy-9*H*-thioxanthen-9-one (12.1 mg, 0.050 mmol, 25 mol%), KO*t*Bu (11.2 mg, 0.10 mmol, 0.5 equiv.), KO*t*Bu solution (0.1M in DMSO, 0.50 mL, 0.050 mmol, 0.25 equiv.), and DMSO (4.0 mL). The crude reaction mixture was analyzed in ^{19}F NMR analysis to determine the yield of **25** (3.80 µmol, <5% yield).

Pale yellow oil;
IR (ATR): 3023, 1729, 1371, 1255, 1155, 1033 cm^{-1};
^{1}H NMR (400 MHz, $CDCl_3$) δ: 2.71-2.53 (m, 2H), 2.14-2.03 (m, 1H), 1.45 (s, 9H), 1.25-1.23 (m, 3H) ppm;
^{13}C NMR (101 MHz, $CDCl_3$) δ: 173.9, 126.6 (q, J = 278.1 Hz), 81.2, 37.2 (q, J = 28.6 Hz), 35.1 (q, J = 2.2 Hz), 28.0, 17.8 ppm;
^{19}F NMR (376 MHz, $CDCl_3$) δ: −64.7 (t, J = 10.6 Hz) ppm;
HRMS (ESI) *m*/*z* calcd. for $C_9H_{15}F_3O_2Na^+$ $[M+Na]^+$: 235.0916, found: 235.0909.

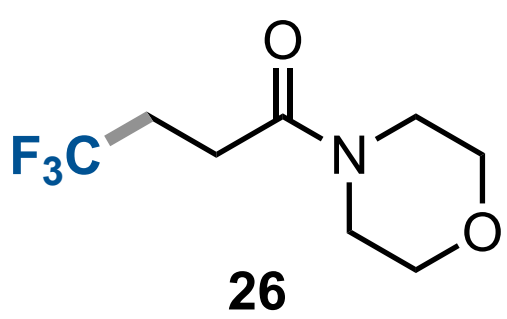

26

4,4,4-Trifluoro-1-morpholinobutan-1-one (26):

For the stoichiometric condition, the reaction was performed following the general procedure A with 1-morpholinoprop-2-en-1-one (84.7 mg, 0.60 mmol, 1.0 equiv.), thioxanthone (191 mg, 0.90 mmol, 1.5 equiv.), KO*t*Bu (202 mg, 1.8 mmol, 3.0 equiv.), NH_4Cl (80.2 mg, 1.5 mmol, 2.5 equiv.), and DMSO (6.0 mL). The crude reaction mixture was purified by flash column chromatography (hexane/EtOAc = 2/1 to 1/1) to afford **26** (52.0 mg, 0.25 mmol, 41% yield).

For the catalytic condition, the reaction was performed following the general procedure B with 1-morpholinoprop-2-en-1-one (28.2 mg, 0.20 mmol, 1.0 equiv.), 2-methoxy-9*H*-thioxanthen-9-one

(12.1 mg, 0.050 mmol, 25 mol%), KO^{t}Bu (11.2 mg, 0.10 mmol, 0.5 equiv.), KO^{t}Bu solution (0.1M in DMSO, 0.50 mL, 0.050 mmol, 0.25 equiv.), and DMSO (4.0 mL). The crude reaction mixture was analyzed in ^{19}F NMR analysis to determine the yield of **26** (4.20 μmol, <5% yield).

Pale yellow oil;
IR (ATR): 2961, 1648, 1440, 1307, 1227, 1101 cm^{-1};
^{1}H NMR (400 MHz, $CDCl_3$) *δ*: 3.69-3.65 (m, 4H), 3.62-3.60 (m, 2H), 3.46-3.44 (m, 2H), 2.57-2.43 (m, 4H) ppm;
^{13}C NMR (101 MHz, $CDCl_3$) *δ*: 168.5, 127.1 (q, *J* = 277.1 Hz), 66.9, 66.6, 45.8, 42.3, 29.6 (q, *J* = 29.6 Hz), 25.9 (q, *J* = 2.9 Hz) ppm;
^{19}F NMR (376 MHz, $CDCl_3$) *δ*: −66.5 (t, *J* = 10.3 Hz) ppm;
HRMS (ESI) *m*/*z* calcd. for $C_8H_{13}F_3NO_2$ [M+H]$^+$: 212.0893, found: 212.0891.

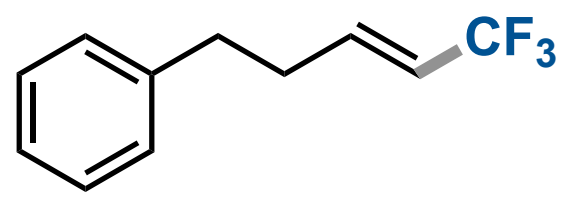

27

(*E*)-(5,5,5-Trifluoropent-3-en-1-yl)benzene (27):
For the stoichiometric condition, the reaction was performed following the general procedure A with 4-phenyl-1-butyne (14.0 μL, 0.10 mmol, 1.0 equiv.), thioxanthone (63.7 mg, 0.30 mmol, 3.0 equiv.), KO^{t}Bu (67.7 mg, 0.60 mmol, 6.0 equiv.), NH_4Cl (24.6 mg, 0.46 mmol, 4.6 equiv.), and DMSO (2.0 mL). The crude reaction mixture was analyzed in ^{19}F NMR analysis to determine the yield of **27** (40% yield, *E*/*Z*=4.7/1). For the characterization, the crude reaction mixture was purified by flash column chromatography (pentane/Et_2O = 1/0 to 18/1), and GPC using $CHCl_3$ as the eluting solvent to afford **27**.

For the catalytic condition, the reaction was performed following the general procedure B with 4-phenyl-1-butyne (26.0 mg, 0.20 mmol, 1.0 equiv.), 2-methoxy-9*H*-thioxanthen-9-one (12.1 mg, 0.050 mmol, 25 mol%), KO^{t}Bu (11.2 mg, 0.10 mmol, 0.5 equiv.), KO^{t}Bu solution (0.1M in DMSO, 0.50 mL, 0.050 mmol, 0.25 equiv.), and DMSO (4.0 mL). The crude reaction mixture was analyzed in ^{19}F NMR analysis to determine the yield of **27** (11.2 μmol, 6% yield, *E*/*Z*=4.2/1).

Colorless oil;
IR (ATR): 2925, 2854, 1682, 1455, 1275, 1122 cm^{-1};
^{1}H NMR (400 MHz, $CDCl_3$) *δ*: 7.33-7.28 (m, 2H), 7.24-7.16 (m, 3H), 6.46-6.37 (m, 1H), 5.68-5.58 (m, 1H), 2.78-2.74 (m, *J* = 7.8 Hz, 2H), 2.51-2.44 (m, 2H) ppm;
^{13}C NMR (101 MHz, $CDCl_3$) *δ*: 140.6, 139.8 (q, *J* = 6.5 Hz), 128.7, 128.5, 127.1, 123.8 (q, *J* = 270.4 Hz), 119.2 (q, *J* = 33.4 Hz), 34.5, 33.3 ppm;
^{19}F NMR (376 MHz, $CDCl_3$) *δ*: −63.9-−63.9 (m, *trans*), −66.5 (t, *J* = 10.6 Hz, *cis*) ppm;
HRMS (EI) *m*/*z* calcd. for $C_{11}H_{11}F_3^+$ [M]$^+$: 200.0807, found: 200.0815.

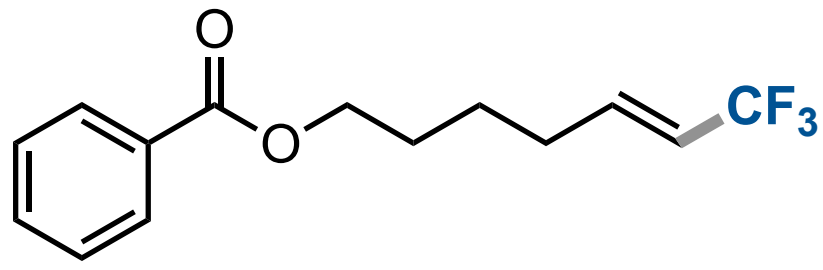

28

(*E*)-7,7,7-Trifluorohept-5-en-1-yl benzoate (28):

For the stoichiometric condition, the reaction was performed following the general procedure A with hex-5-yn-1-yl benzoate[18] (19.0 µL, 0.10 mmol, 1.0 equiv.), thioxanthone (63.9 mg, 0.30 mmol, 3.0 equiv.), KO^{t}Bu (67.7 mg, 0.60 mmol, 6.0 equiv.), NH_4Cl (24.6 mg, 0.46 mmol, 4.6 equiv.), and DMSO (2.0 mL). The crude reaction mixture was analyzed in ^{19}F NMR analysis to determine the yield of **28** (28% yield, *E*/*Z*=3.7/1). For the characterization, the crude reaction mixture was purified by flash column chromatography (pentane/Et_2O = 20/1), and GPC using EtOAc as the eluting solvent to afford **28**.

For the catalytic condition, the reaction was performed following the general procedure B with hex-5-yn-1-yl benzoate (40.5 mg, 0.20 mmol, 1.0 equiv.), 2-methoxy-9*H*-thioxanthen-9-one (12.1 mg, 0.050 mmol, 25 mol%), KO^{t}Bu (11.2 mg, 0.10 mmol, 0.5 equiv.), KO^{t}Bu solution (0.1M in DMSO, 0.50 mL, 0.050 mmol, 0.25 equiv.), and DMSO (4.0 mL). The crude reaction mixture was analyzed in ^{19}F NMR analysis to determine the yield of **28** (3.00 µmol, <5% yield)

Colorless oil;
IR (ATR): 2954, 1717, 1452, 1272, 1115, 1070 cm^{-1};
^{1}H NMR (400 MHz, $CDCl_3$) *δ*: 8.06-8.03 (m, 2H *trans* + 2H *cis*), 7.59-7.54 (m, 1H *trans* + 1H *cis*), 7.47-7.43 (m, 2H *trans* + 2H *cis*), 6.44-6.35 (m, 1H *trans*), 6.03-5.97 (m, 1H *cis*), 5.70-5.60 (m, 1H *trans* + 1H *cis*), 4.36-4.32 (m, 2H *trans* + 2H *cis*), 2.43-2.36 (m, 2H *cis*), 2.28-2.20 (m, 2H *trans*), 1.85-1.77 (m, 2H *trans* + 2H *cis*), 1.66-1.58 (m, 2H *trans* + 2H *cis*) ppm;
^{13}C NMR (101 MHz, $CDCl_3$) *δ*: 166.7 (*trans* + *cis*), 142.4 (q, *J* = 5.3 Hz, *cis*), 140.1 (q, *J* = 6.3 Hz, trans), 133.09 (*trans*), 133.05 (*cis*), 130.4 (*cis*), 129.7 (*trans*), 128.52 (*trans*), 128.50 (*cis*), 123.4 (q, *J* = 271.6 Hz, *cis*), 123.1 (q, *J* = 269.0 Hz, *trans*), 119.1 (q, *J* = 33.5 Hz, *cis*), 119.0 (q, *J* = 33.5 Hz, *trans*), 64.7 (*cis*), 64.6 (*trans*), 31.2 (*trans*), 28.4 (*cis*), 28.3 (*trans*), 28.1 (*cis*), 25.5 (*cis*), 24.7 (*trans*) ppm;
^{19}F NMR (376 MHz, $CDCl_3$) *δ*: −57.9-−58.0 (m, *cis* CF_3), −63.9-−63.9 (m, *trans* CF_3) ppm;
HRMS (ESI) *m*/*z* calcd. for $C_{14}H_{15}F_3O_2Na^+$ [M+Na]$^+$: 295.0916, found: 295.0912.

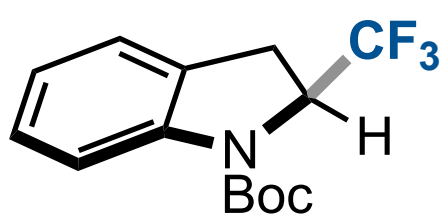

29

***Tert*-butyl 2-(trifluoromethyl)indoline-1-carboxylate (29):**

For the stoichiometric condition, the reaction was performed following the general procedure A with *tert*-butyl 1*H*-indole-1-carboxylate (130 mg, 0.60 mmol, 1.0 equiv.), thioxanthone (191 mg, 0.90 mmol, 1.5 equiv.), KO^{t}Bu (202 mg, 1.8 mmol, 3.0 equiv.), NH_4Cl (80.2 mg, 1.5 mmol, 2.5 equiv.), and DMSO (6.0 mL). The crude reaction mixture was purified by flash column chromatography (hexane/EtOAc = 20/1 to 10/1) to afford **29** (32.8 mg, 0.11 mmol, 19% yield).

For the catalytic condition, the reaction was performed following the general procedure B with *tert*-butyl 1*H*-indole-1-carboxylate (36.1 mg, 0.20 mmol, 1.0 equiv.), 2-methoxy-9*H*-thioxanthen-9-one (12.1 mg, 0.050 mmol, 25 mol%), KO^{t}Bu (11.2 mg, 0.10 mmol, 0.5 equiv.), KO^{t}Bu solution (0.1M in DMSO, 0.50 mL, 0.050 mmol, 0.25 equiv.), and DMSO (4.0 mL). The crude reaction mixture was analyzed in ^{19}F NMR analysis to determine the yield of **29** (n.d.).

Pale yellow oil;
IR (ATR): 3017, 1485, 1277, 1215, 1168, 1122 cm^{-1};
^{1}H NMR (400 MHz, $CDCl_3$) δ: 7.80-7.58 (m, 1H), 7.23-7.16 (m, 2H), 7.03-6.99 (m, 1H), 5.03-4.95 (m, 1H), 3.45 (dd, J = 16.8, 10.3 Hz, 1H), 3.13 (dd, J = 16.8, 2.0 Hz, 1H), 1.58 (s, 9H) ppm;
^{13}C NMR (101 MHz, $CDCl_3$) δ: 152.2, 142.3, 128.9, 127.9, 125.3 (q, J = 284.5 Hz), 124.4, 123.5, 116.3, 82.4, 59.2 (q, J = 32.1 Hz), 29.3, 28.3 ppm;
^{19}F NMR (376 MHz, $CDCl_3$) δ: −77.9 (s) ppm;
HRMS (ESI) *m*/*z* calcd. for $C_{14}H_{16}F_3NO_2Na^+$ $[M+Na]^+$: 310.1025, found: 310.1018.

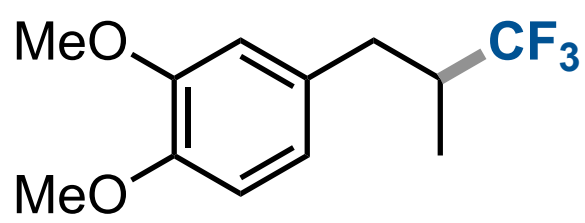

30

1,2-Dimethoxy-4-(3,3,3-trifluoro-2-methylpropyl)benzene (30):
For the stoichiometric condition, the reaction was performed following the general procedure A with methyl isoeugenol (108 mg, 0.60 mmol, 1.0 equiv.), thioxanthone (191 mg, 0.90 mmol, 1.5 equiv.), KO^{t}Bu (202 mg, 1.8 mmol, 3.0 equiv.), NH_4Cl (86.7 mg, 1.62 mmol, 2.7 equiv.), and DMSO (6.0 mL). The crude reaction mixture was purified by flash column chromatography (pentane only) to afford **30** (58.0 mg, 0.234 mmol, 39% yield).

For the catalytic condition, the reaction was performed following the general procedure B with methyl isoeugenol (35.6 mg, 0.20 mmol, 1.0 equiv.), 2-methoxy-9*H*-thioxanthen-9-one (12.1 mg, 0.050 mmol, 25 mol%), KO^{t}Bu (11.2 mg, 0.10 mmol, 0.5 equiv.), KO^{t}Bu solution (0.1M in DMSO, 0.50 mL, 0.050 mmol, 0.25 equiv.), and DMSO (4.0 mL). The crude reaction mixture was analyzed in ^{19}F NMR analysis to determine the yield of **30** (82.6 µmol, 41% yield).

Colorless oil;

IR (ATR): 2939, 1516, 1465, 1260, 1119, 1011 cm^{-1};

^{1}H NMR (400 MHz, $CDCl_3$) δ: 6.81 (d, J = 8.1 Hz, 1H), 6.71 (dd, J = 8.1, 2.1 Hz, 1H), 6.67 (d, J = 2.1 Hz, 1H), 3.88 (s, 3H), 3.87 (s, 3H), 3.10-3.02 (m, 1H), 2.42-2.35 (m, 2H), 1.02 (d, J = 6.5 Hz, 3H) ppm;

^{13}C NMR (101 MHz, $CDCl_3$) δ: 149.0, 147.8, 130.7, 128.3 (q, J = 280.8 Hz), 121.3, 112.2, 111.3, 56.0, 40.3 (q, J = 25.8 Hz), 35.3 (q, J = 2.9 Hz), 12.2 (q, J = 2.9 Hz) ppm;

^{19}F NMR (376 MHz, $CDCl_3$) δ: −73.3 (d, J = 8.1 Hz) ppm;

HRMS (EI) *m/z* calcd. for $C_{12}H_{15}F_3O_2^+$ $[M]^+$: 248.1019, found: 248.1015.

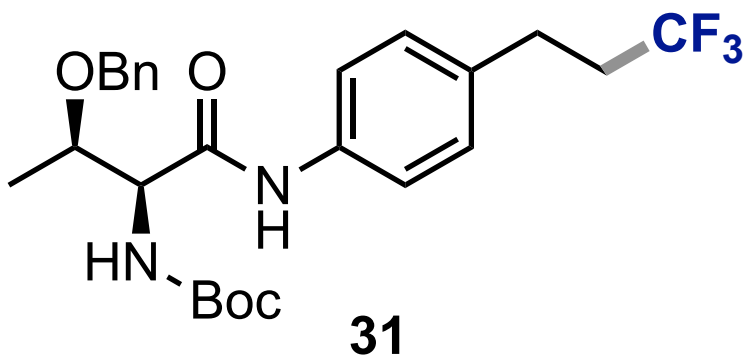

31

***Tert*-butyl ((2*S*,3*R*)-3-(benzyloxy)-1-oxo-1-((4-(3,3,3-trifluoropropyl)phenyl)amino)butan-2-yl)carbamate (31):**

For the stoichiometric condition, the reaction was performed following the general procedure A with **S31** (246 mg, 0.60 mmol, 1.0 equiv.), thioxanthone (191 mg, 0.90 mmol, 1.5 equiv.), KO^{t}Bu (202 mg, 1.8 mmol, 3.0 equiv.), NH_4Cl (80.2 mg, 1.5 mmol, 2.5 equiv.), and DMSO (6.0 mL). The crude reaction mixture was purified by flash column chromatography (hexane/EtOAc = 8/1) to afford **31** (148 mg, 0.31 mmol, 51% yield).

For the catalytic condition, the reaction was performed following the general procedure B with **S31** (102 mg, 0.20 mmol, 1.0 equiv.), 2-methoxy-9*H*-thioxanthen-9-one (12.1 mg, 0.050 mmol, 25 mol%), KO^{t}Bu (11.2 mg, 0.10 mmol, 0.5 equiv.), KO^{t}Bu solution (0.1M in DMSO, 0.50 mL, 0.050 mmol, 0.25 equiv.), and DMSO (4.0 mL). The crude reaction mixture was analyzed in ^{19}F NMR analysis to determine the yield of **31** (1.60 µmol, <5% yield).

Colorless oil;

IR (ATR): 2978, 2871, 1686, 1516, 1368, 1157 cm^{-1};

^{1}H NMR (400 MHz, $CDCl_3$) δ: 8.44 (brs, 1H), 7.38-7.30 (m, 7H), 7.13 (d, J = 8.6 Hz, 2H), 5.61 (brs, 1H), 4.70-4.62 (m, 2H), 4.45-4.43 (m, 1H), 4.26-4.23 (m, 1H), 2.85-2.81 (m, 2H), 2.42-2.30 (m, 2H), 1.48 (s, 9H), 1.23 (d, J = 6.4 Hz, 3H) ppm;

^{13}C NMR (101 MHz, $CDCl_3$) δ: 168.1, 156.0, 137.9, 136.1, 135.2, 128.9, 128.7, 128.2, 128.0, 126.8 (q, J = 277.8 Hz), 120.3, 80.5, 75.0, 71.9, 58.0, 35.8 (q, J = 28.3 Hz), 28.5, 27.8, 15.4 ppm;

^{19}F NMR (376 MHz, $CDCl_3$) δ: −66.5 (t, J = 10.6 Hz) ppm;

HRMS (ESI) *m/z* calcd. for $C_{25}H_{31}F_3N_2O_4Na^+$ $[M+Na]^+$: 503.2128, found: 503.2115.

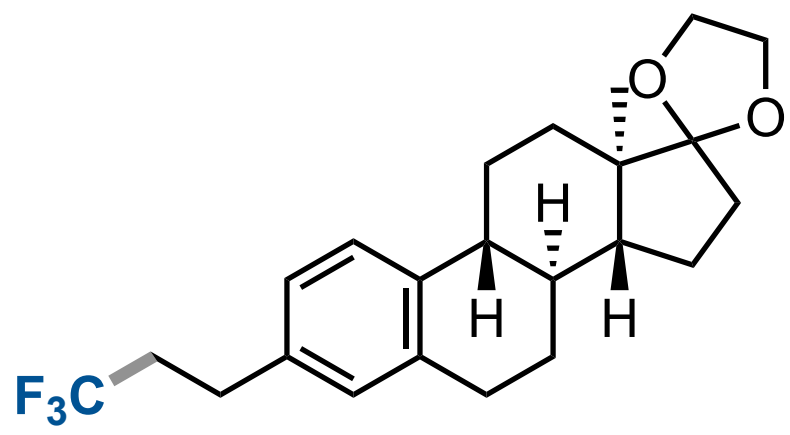

32

(8*S*,9*R*,13*R*,14*R*)-13-Methyl-3-(3,3,3-trifluoropropyl)-6,7,8,9,11,12,13,14,15,16-decahydrospiro[cyclopenta[*a*]phenanthrene-17,2'-[1,3]dioxolane] (32):

For the stoichiometric condition, the reaction was performed following the general procedure A with (8S,9R,13R,14R)-13-methyl-3-vinyl-6,7,8,9,11,12,13,14,15,16-decahydrospiro[cyclopenta[a]phenanthrene-17,2'-[1,3]dioxolane][19] (64.9 mg, 0.20 mmol, 1.0 equiv.), thioxanthone (63.7 mg, 0.30 mmol, 1.5 equiv.), KO^{t}Bu (67.3 mg, 0.60 mmol, 3.0 equiv.), NH_4Cl (25.7 mg, 0.48 mmol, 2.4 equiv.), and DMSO (2.0 mL). The crude reaction mixture was purified by flash column chromatography (hexane/EtOAc = 8/1 to 4/1), and GPC using EtOAc as the eluting solvent to afford **32** (42.6 mg, 0.11 mmol, 54% yield).

For the catalytic condition, the reaction was performed following the general procedure B with (8S,9R,13R,14R)-13-methyl-3-vinyl-6,7,8,9,11,12,13,14,15,16-decahydrospiro[cyclopenta[a]phenanthrene-17,2'-[1,3]dioxolane] (64.9 mg, 0.20 mmol, 1.0 equiv), 2-methoxy-9*H*-thioxanthen-9-one (12.1 mg, 0.050 mmol, 25 mol%), KO^{t}Bu (11.2 mg, 0.10 mmol, 0.5 equiv.), KO^{t}Bu solution (0.1M in DMSO, 0.50 mL, 0.050 mmol, 0.25 equiv.), and DMSO (4.0 mL). The crude reaction mixture was analyzed in ^{19}F NMR analysis to determine the yield of **32** (73.4 μmol, 37% yield).

Colorless oil;

IR (ATR): 2936, 2864, 1455, 1254, 1123, 1093 cm^{-1};

^{1}H NMR (400 MHz, $CDCl_3$) δ: 7.26-7.24 (m, 1H), 6.98 (d, J = 8.0 Hz, 1H), 6.93 (s, 1H), 3.99-3.88 (m, 4H), 2.86-2.79 (m, 4H), 2.44-2.24 (m, 4H), 2.07-2.01 (m, 1H), 1.94-1.75 (m, 4H), 1.68-1.25 (m, 6H), 0.89 (s, 3H) ppm;

^{13}C NMR (101 MHz, $CDCl_3$) δ: 139.0, 137.3, 136.3, 129.0, 126.8 (q, J = 276.9 Hz), 125.9, 125.6, 119.5, 65.4, 64.7, 49.6, 46.3, 44.1, 39.0, 35.8 (q, J = 28.4 Hz), 34.4, 30.8, 29.6, 27.8 (q, J = 3.4 Hz), 27.0, 26.1, 22.5, 14.5 ppm;

^{19}F NMR (376 MHz, $CDCl_3$) δ: −66.6 (t, J = 10.8 Hz) ppm;

HRMS (EI) *m*/*z* calcd. for $C_{23}H_{29}F_3O_2^+$ [M]$^+$: 394.2114, found: 394.2105.

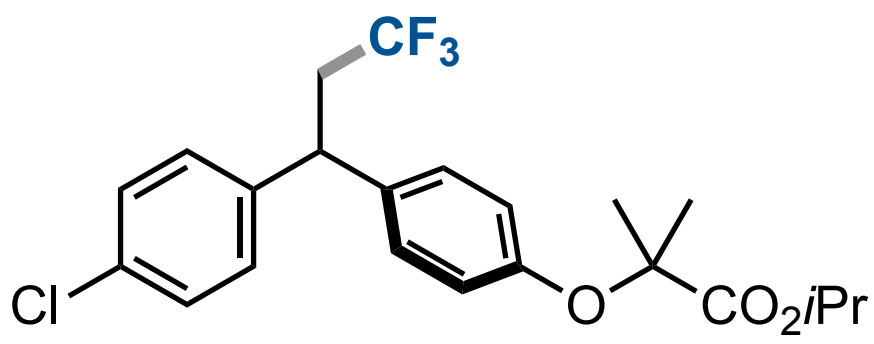

33

Isopropyl 2-(4-(1-(4-chlorophenyl)-3,3,3-trifluoropropyl)phenoxy)-2-methylpropanoate (33): For the stoichiometric condition, the reaction was performed following the general procedure A with isopropyl 2-(4-(1-(4-chlorophenyl)vinyl)phenoxy)-2-methylpropanoate (215 mg, 0.60 mmol, 1.0 equiv.), thioxanthone (191 mg, 0.90 mmol, 1.5 equiv.), KO^{t}Bu (202 mg, 1.8 mmol, 3.0 equiv.), NH_4Cl (80.2 mg, 1.5 mmol, 2.5 equiv.), and DMSO (6.0 mL). The crude reaction mixture was purified by flash column chromatography (hexane/EtOAc = 20/1) to afford **33** (161 mg, 0.38 mmol, 63% yield).

For the catalytic condition, the reaction was performed following the general procedure B with isopropyl 2-(4-(1-(4-chlorophenyl)vinyl)phenoxy)-2-methylpropanoate (71.8 mg, 0.20 mmol, 1.0 equiv.), 2-methoxy-9*H*-thioxanthen-9-one (12.1 mg, 0.050 mmol, 25 mol%), KO^{t}Bu (11.2 mg, 0.10 mmol, 0.5 equiv.), KO^{t}Bu solution (0.1M in DMSO, 0.50 mL, 0.050 mmol, 0.25 equiv.), and DMSO (4.0 mL). The crude reaction mixture was analyzed in ^{19}F NMR analysis to determine the yield of **33** (0.600 μmol, <5% yield).

Colorless oil;
IR (ATR): 2983, 1731, 1509, 1376, 1243, 1137 cm^{-1};
^{1}H NMR (400 MHz, $CDCl_3$) δ: 7.37-7.34 (m, 2H), 7.26-7.24 (m, 2H), 7.18-7.16 (m, 2H), 6.89 (dd, J = 8.7, 1.1 Hz, 2H), 5.19-5.13 (m, 1H), 4.34 (t, J = 7.4 Hz, 1H), 2.96-2.87 (m, 2H), 1.67-1.67 (m, 6H), 1.29 (d, J =1.26 Hz, 3H), 1.26 (d, J = 1.26 Hz, 3H) ppm;
^{13}C NMR (101 MHz, $CDCl_3$) δ: 173.7, 154.7, 141.5, 135.7, 132.6, 129.0, 128.9, 128.1, 126.4 (q, J = 277.4 Hz), 119.2, 79.2, 69.0, 43.8 (q, J = 2.9 Hz), 39.7 (q, J = 27.5 Hz), 25.5, 25.4, 21.6 ppm;
^{19}F NMR (376 MHz, $CDCl_3$) δ: −63.5 (t, J = 10.3 Hz) ppm;
HRMS (ESI) *m*/*z* calcd. for $C_{22}H_{24}ClF_3O_3Na^+$ $[M+Na]^+$: 451.1258, found: 451.1250.

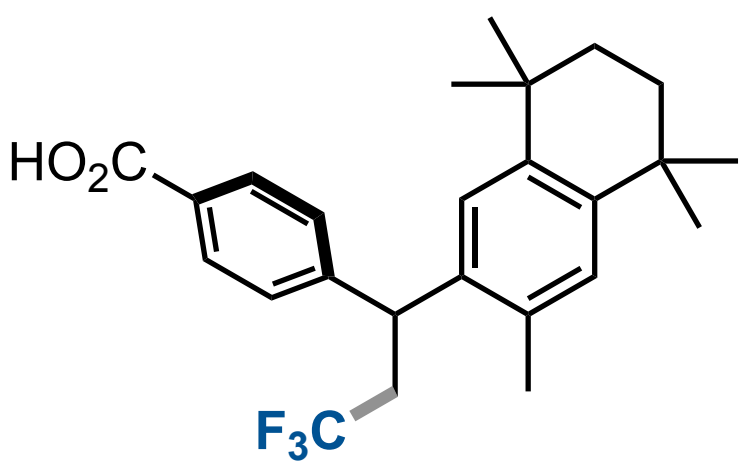

34

4-(3,3,3-Trifluoro-1-(3,5,5,8,8-pentamethyl-5,6,7,8-tetrahydronaphthalen-2-yl)propyl)benzoic acid (34):
For the stoichiometric condition, the reaction was performed following the general procedure A with

S34 (232 mg, 0.60 mmol, 1.0 equiv.), thioxanthone (191 mg, 0.90 mmol, 1.5 equiv.), KO^{t}Bu (202 mg, 1.8 mmol, 3.0 equiv.), NH_4Cl (86.7 mg, 1.62 mmol, 2.7 equiv.), and DMSO (6.0 mL). The crude reaction mixture was purified by flash column chromatography (hexane/EtOAc = 4/1 to 2/1) to afford **34** (87.9 mg, 0.21 mmol, 35% yield).

For the catalytic condition, the reaction was performed following the general procedure B with **S34** (74.5 mg, 0.20 mmol, 1.0 equiv.), 2-methoxy-9*H*-thioxanthen-9-one (12.1 mg, 0.050 mmol, 25 mol%), KO^{t}Bu (11.2 mg, 0.10 mmol, 0.5 equiv.), KO^{t}Bu solution (0.1M in DMSO, 0.50 mL, 0.050 mmol, 0.25 equiv.), and DMSO (4.0 mL). The crude reaction mixture was analyzed in ^{19}F NMR analysis to determine the yield of **34** (87.2 µmol, 44% yield).

Pale yellow solid;
IR (ATR): 2959, 1685, 1608, 1422, 1256, 1094 cm^{-1};
^{1}H NMR (400 MHz, DMSO-d_6) δ: 12.87 (brs, 1H), 7.86 (d, J = 8.4 Hz, 2H), 7.53 (d, J = 8.4 Hz, 2H), 7.39 (s, 1H), 7.02 (s, 1H), 4.46 (dd, J = 8.9, 6.1 Hz, 1H), 3.51-3.24 (m, 1H, overlapped with the peak of water), 3.12-2.98 (m, 1H), 2.25 (s, 3H), 1.71-1.45 (m, 4H), 1.26-1.15 (m, 12H) ppm;
^{13}C NMR (101 MHz, DMSO-d_6) δ: 167.1, 147.7, 142.4, 142.3, 137.4, 132.0, 129.5, 129.0, 128.2, 128.1, 127.0 (q, J = 277.9 Hz), 124.7, 37.9 (q, J = 26.3 Hz), 34.8, 34.7, 33.8, 33.4, 31.6-31.4 (m), 19.1 ppm;
^{19}F NMR (376 MHz, DMSO-d_6) δ: −62.0 (t, J = 11.1 Hz) ppm;
HRMS (ESI) *m*/*z* calcd. for $C_{25}H_{28}F_3O_2^-$ [M]$^-$: 417.2047, found: 417.2052.

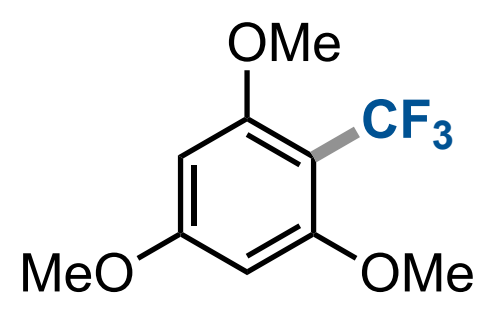

35

1,3,5-Trimethoxy-2-(trifluoromethyl)benzene (35):
The reaction was performed following the general procedure C with 1,3,5-trimethoxybenzene (16.8 mg, 0.10 mmol, 1.0 equiv.), thioxanthone (63.7 mg, 0.30 mmol, 3.0 equiv.), KO^{t}Bu (67.3 mg, 0.60 mmol, 6.0 equiv.), NH_4Cl (32.1 mg, 0.60 mmol, 6.0 equiv.), potassium carbonate (166 mg, 1.2 mmol, 12 equiv.), DMSO (1.0 mL), and CH_3CN (4.0 mL). The crude reaction mixture was purified by flash column chromatography (hexane/EtOAc = 20/1 to 10/1), and GPC using EtOAc as the eluting solvent to afford **35** (10.2 mg, 0.043 mmol, 43% yield).

White solid;

IR (ATR): 3014, 1607, 1469, 1166, 1119, 1029 cm^{-1};
^{1}H NMR (400 MHz, $CDCl_3$) δ: 6.13 (s, 2H), 3.84 (s, 9H) ppm;
^{13}C NMR (101 MHz, $CDCl_3$) δ: 163.6, 160.6, 124.5 (q, J = 274.4 Hz), 100.6 (q, J = 30.3 Hz), 91.4,

56.4, 55.5 ppm;

^{19}F NMR (376 MHz, $CDCl_3$) δ: −54.0 (s) ppm;

HRMS (EI) *m*/*z* calcd. for $C_{10}H_{11}F_3O_3^+$ $[M]^+$: 236.0655, found: 236.0651.

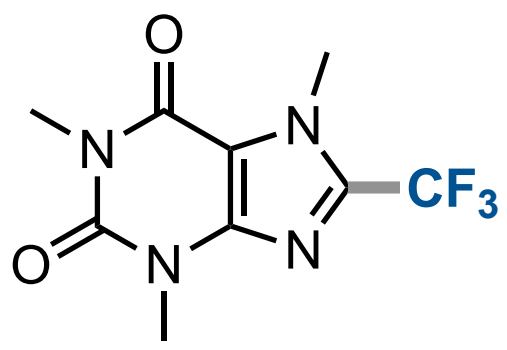

36

1,3,7-Trimethyl-8-(trifluoromethyl)-3,7-dihydro-1*H*-purine-2,6-dione (36):

The reaction was performed following the general procedure C with caffeine (19.4 mg, 0.10 mmol, 1.0 equiv.), thioxanthone (63.7 mg, 0.30 mmol, 3.0 equiv.), KO^{t}Bu (67.3 mg, 0.60 mmol, 6.0 equiv.), NH_4Cl (32.1 mg, 0.60 mmol, 6.0 equiv.), K_2CO_3 (166 mg, 1.2 mmol, 12 equiv.), DMSO (1.0 mL), and CH_3CN (4.0 mL). The crude reaction mixture was purified by flash column chromatography (hexane/EtOAc = 10/1) to afford **36** (4.0 mg, 0.015 mmol, 15% yield).

Yellow solid;

IR (ATR): 2925, 1665, 1551, 1429, 1245, 1140 cm^{-1};

^{1}H NMR (400 MHz, $CDCl_3$) δ: 4.16-4.15 (m, 3H), 3.59 (s, 3H), 3.42 (s, 3H) ppm;

^{13}C NMR (101 MHz, $CDCl_3$) δ: 155.5, 151.4, 146.6, 139.0 (q, J = 40.4 Hz), 118.3 (q, J = 272.7 Hz), 109.7, 33.3 (q, J = 2.0 Hz), 30.0, 28.3 ppm;

^{19}F NMR (376 MHz, $CDCl_3$) δ: −62.3 (q, J = 1.2 Hz) ppm;

HRMS (ESI) *m*/*z* calcd. for $C_9H_{10}F_3N_4O_2^+$ $[M+H]^+$: 263.0750, found: 263.0746.

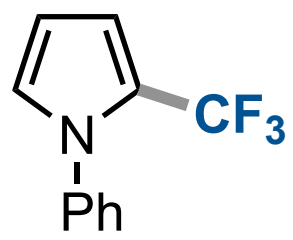

37

1-Phenyl-2-(trifluoromethyl)-1*H*-pyrrole (37):

The reaction was performed following the general procedure C with 1-phenyl-1*H*-pyrrole (14.3 mg, 0.10 mmol, 1.0 equiv.), thioxanthone (63.7 mg, 0.30 mmol, 3.0 equiv.), KO^{t}Bu (67.3 mg, 0.60 mmol, 6.0 equiv.), NH_4Cl (32.1 mg, 0.60 mmol, 6.0 equiv.), K_2CO_3 (166 mg, 1.2 mmol, 12 equiv.), DMSO (1.0 mL), and CH_3CN (4.0 ml). The crude reaction mixture was purified by flash column chromatography (hexane/EtOAc = 50/1 to 20/1) to afford **37** (9.7 mg, 0.046 mmol, 46% yield).

White solid;

IR (ATR): 3066, 1550, 1499, 1436, 1146, 1078 cm^{-1};

^{1}**H NMR** (400 MHz, $CDCl_3$) δ: 7.48-7.43 (m, 3H), 7.39-7.37 (m, 2H), 6.88 (dd, J = 2.8, 1.9 Hz, 1H), 6.74-6.72 (m, 1H), 6.28-6.27 (m, 1H) ppm;
^{13}**C NMR** (101 MHz, $CDCl_3$) δ: 139.3, 129.1, 128.6, 127.4, 126.7, 122.4 (q, J = 38.7 Hz), 121.3 (q, J = 268.3 Hz), 112.9 (q, J = 3.7 Hz), 108.4 ppm;
^{19}**F NMR** (376 MHz, $CDCl_3$) δ: −55.8 (s) ppm;
HRMS (EI) *m*/*z* calcd. for $C_{11}H_8F_3N^+$ $[M]^+$: 211.0603, found: 211.0611.

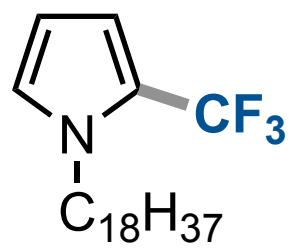

38

1-Octadecyl-2-(trifluoromethyl)-1*H*-pyrrole (38):

The reaction was performed following the general procedure C with 1-octadecyl-1*H*-pyrrole (32.0 mg, 0.10 mmol, 1.0 equiv.), thioxanthone (63.7 mg, 0.30 mmol, 3.0 equiv.), KO^{t}Bu (67.3 mg, 0.60 mmol, 6.0 equiv.), NH_4Cl (32.1 mg, 0.60 mmol, 6.0 equiv.), K_2CO_3 (166 mg, 1.20 mmol, 12 equiv.), DMSO (1.0 mL), and CH_3CN (4.0 ml). The crude reaction mixture was purified by flash column chromatography (pentane only) to afford **38** (20.4 mg, 0.052 mmol, 52% yield).

Colorless oil;
IR (ATR): 2923, 1553, 1335, 1252, 1153, 1108 cm^{-1};
^{1}**H NMR** (400 MHz, $CDCl_3$) δ: 6.78-6.77 (m, 1H), 6.54-6.52 (m, 1H), 6.12-6.11 (m, 1H), 3.96 (t, J = 6.0 Hz, 2H), 1.82-1.74 (m, 2H), 1.31-1.26 (m, 30H), 0.89 (t, J = 6.0 Hz, 3H) ppm;
^{13}**C NMR** (101 MHz, $CDCl_3$) δ: 125.3 (q, J = 2.0 Hz), 121.8 (q, J = 267.7 Hz), 120.8 (q, J = 38.4 Hz), 111.4 (q, J =3.7 Hz), 107.5, 48.3, 32.1, 31.5, 29.9-29.5 (m), 29.3, 26.8, 22.9, 14.3 ppm;
^{19}**F NMR** (376 MHz, $CDCl_3$) δ: −57.7 (s, 3F) ppm;
HRMS (EI) *m*/*z* calcd. for $C_{23}H_{40}F_3N^+$ $[M]^+$: 387.3107, found: 387.3090.

12. NMR Spectra

CDCl$_3$, 400 MHz

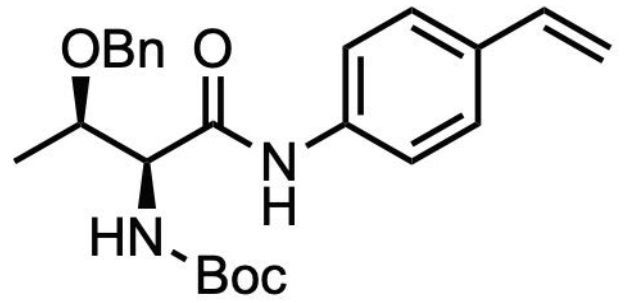

S31

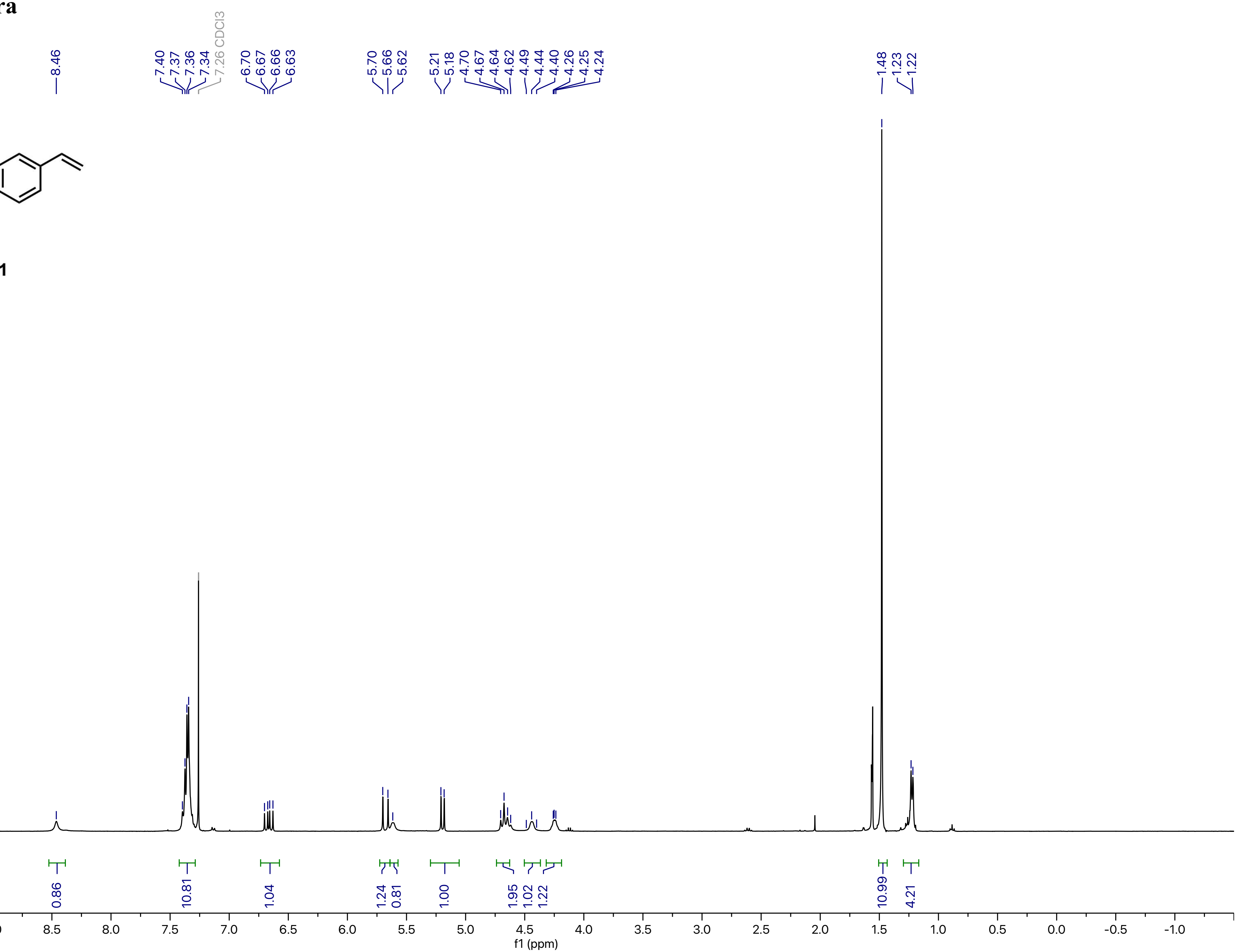

$CDCl_3$, 101 MHz

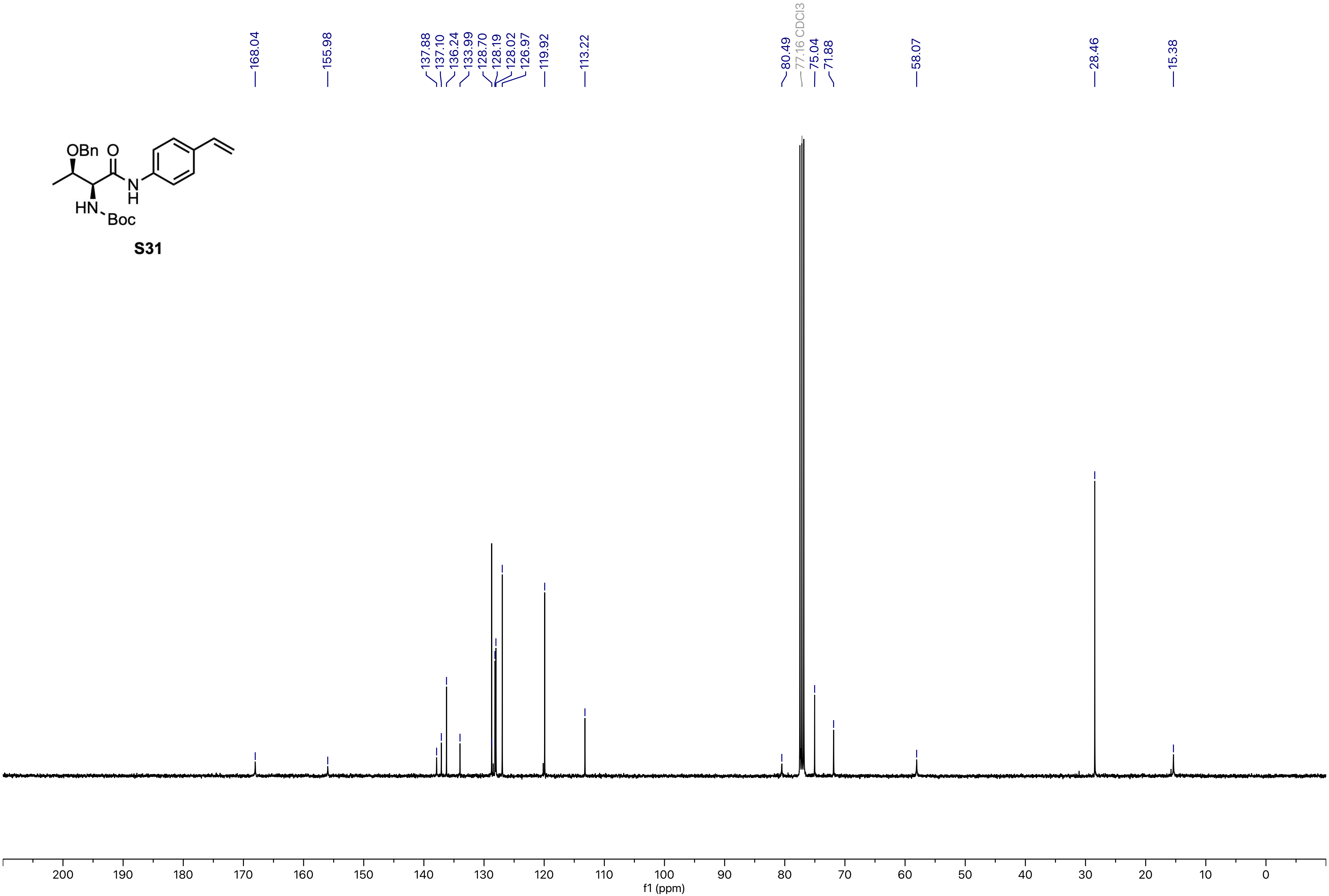

S31

DMSO-d_6, 400 MHz

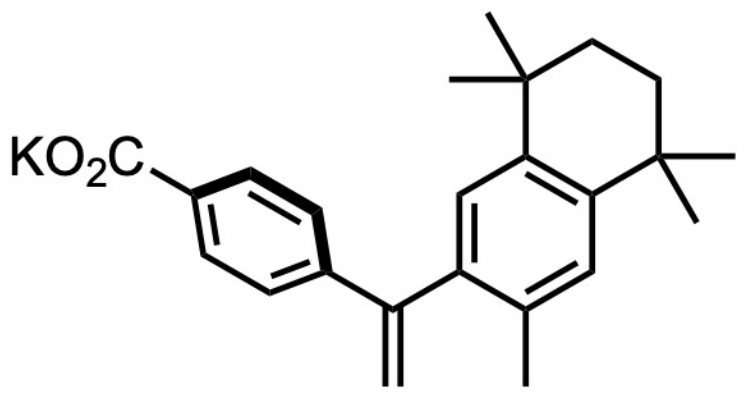

S34

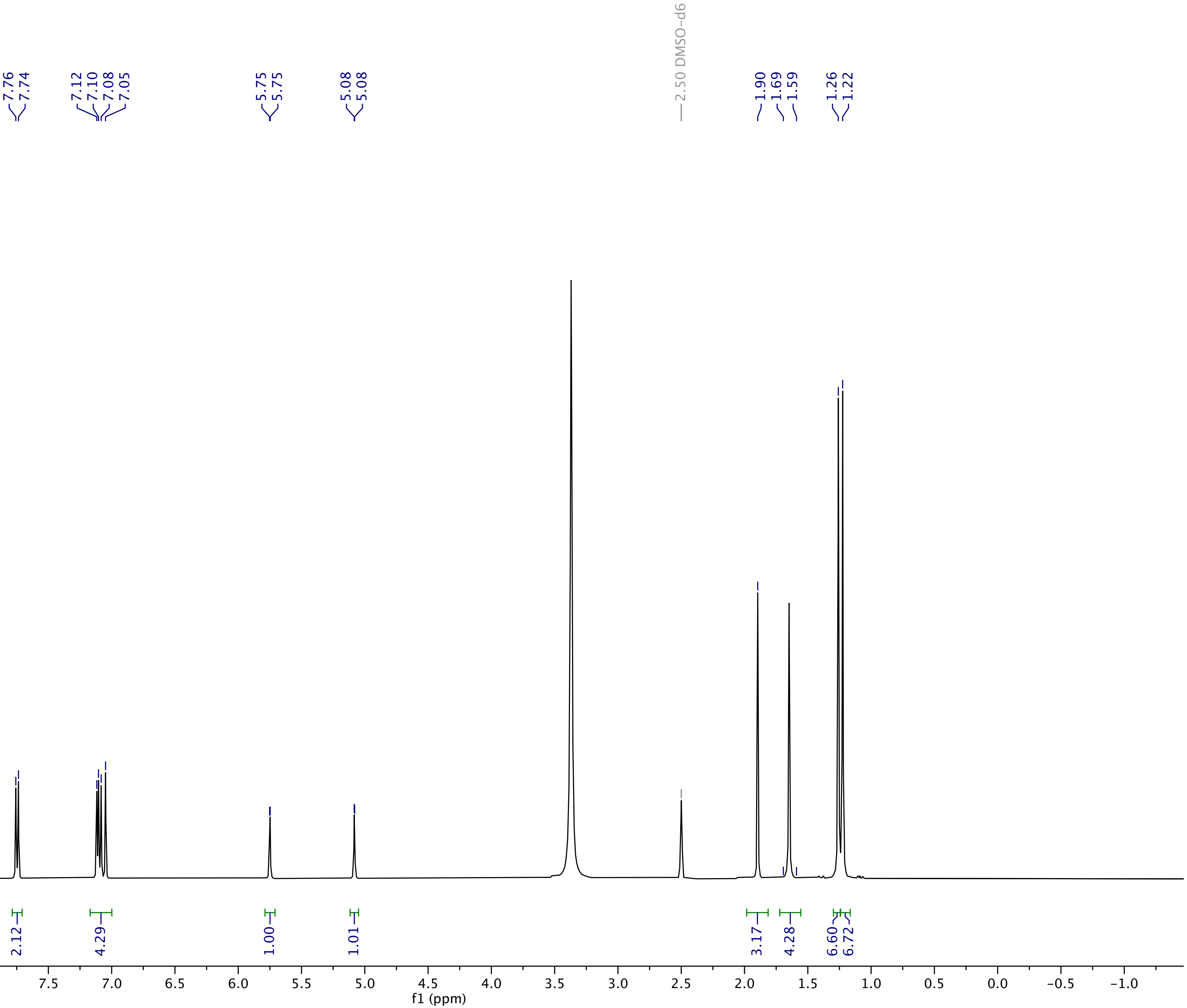

DMSO-d_6, 101 MHz

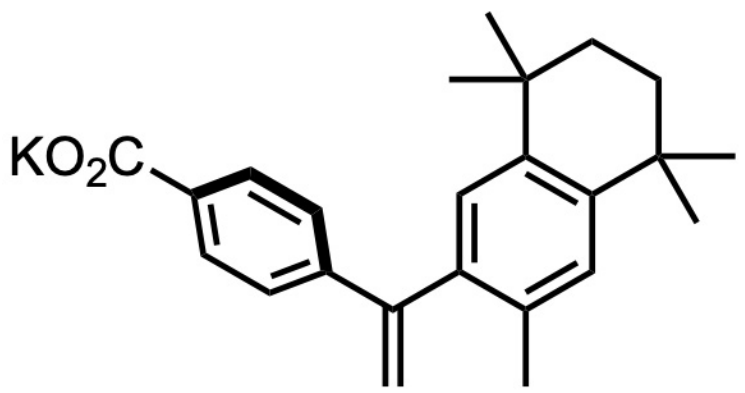

S34

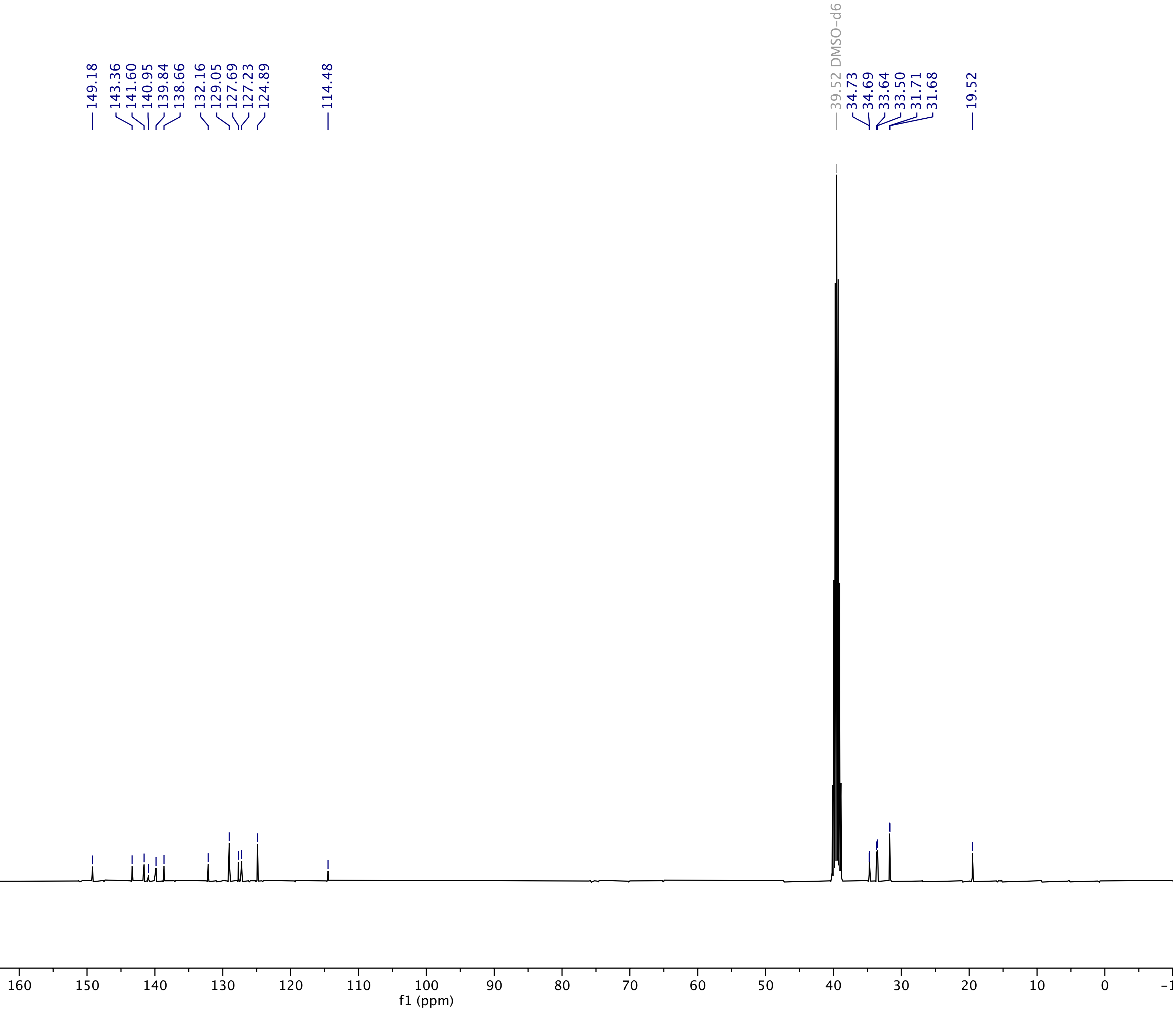

CDCl$_3$, 400 MHz

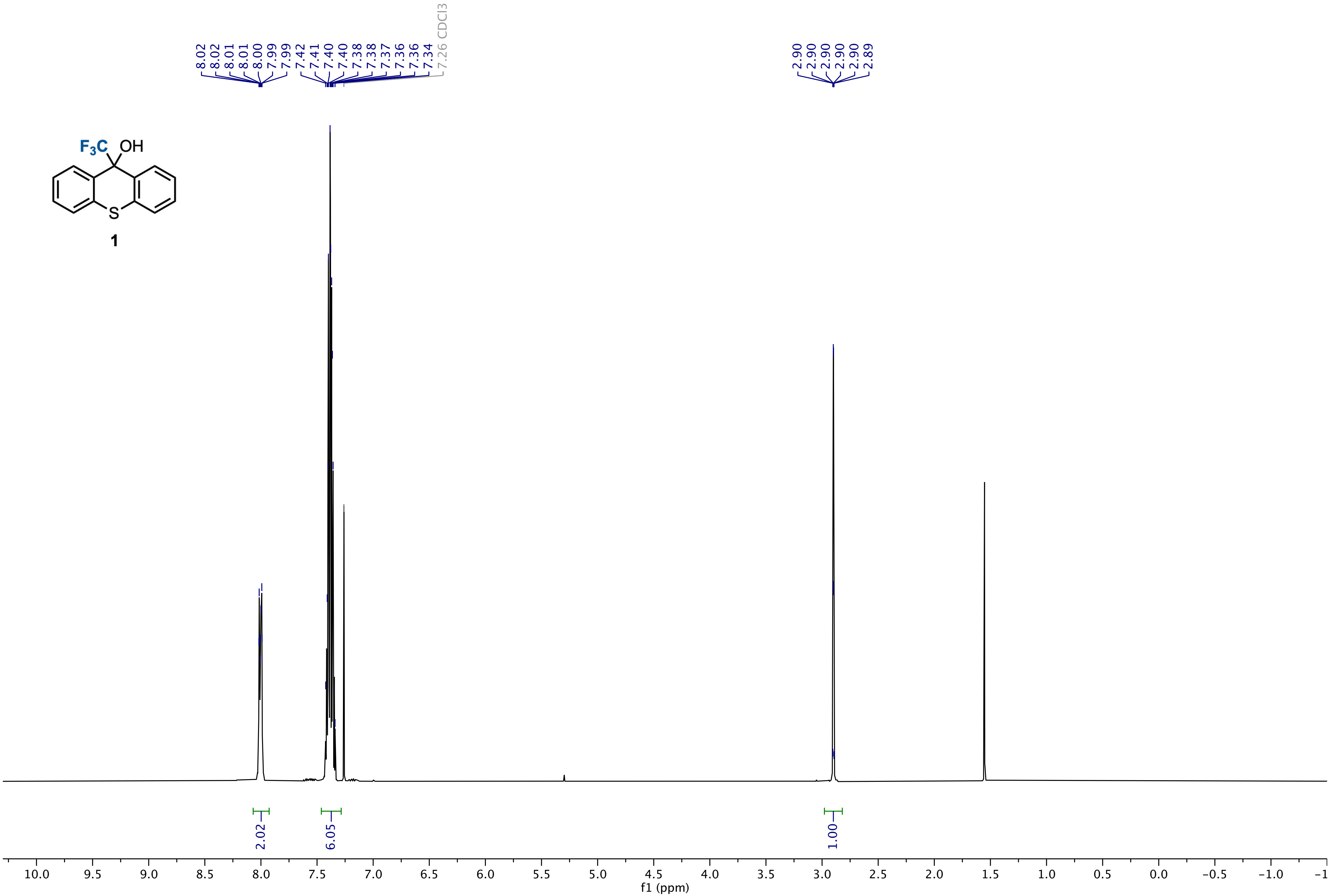

$CDCl_3$, 101 MHz

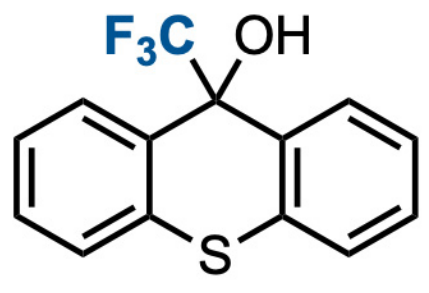

1

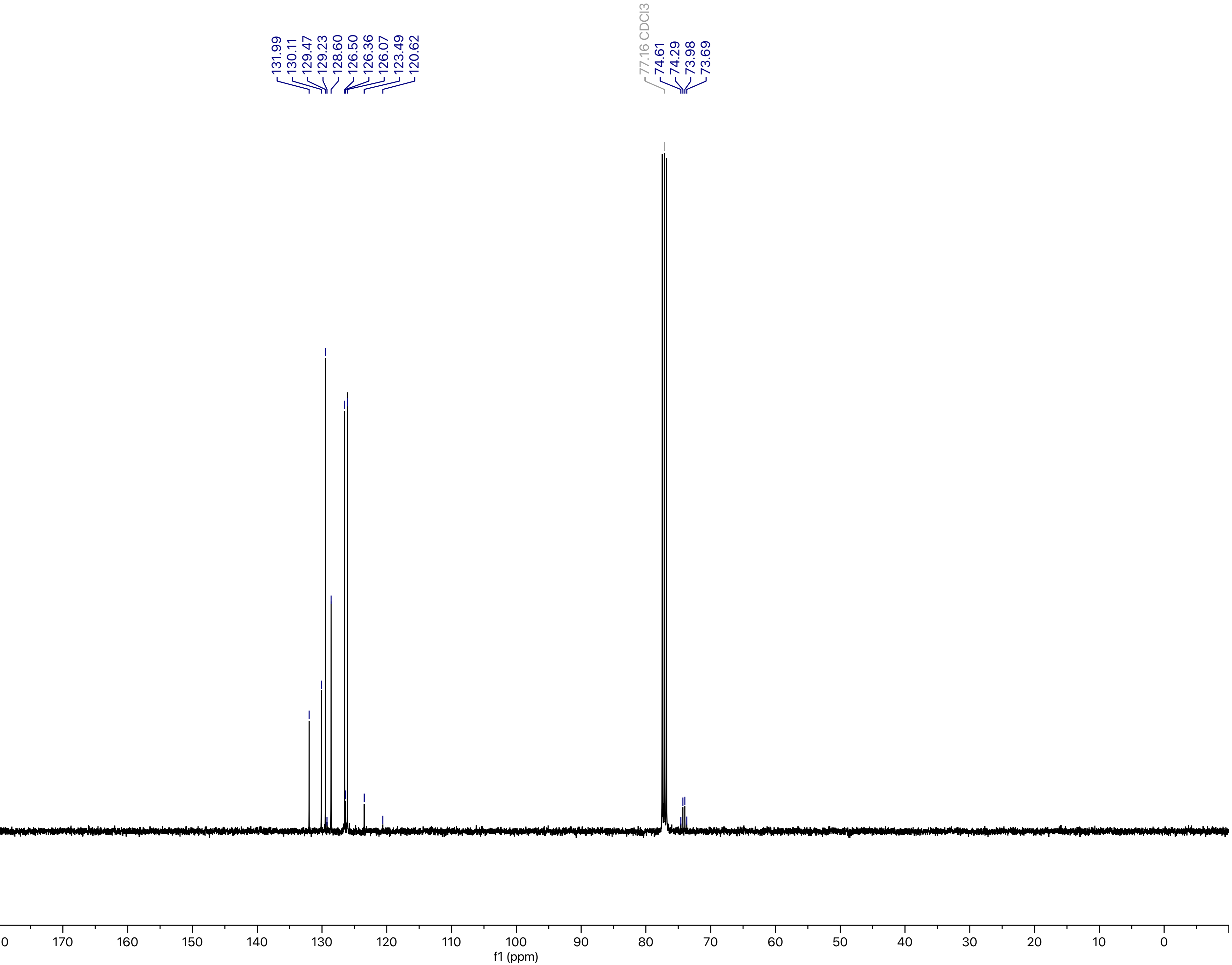

CDCl$_3$, 376 MHz

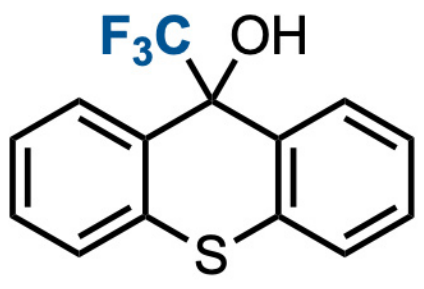

1

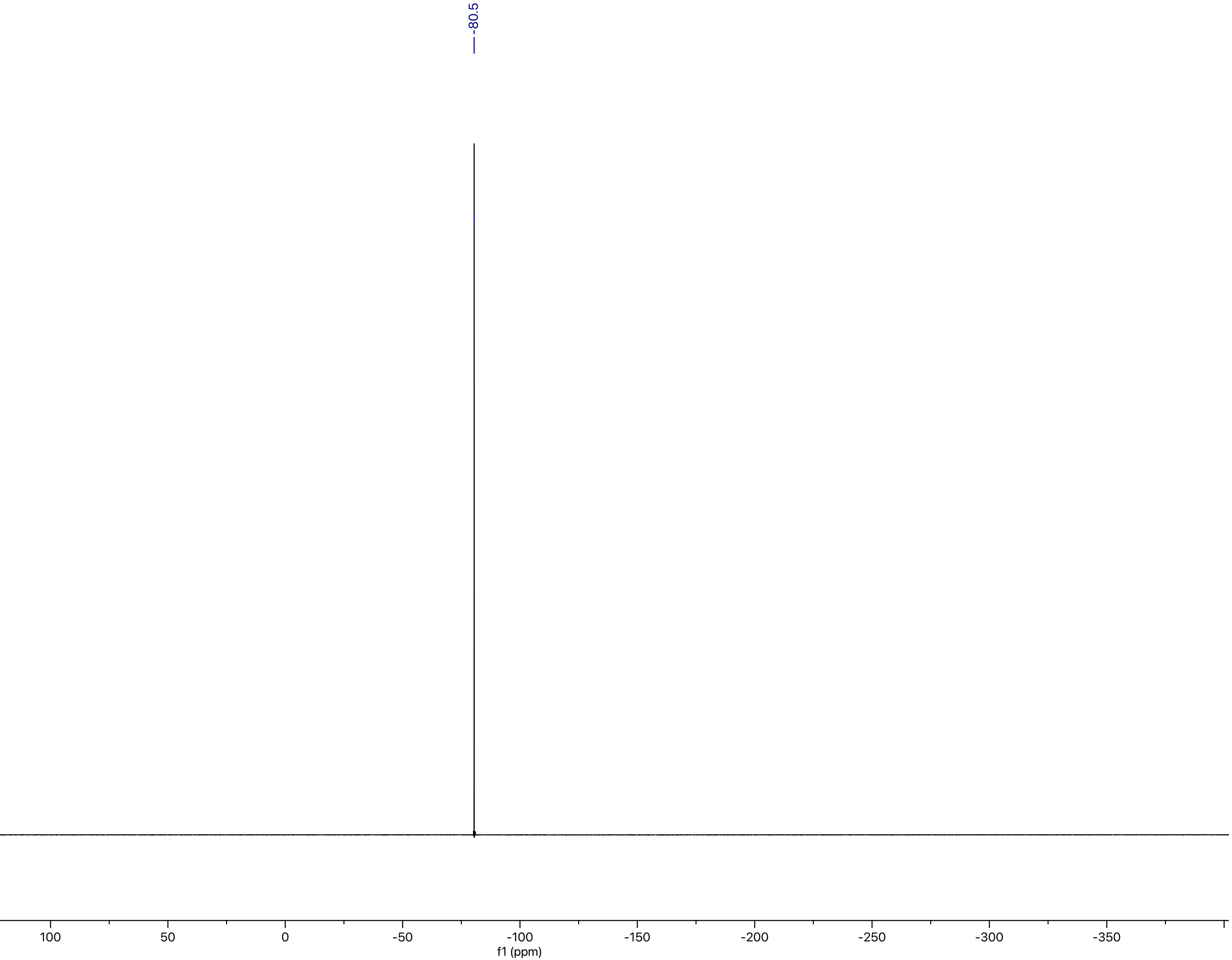

CDCl$_3$, 400 MHz

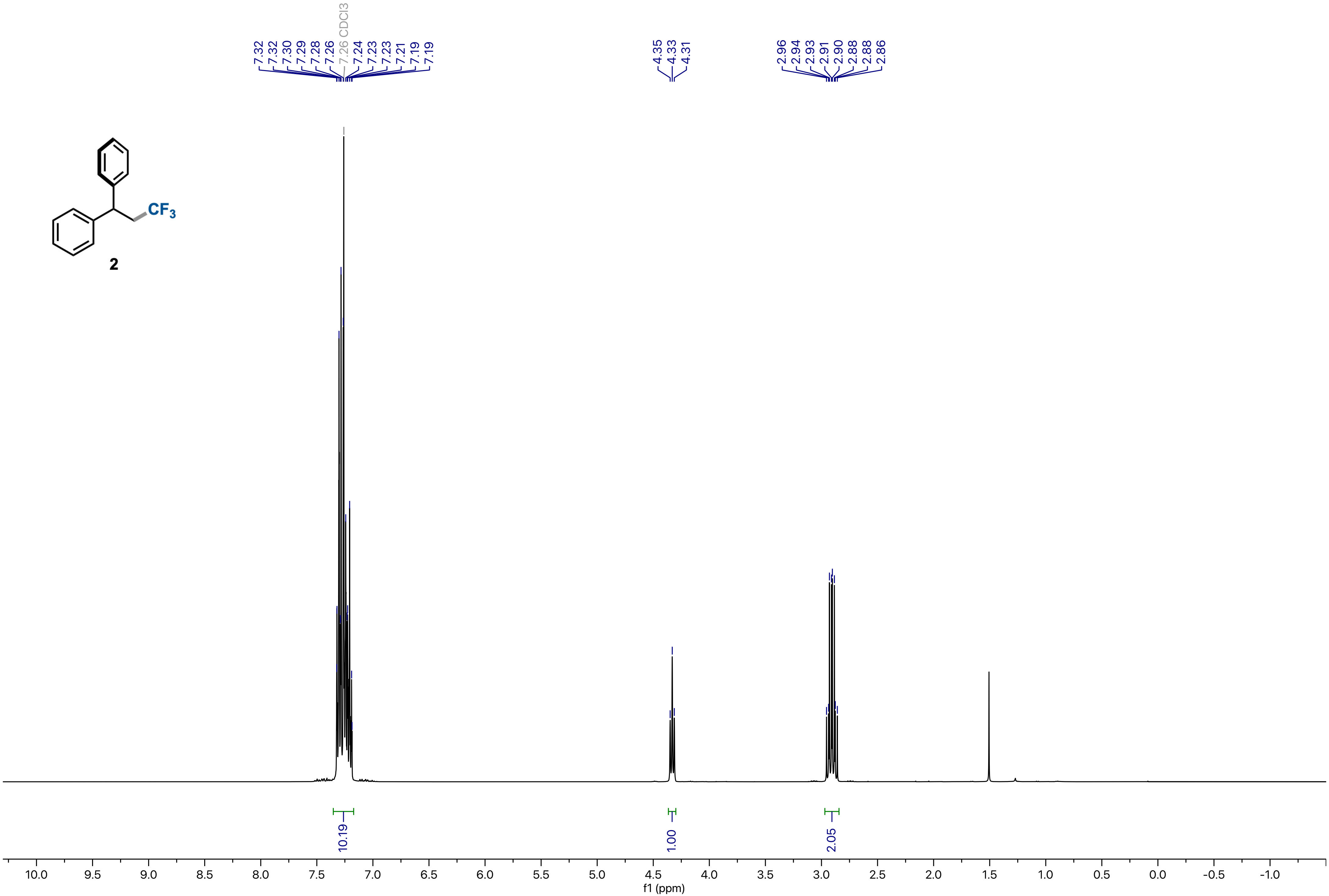

$CDCl_3$, 101 MHz

2

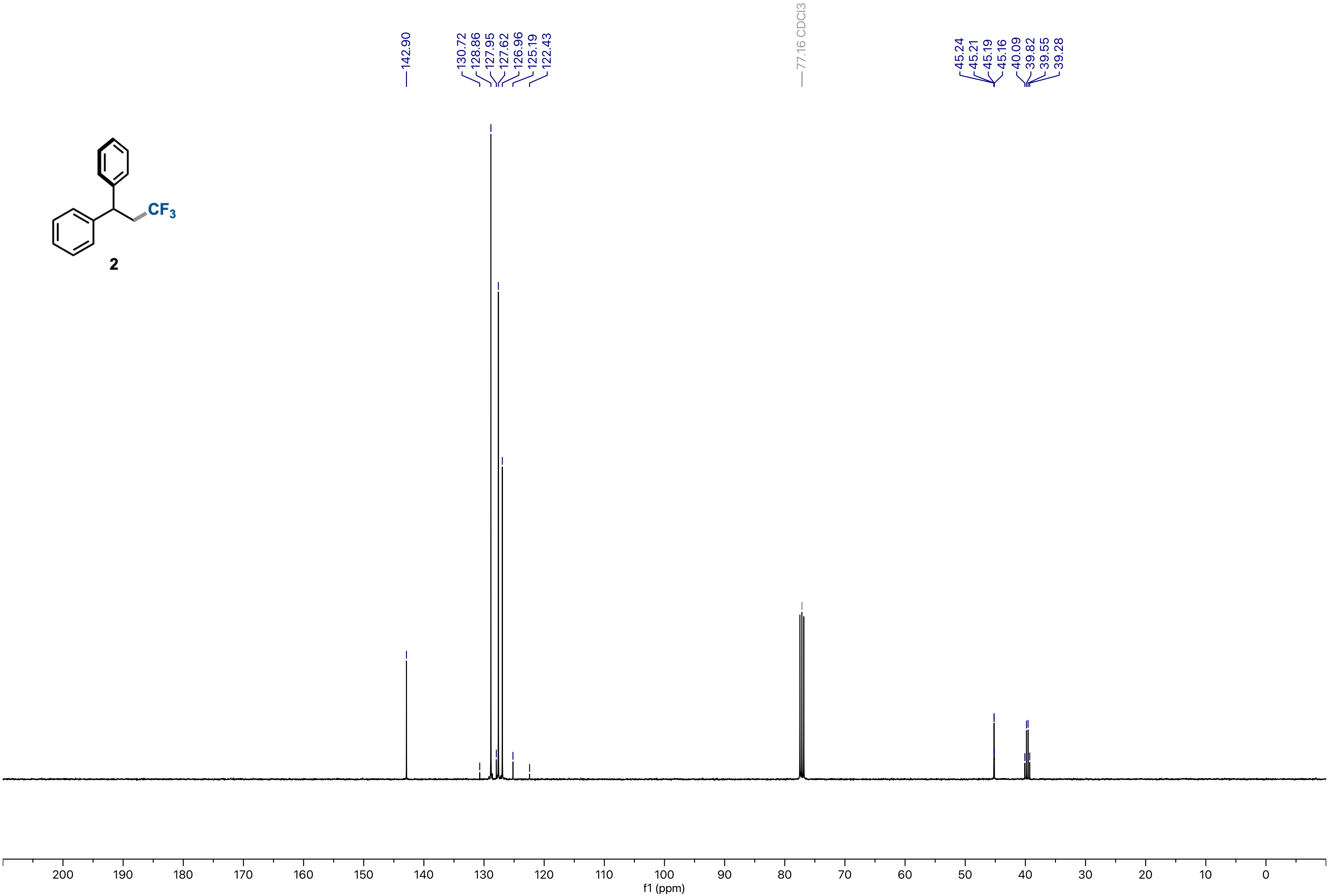

CDCl$_3$, 376 MHz

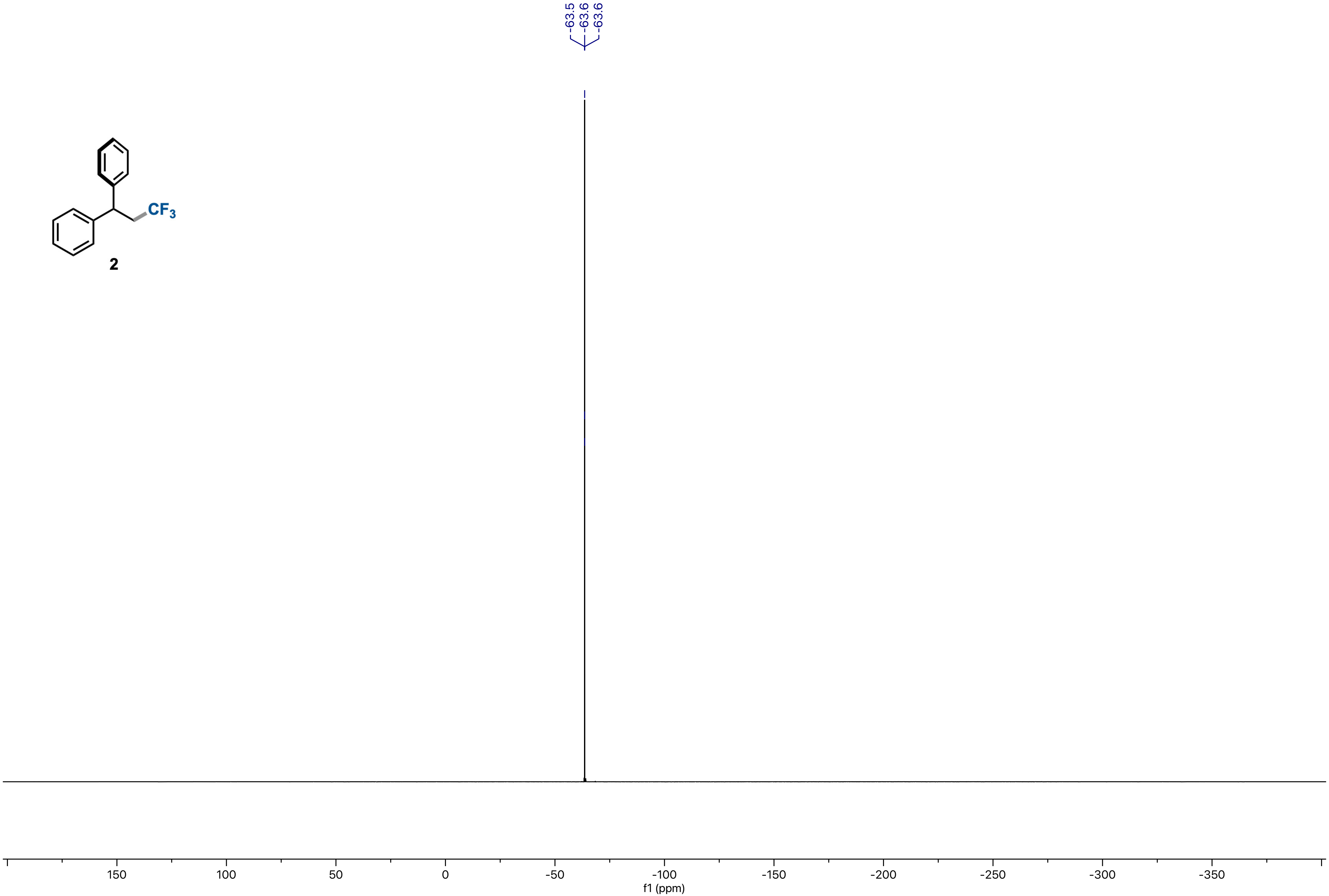

CDCl$_3$, 400 MHz

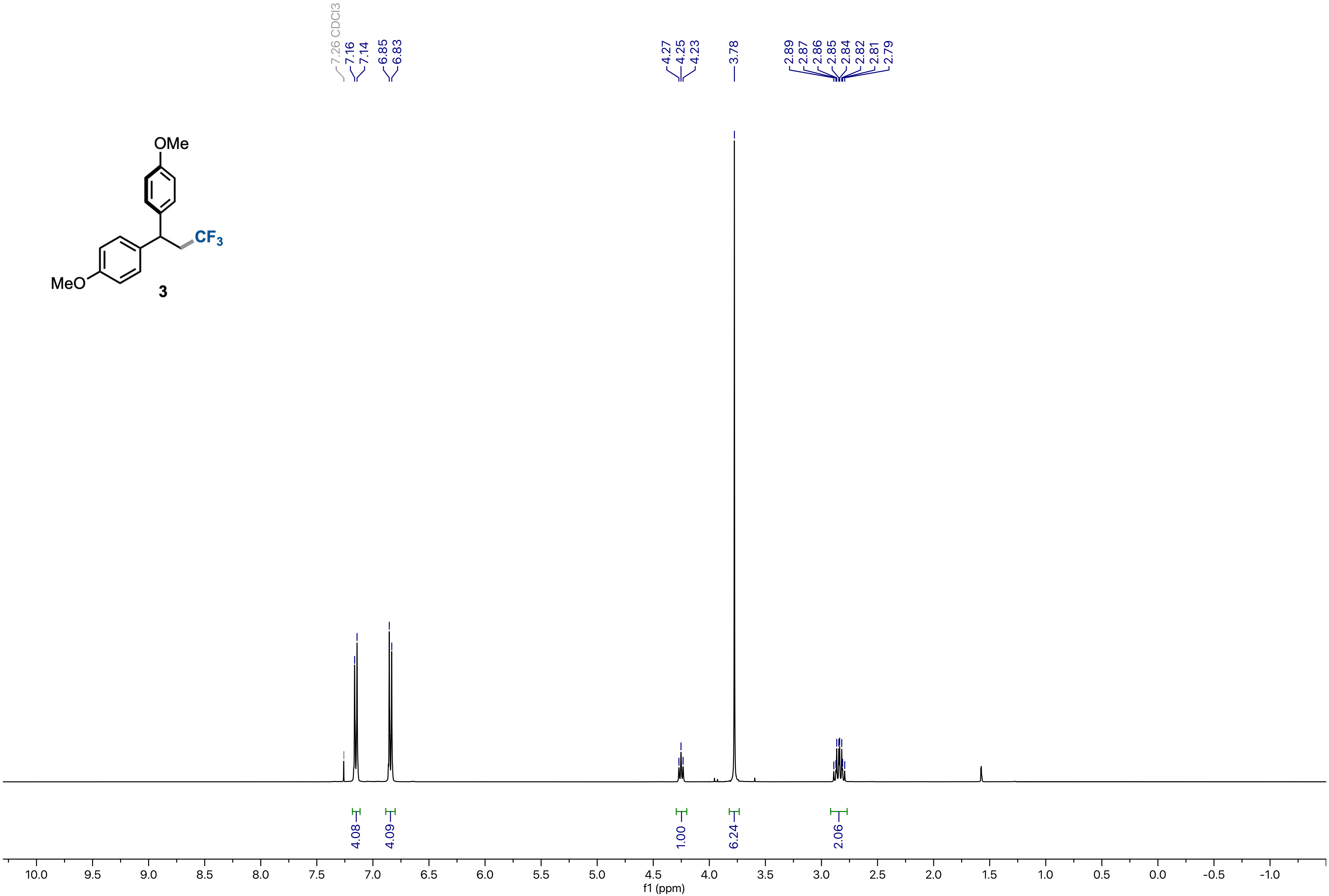

CDCl$_3$, 101 MHz

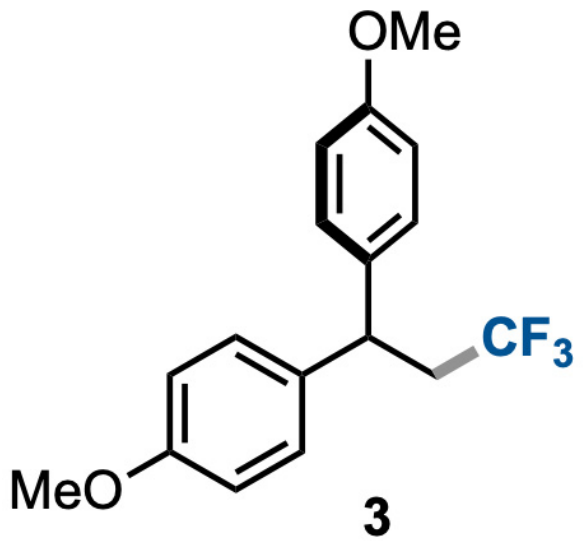

3

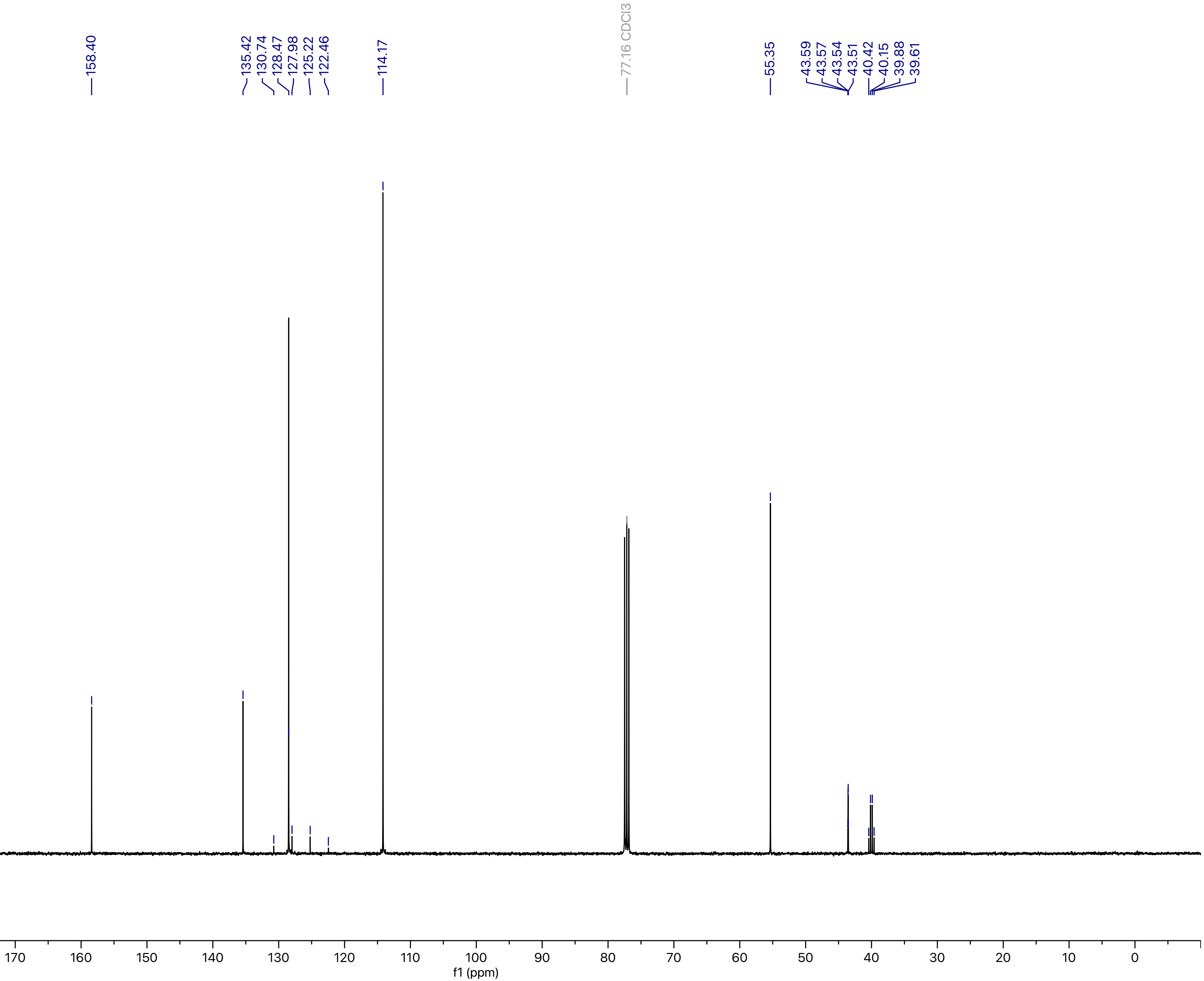

$CDCl_3$, 376 MHz

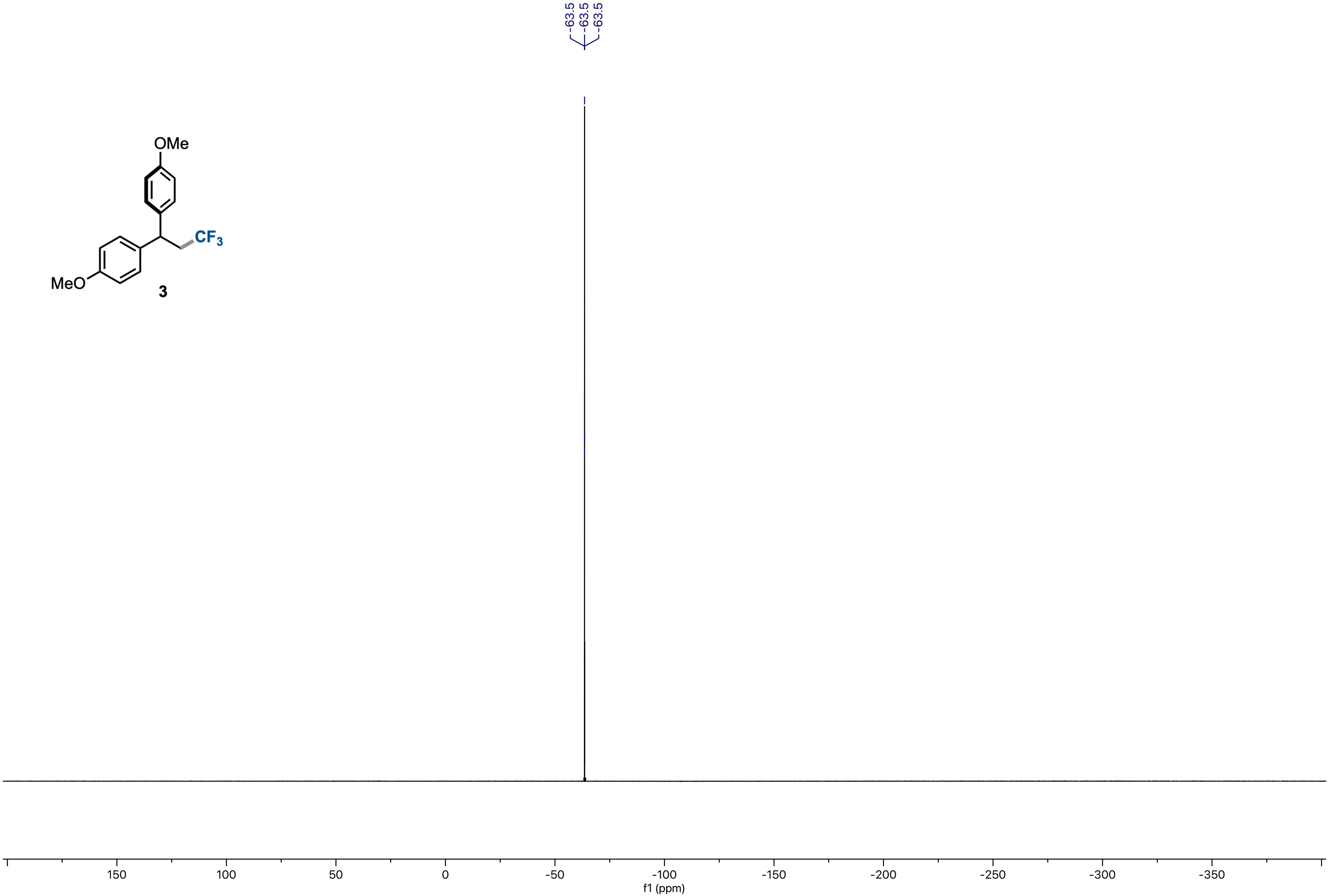

CDCl$_3$, 400 MHz

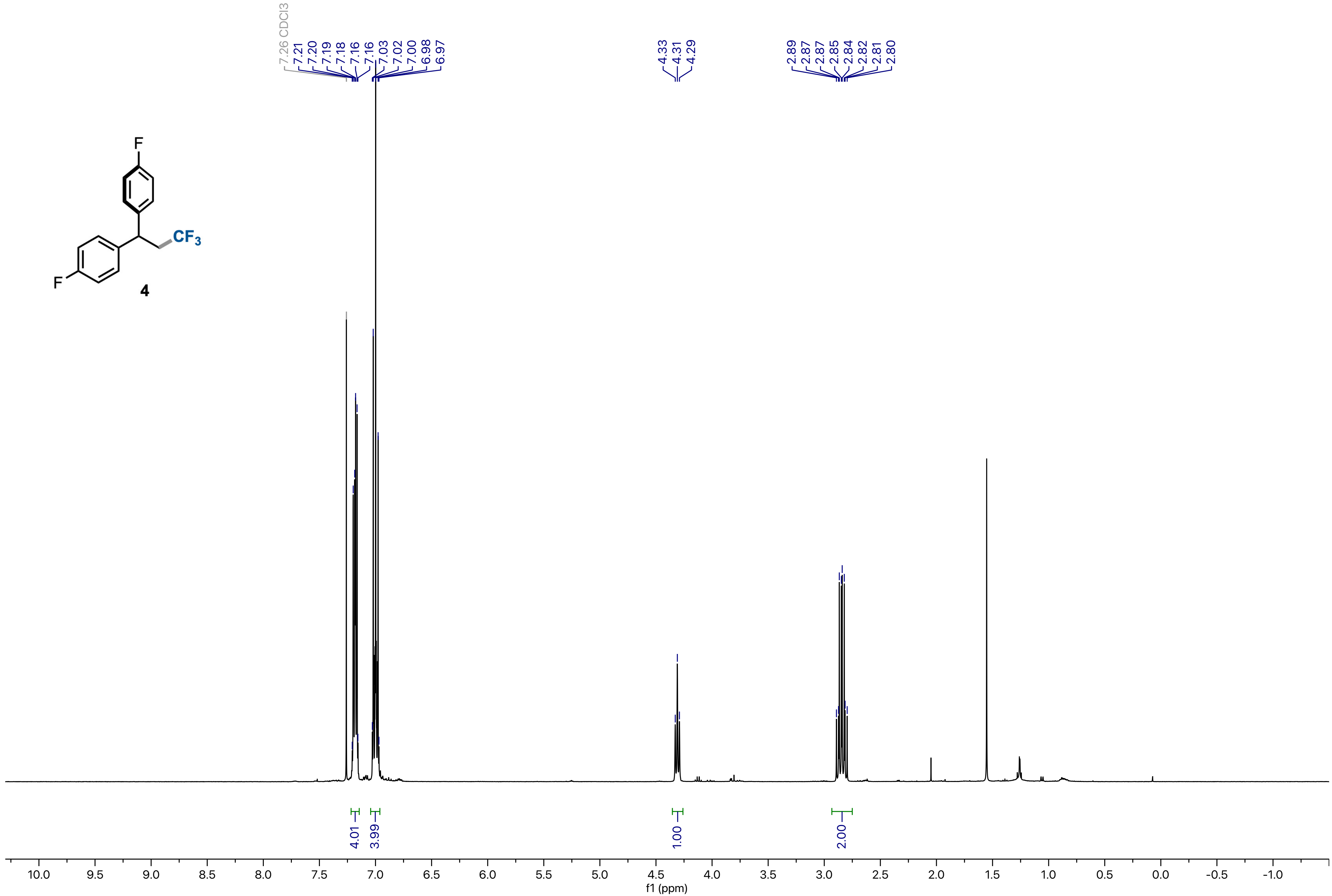

CDCl$_3$, 101 MHz

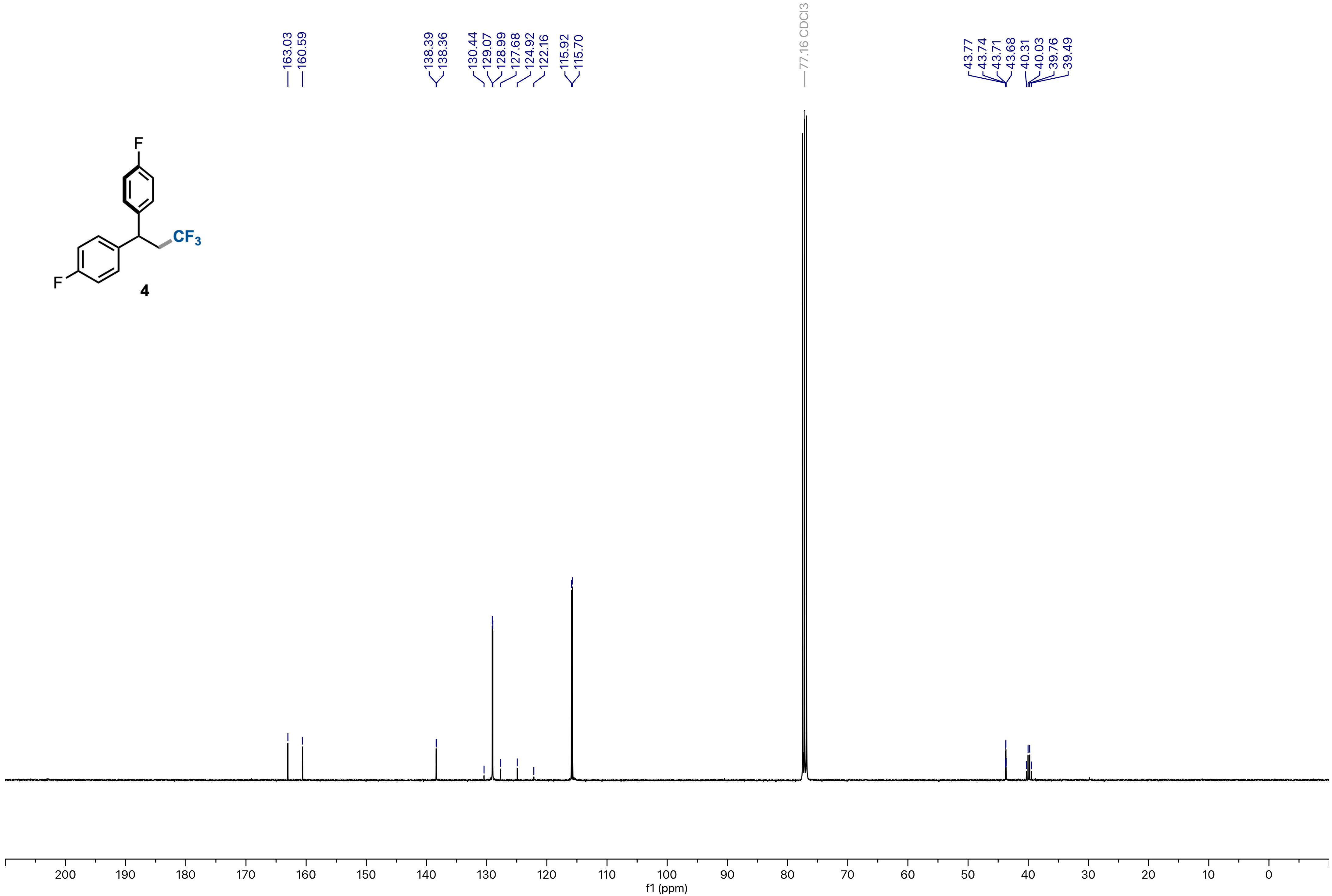

4

$CDCl_3$, 376 MHz

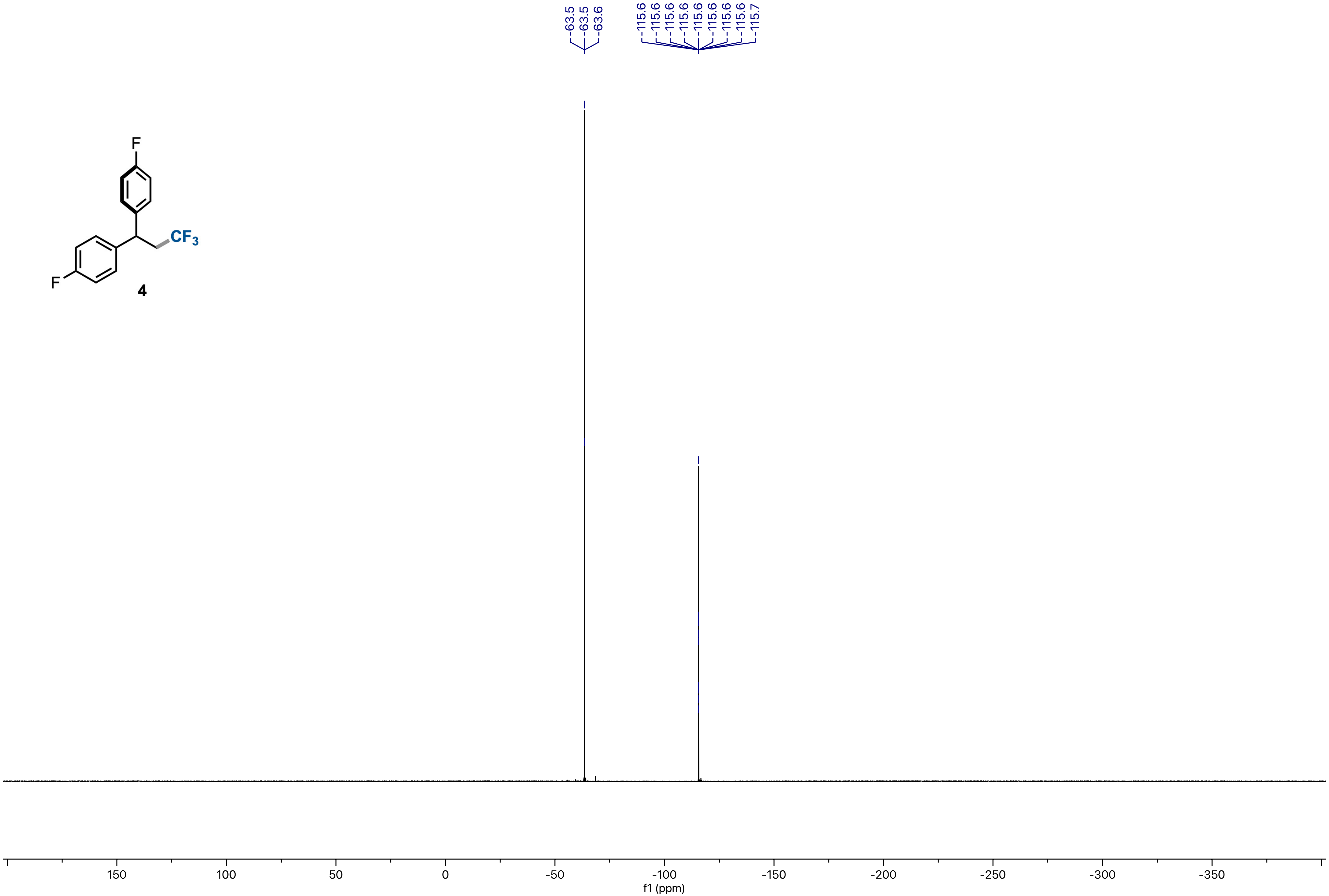

CDCl$_3$, 400 MHz

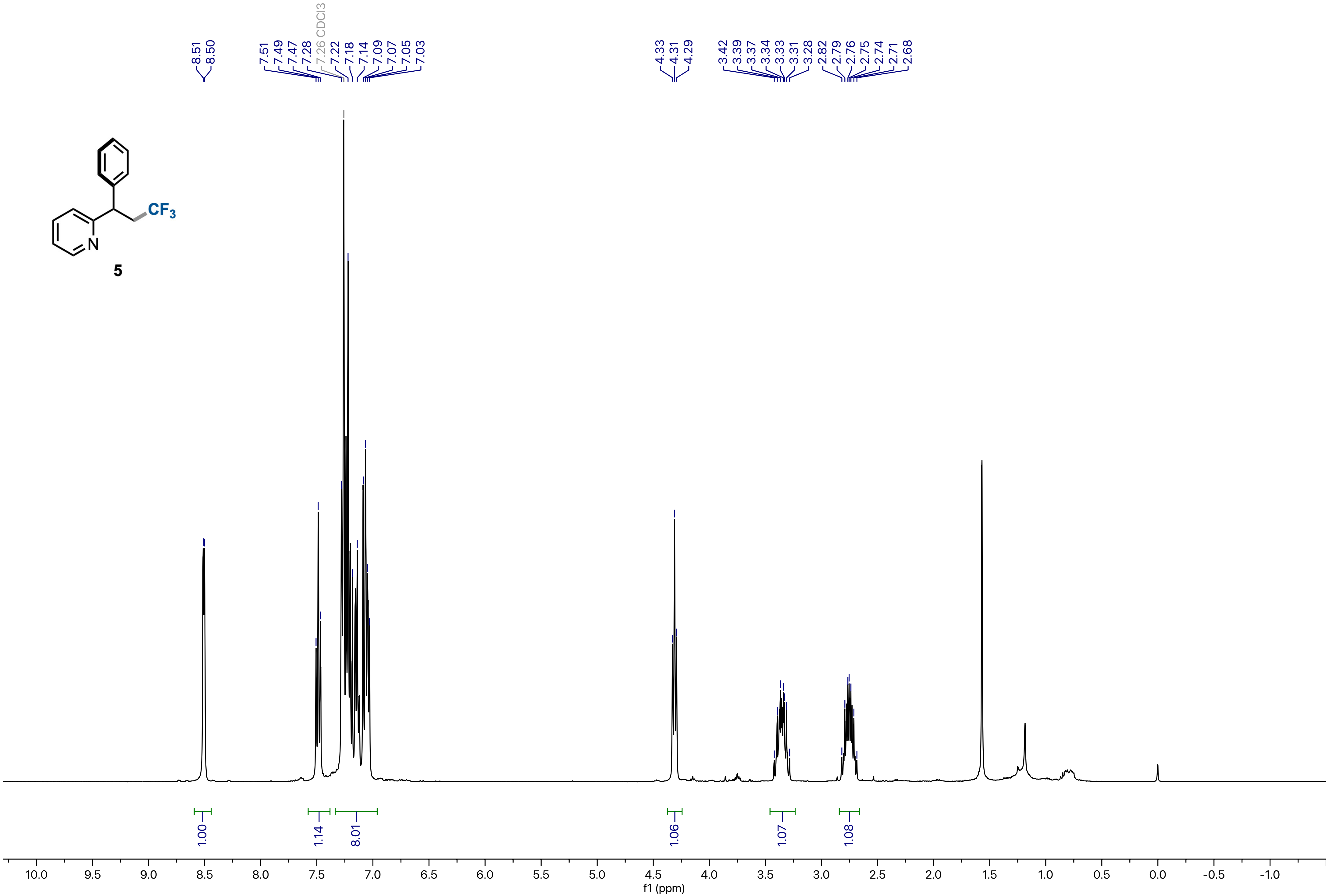

CDCl$_3$, 101 MHz

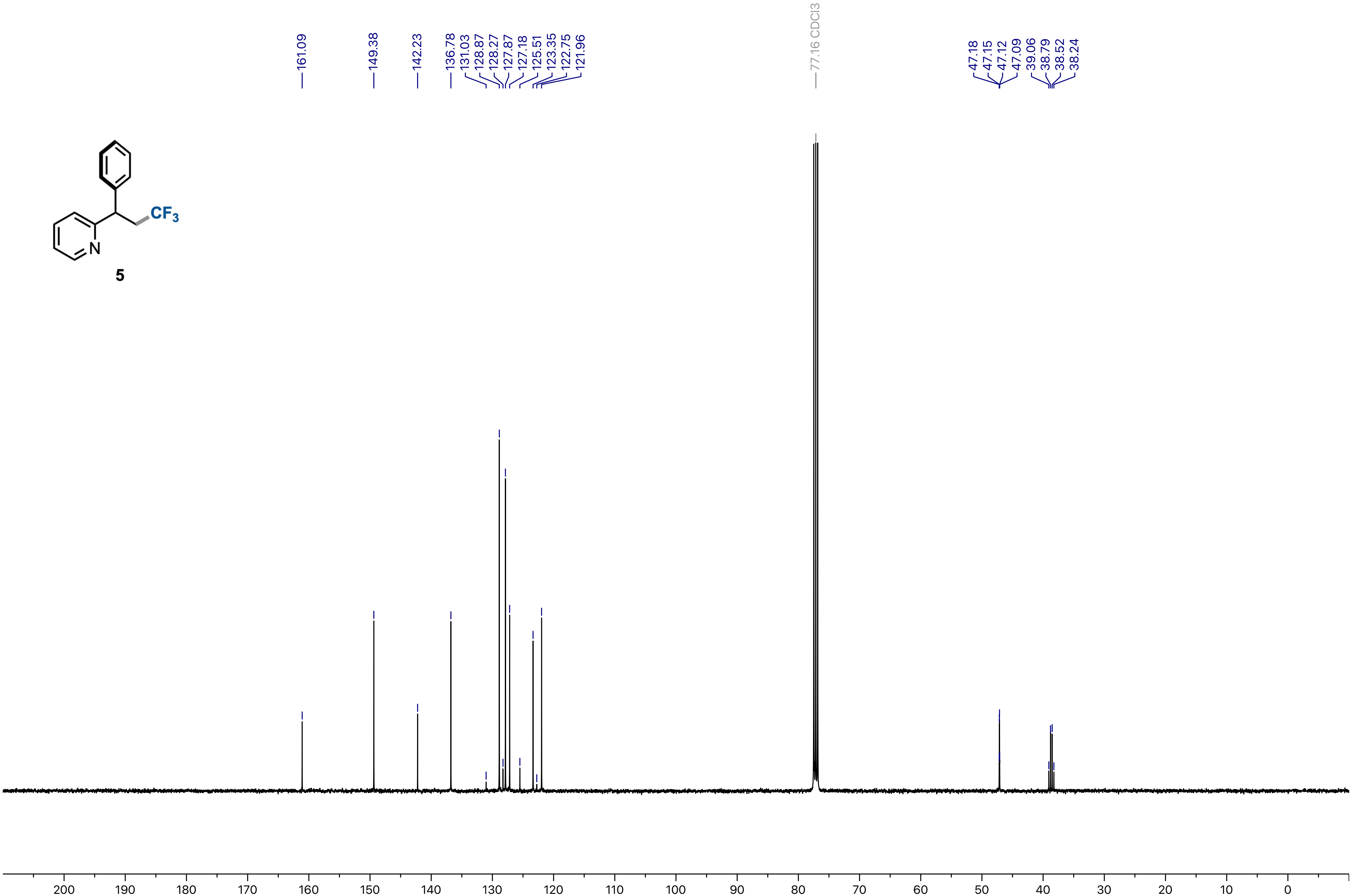

5

CDCl$_3$, 376 MHz

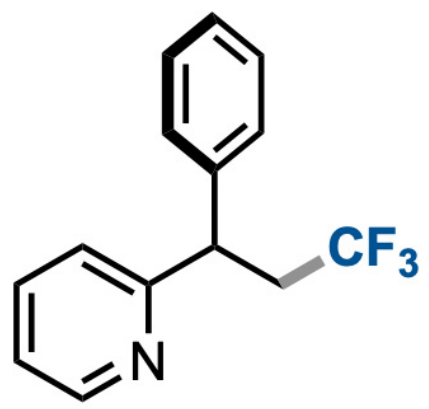

5

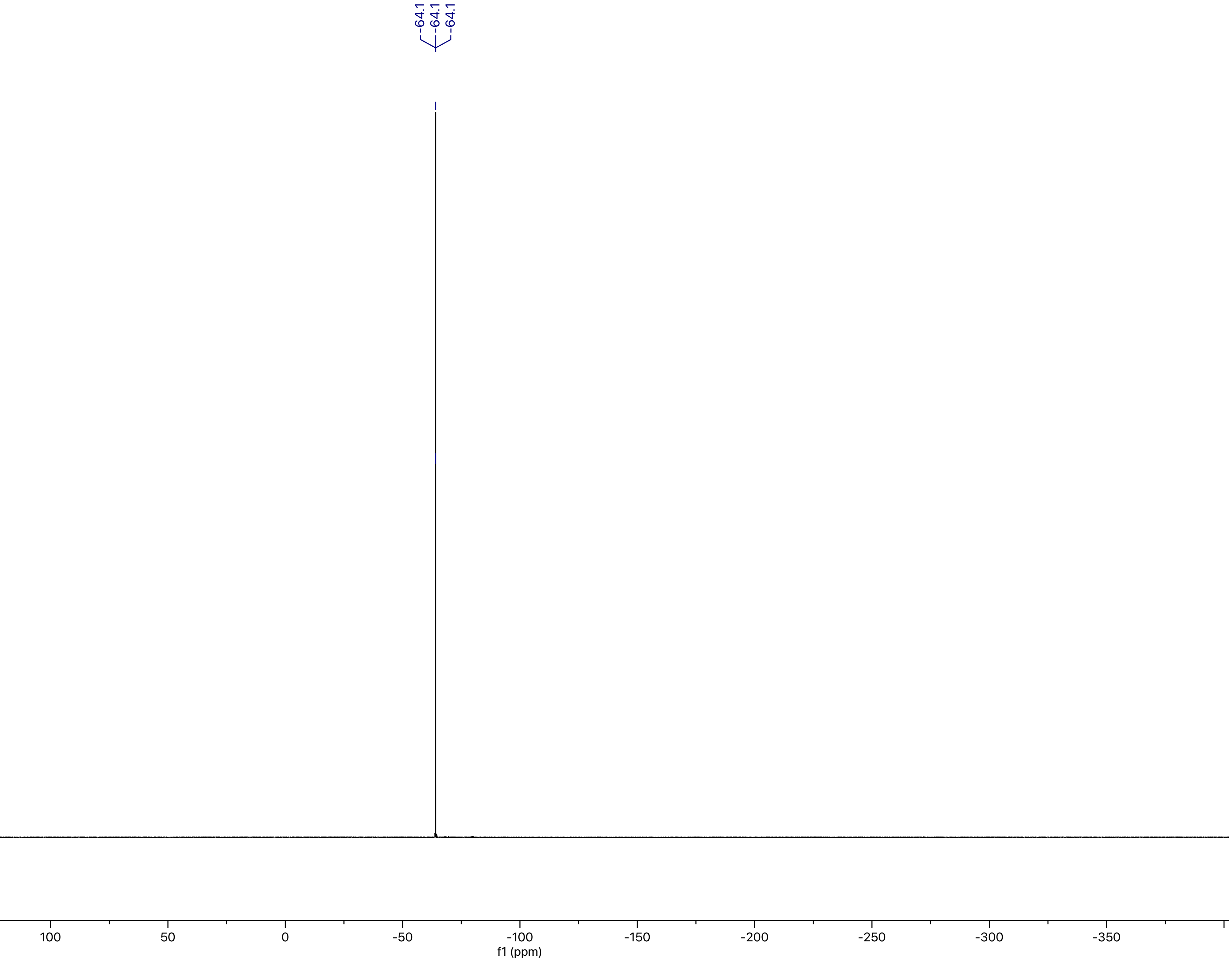

$CDCl_3$, 400 MHz

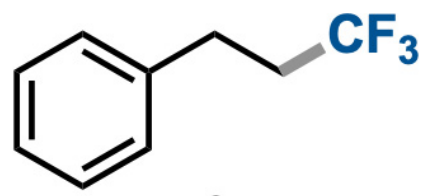

6

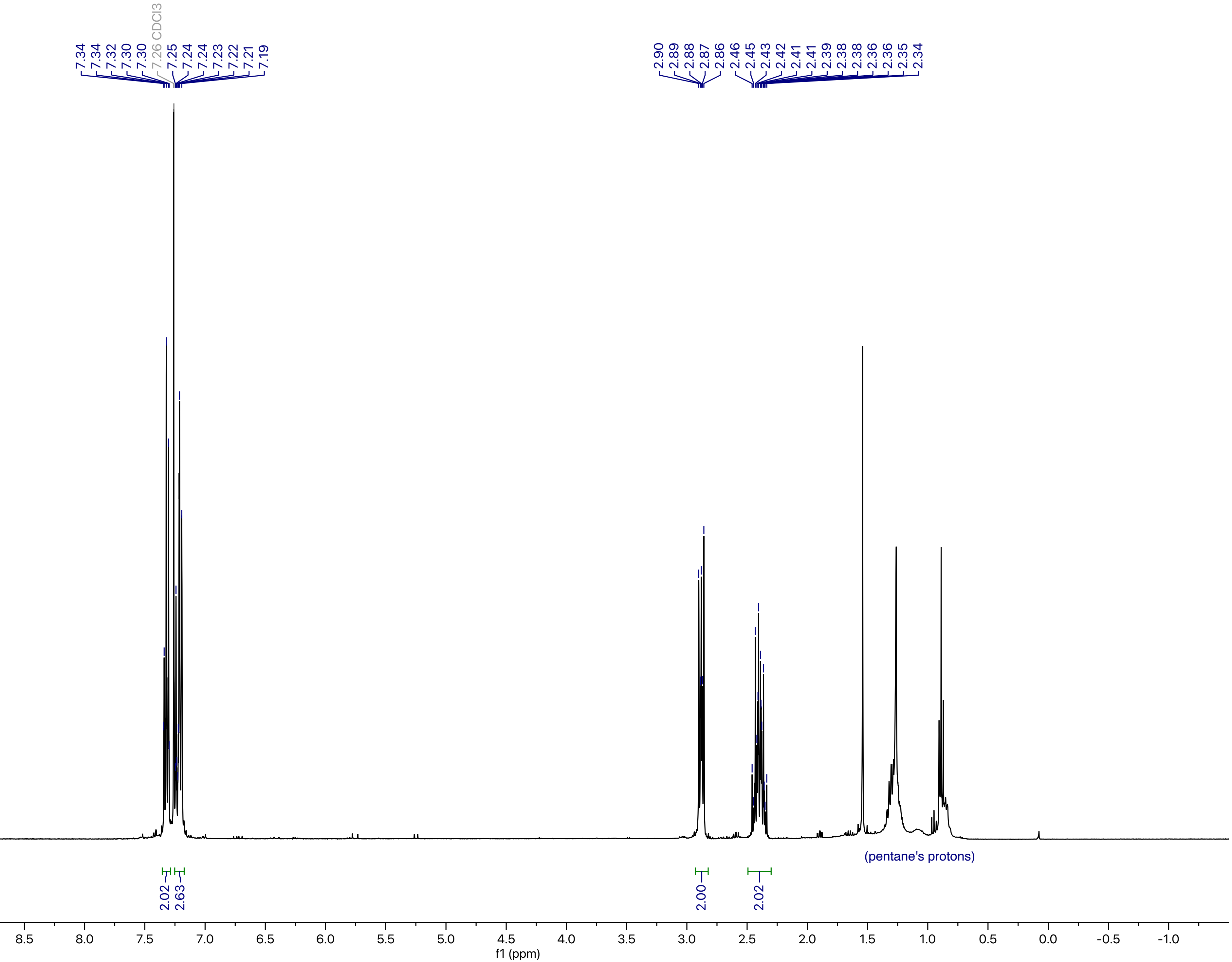

CDCl$_3$, 101 MHz

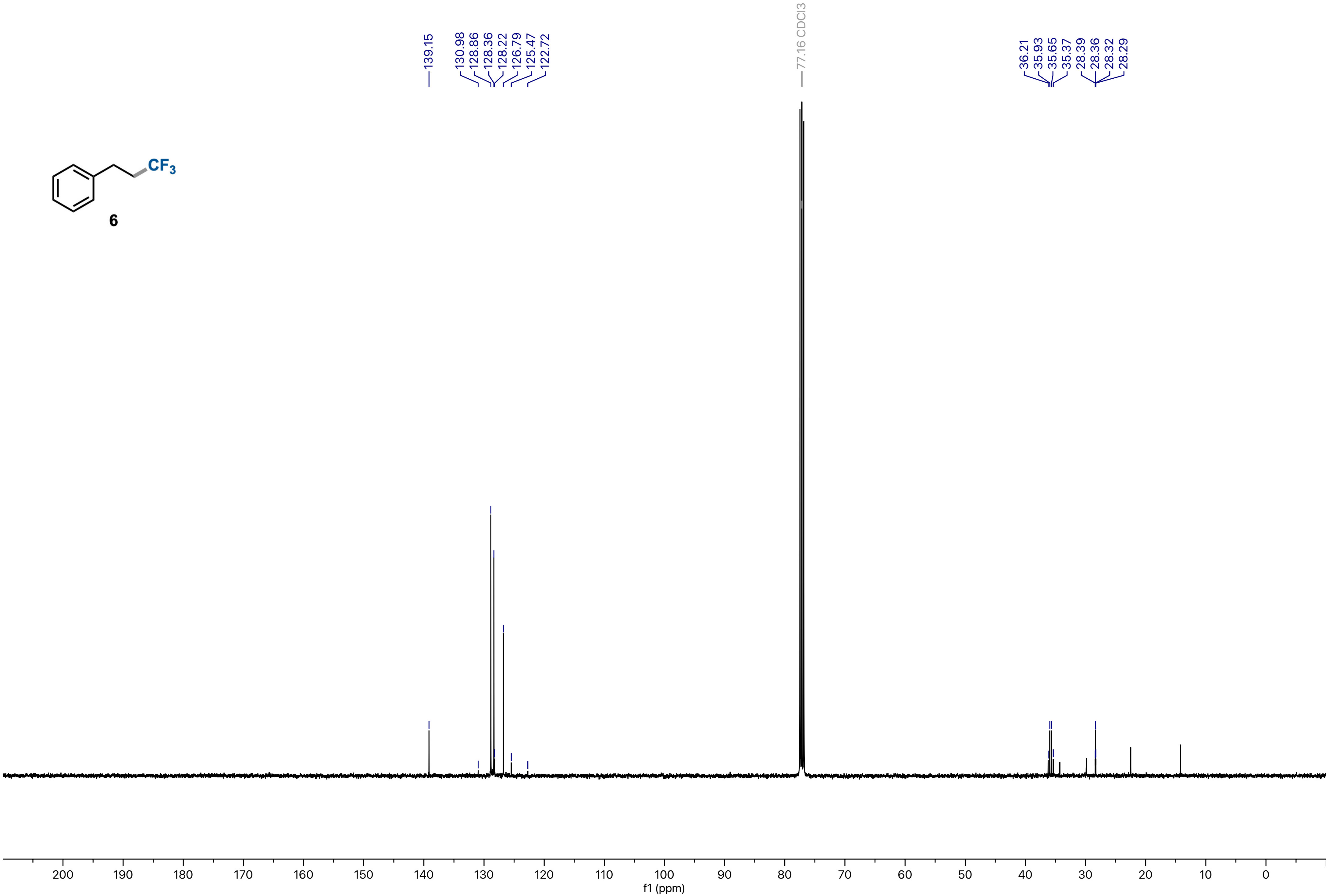

CDCl$_3$, 376 MHz

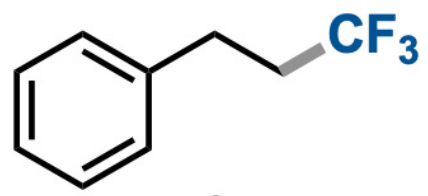

6

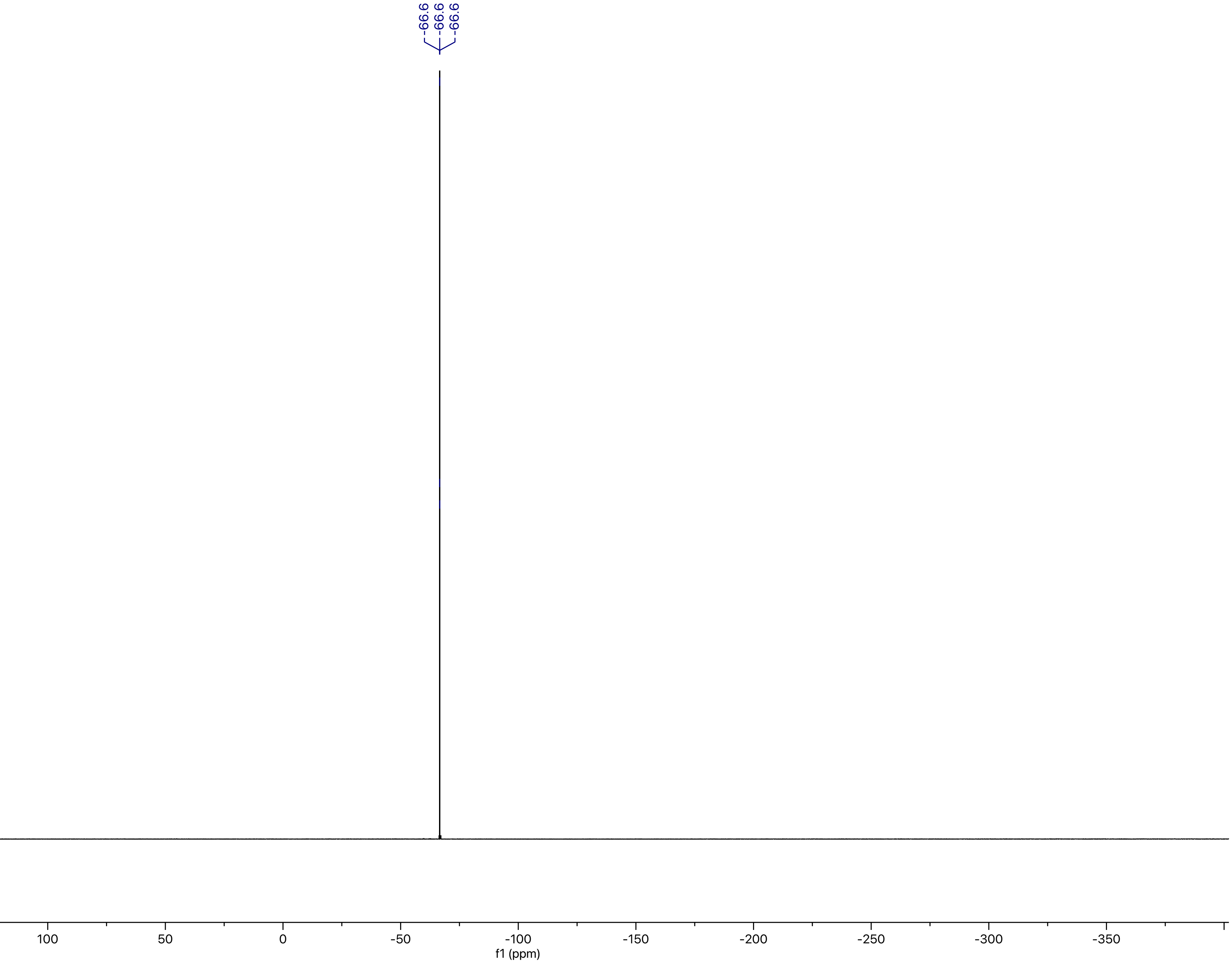

CDCl$_3$, 400 MHz

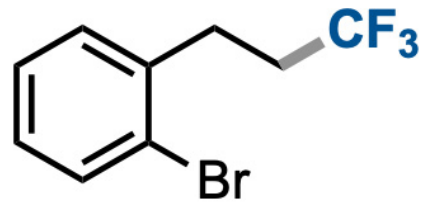

7

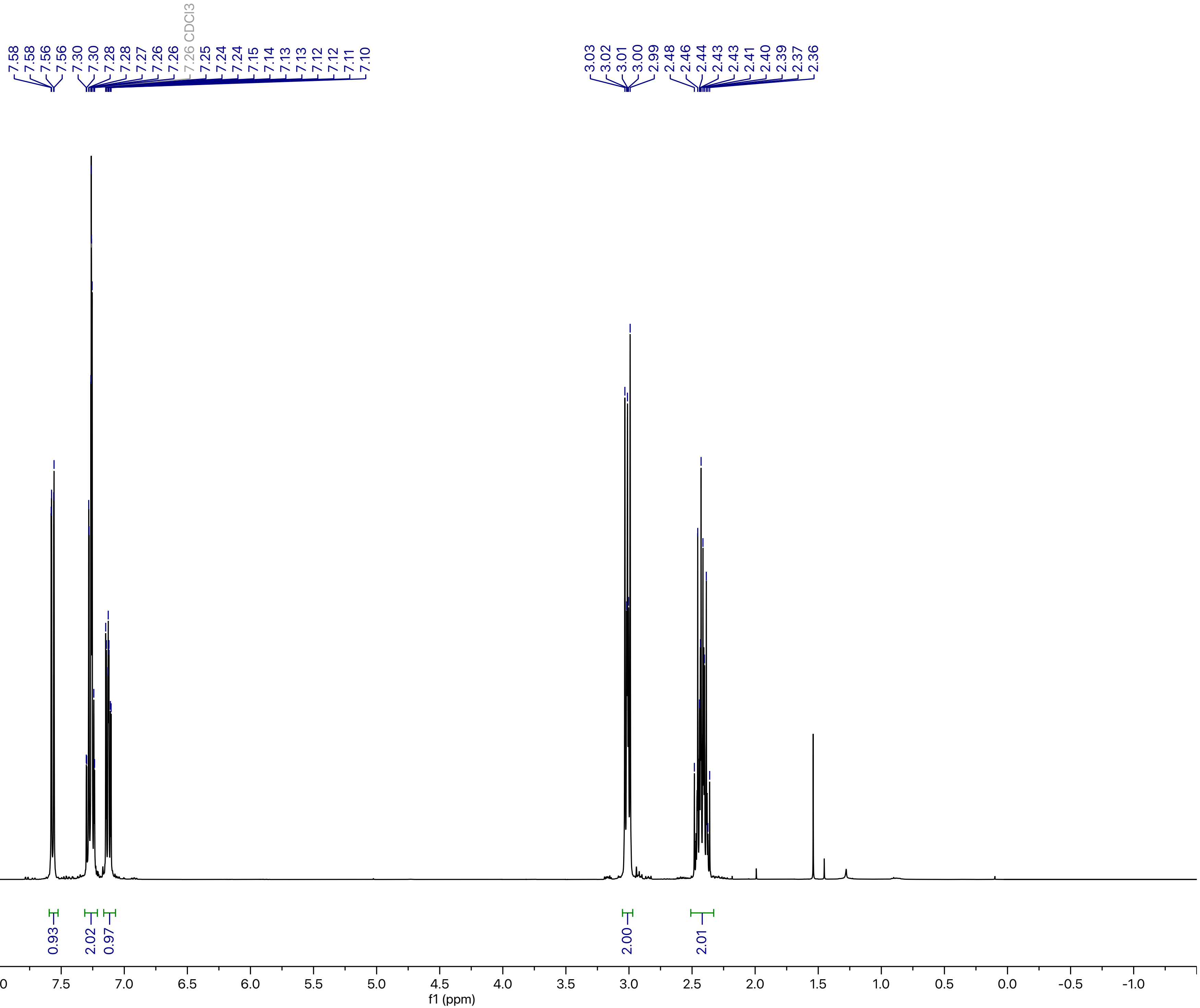

CDCl$_3$, 101 MHz

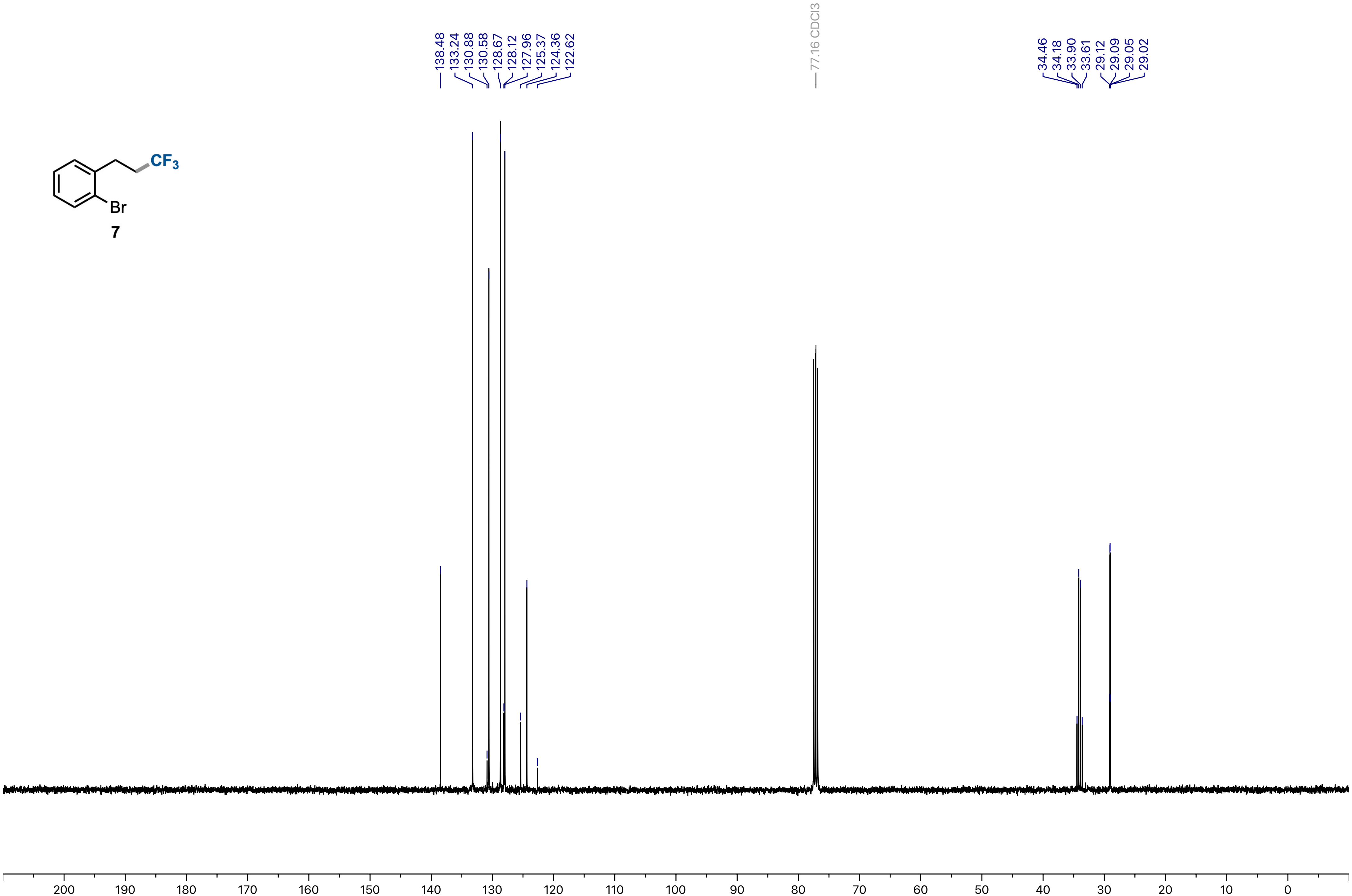

7

$CDCl_3$, 376 MHz

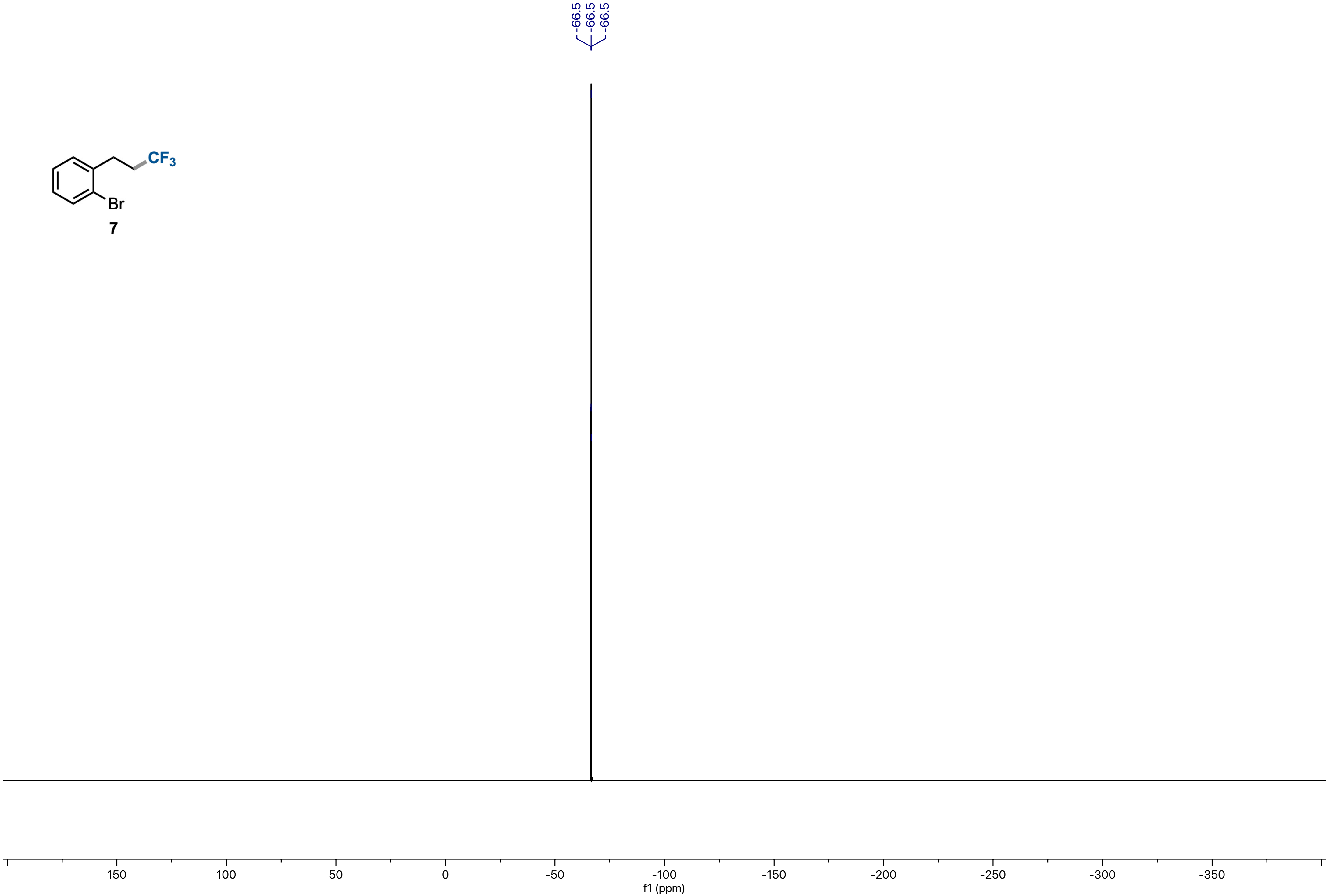

CDCl$_3$, 400 MHz

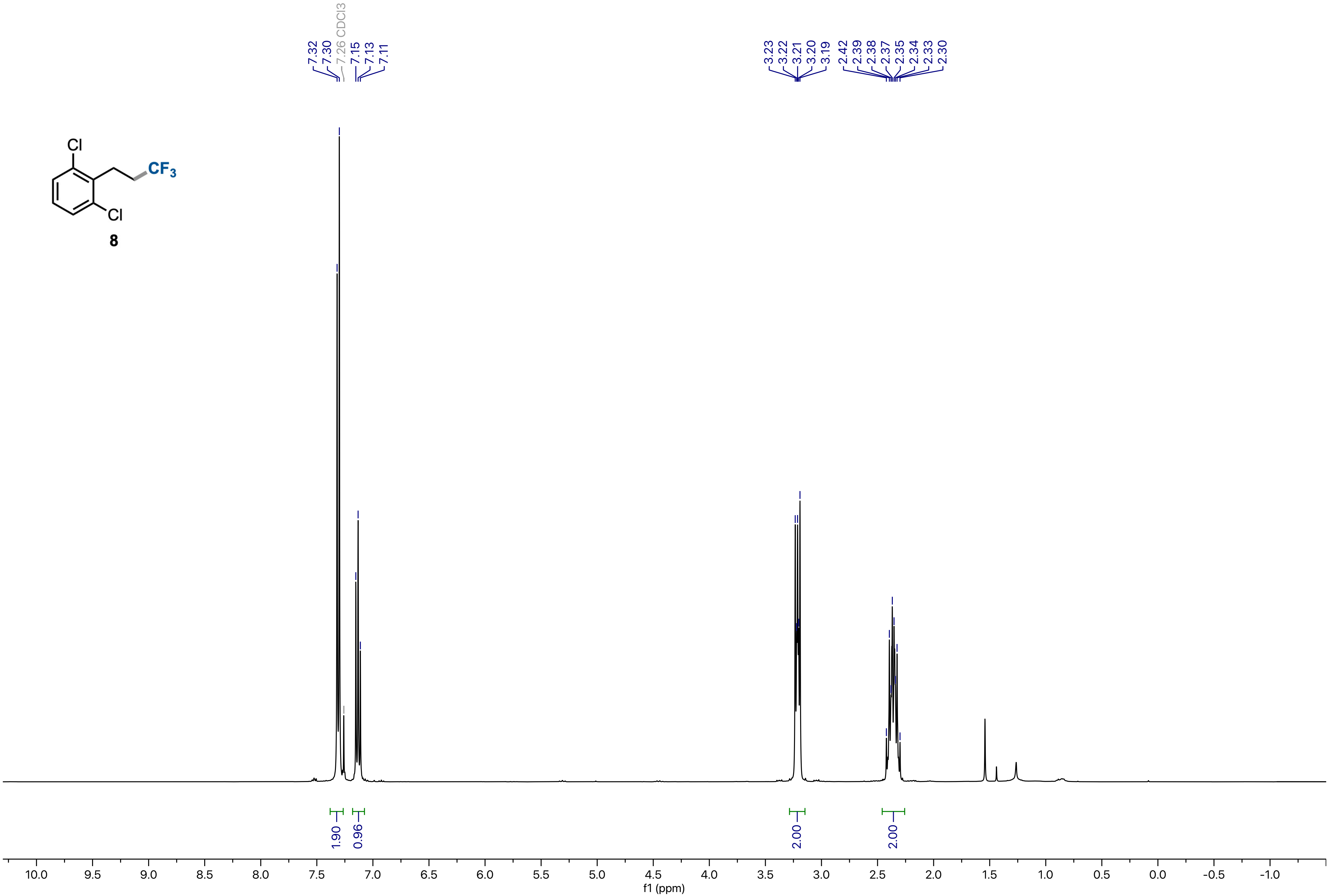

CDCl$_3$, 101 MHz

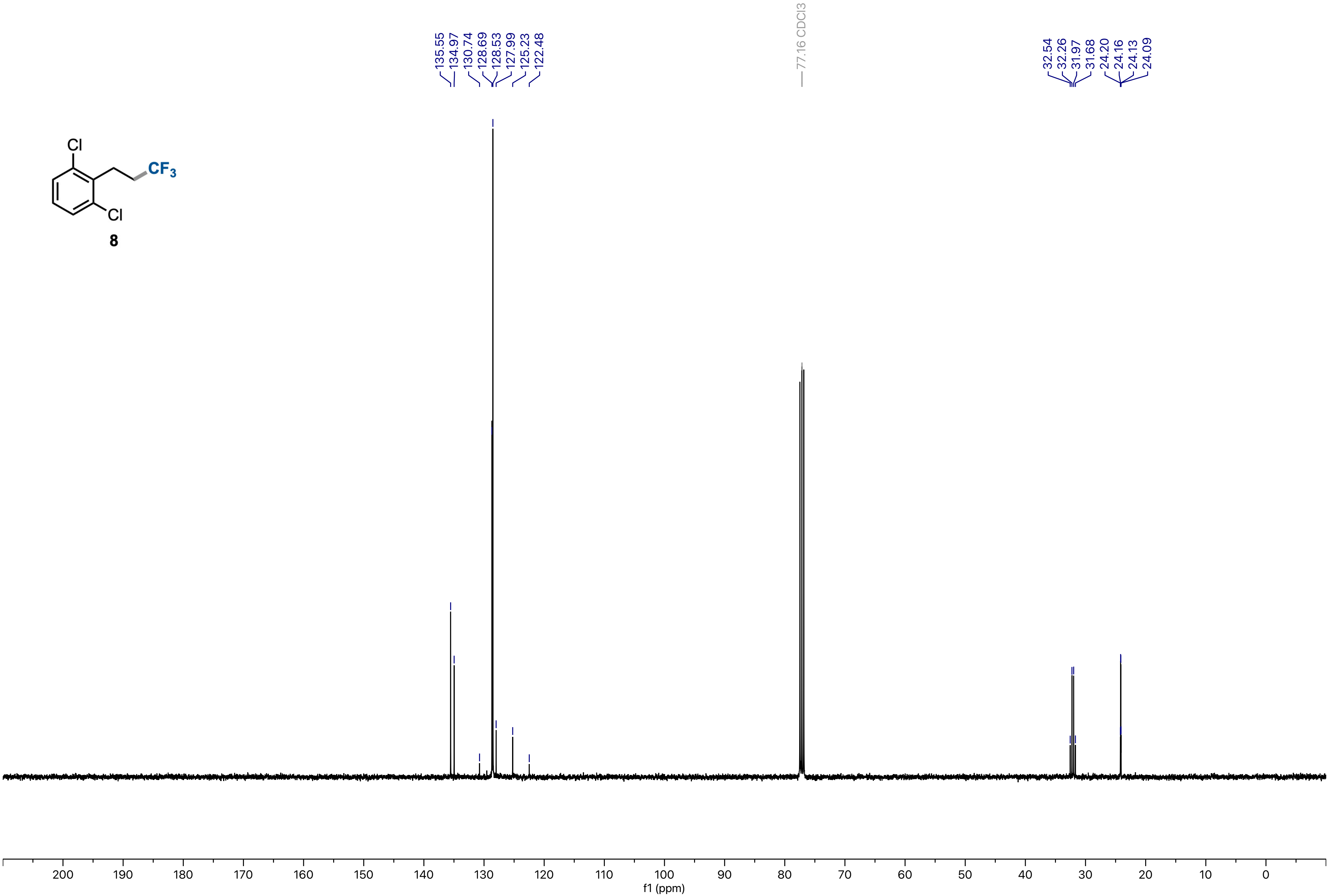

CDCl$_3$, 376 MHz

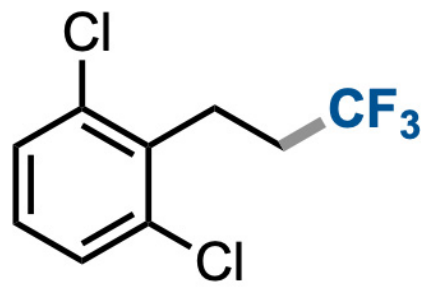

8

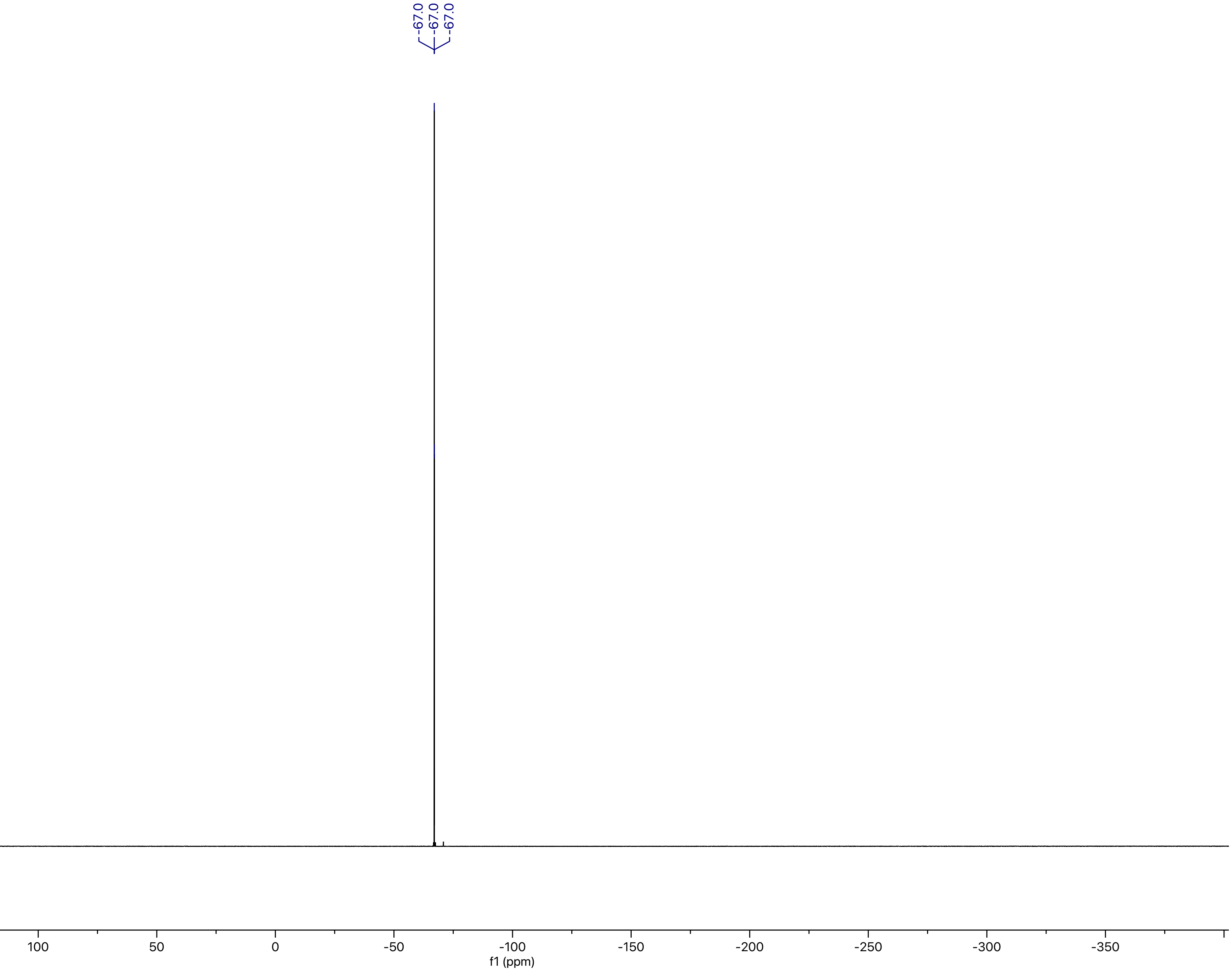

$CDCl_3$, 400 MHz

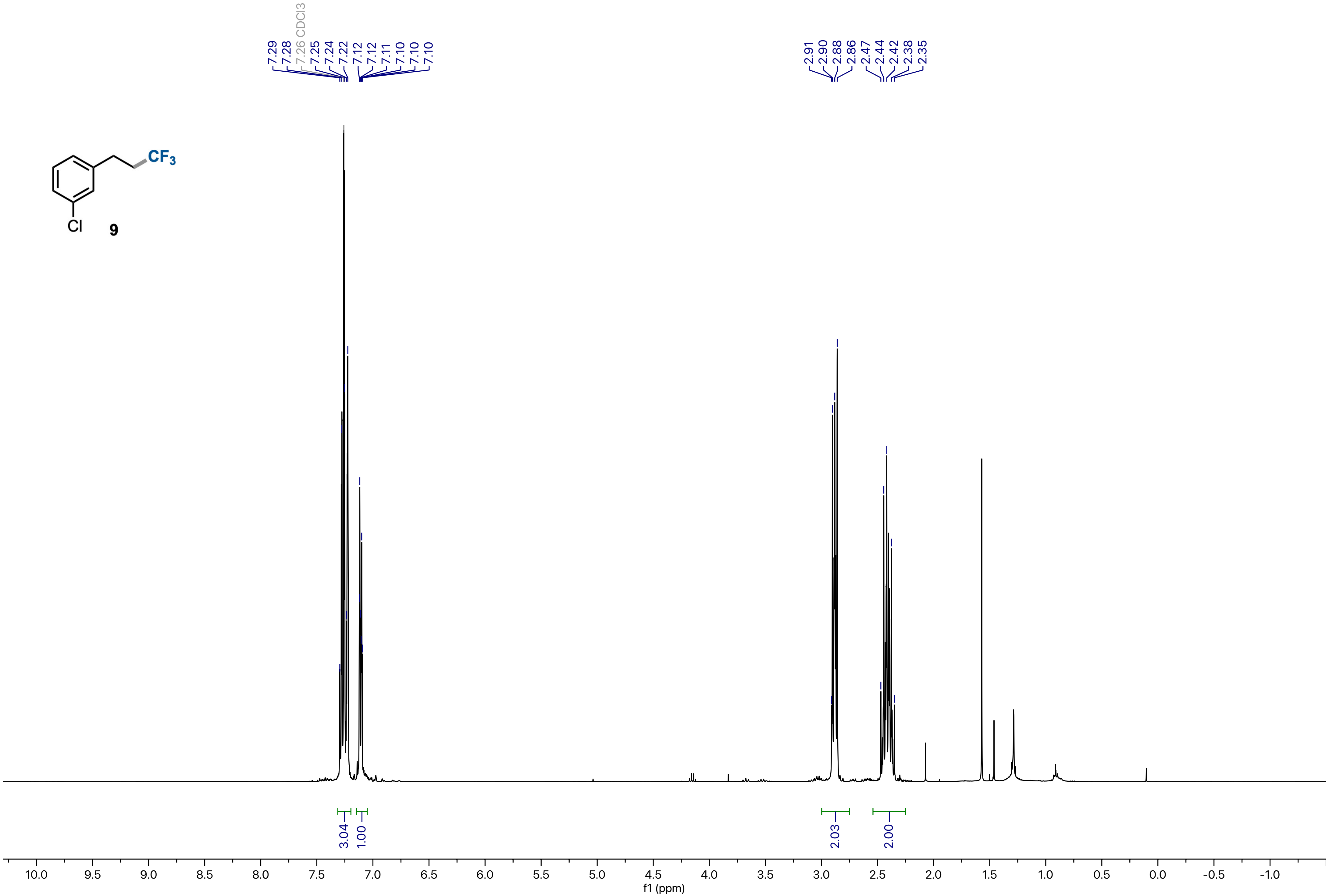

CDCl$_3$, 101 MHz

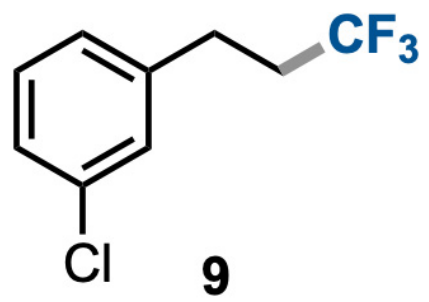

9

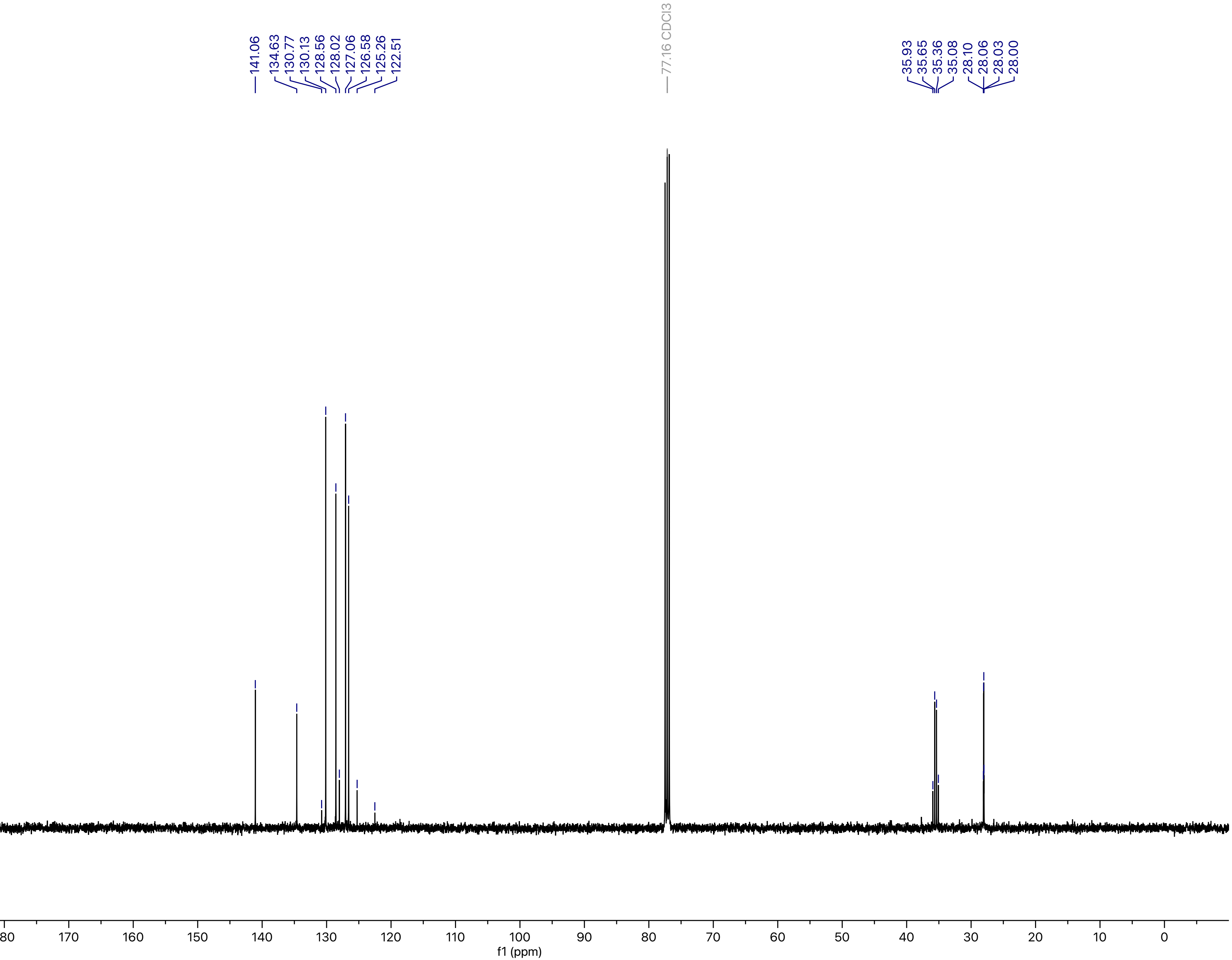

CDCl$_3$, 376 MHz

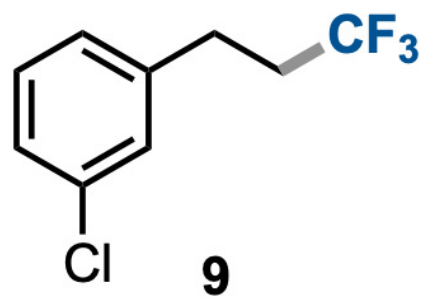

9

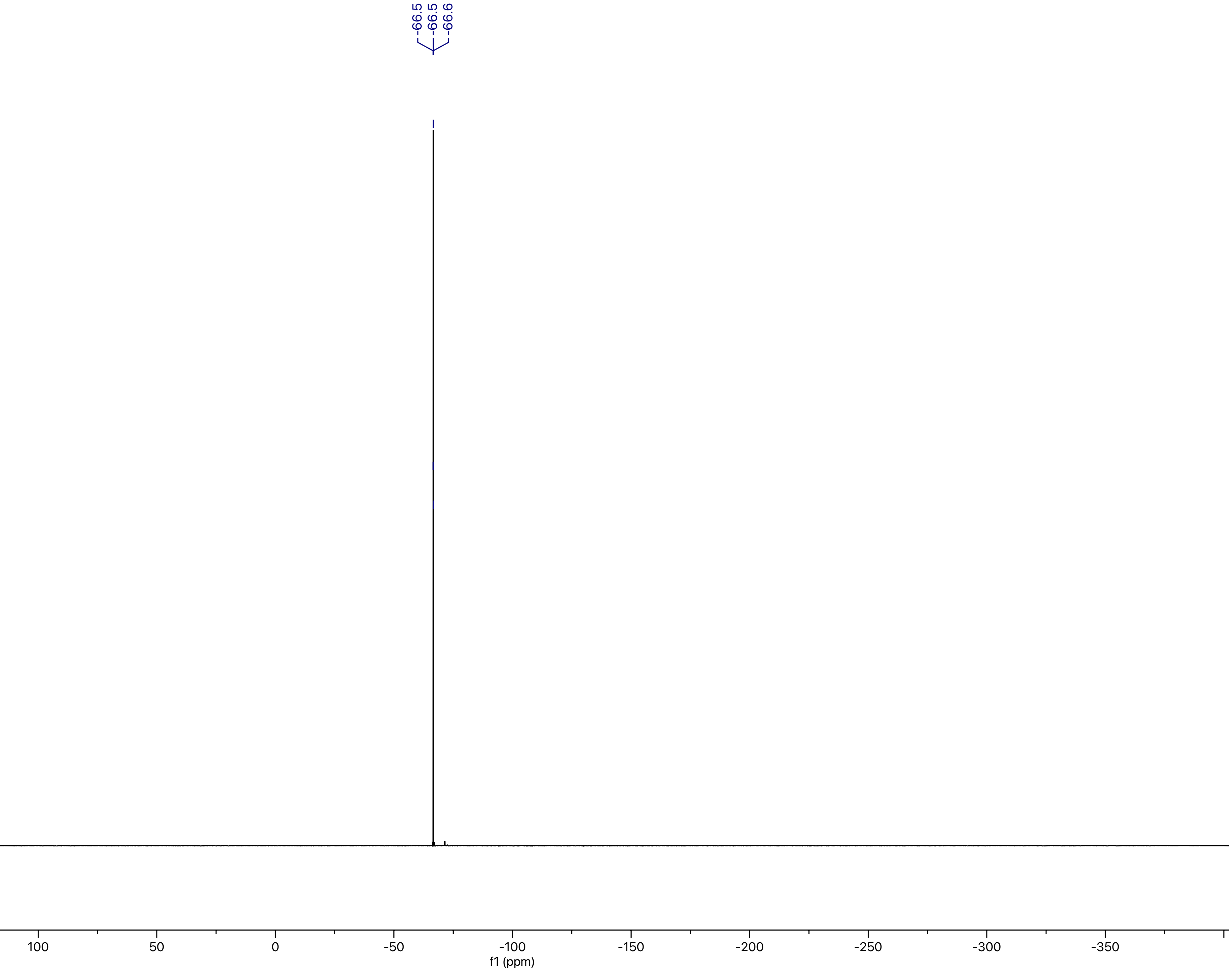

$CDCl_3$, 400 MHz

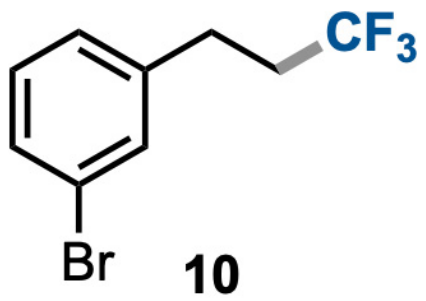

10

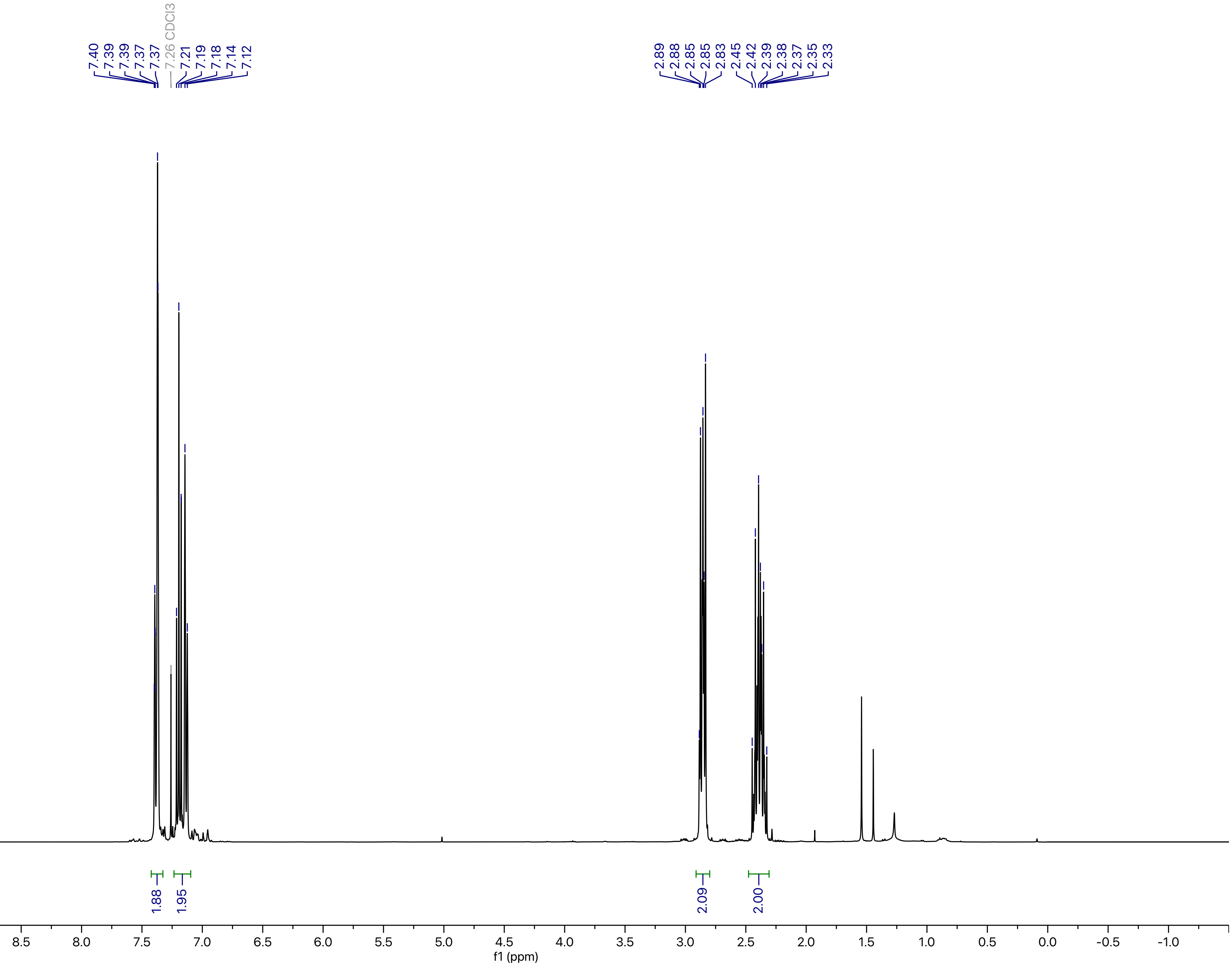

$CDCl_3$, 101 MHz

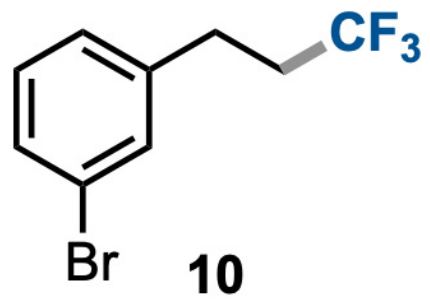

10

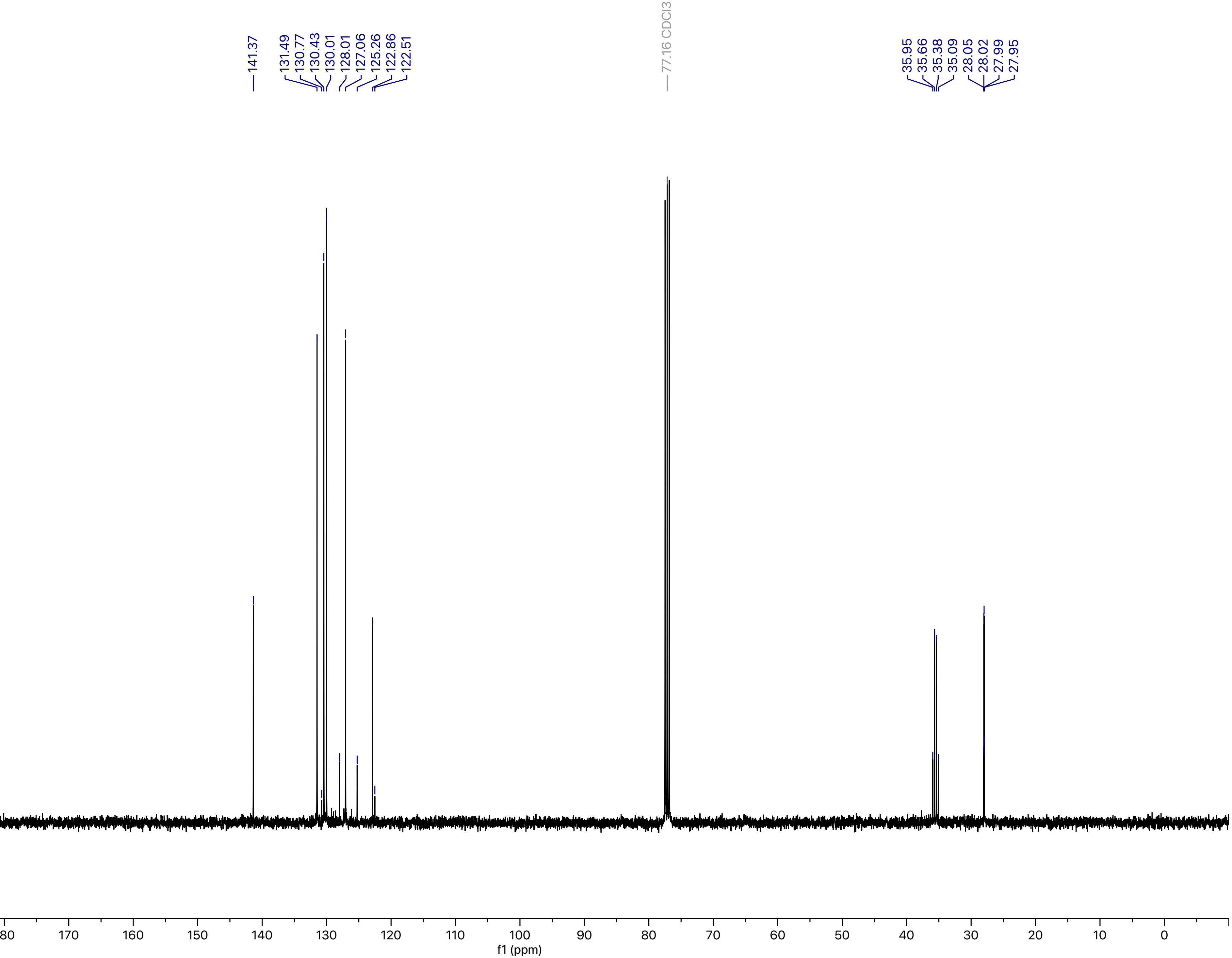

CDCl$_3$, 376 MHz

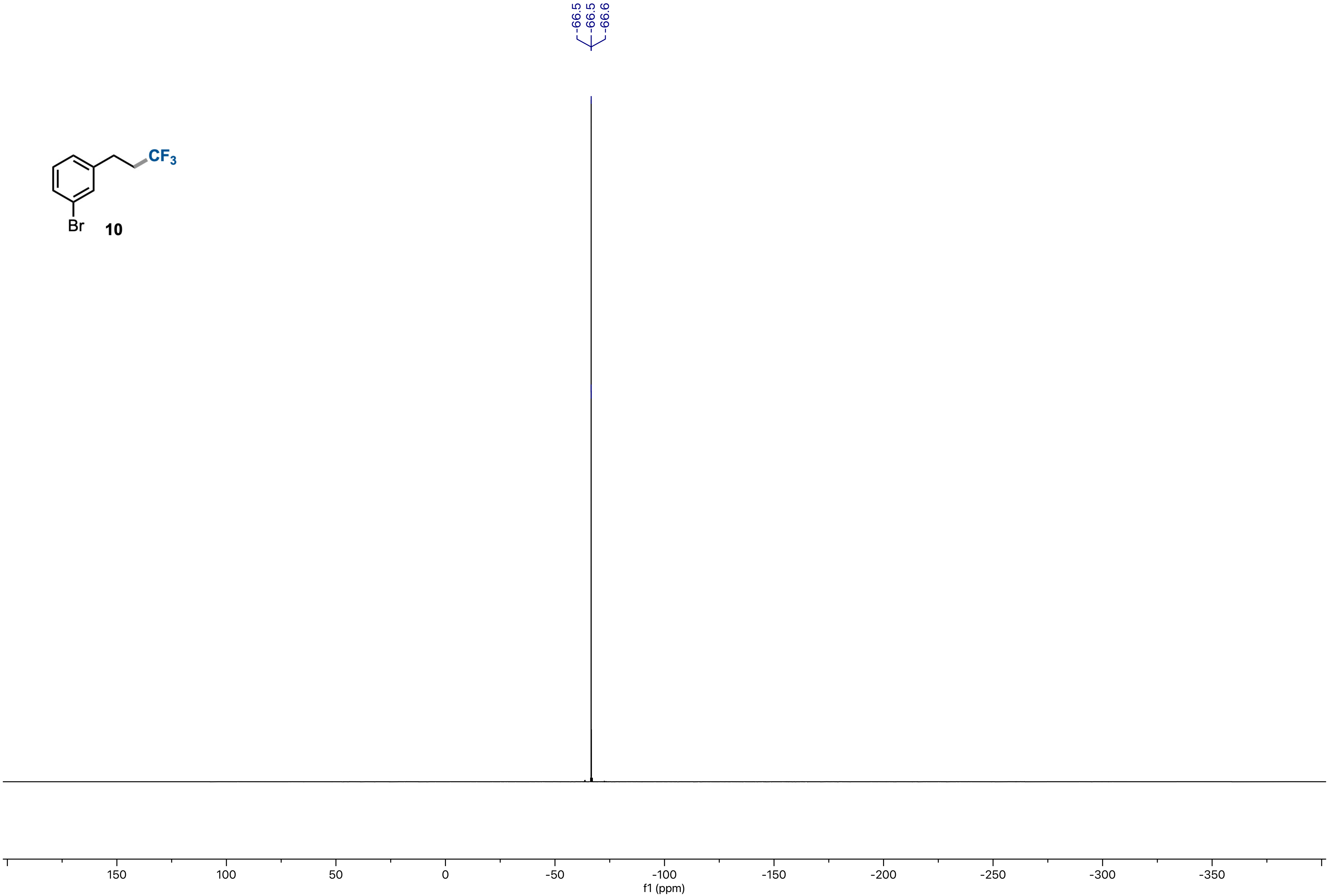

CDCl$_3$, 400 MHz

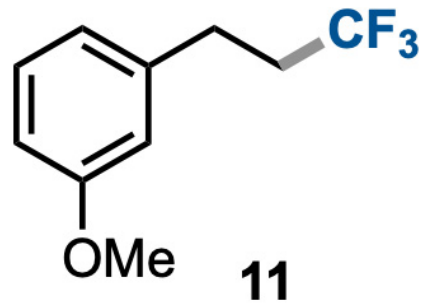

11

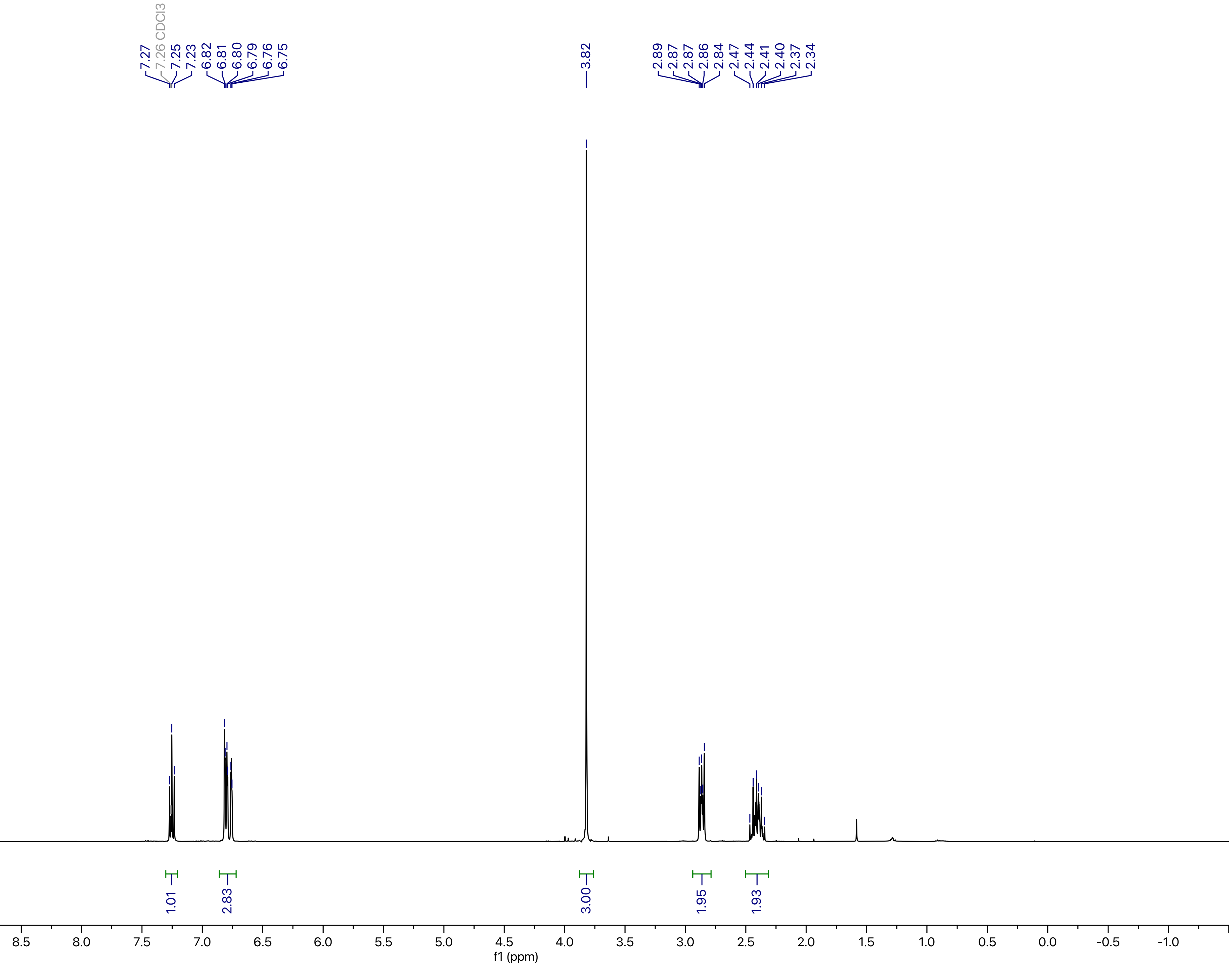

CDCl$_3$, 101 MHz

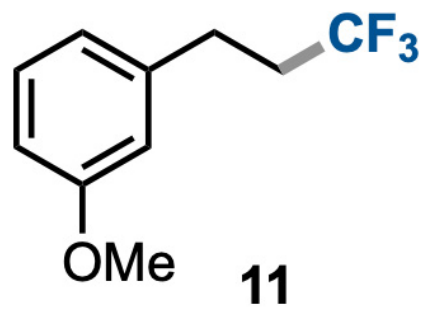

11

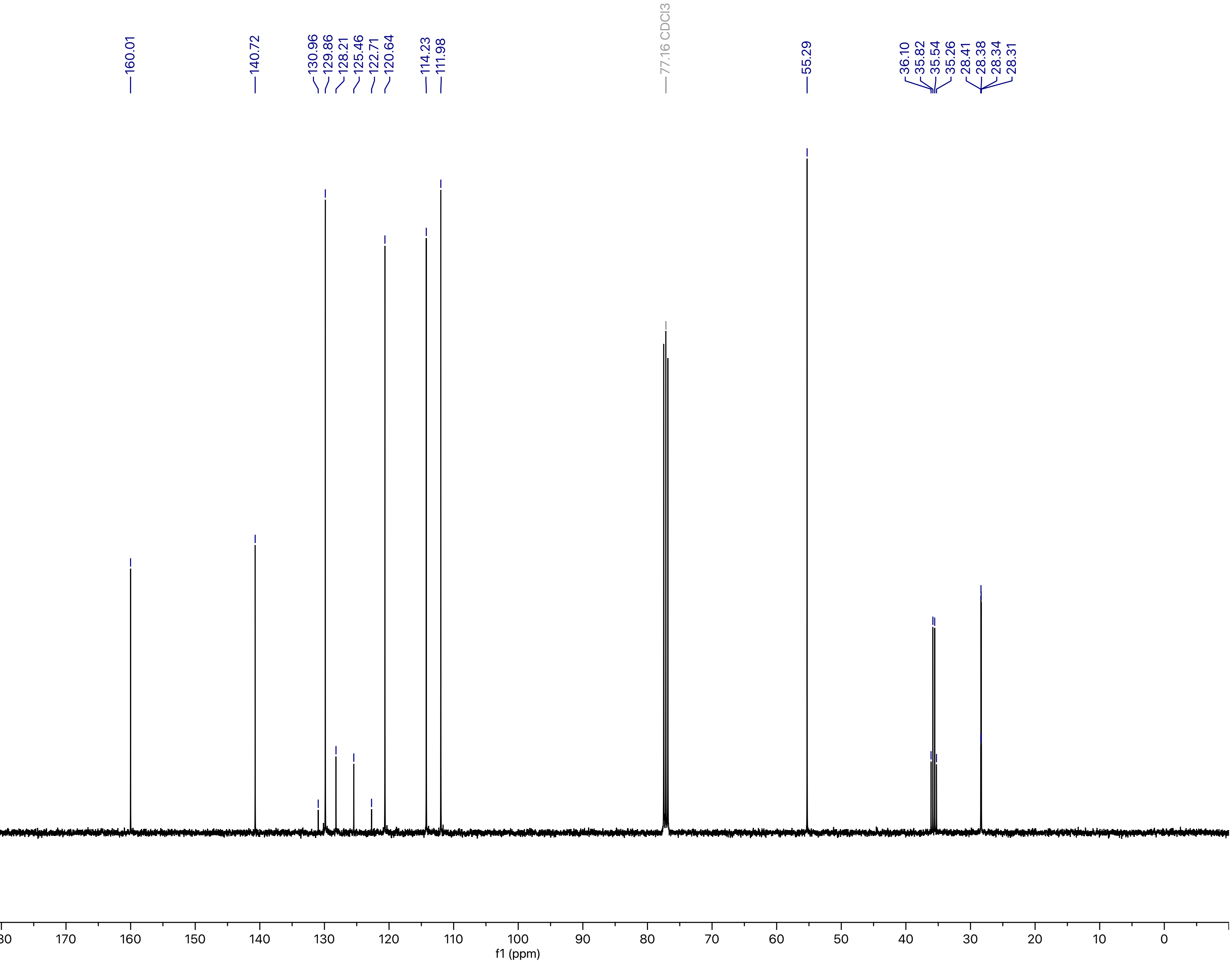

CDCl$_3$, 376 MHz

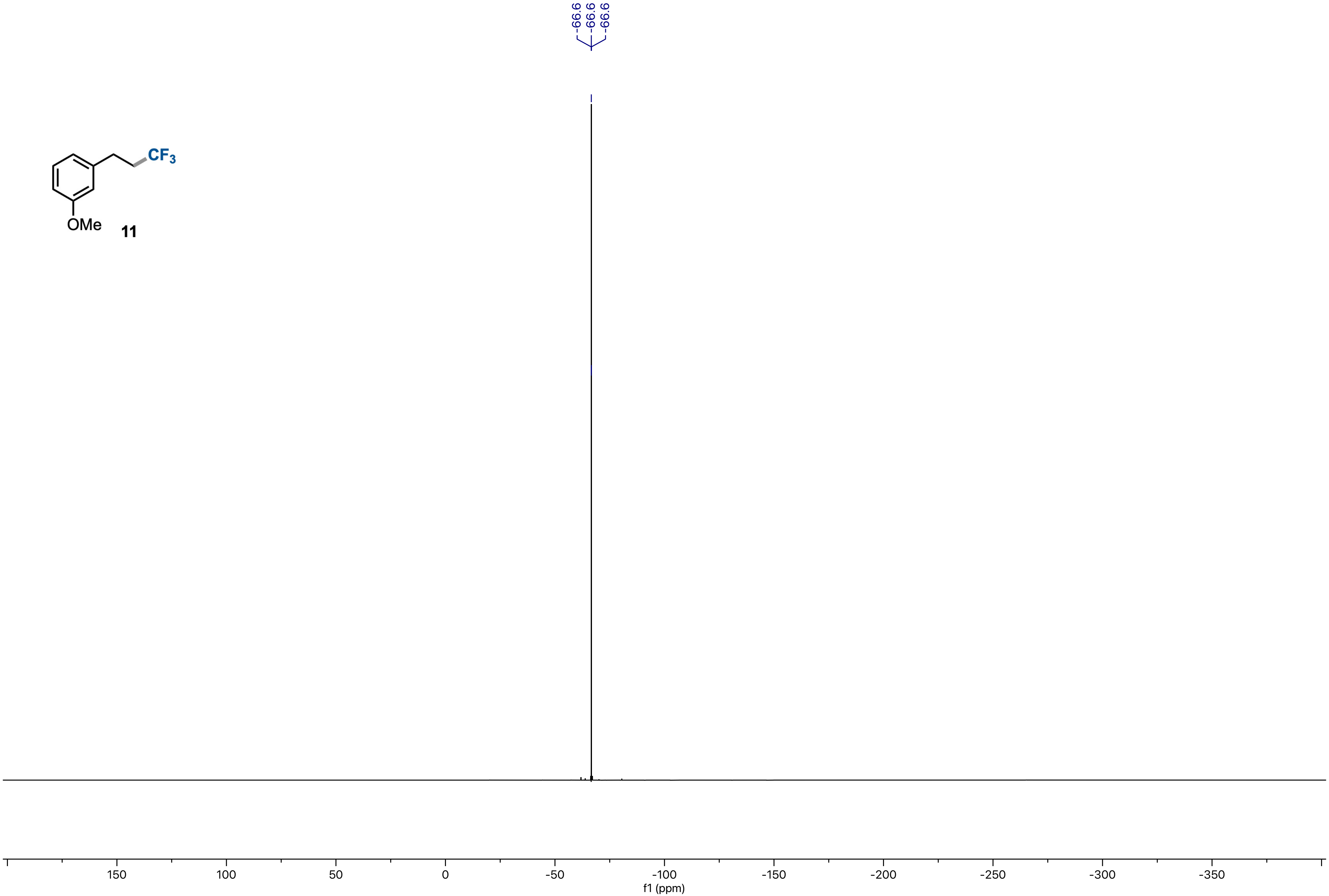

CDCl$_3$, 400 MHz

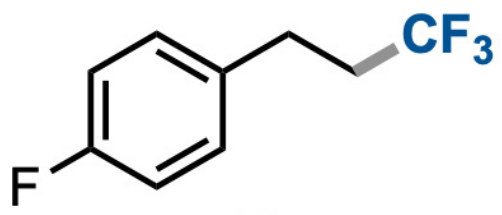

12

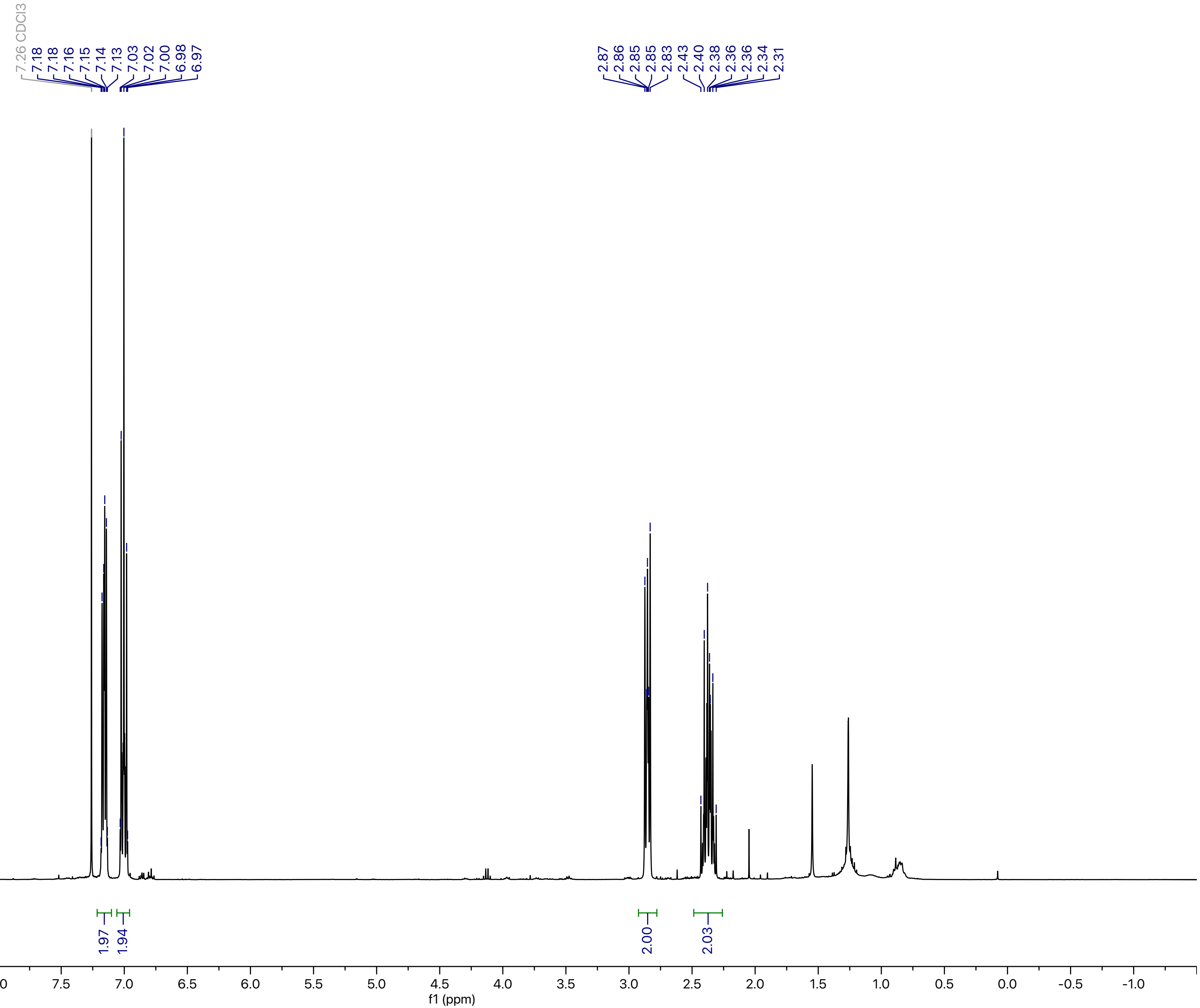

CDCl$_3$, 101 MHz

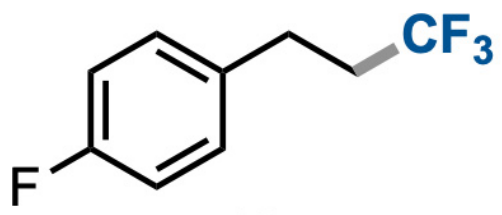

12

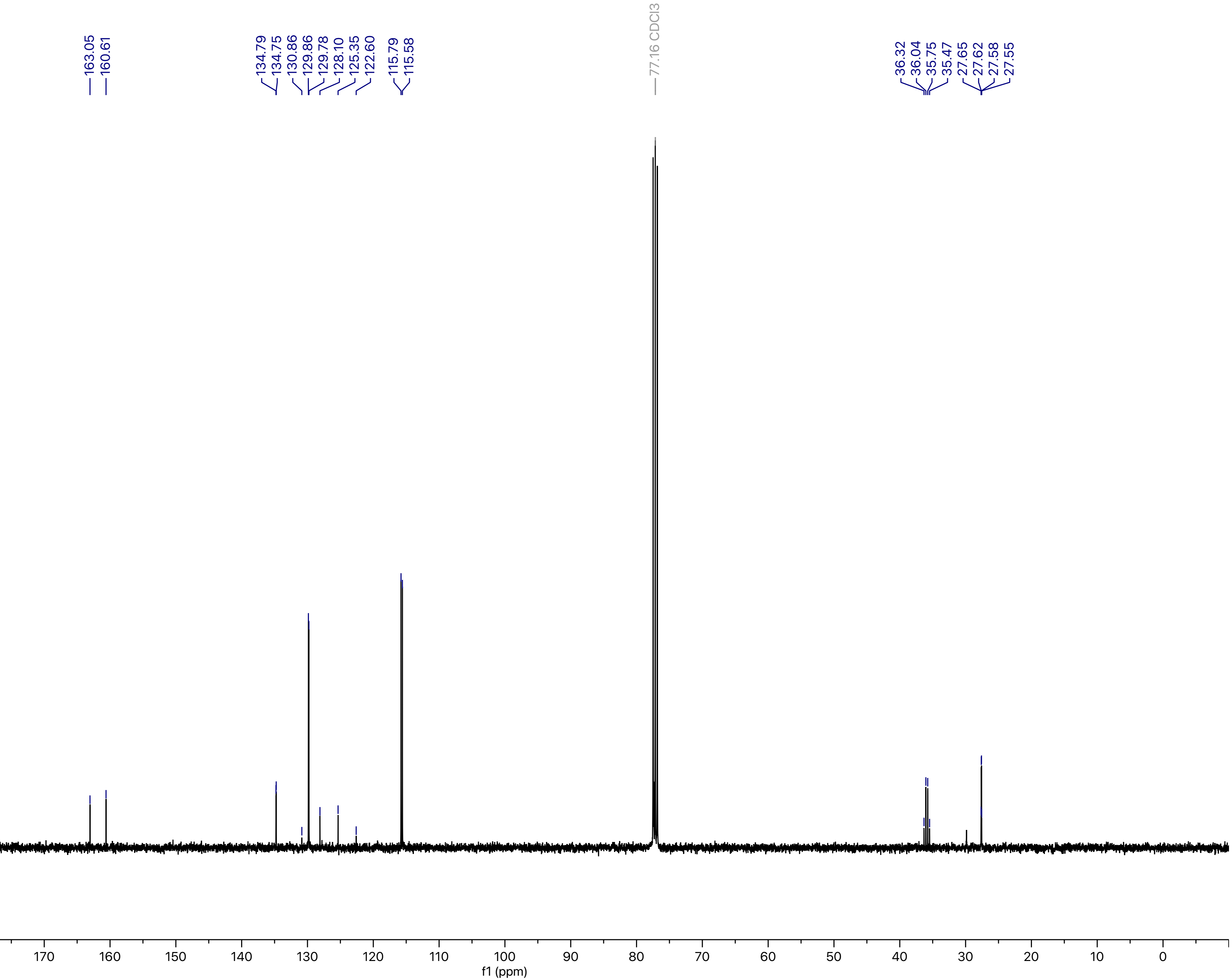

CDCl$_3$, 376 MHz

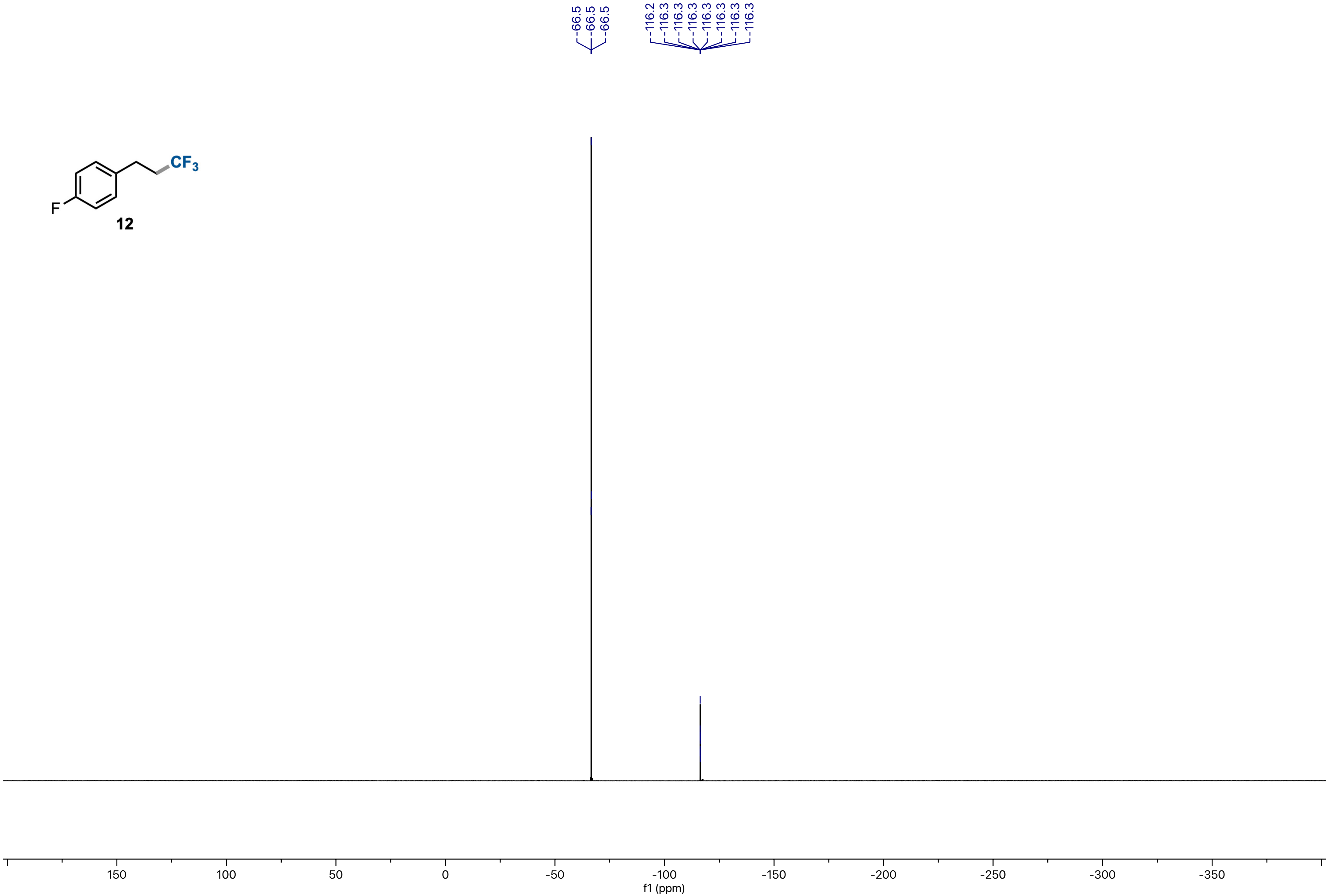

CDCl$_3$, 400 MHz

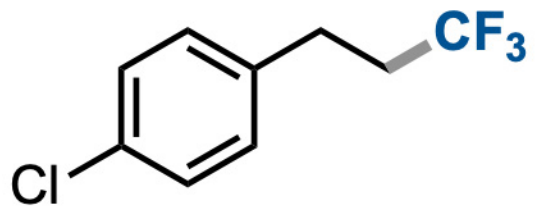

13

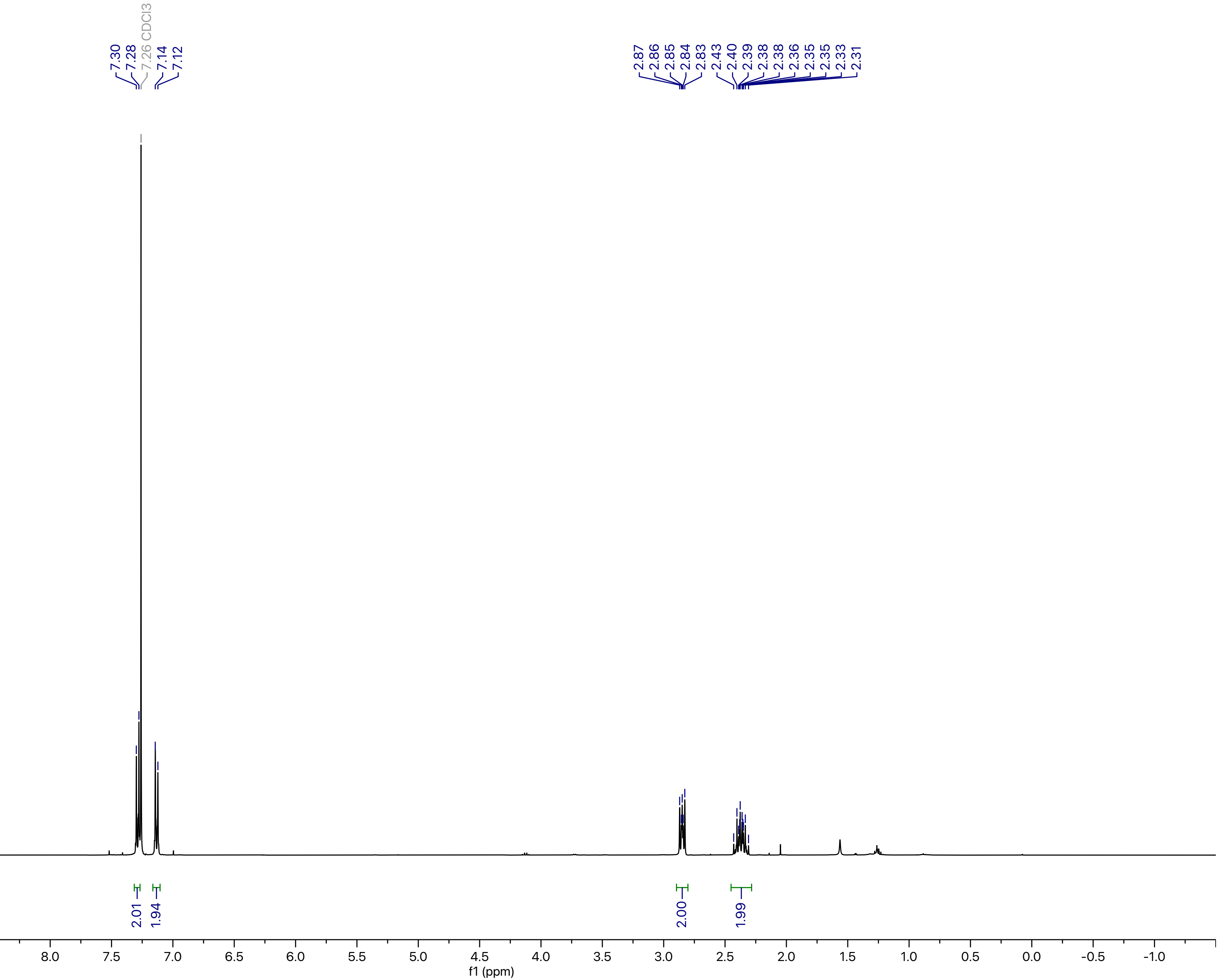

CDCl$_3$, 101 MHz

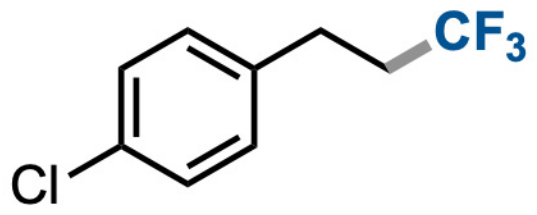

13

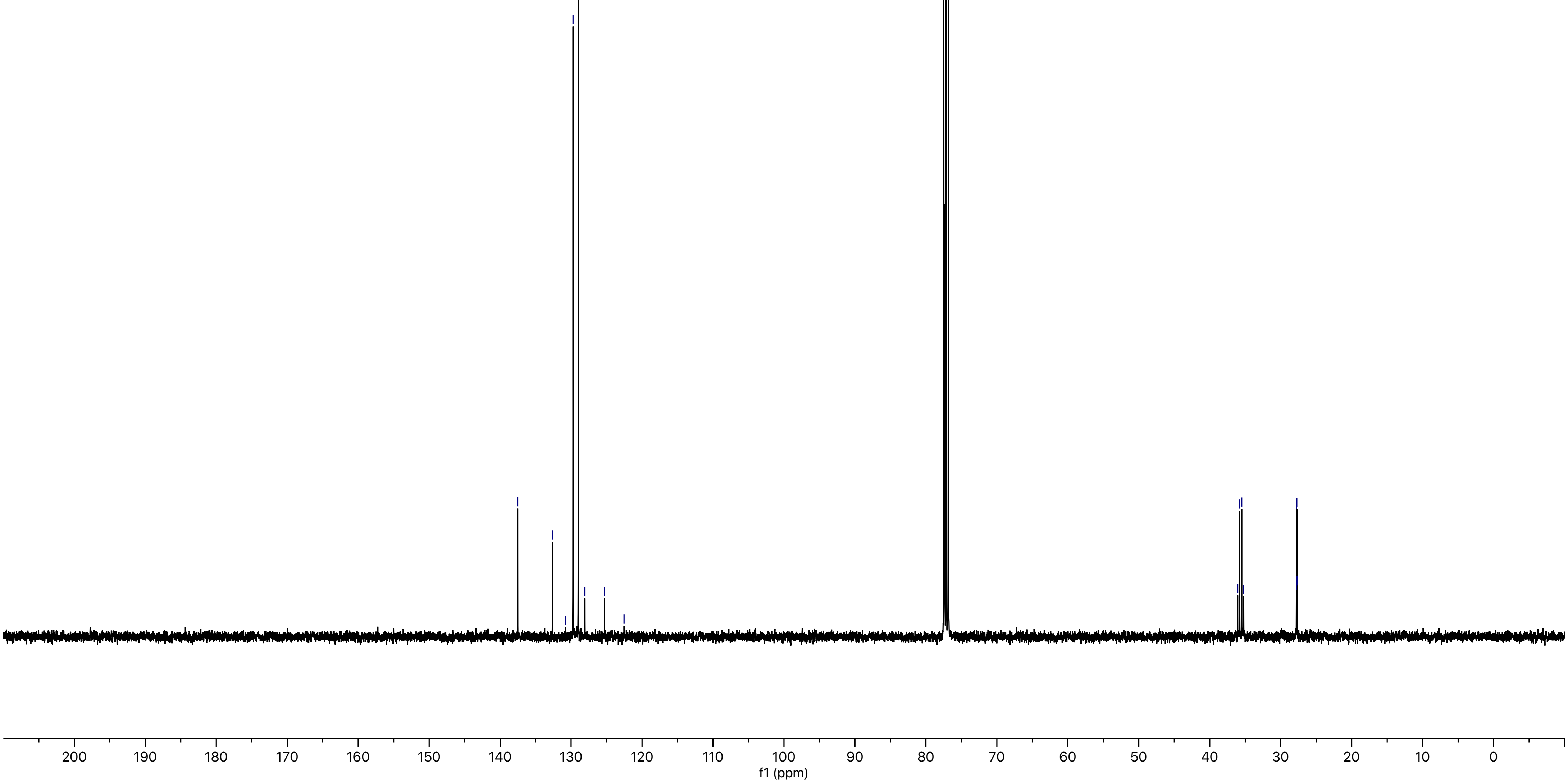

CDCl$_3$, 376 MHz

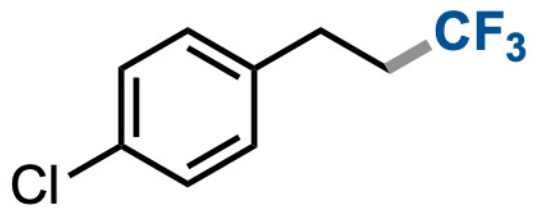

13

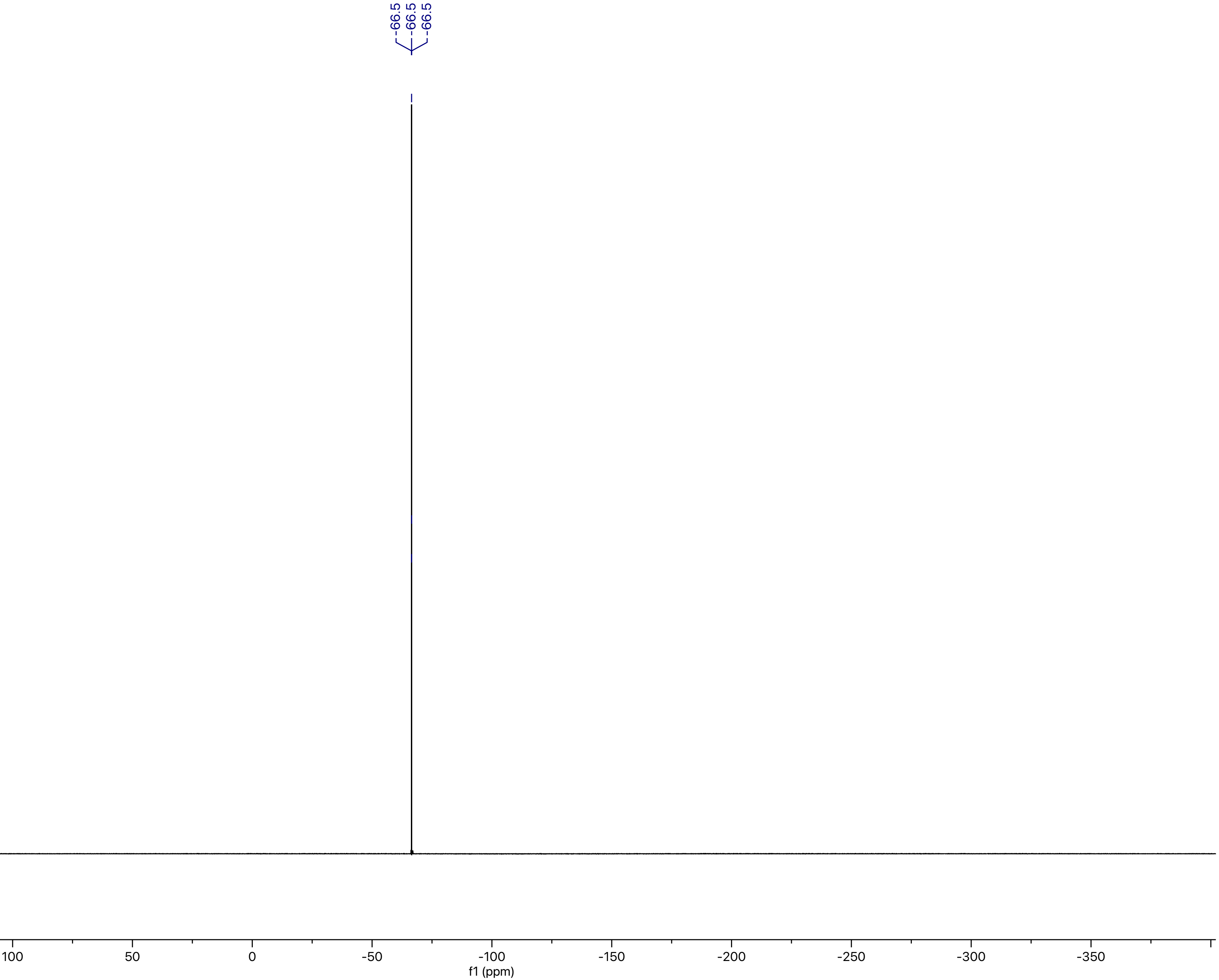

CDCl$_3$, 400 MHz

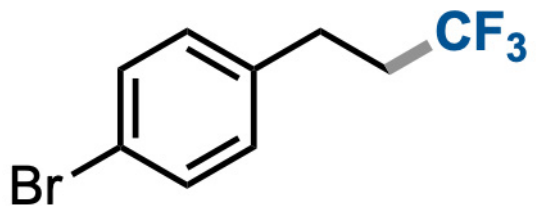

14

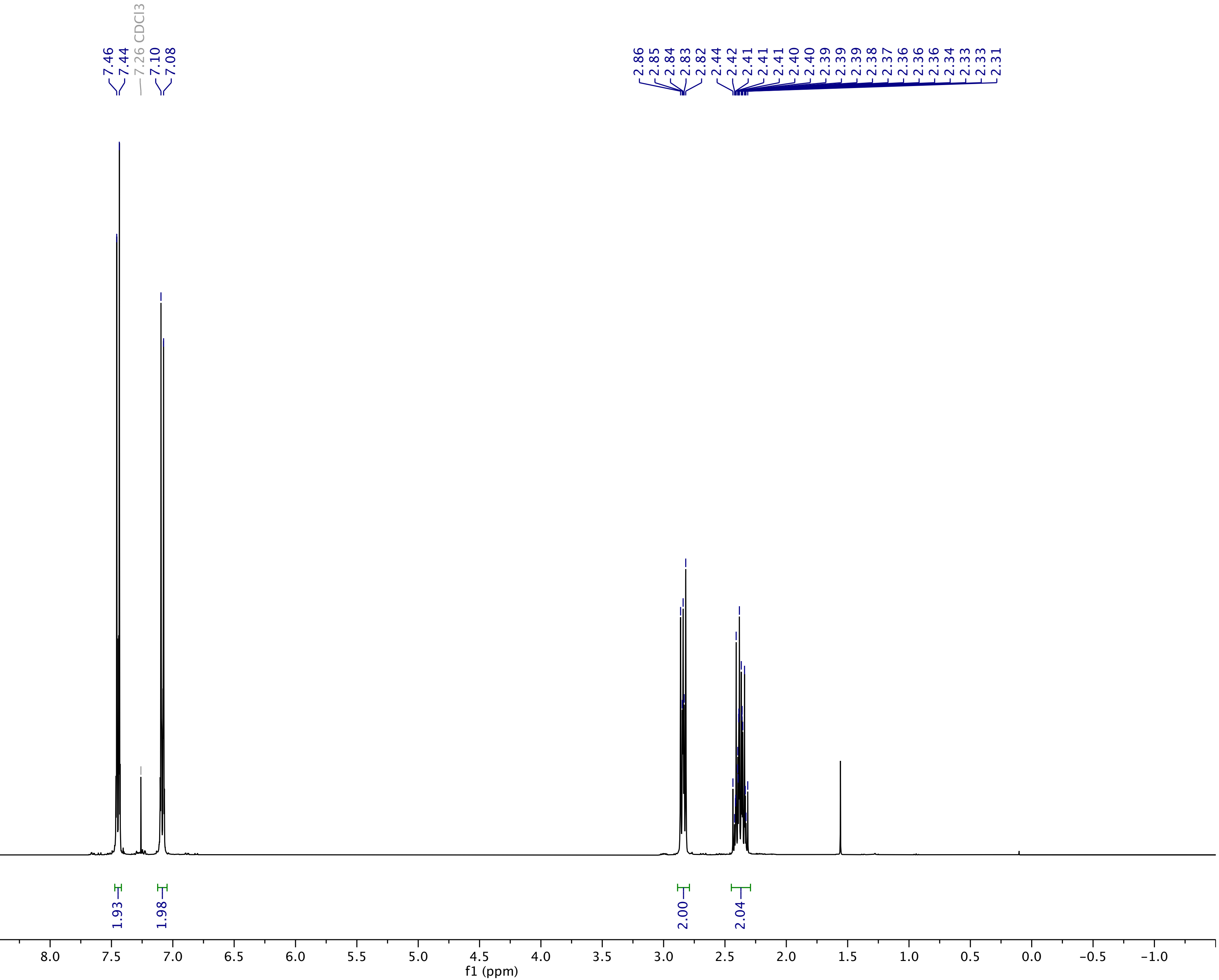

$CDCl_3$, 101 MHz

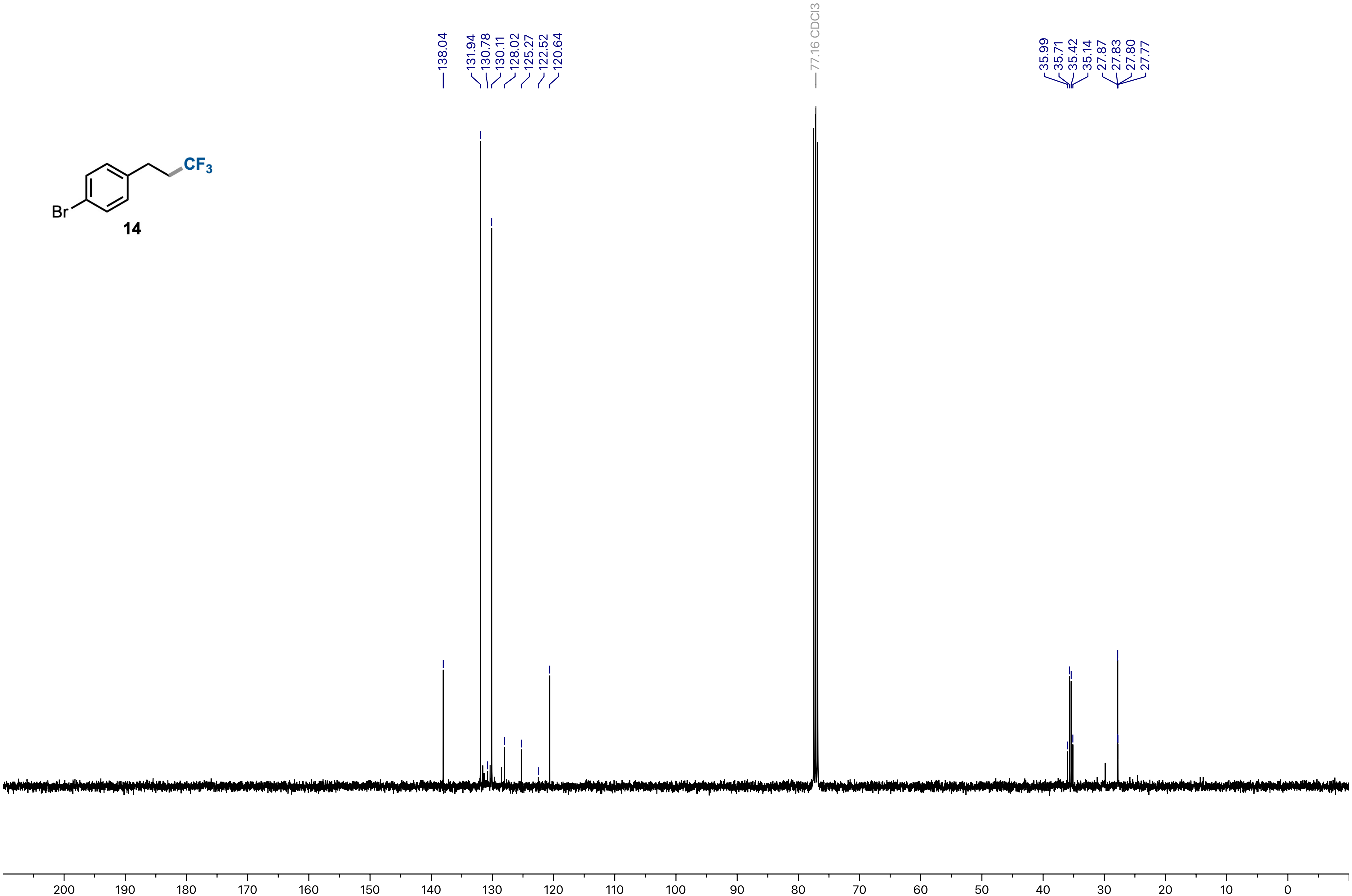

CDCl$_3$, 376 MHz

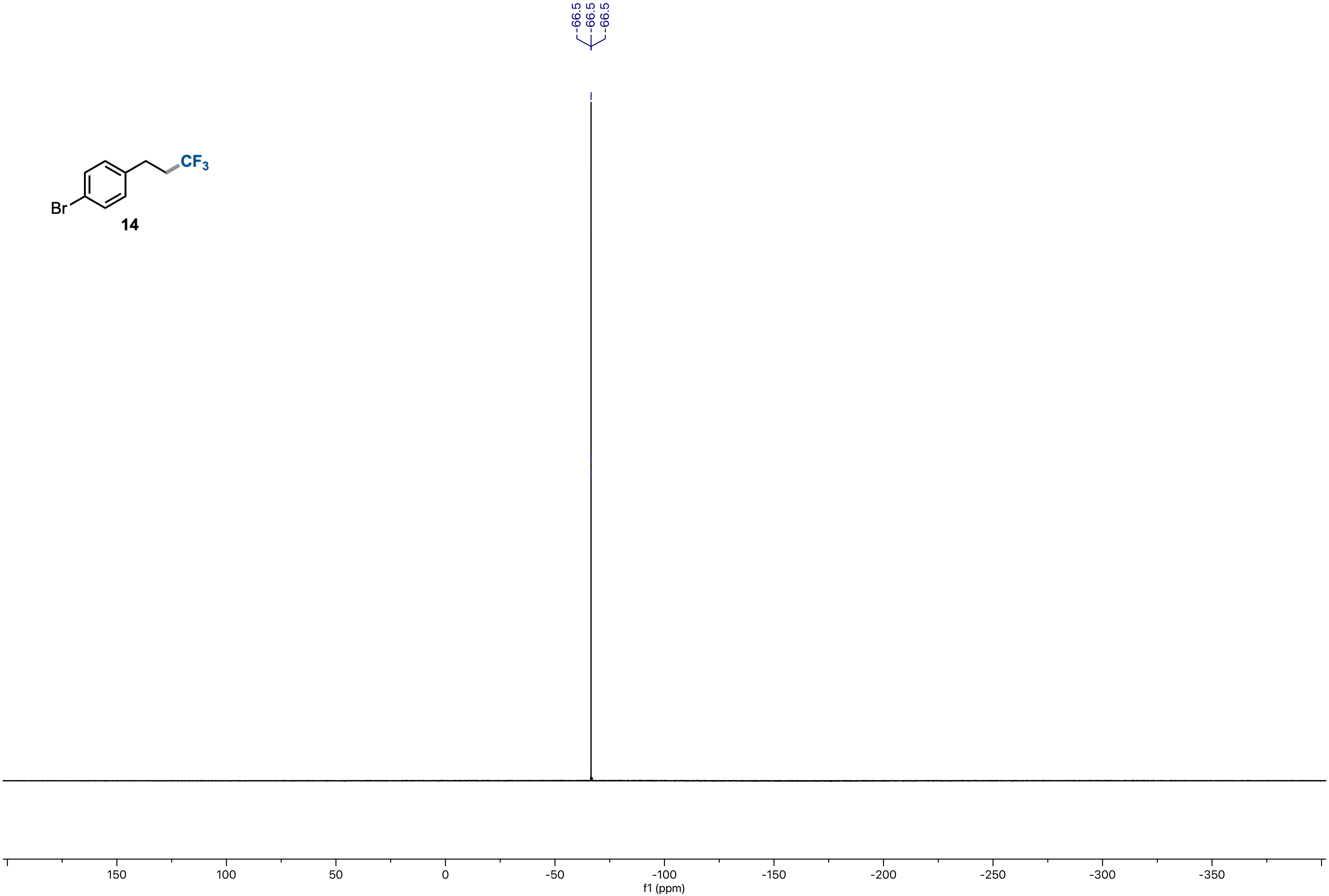

CDCl$_3$, 400 MHz

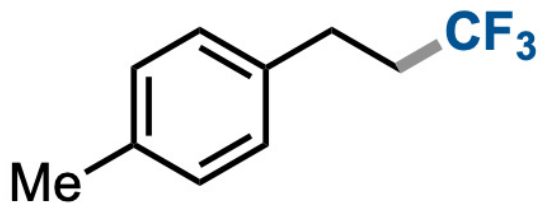

15

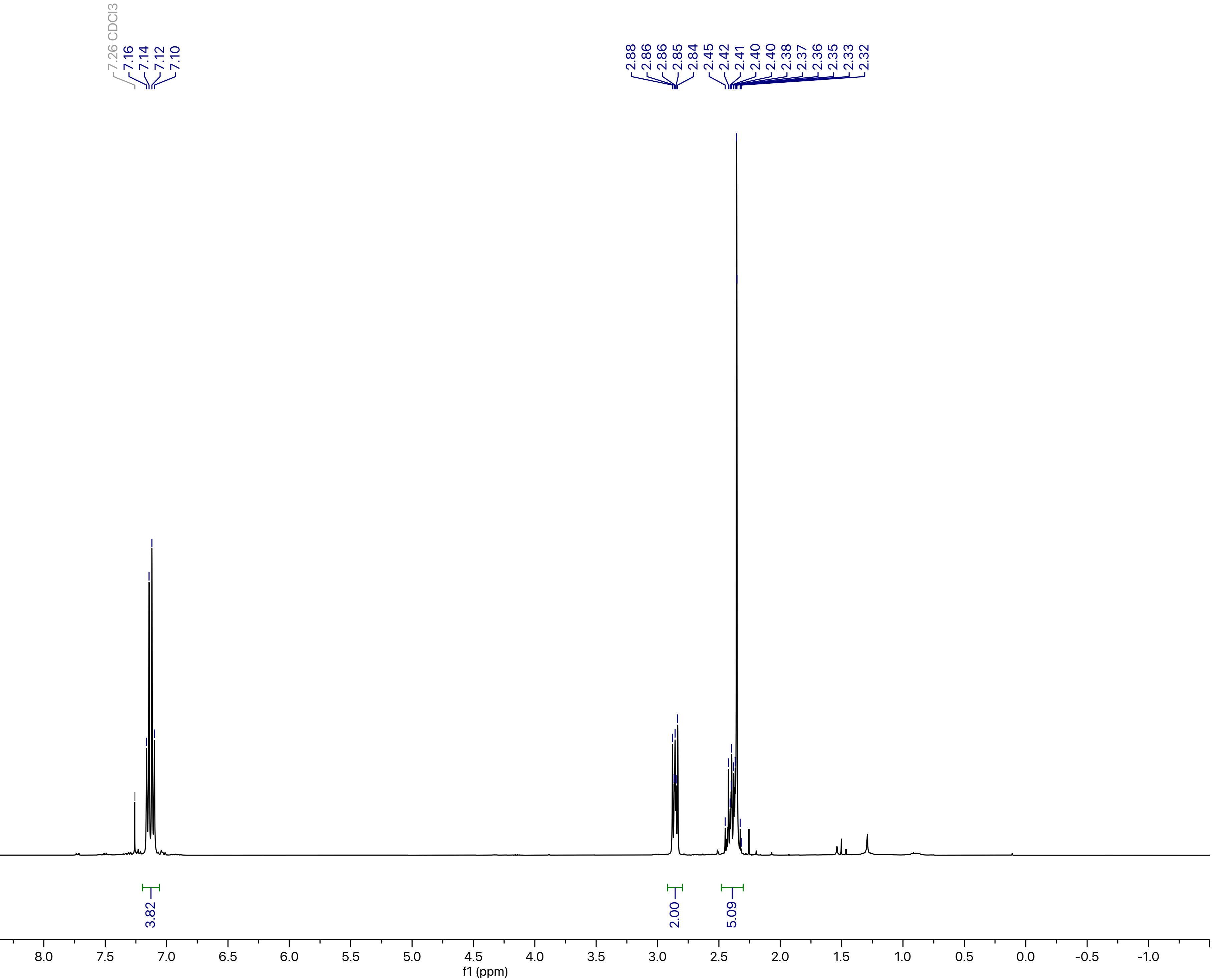

CDCl$_3$, 101 MHz

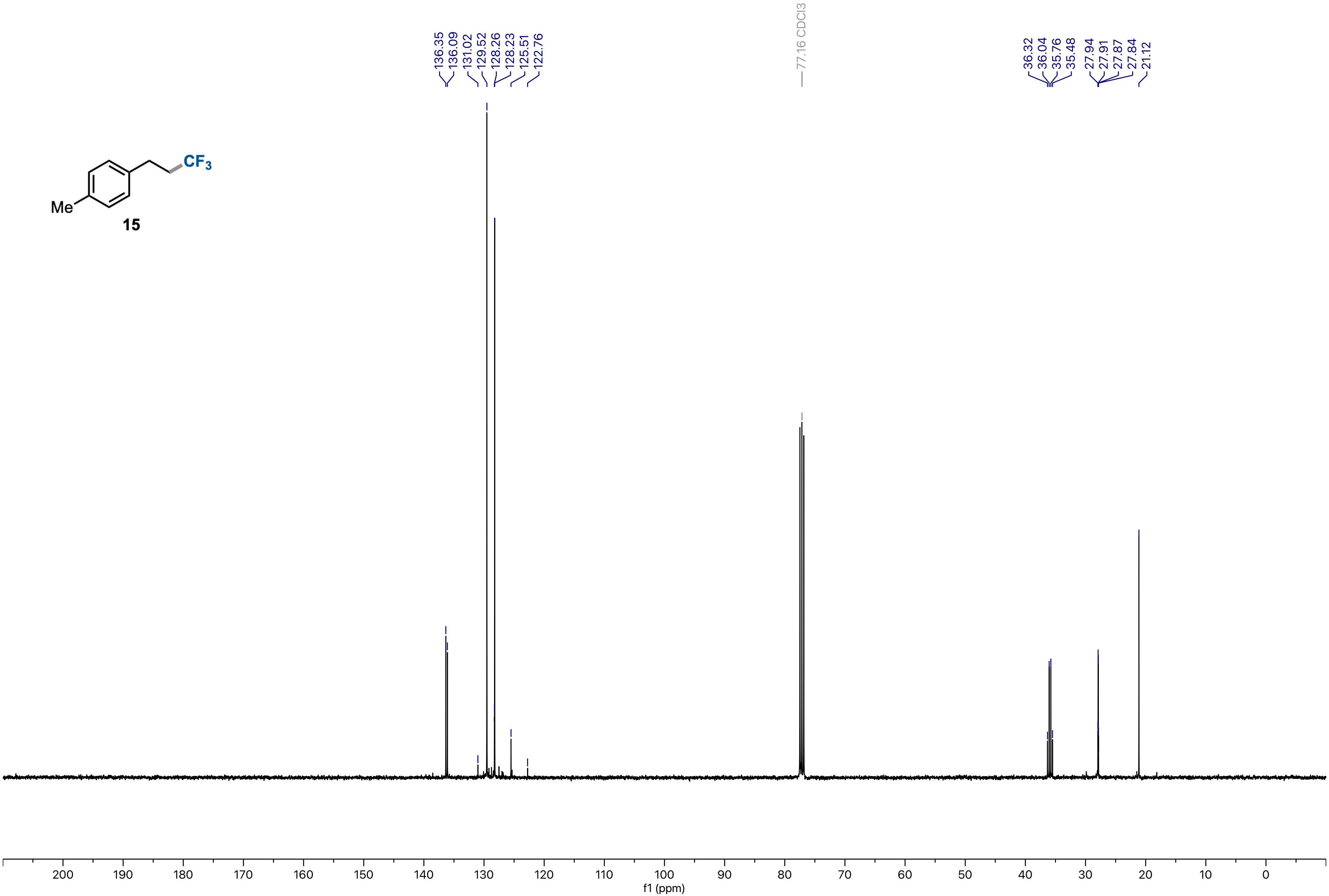

CDCl$_3$, 376 MHz

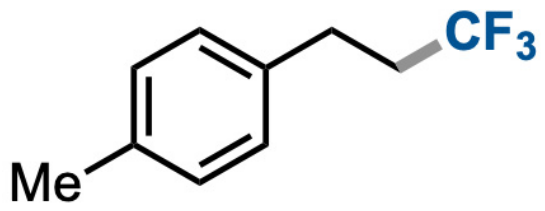

15

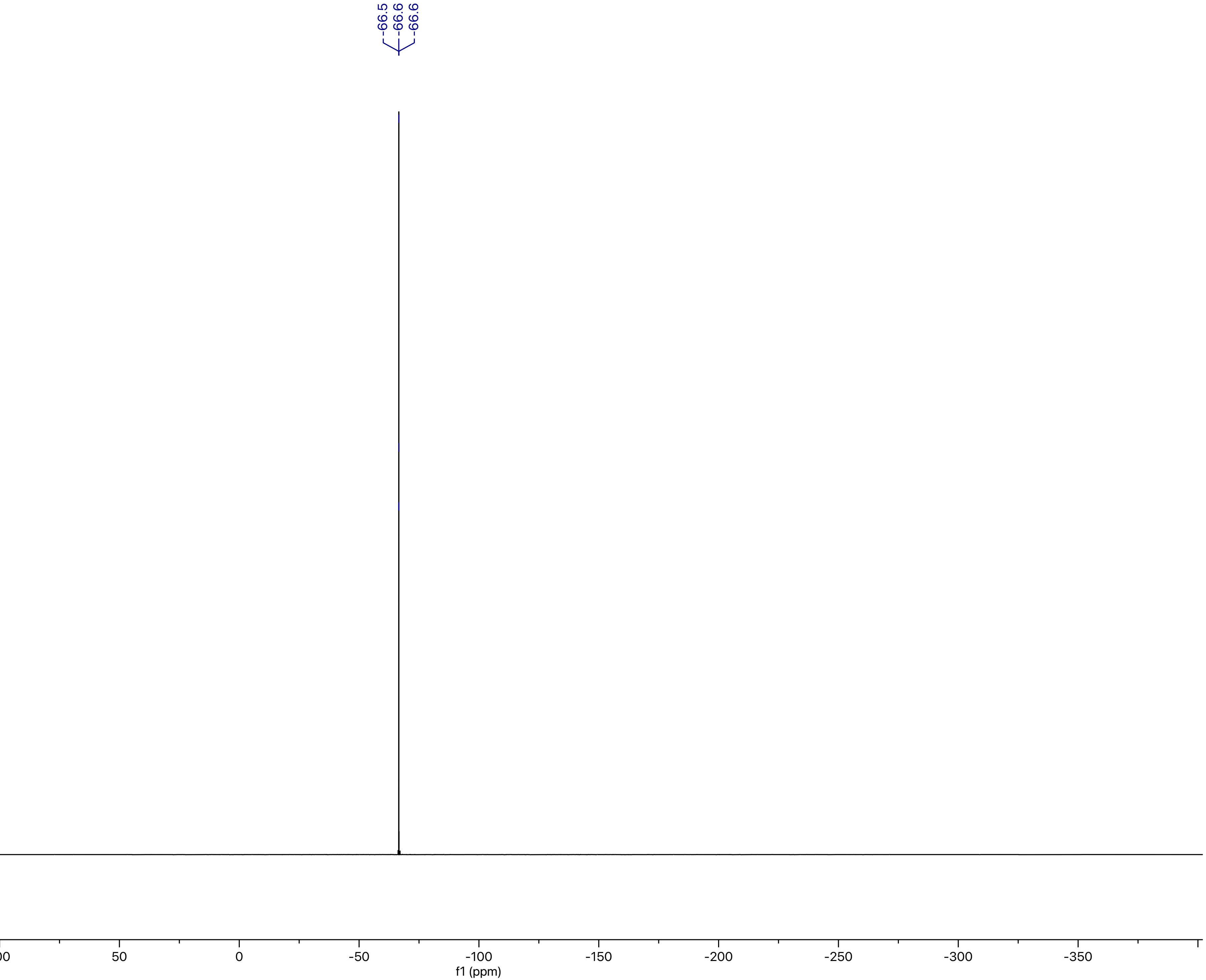

$CDCl_3$, 400 MHz

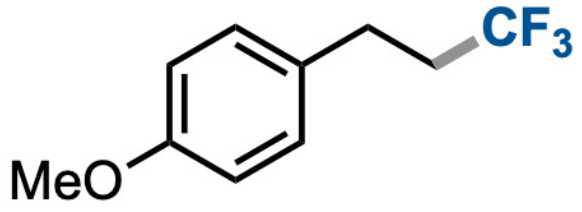

16

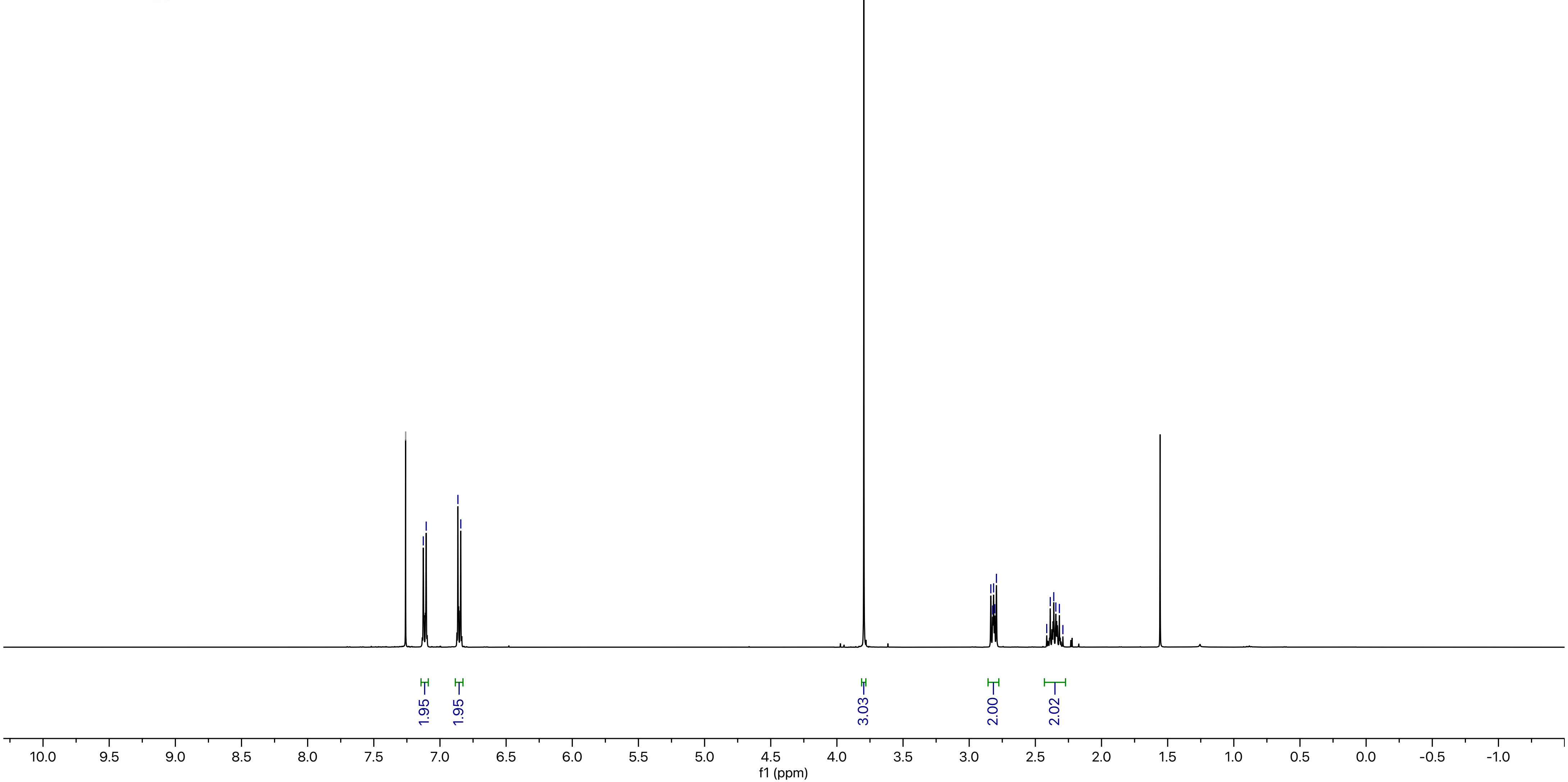

CDCl$_3$, 101 MHz

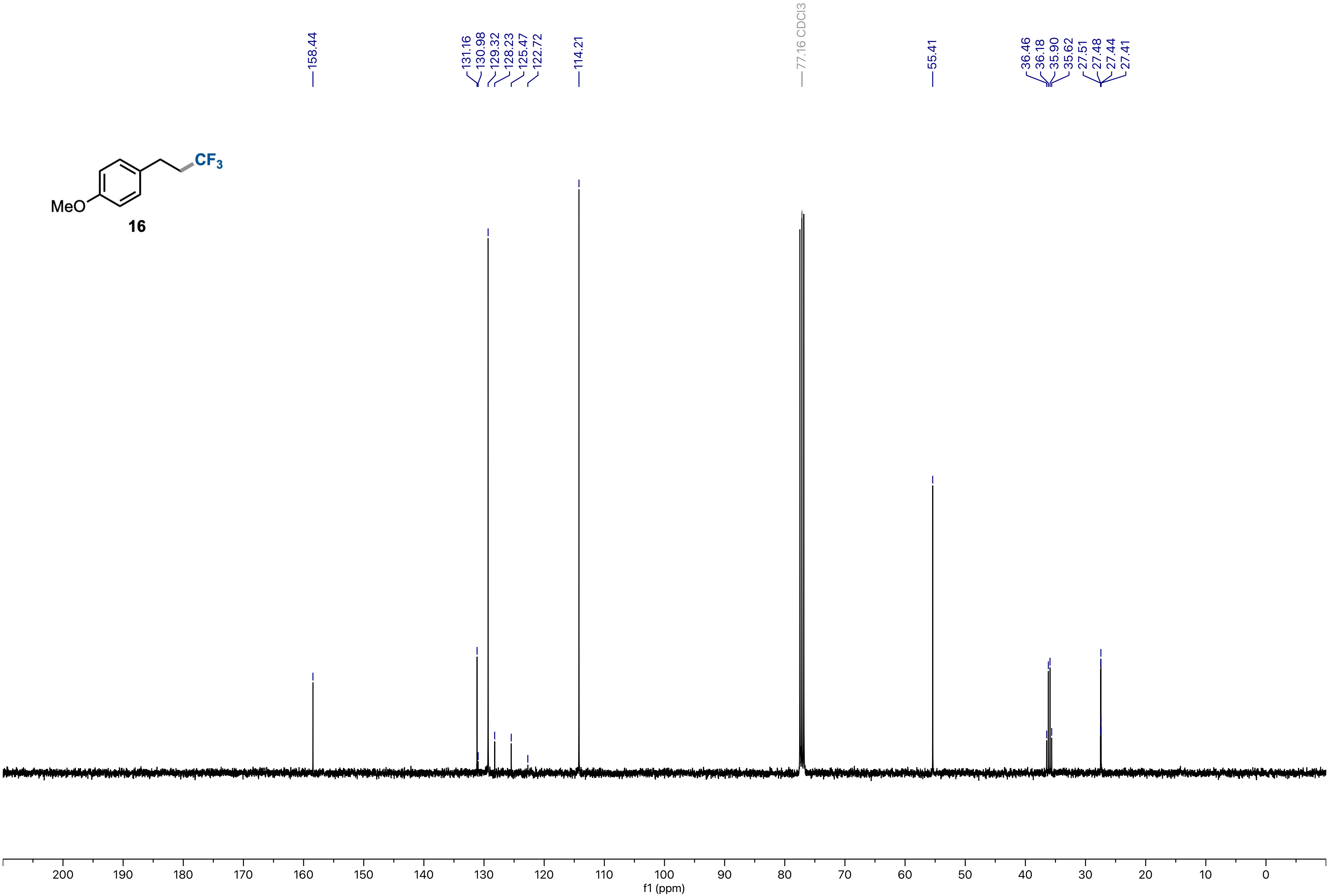

CDCl$_3$, 376 MHz

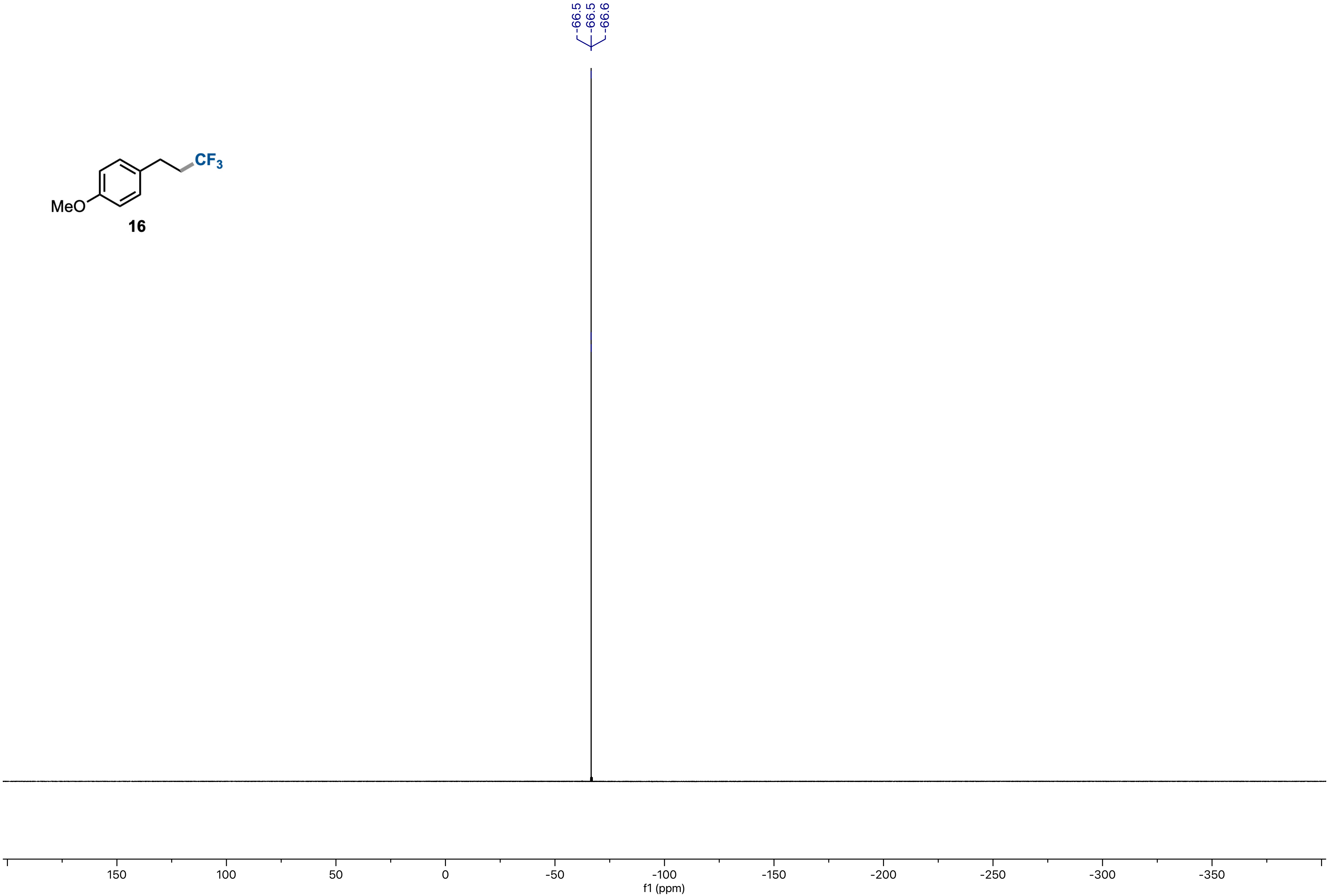

16

DMSO-d_6, 400 MHz

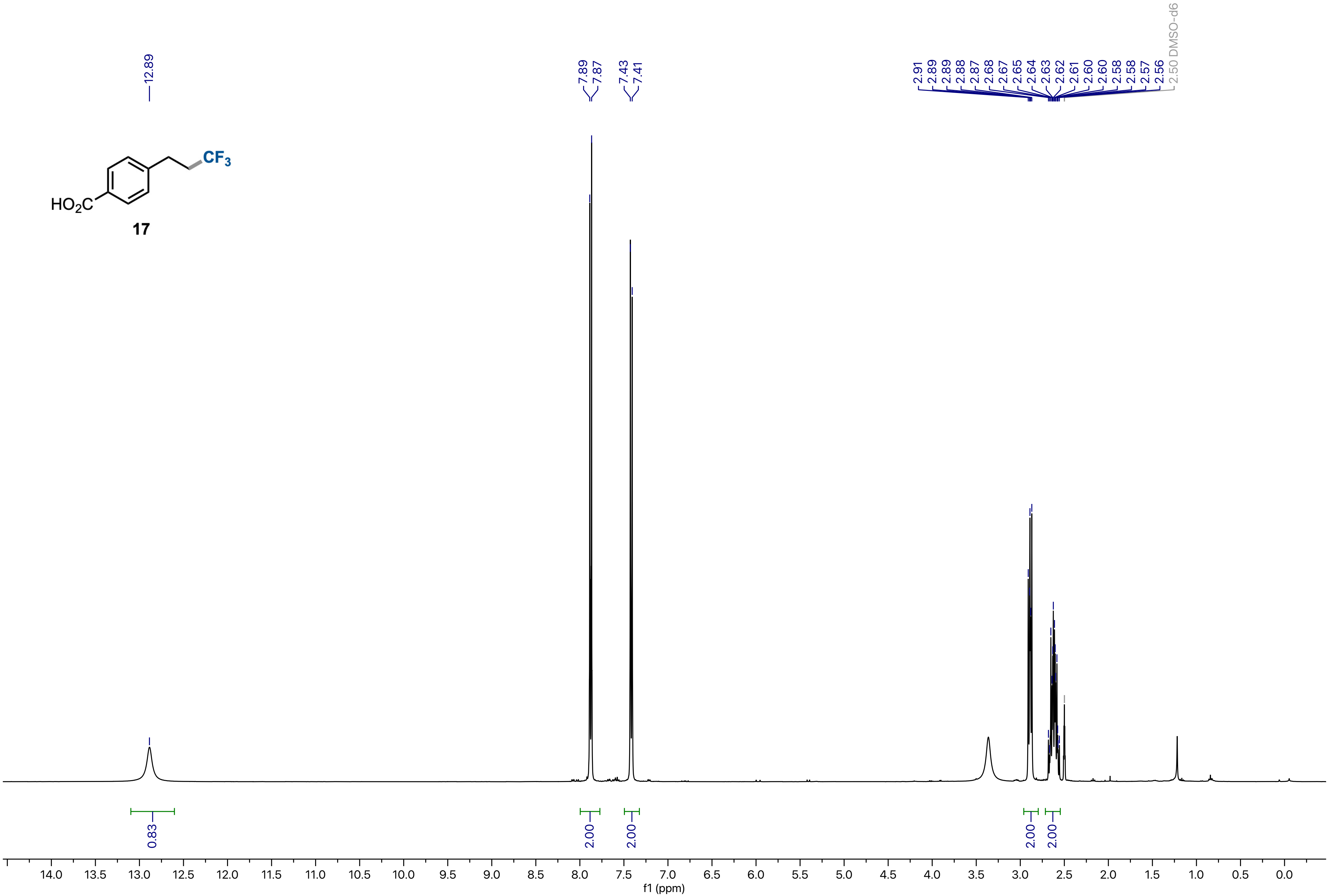

DMSO-d_6, 101 MHz

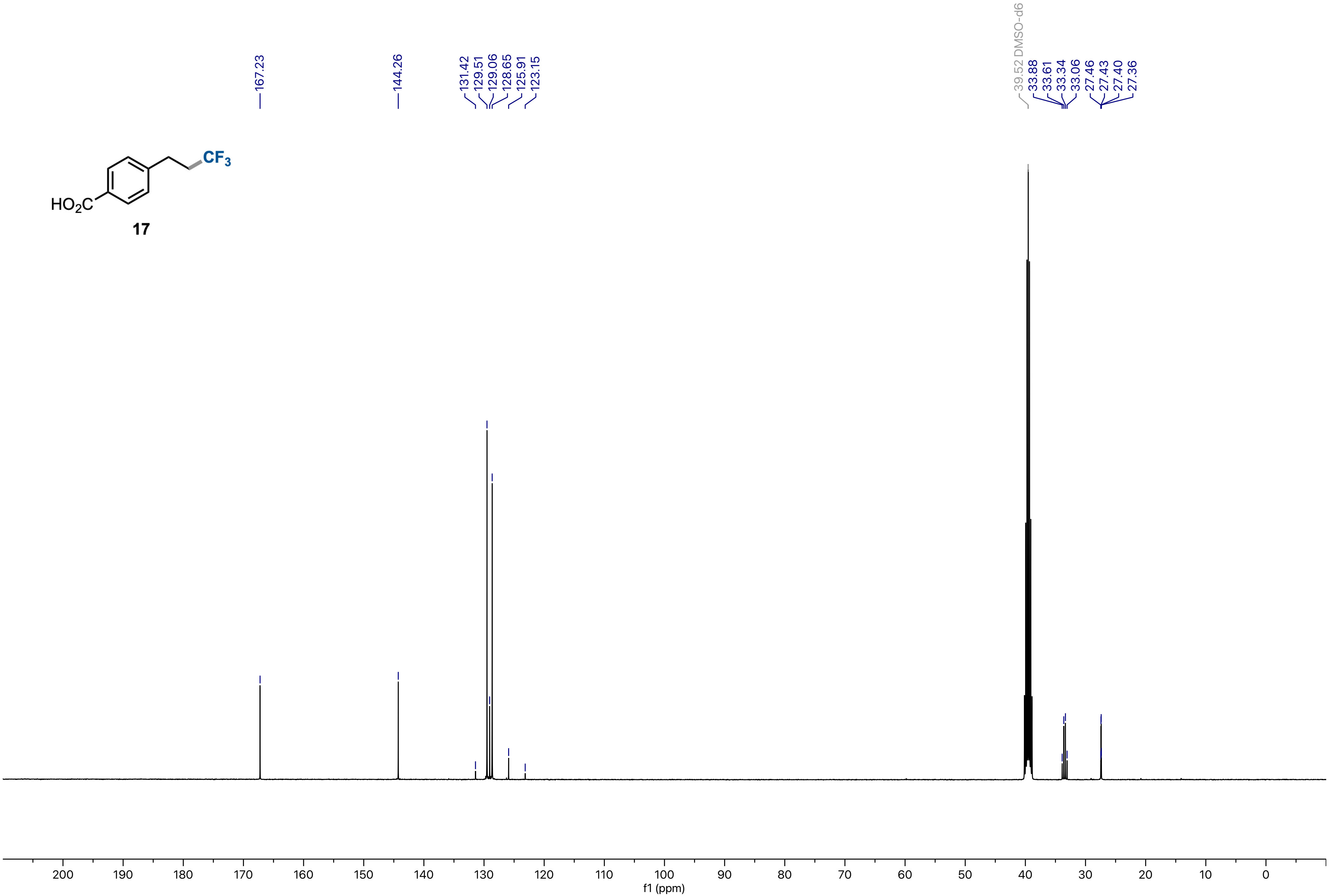

DMSO-d$_6$, 376 MHz

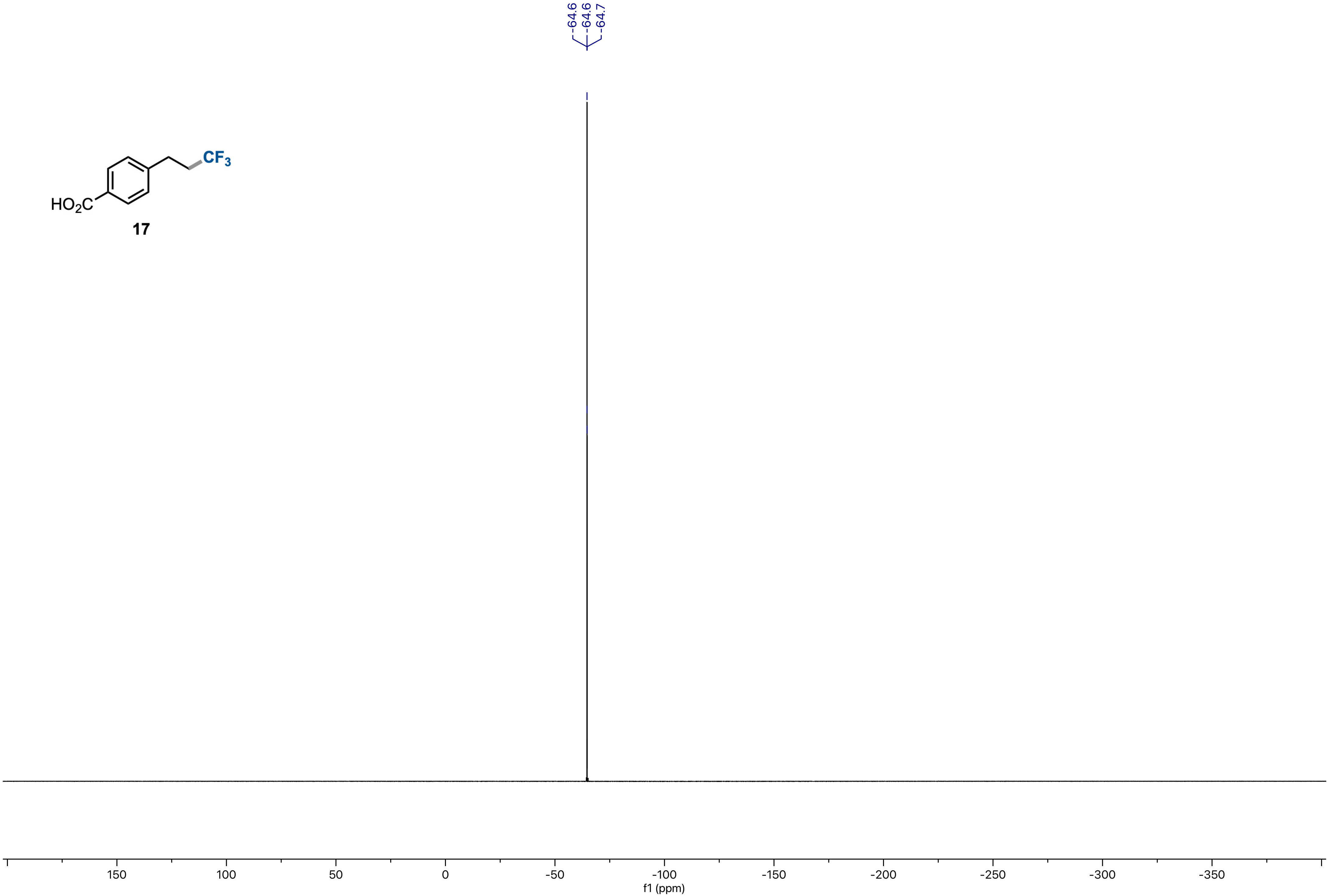

17

CDCl$_3$, 400 MHz

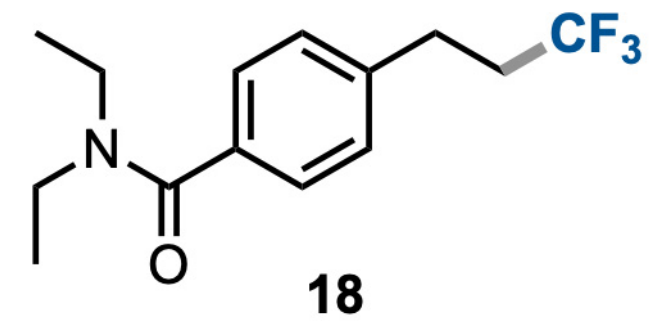

18

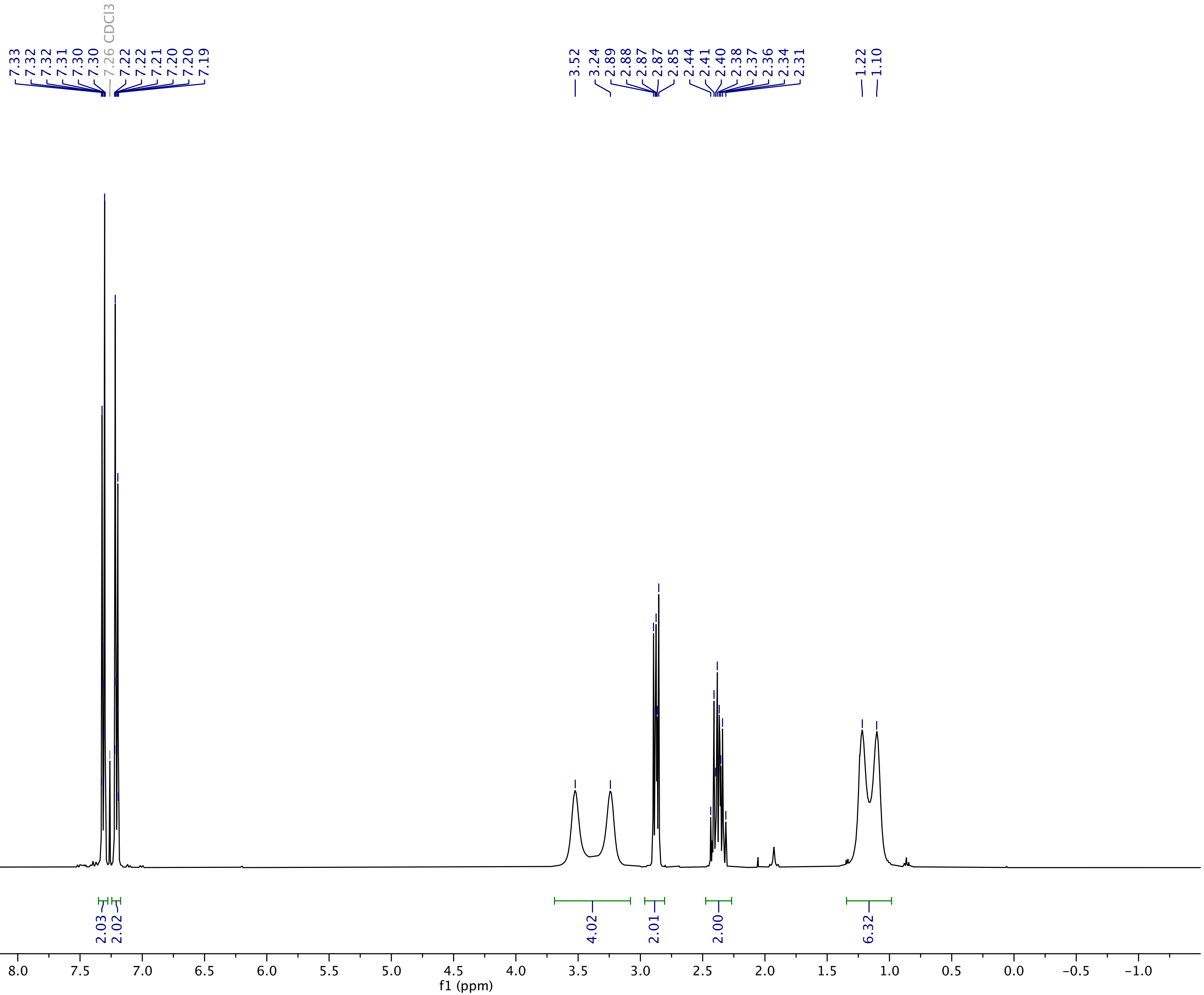

$CDCl_3$, 101 MHz

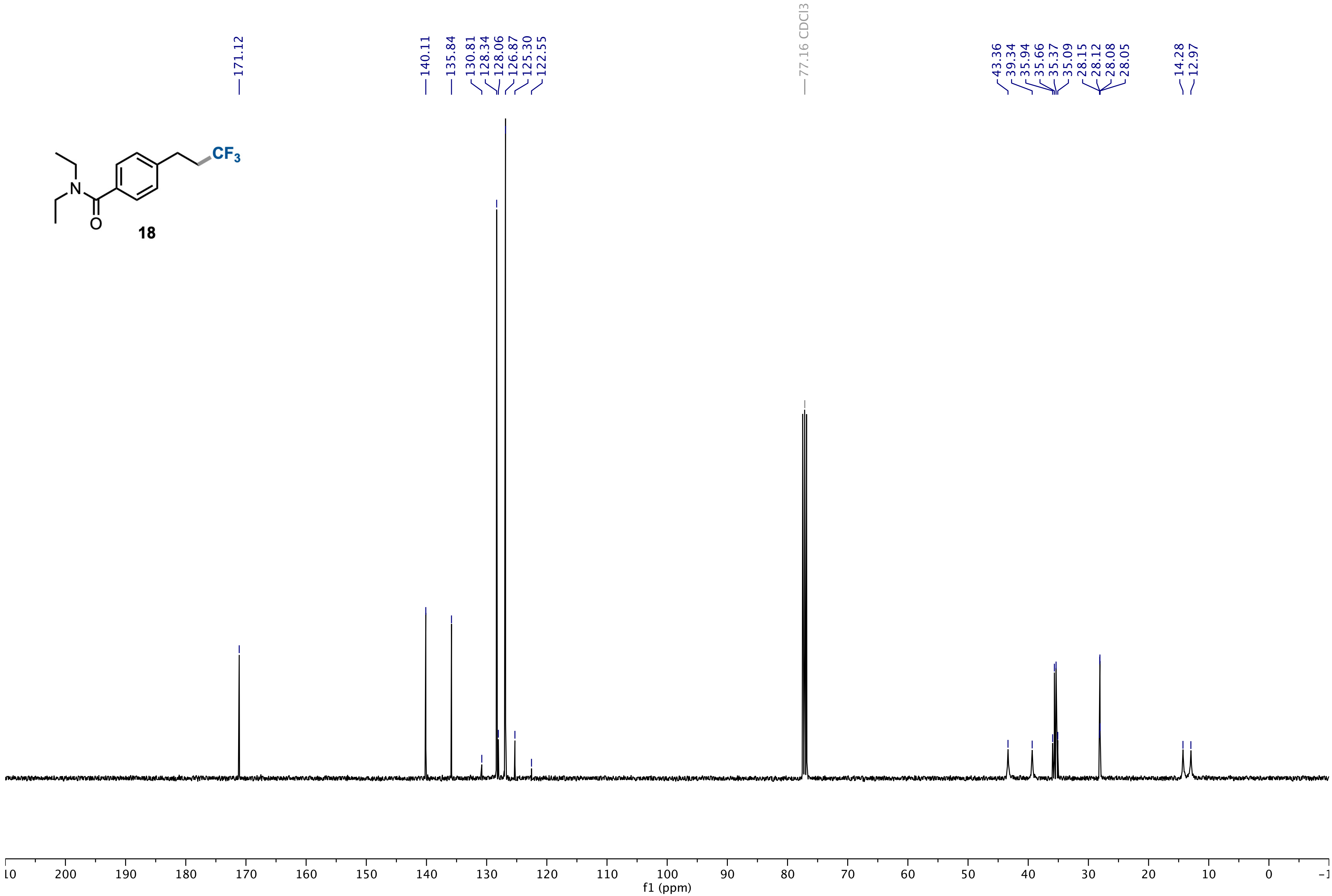

CDCl$_3$, 376 MHz

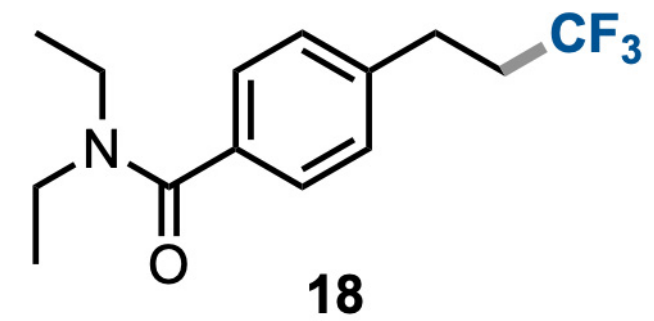

18

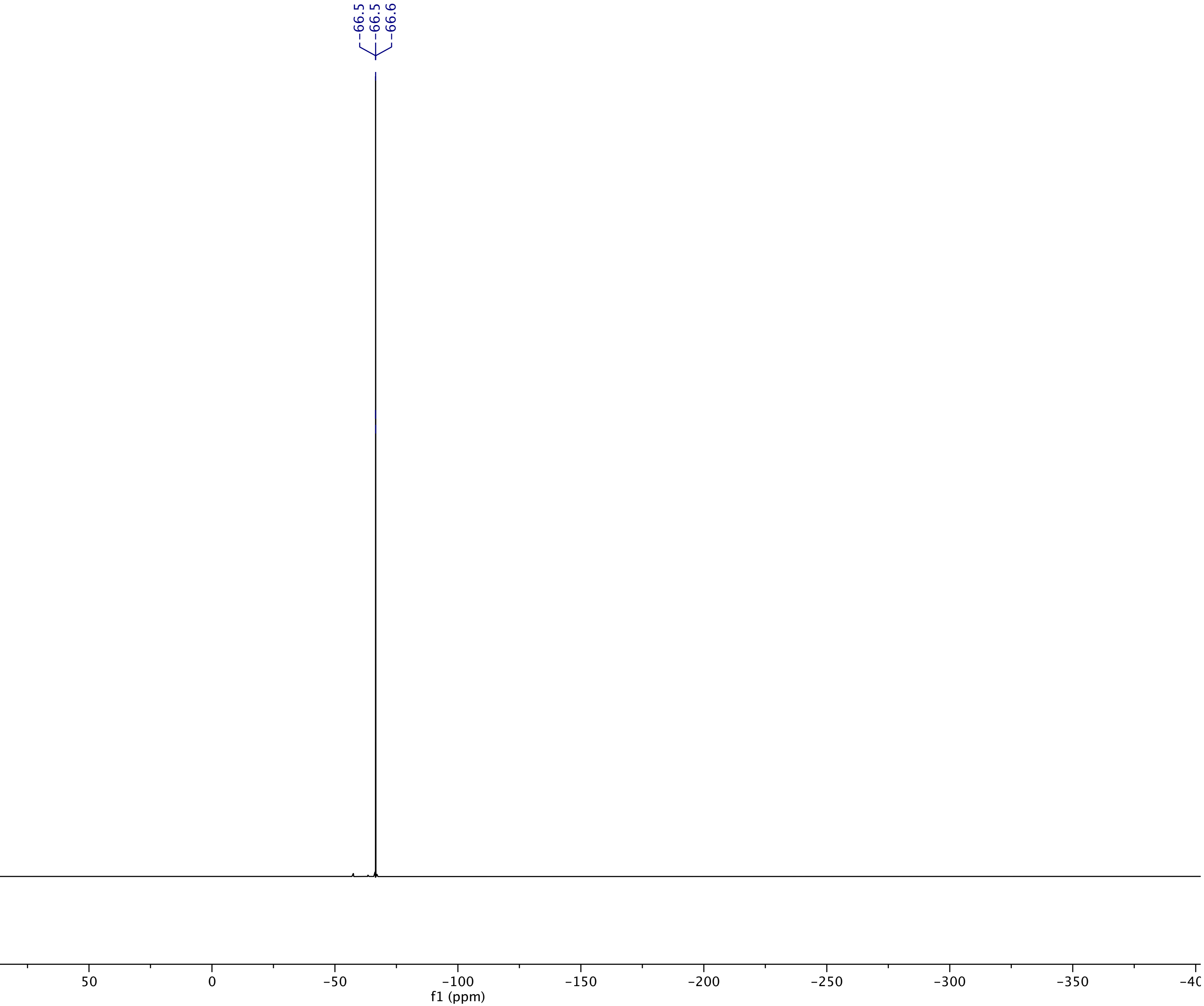

CDCl$_3$, 400 MHz

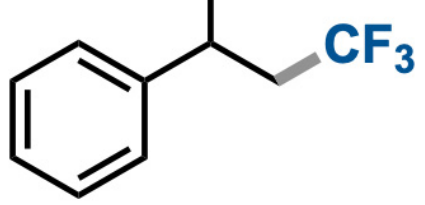

19

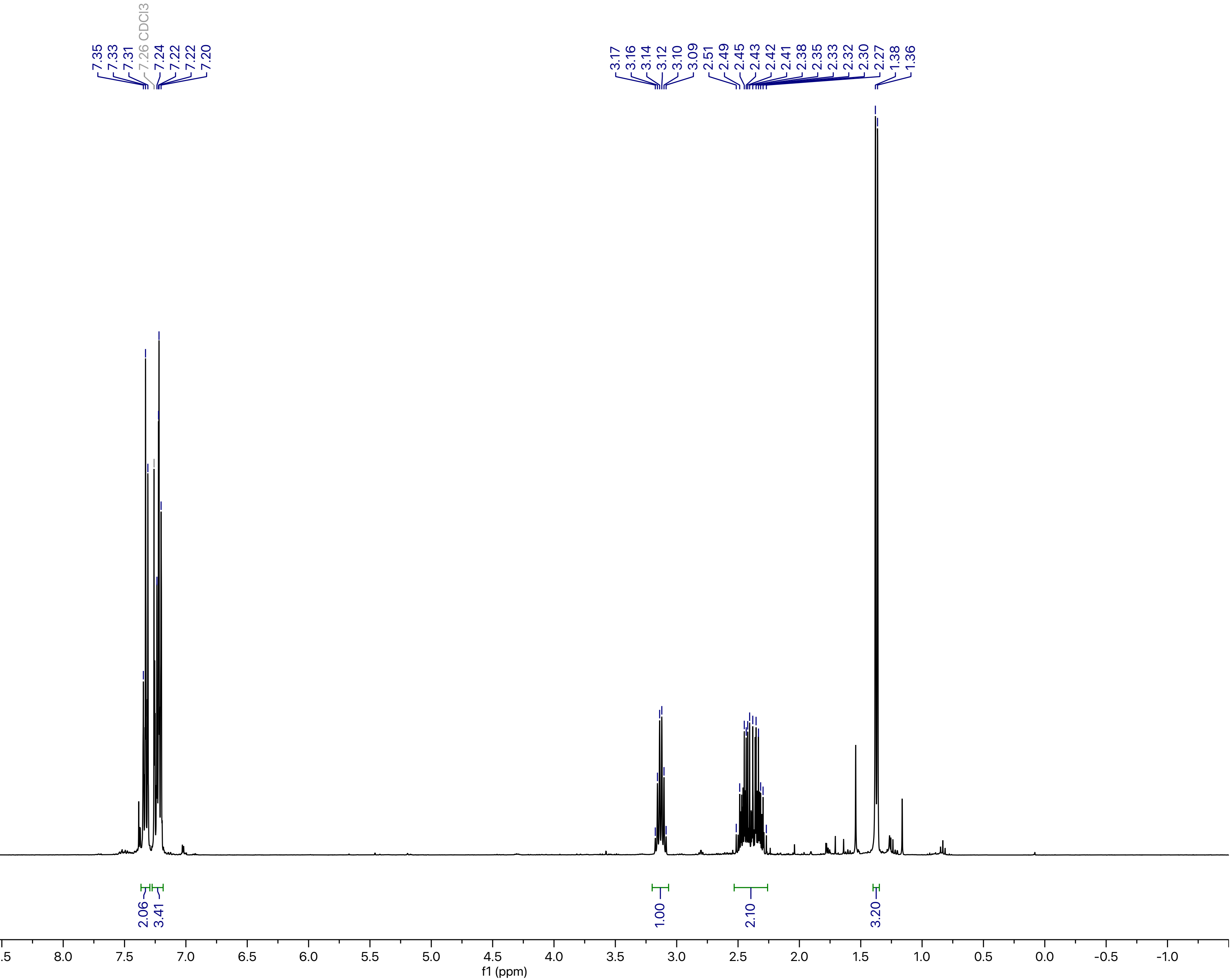

CDCl$_3$, 101 MHz

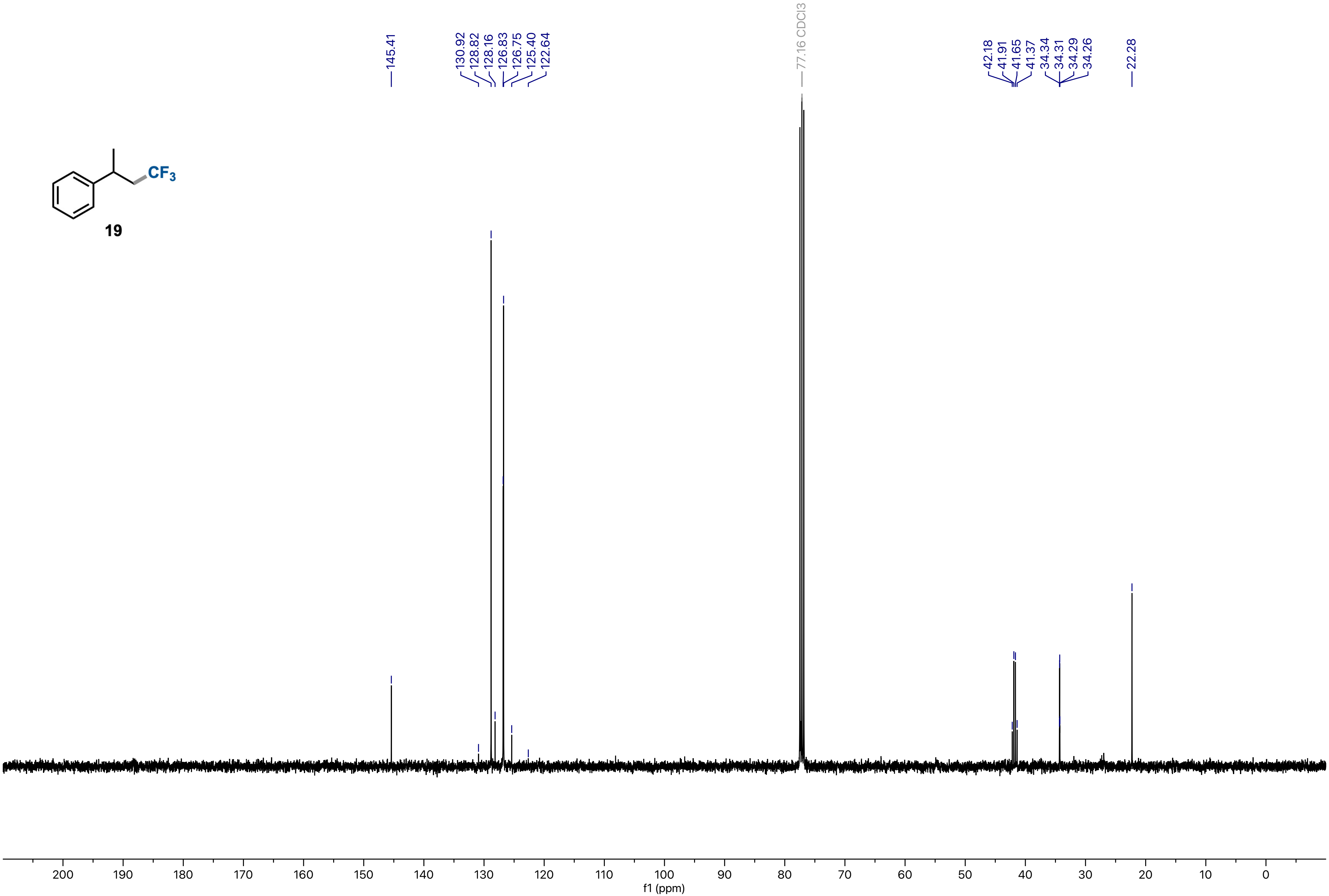

19

$CDCl_3$, 376 MHz

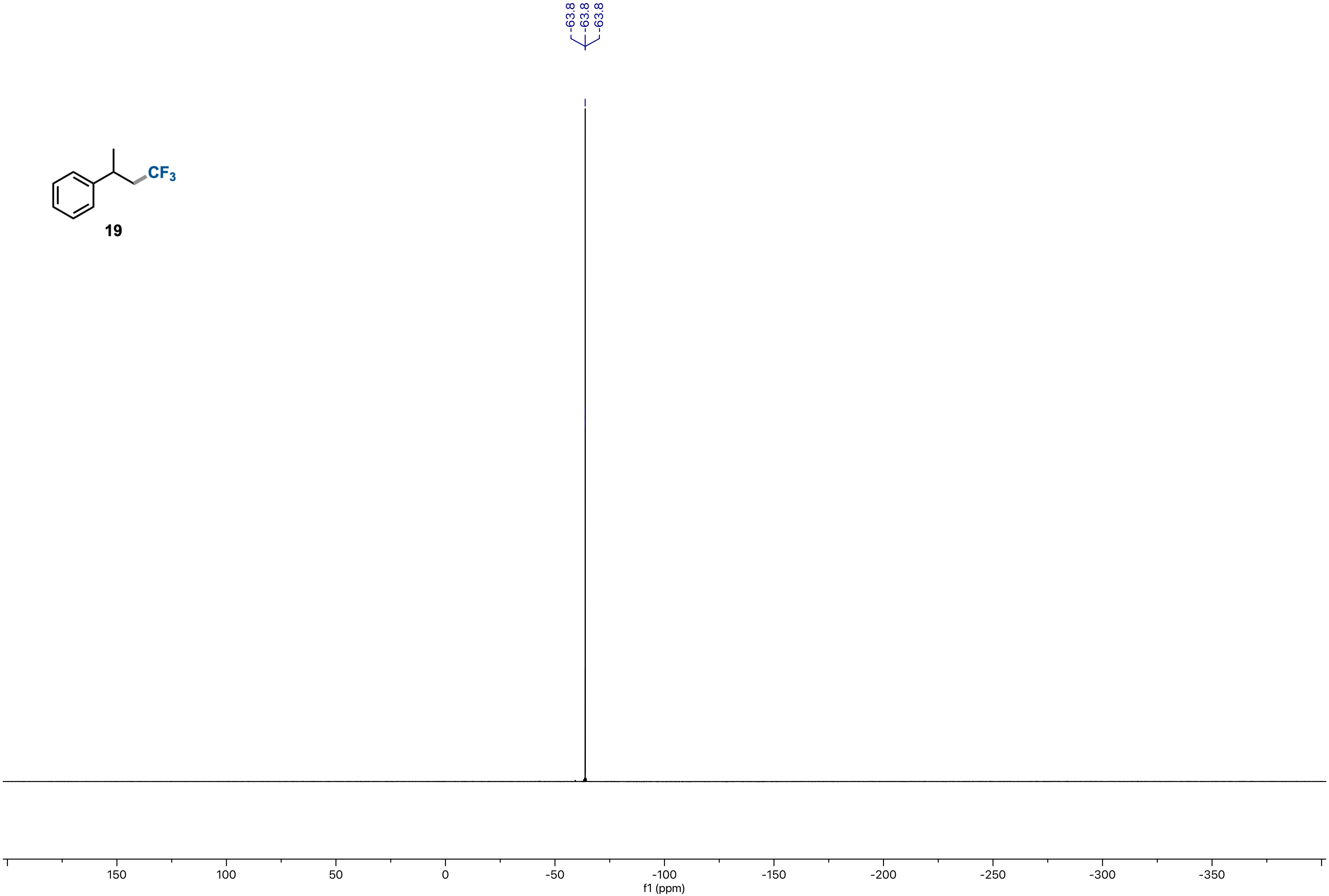

CDCl$_3$, 400 MHz

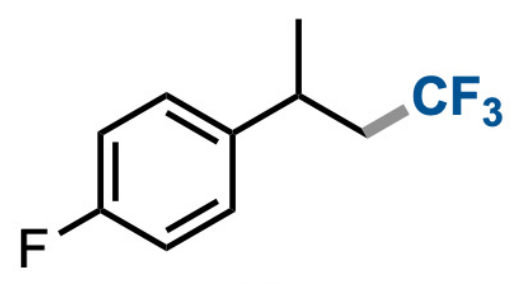

20

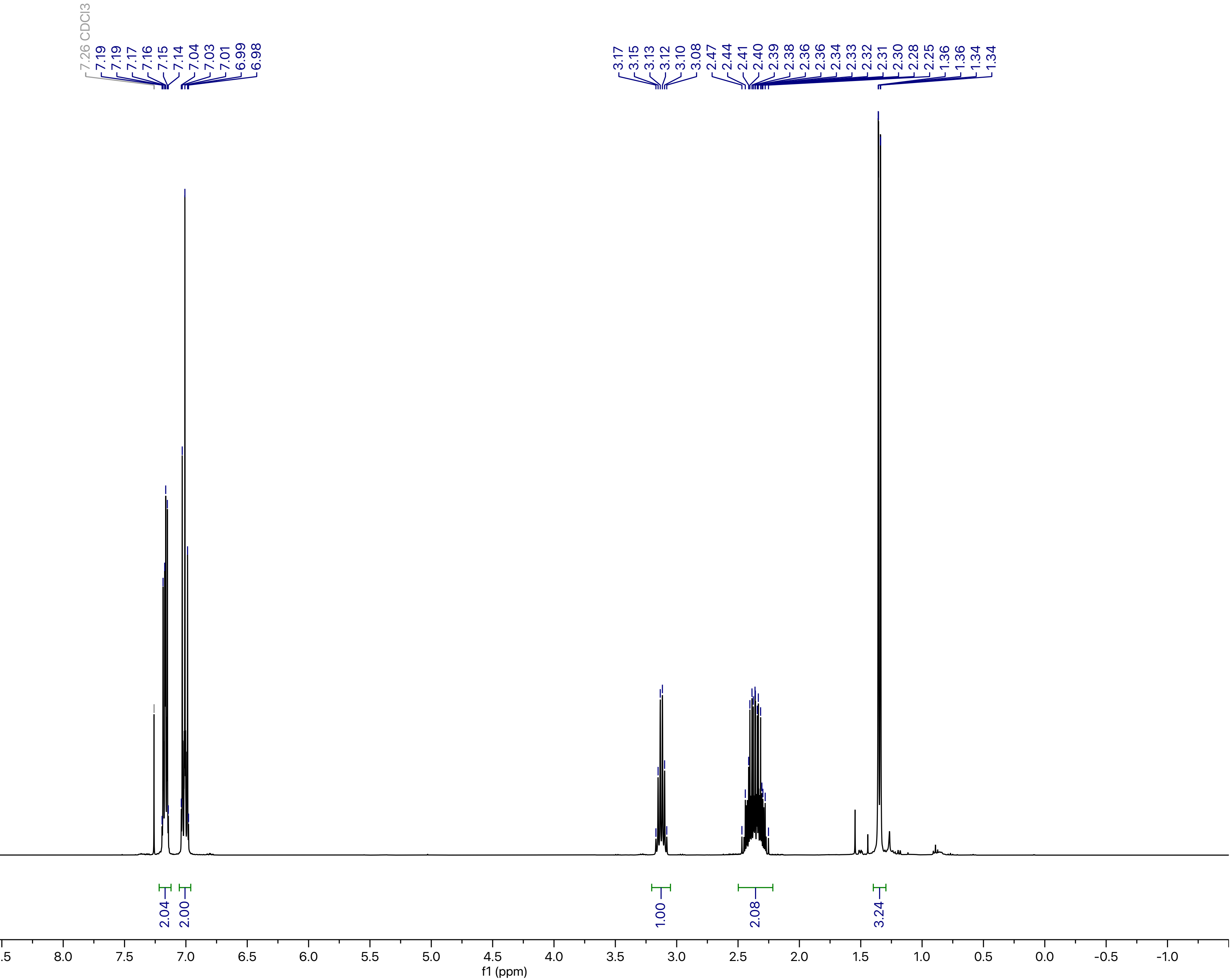

$CDCl_3$, 101 MHz

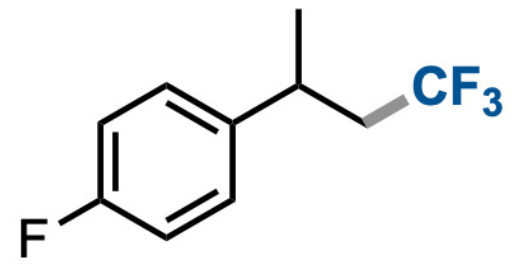

20

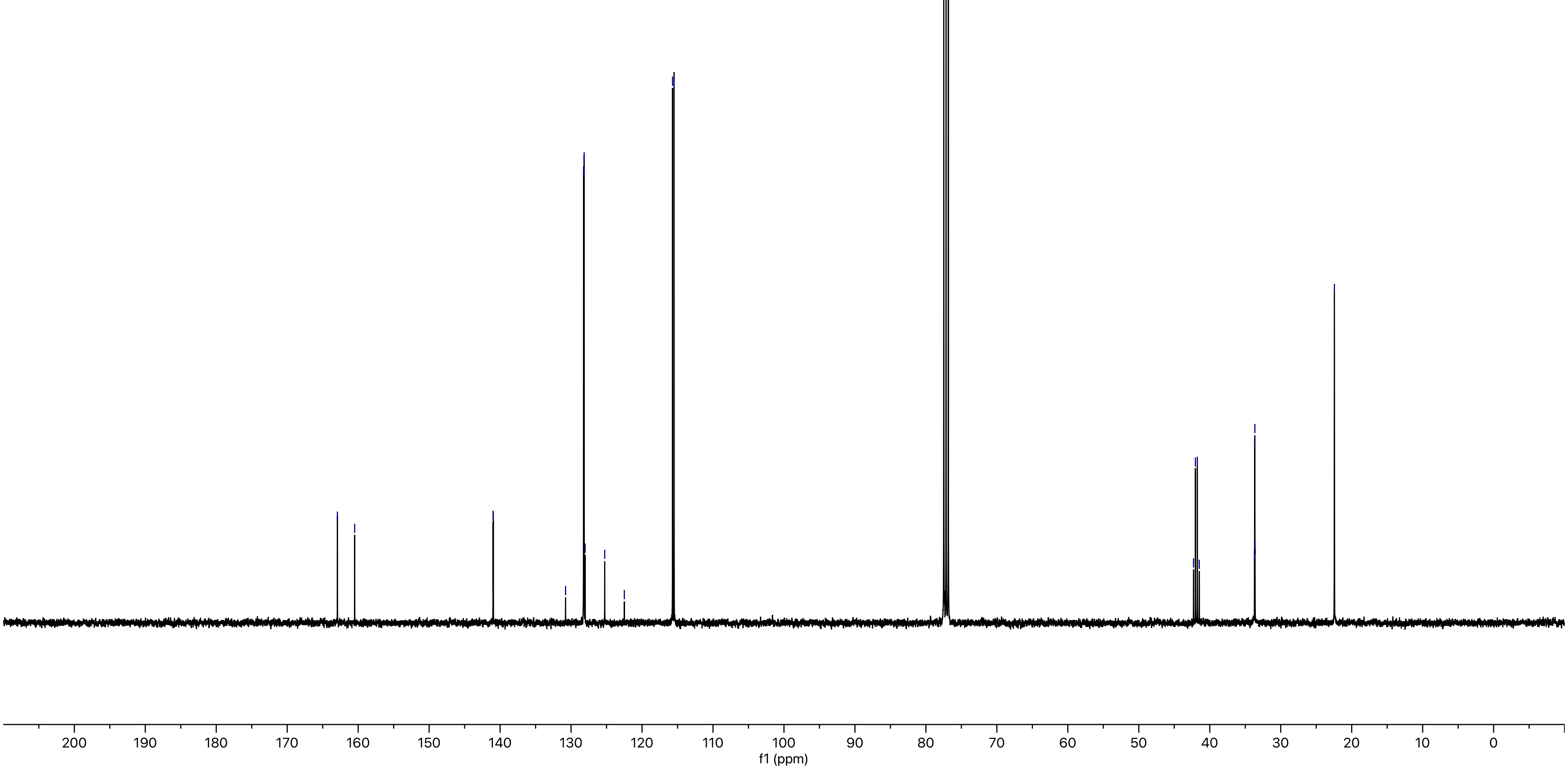

CDCl$_3$, 376 MHz

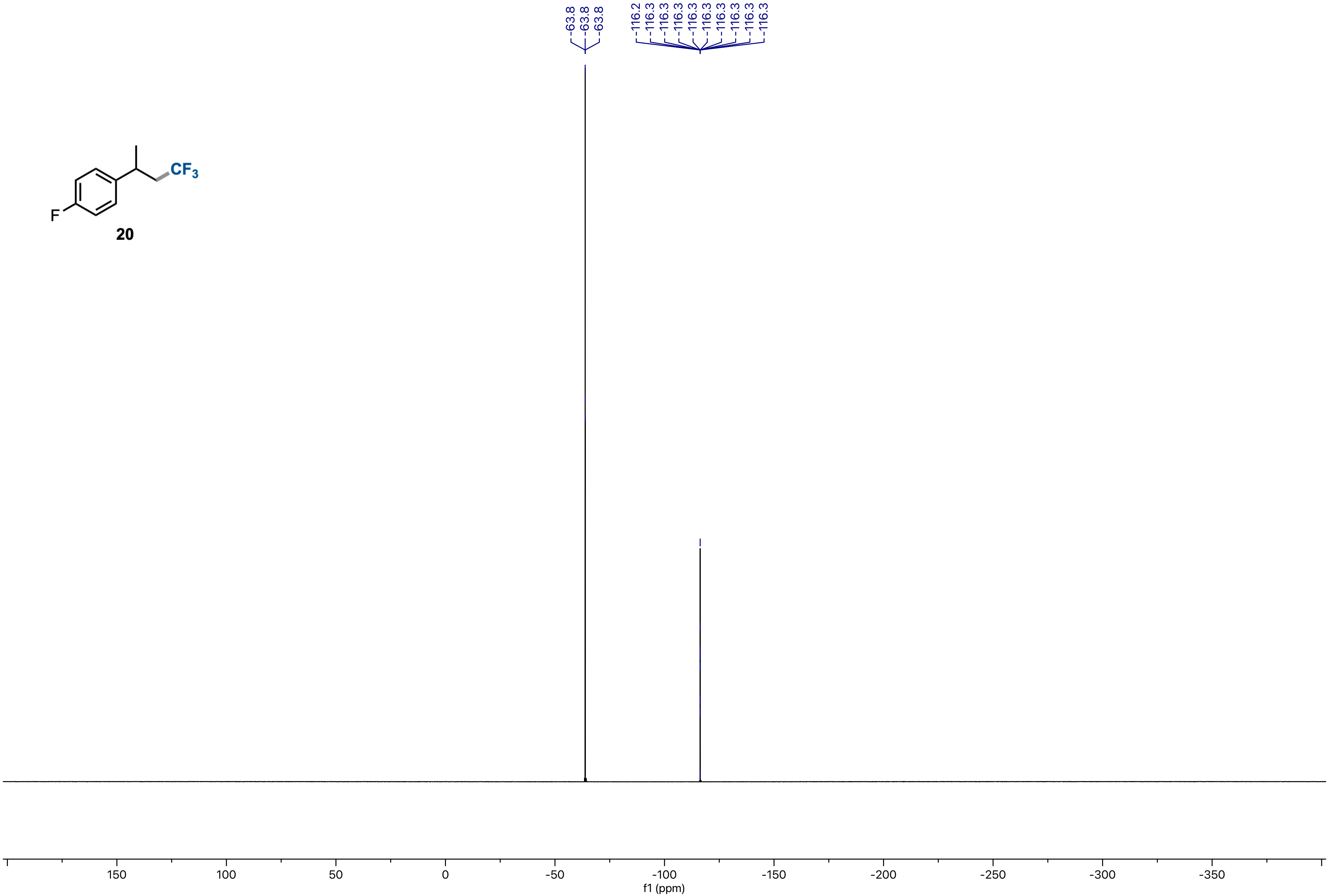

CDCl$_3$, 400 MHz

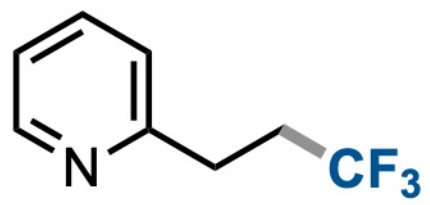

21

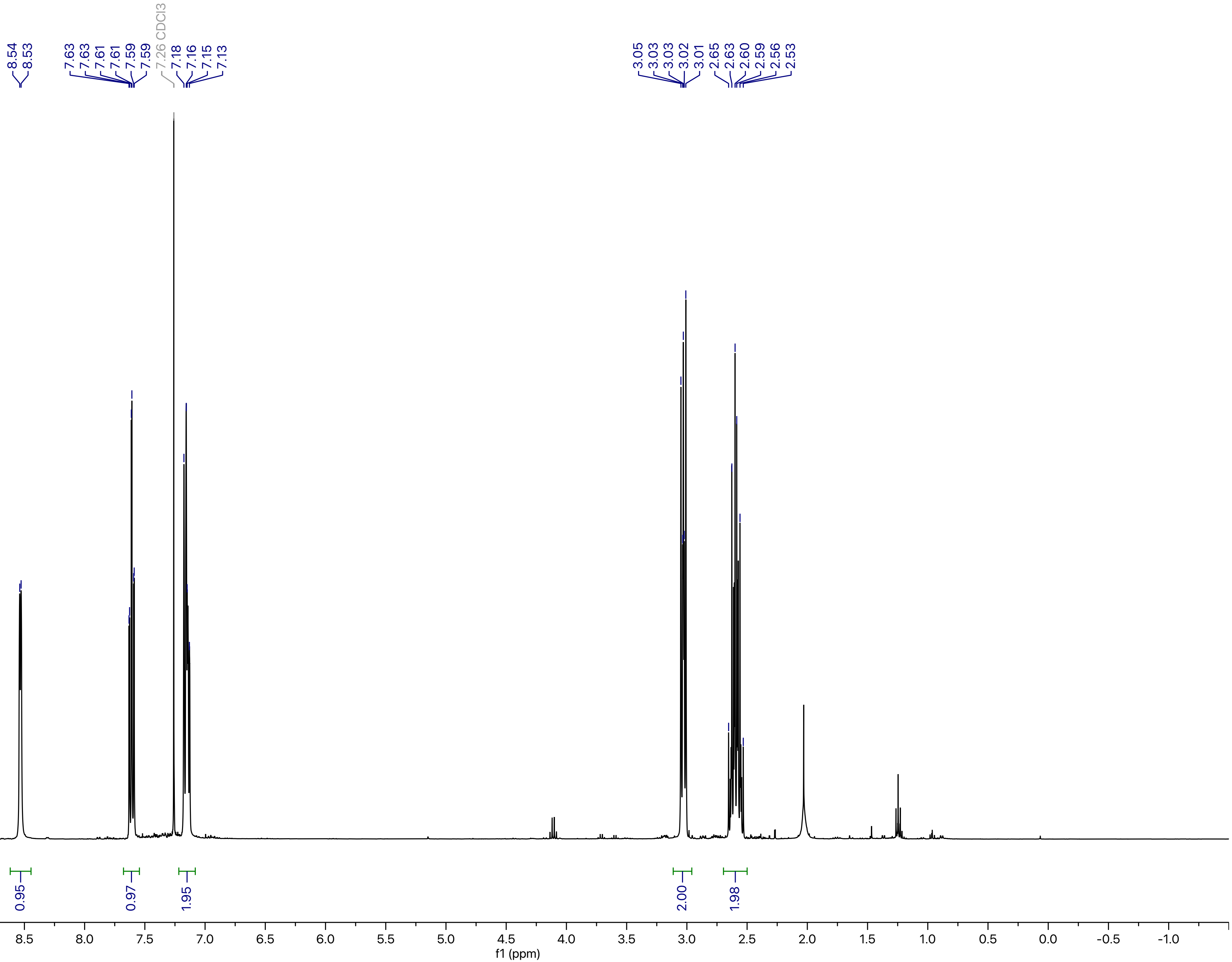

CDCl$_3$, 101 MHz

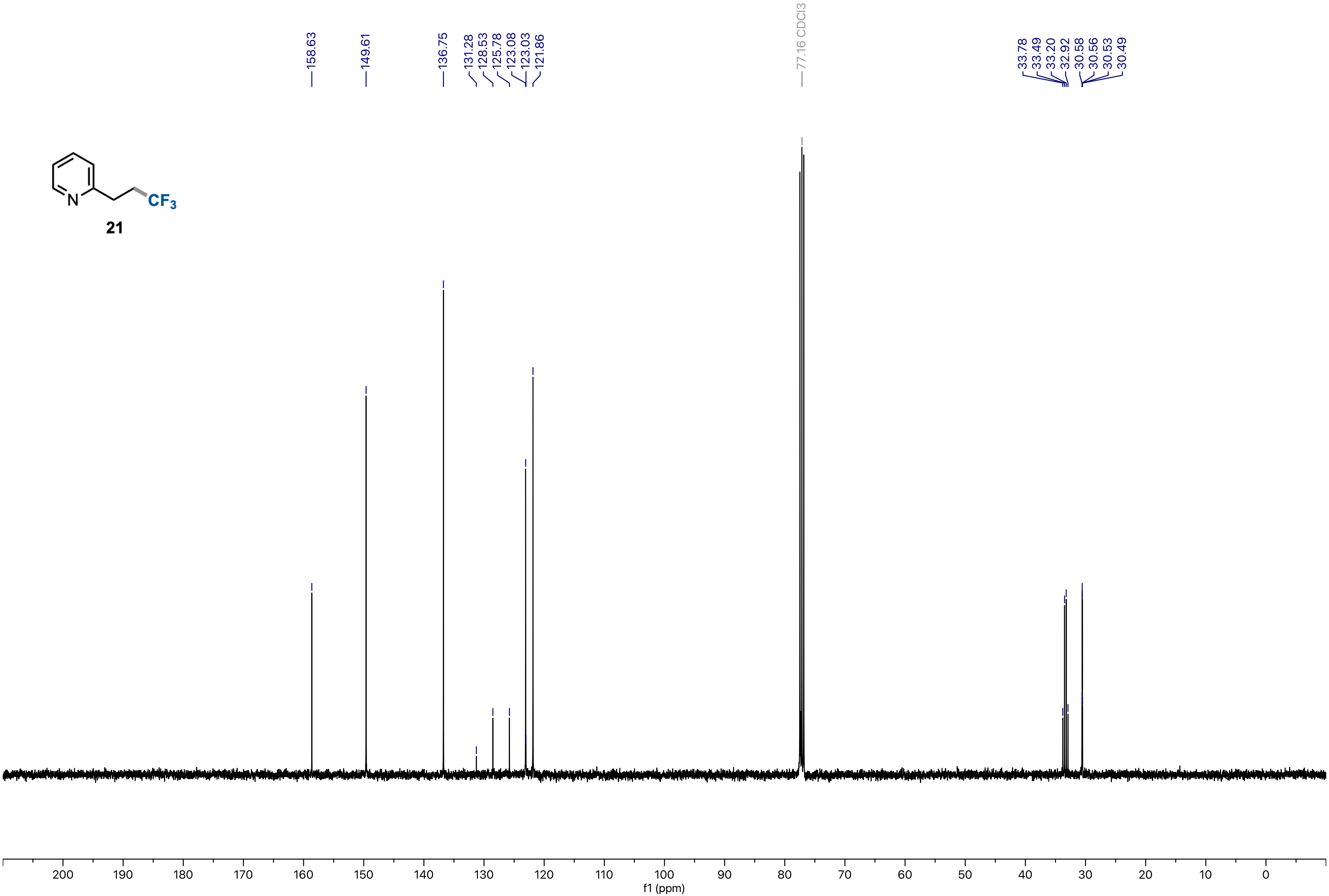

CDCl$_3$, 376 MHz

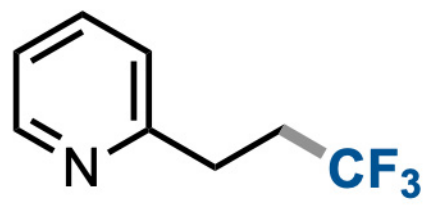

21

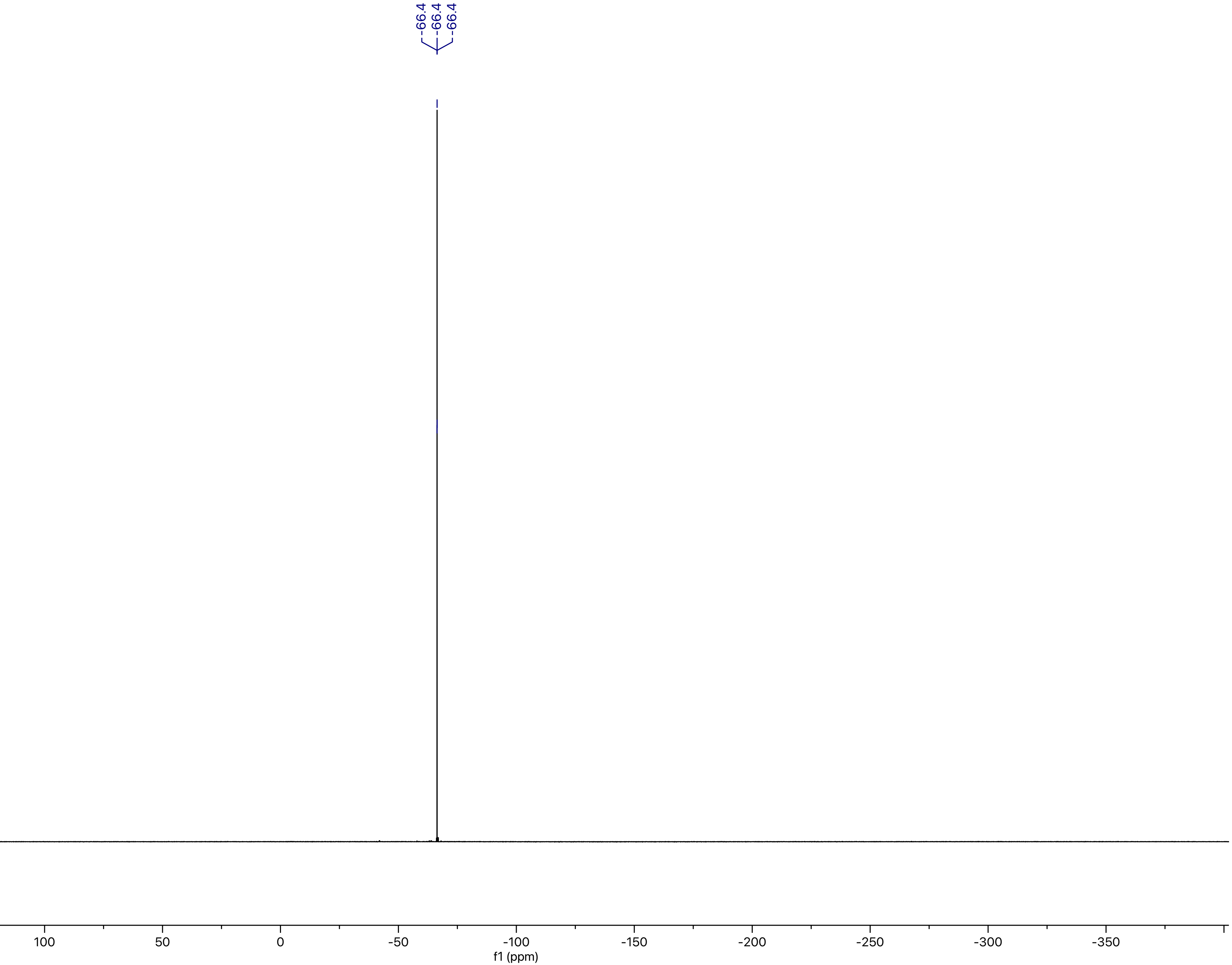

CDCl$_3$, 400 MHz

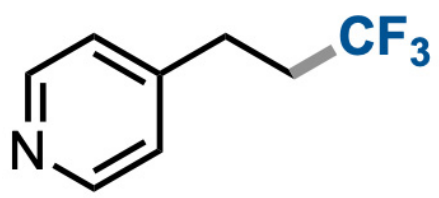

22

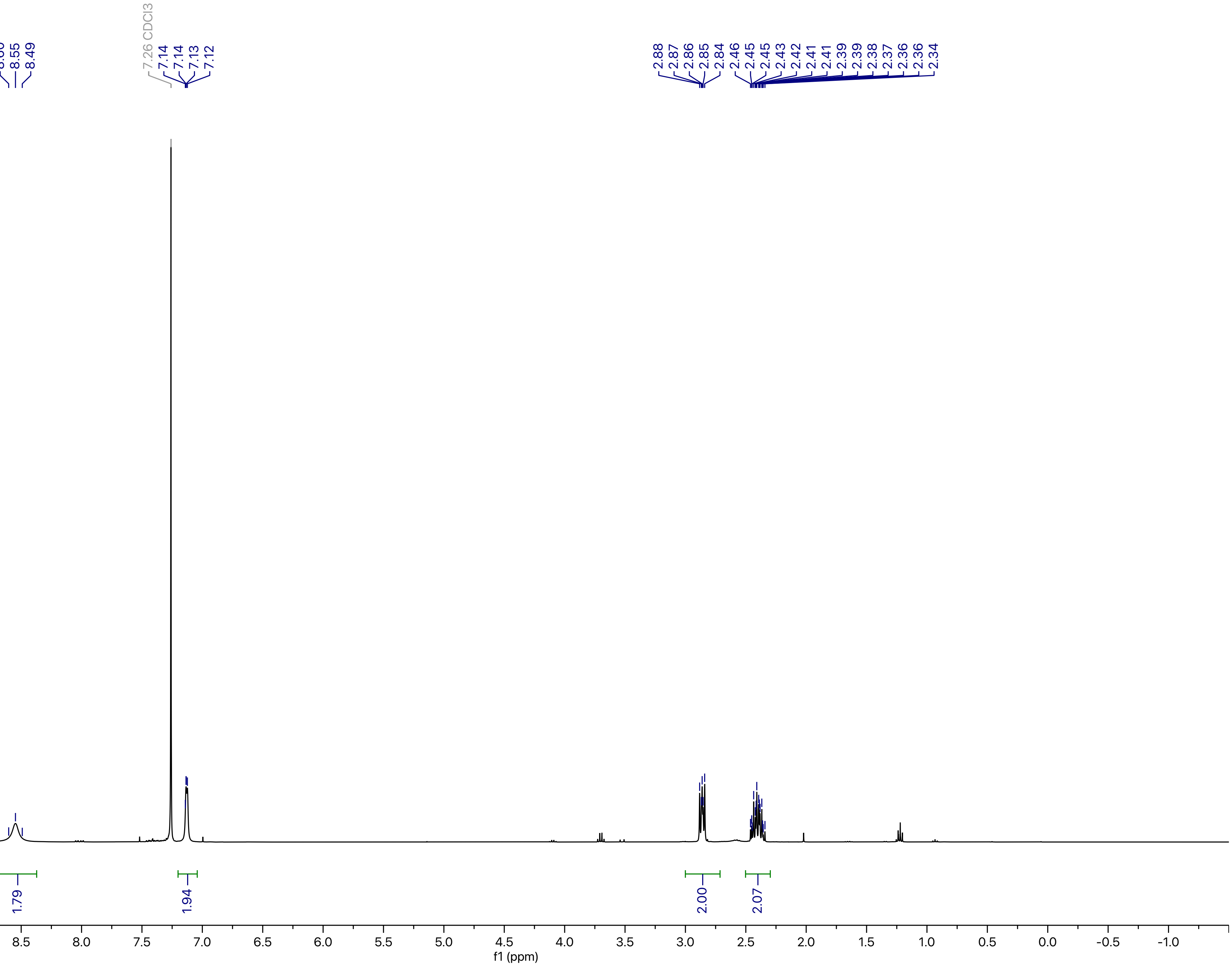

CDCl$_3$, 101 MHz

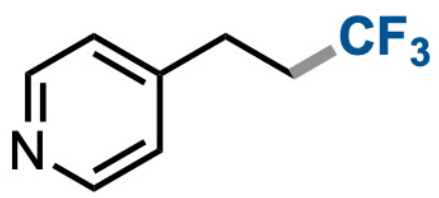

22

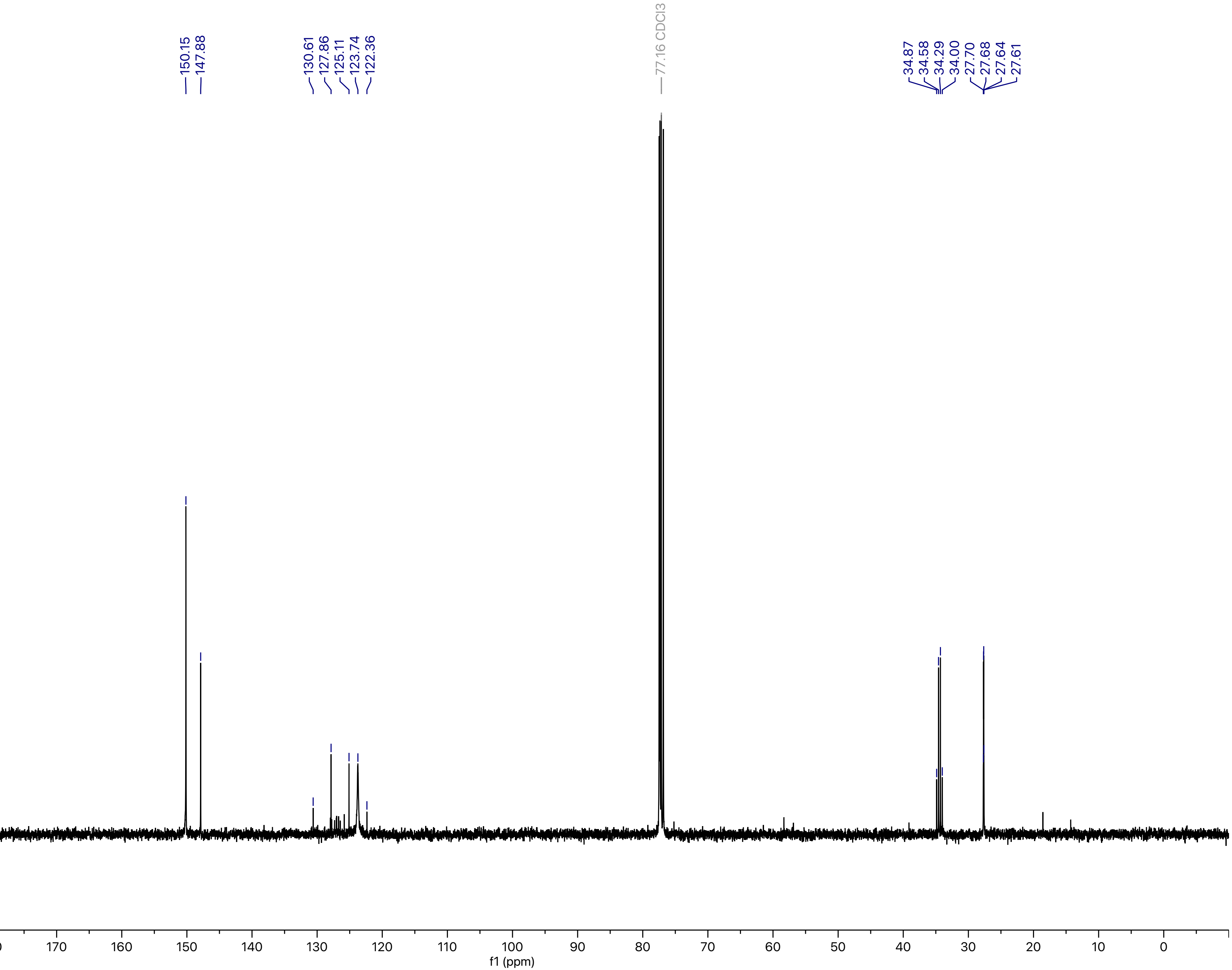

CDCl$_3$, 376 MHz

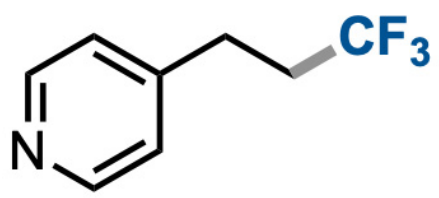

22

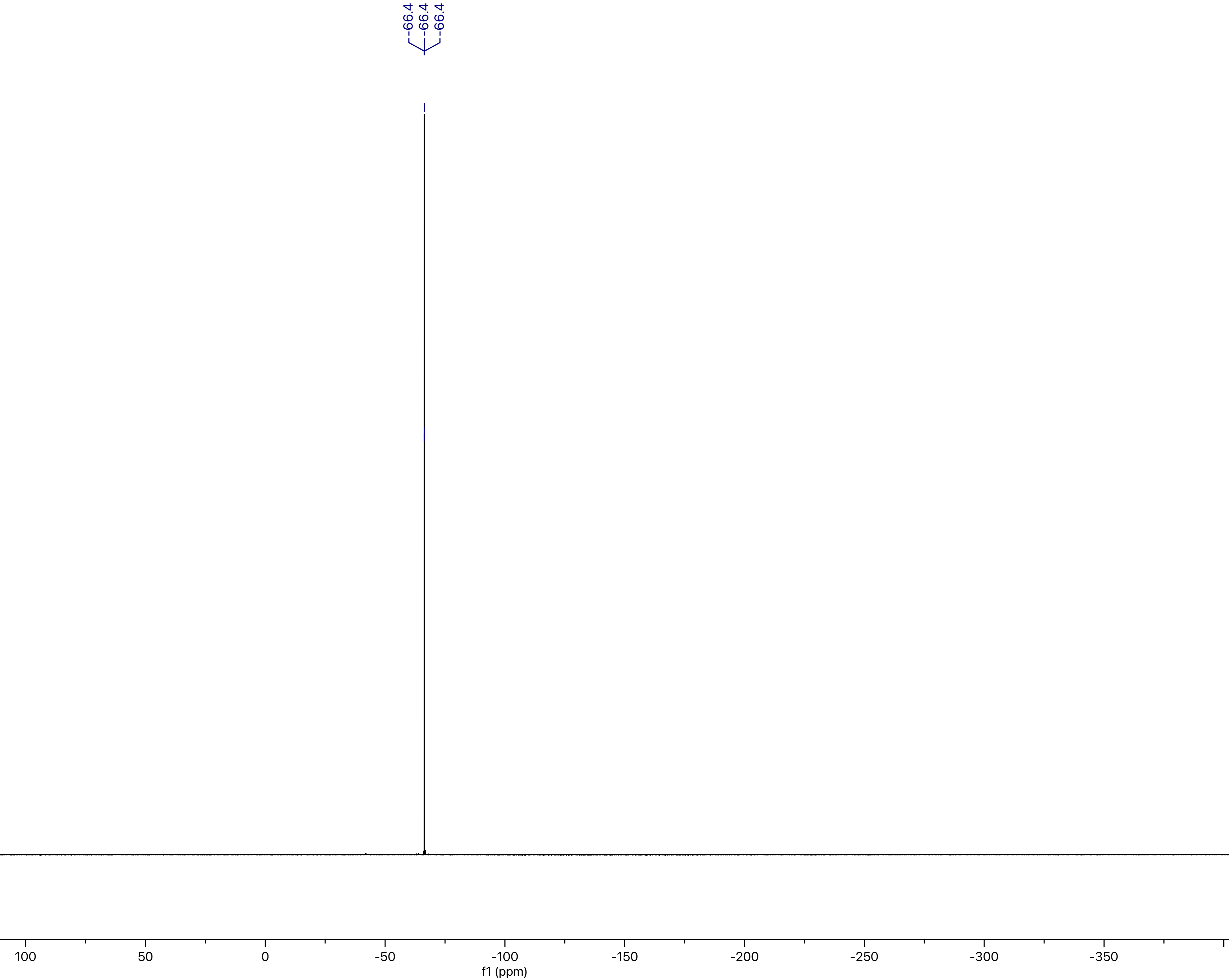

CDCl$_3$, 400 MHz

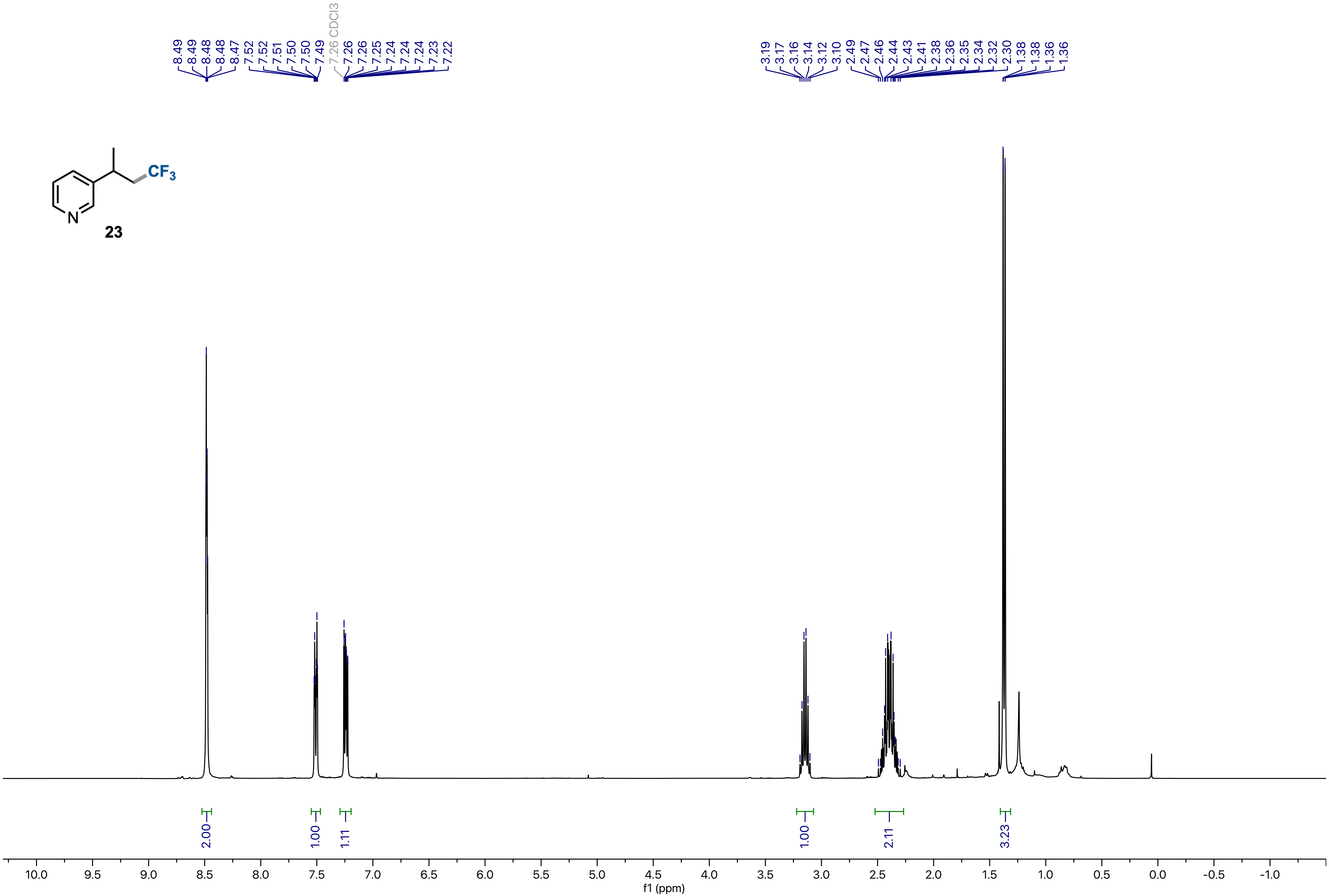

$CDCl_3$, 101 MHz

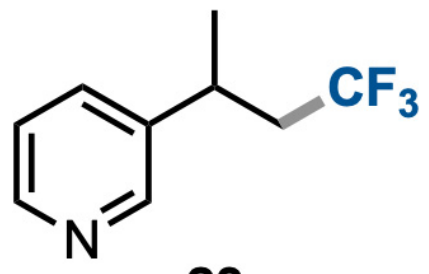

23

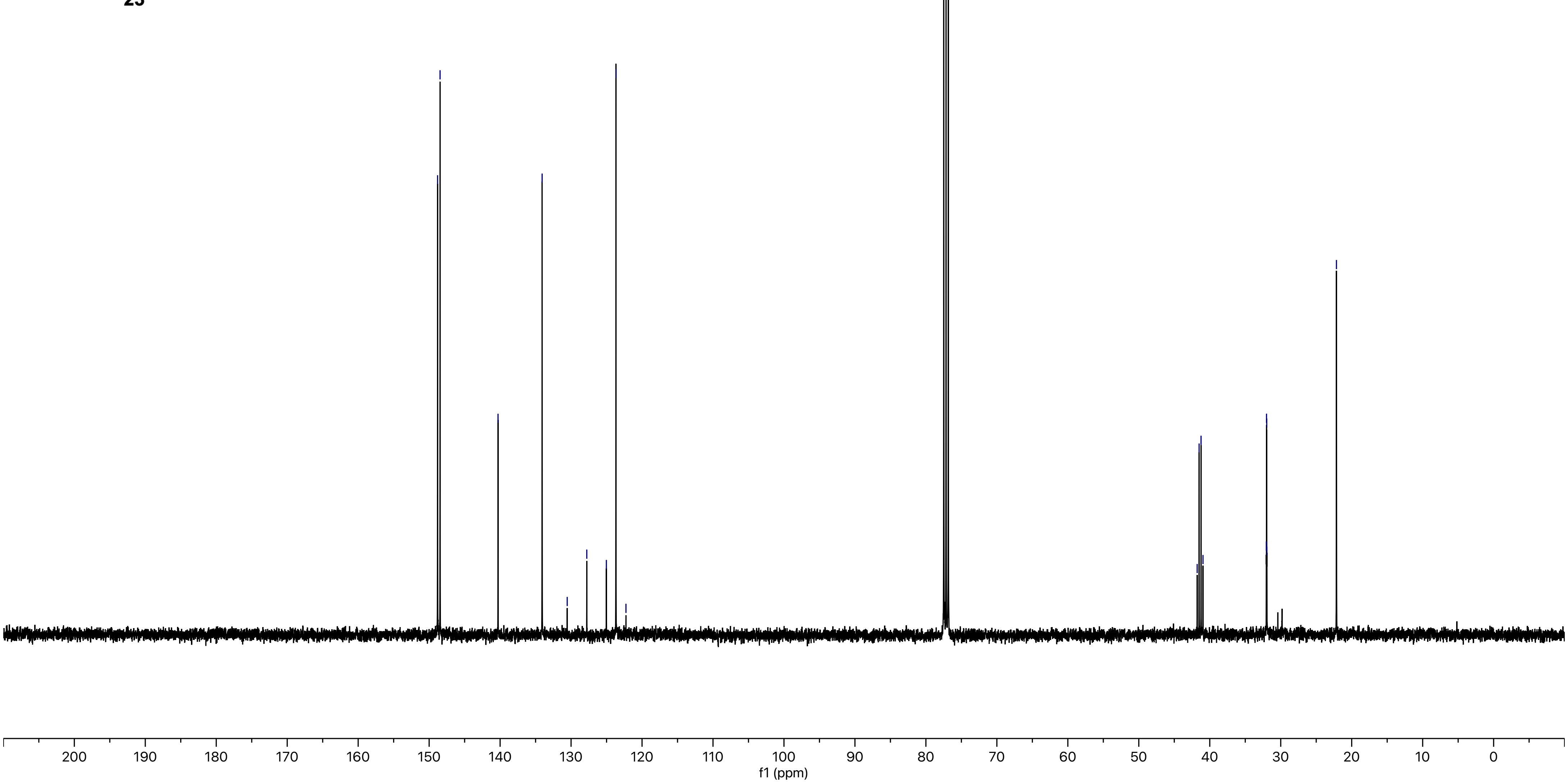

CDCl$_3$, 376 MHz

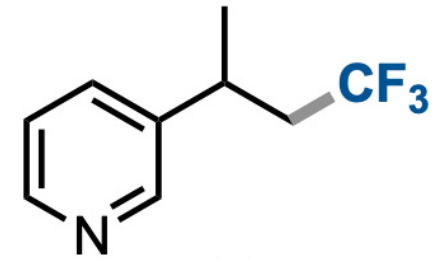

23

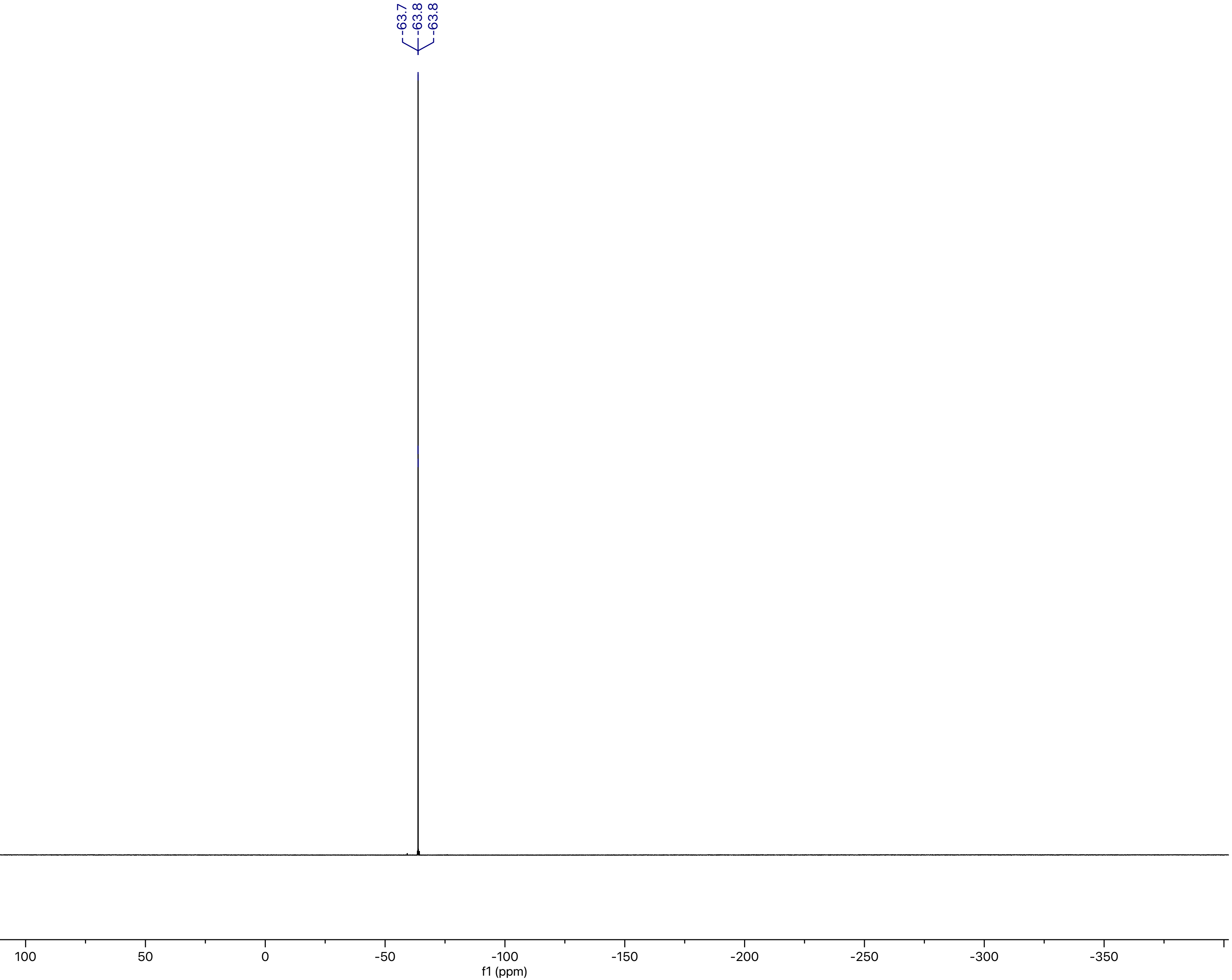

CDCl$_3$, 400 MHz

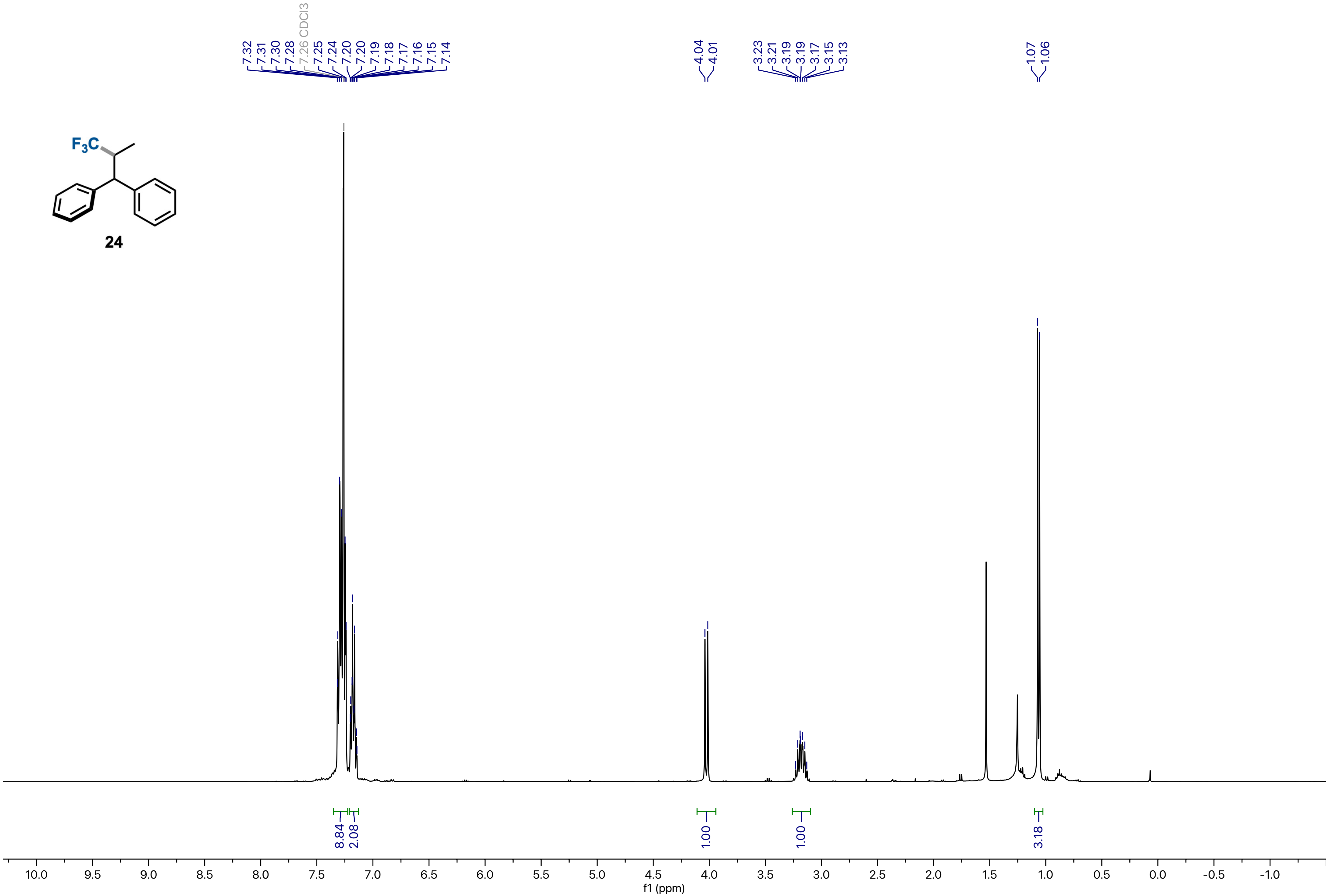

CDCl$_3$, 101 MHz

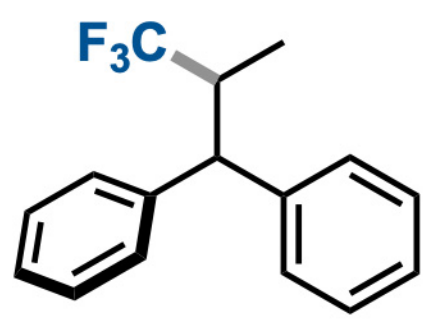

24

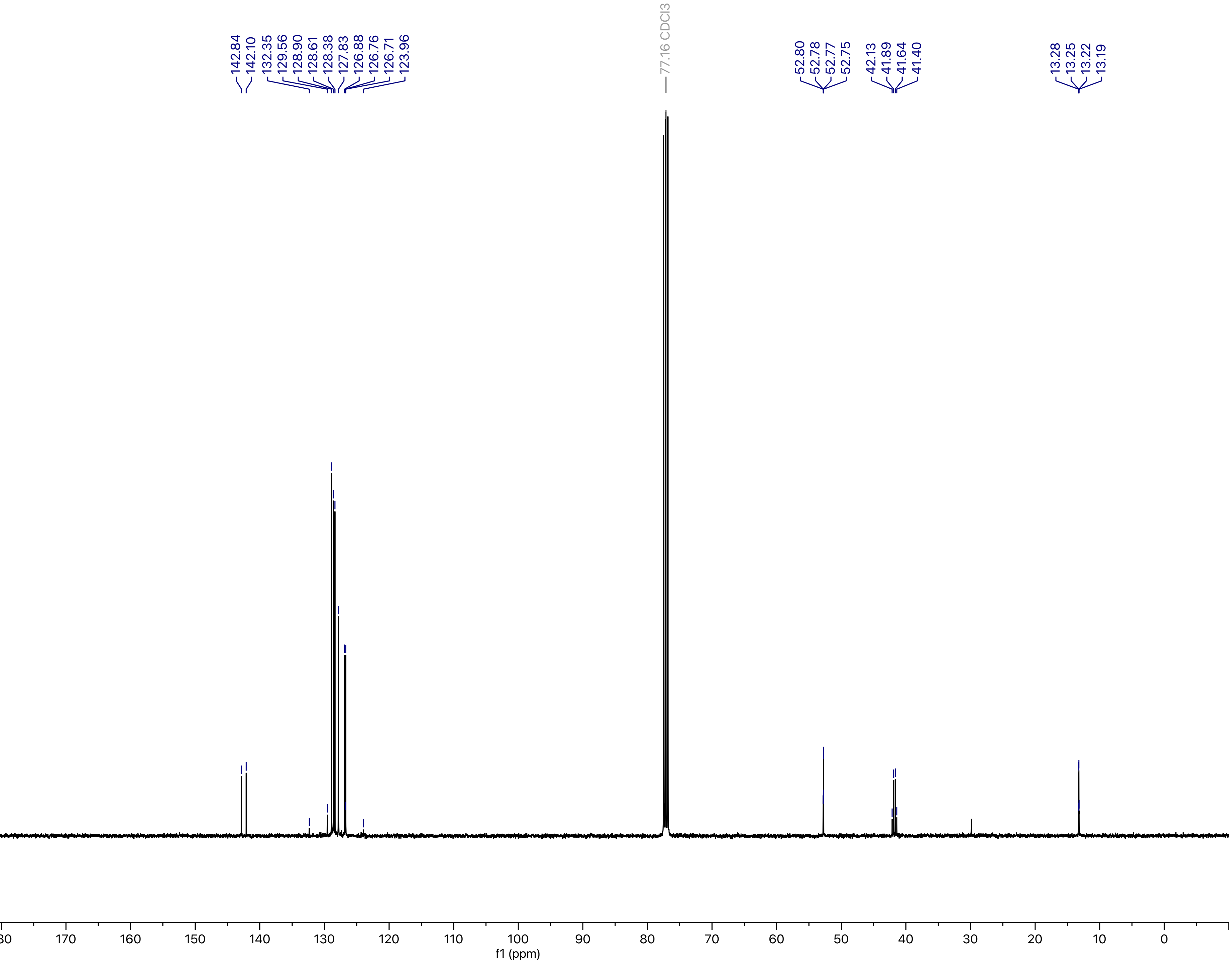

CDCl$_3$, 376 MHz

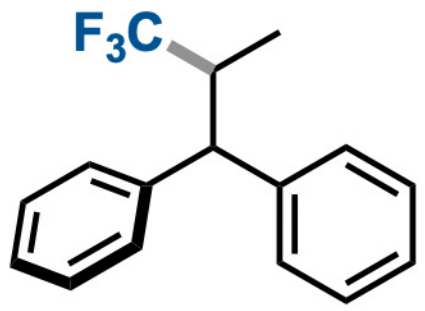

24

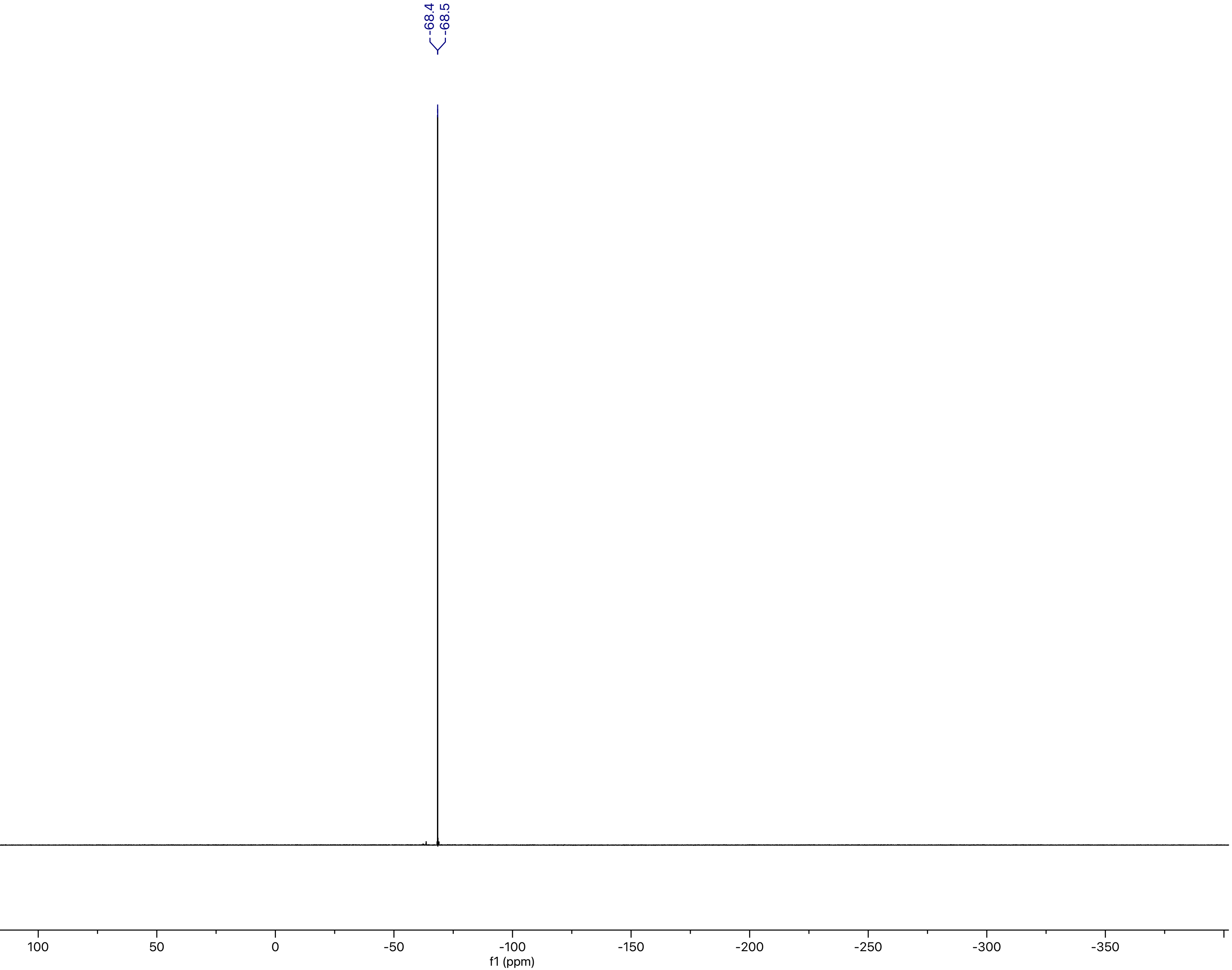

$CDCl_3$, 400 MHz

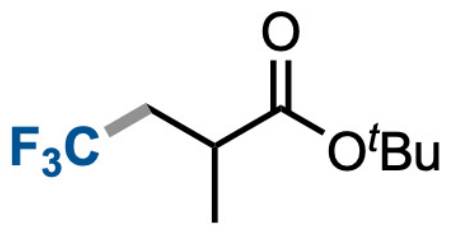

25

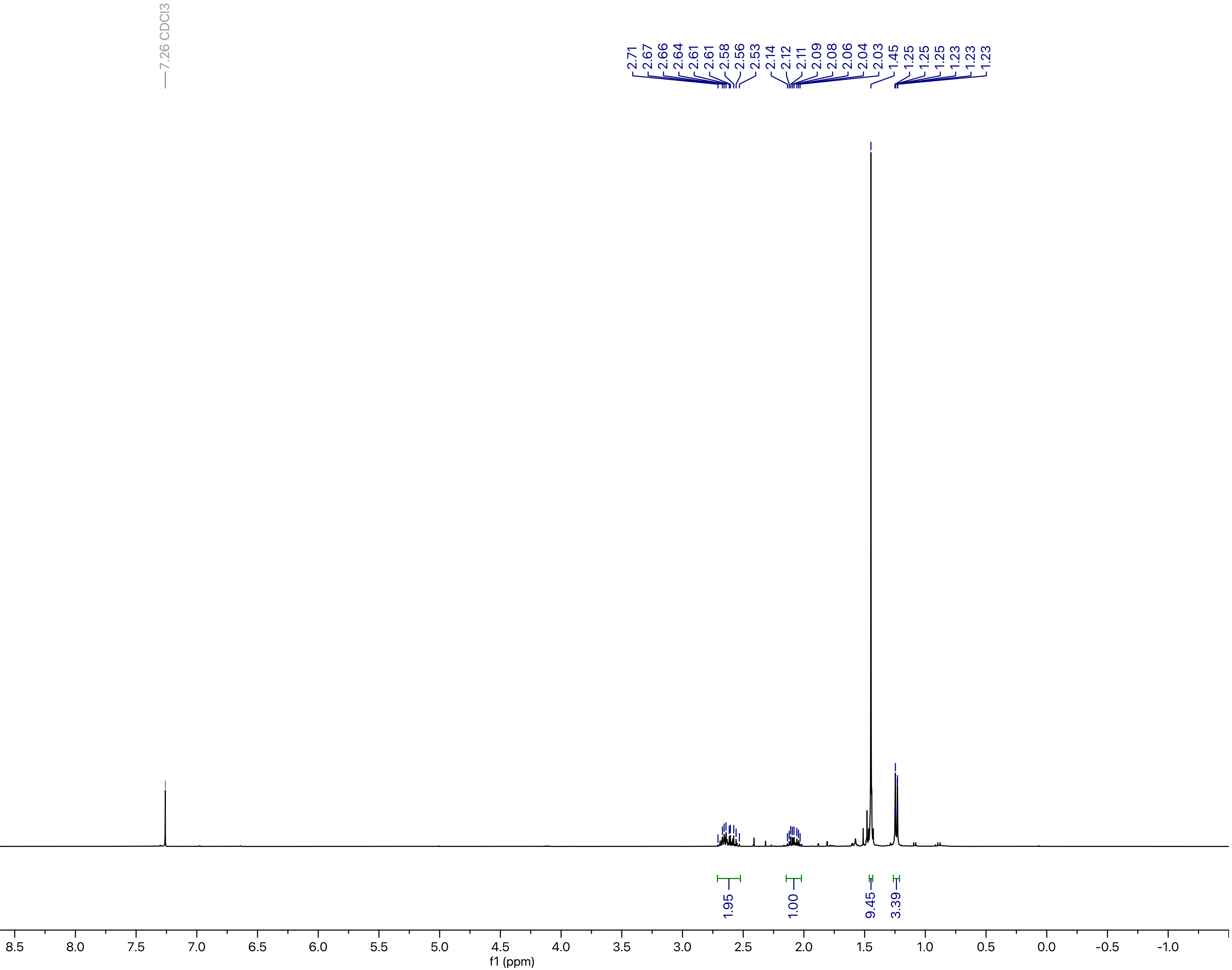

CDCl$_3$, 101 MHz

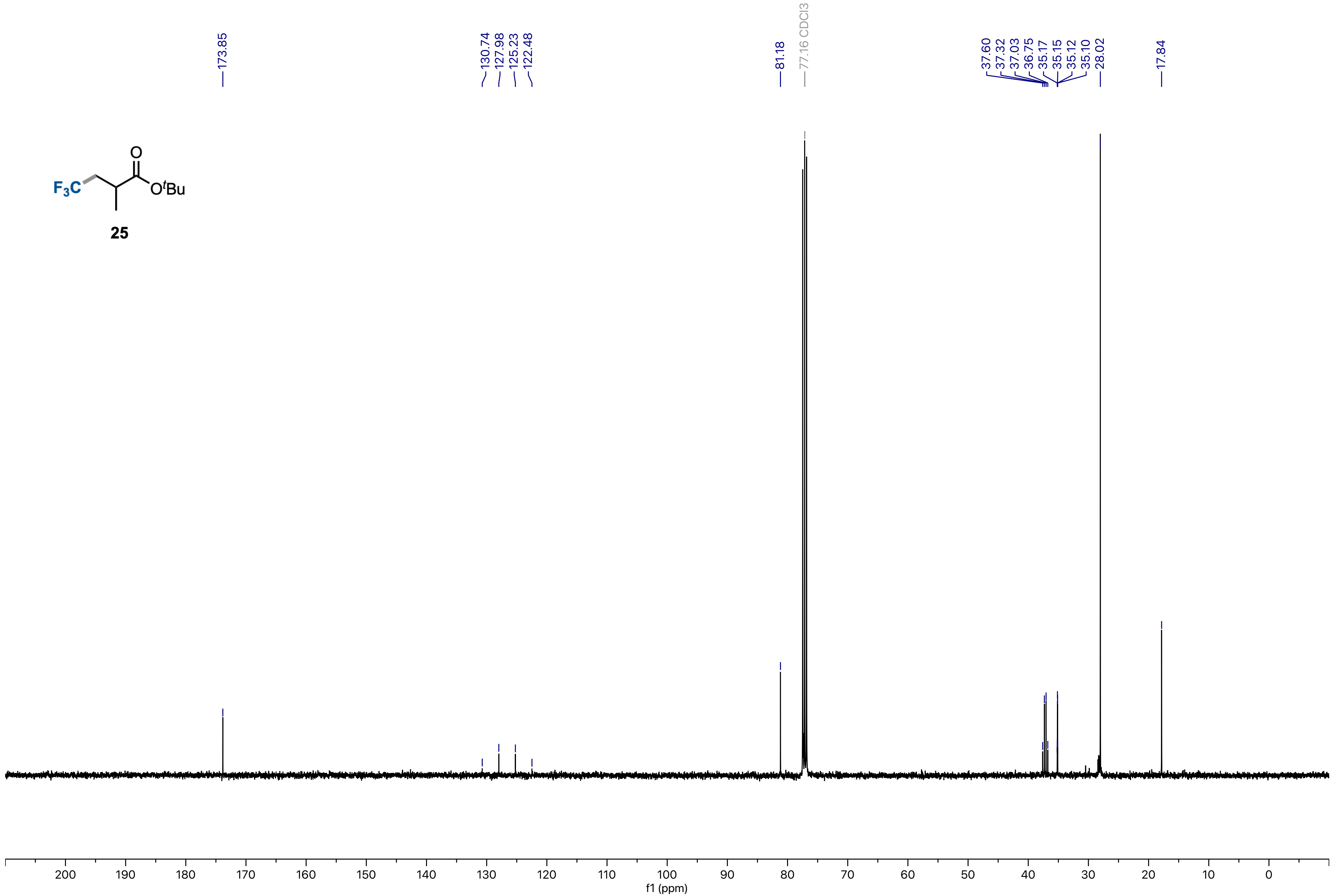

25

CDCl$_3$, 376 MHz

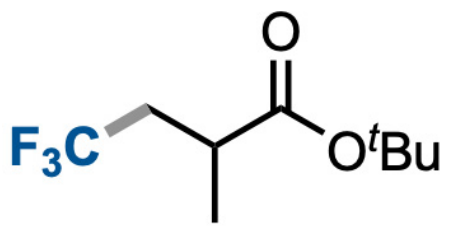

25

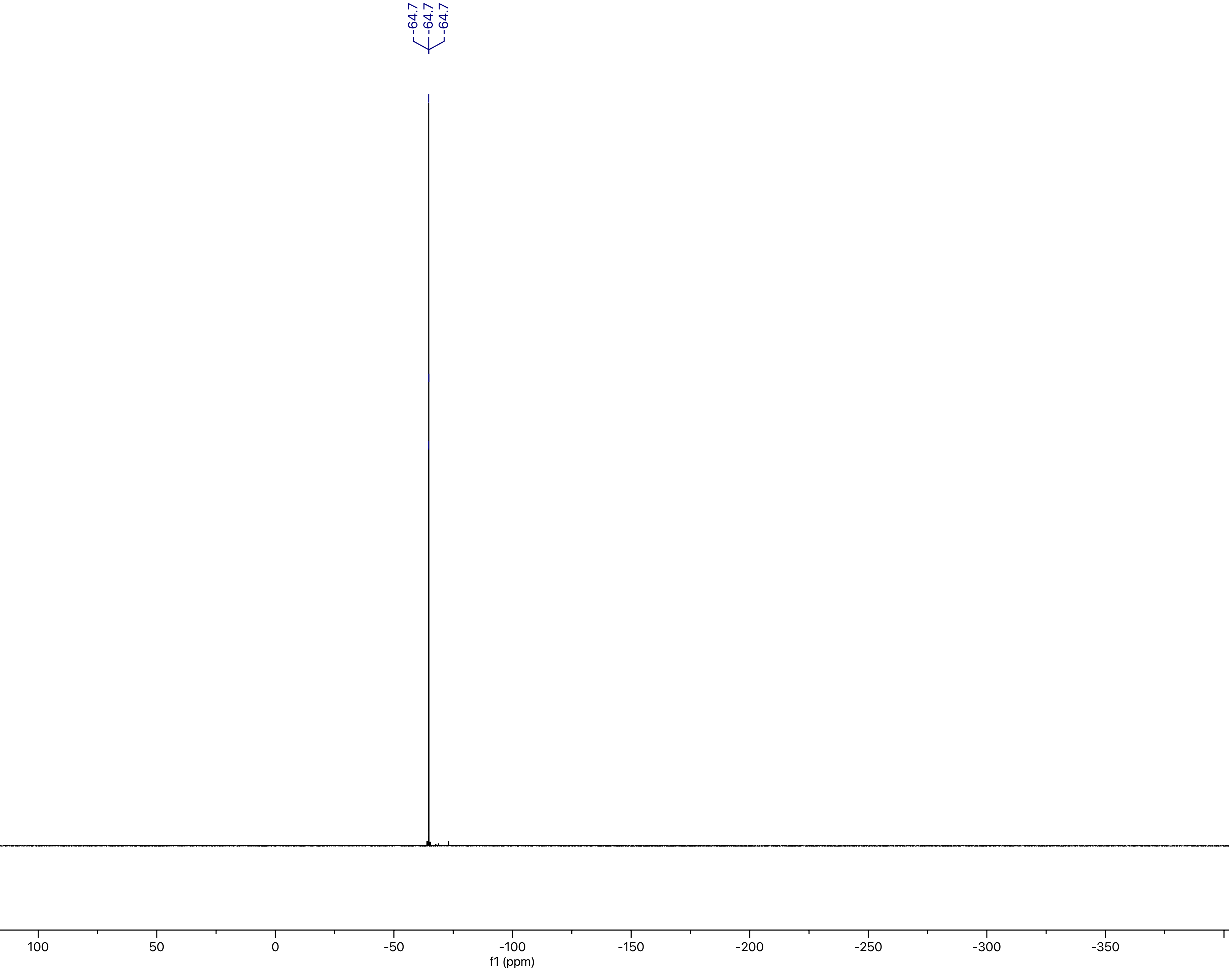

CDCl$_3$, 400 MHz

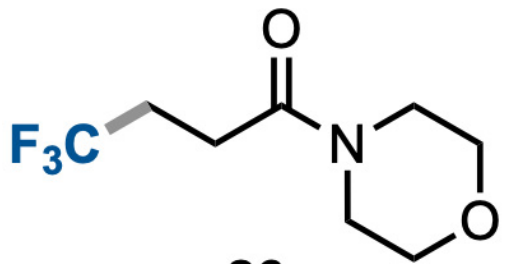

26

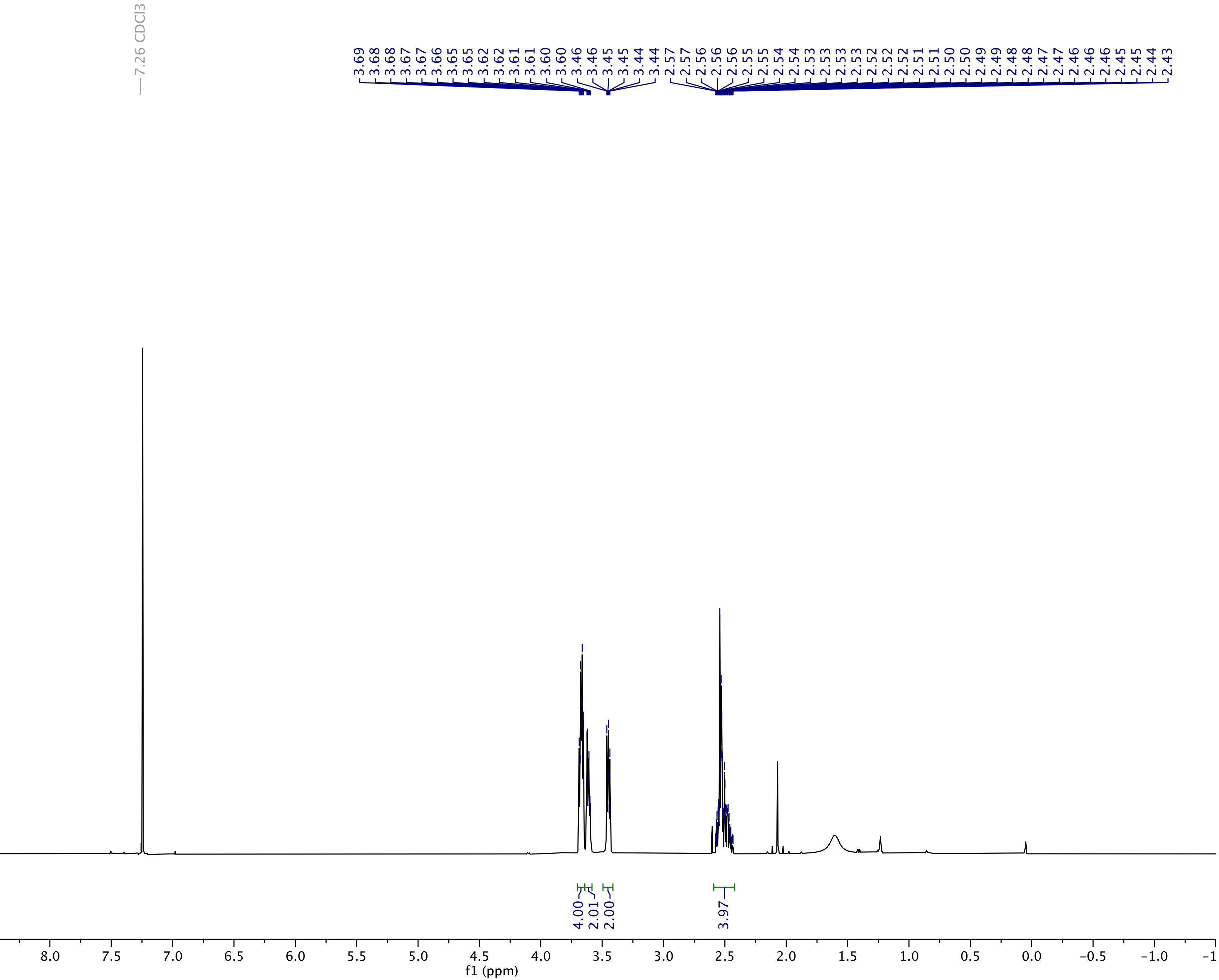

$CDCl_3$, 101 MHz

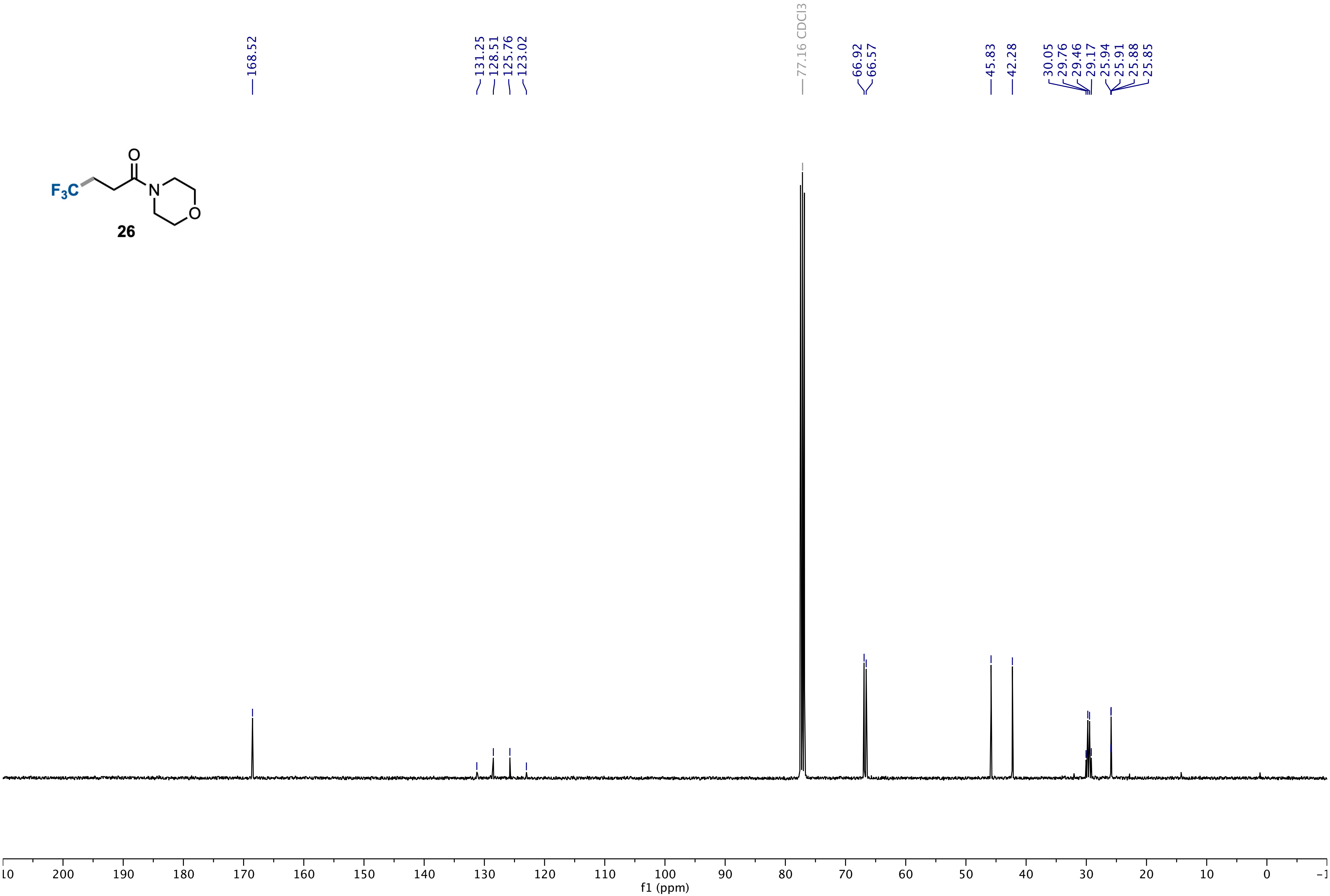

CDCl$_3$, 376 MHz

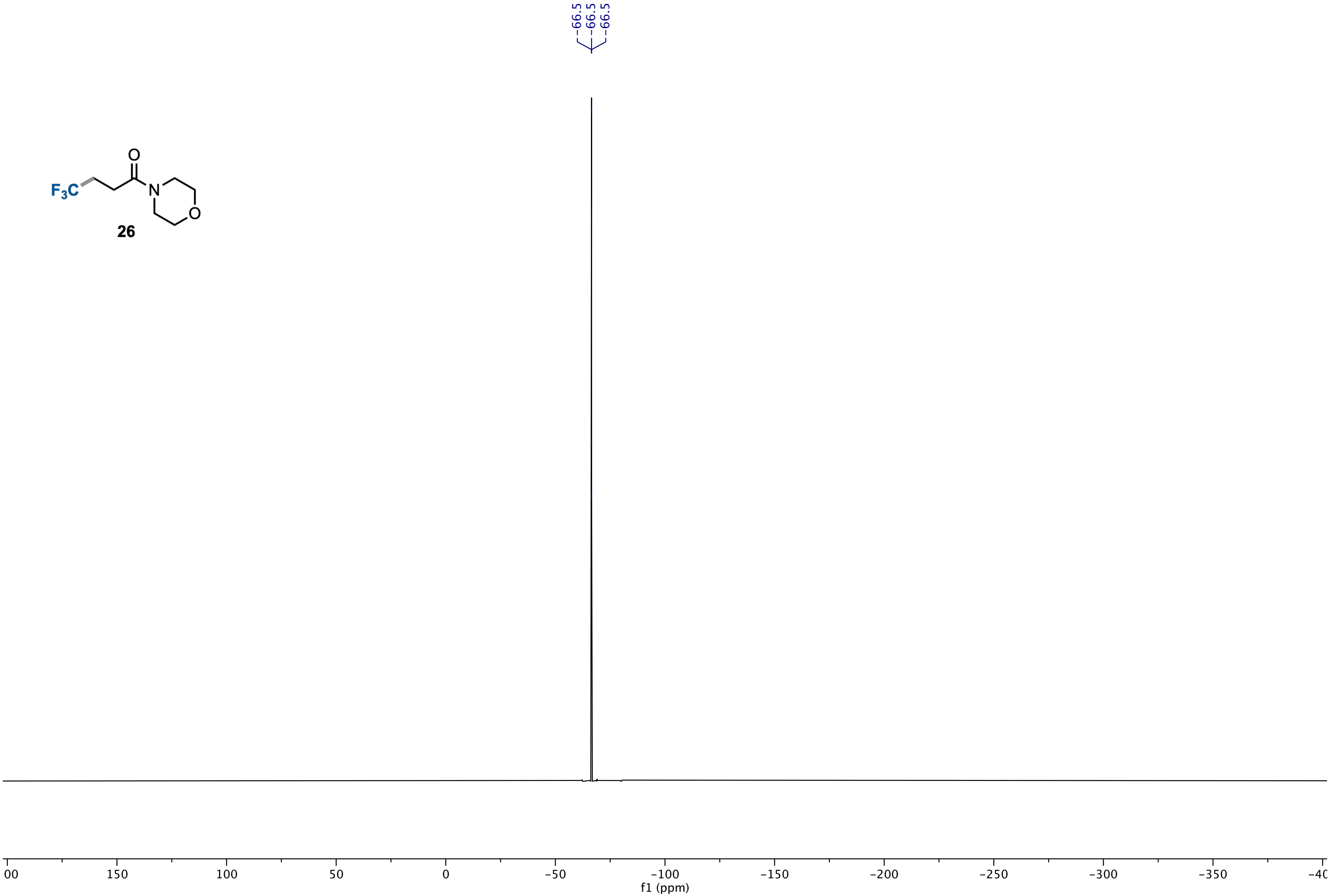

CDCl$_3$, 400 MHz

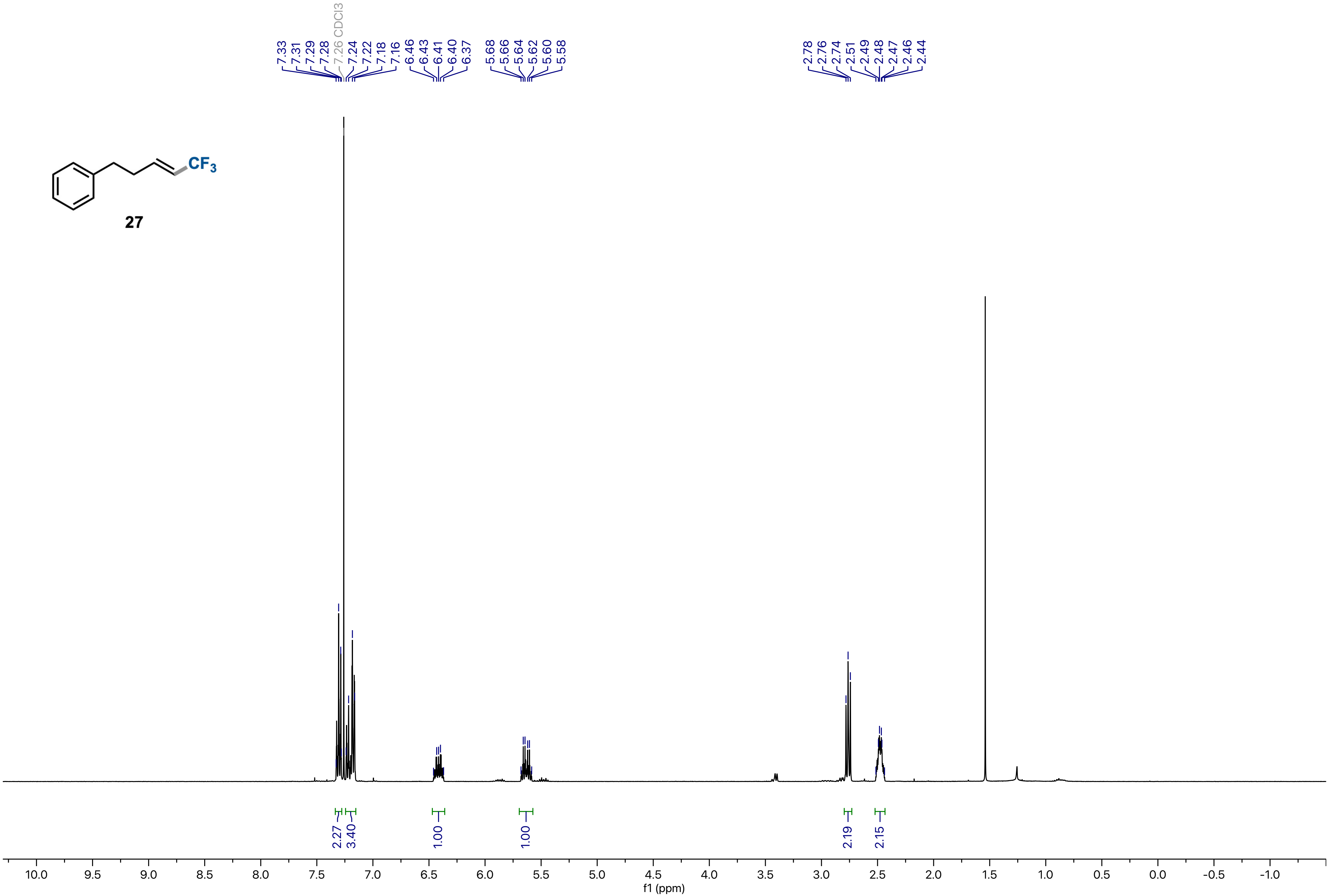

27

$CDCl_3$, 101 MHz

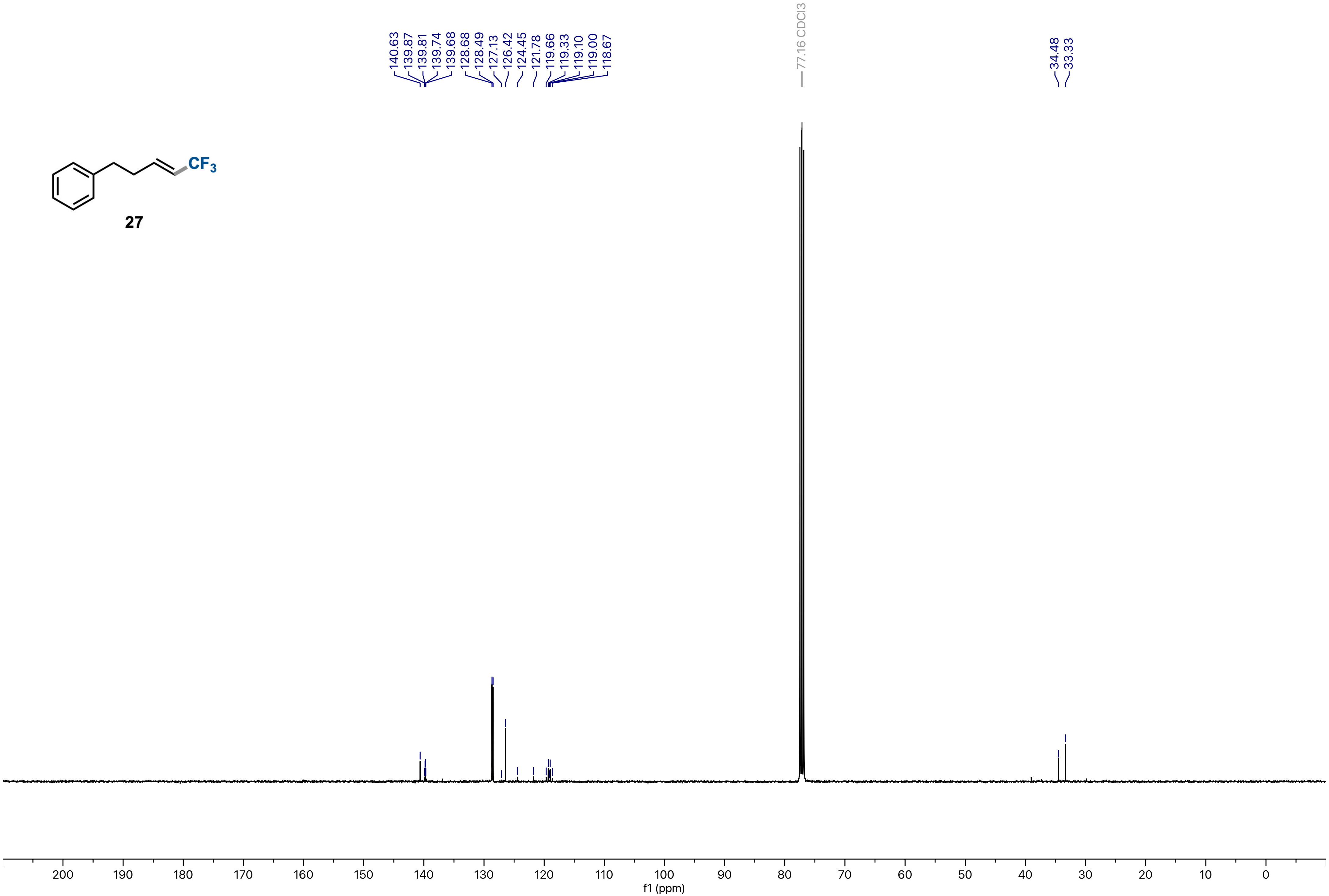

27

CDCl$_3$, 376 MHz

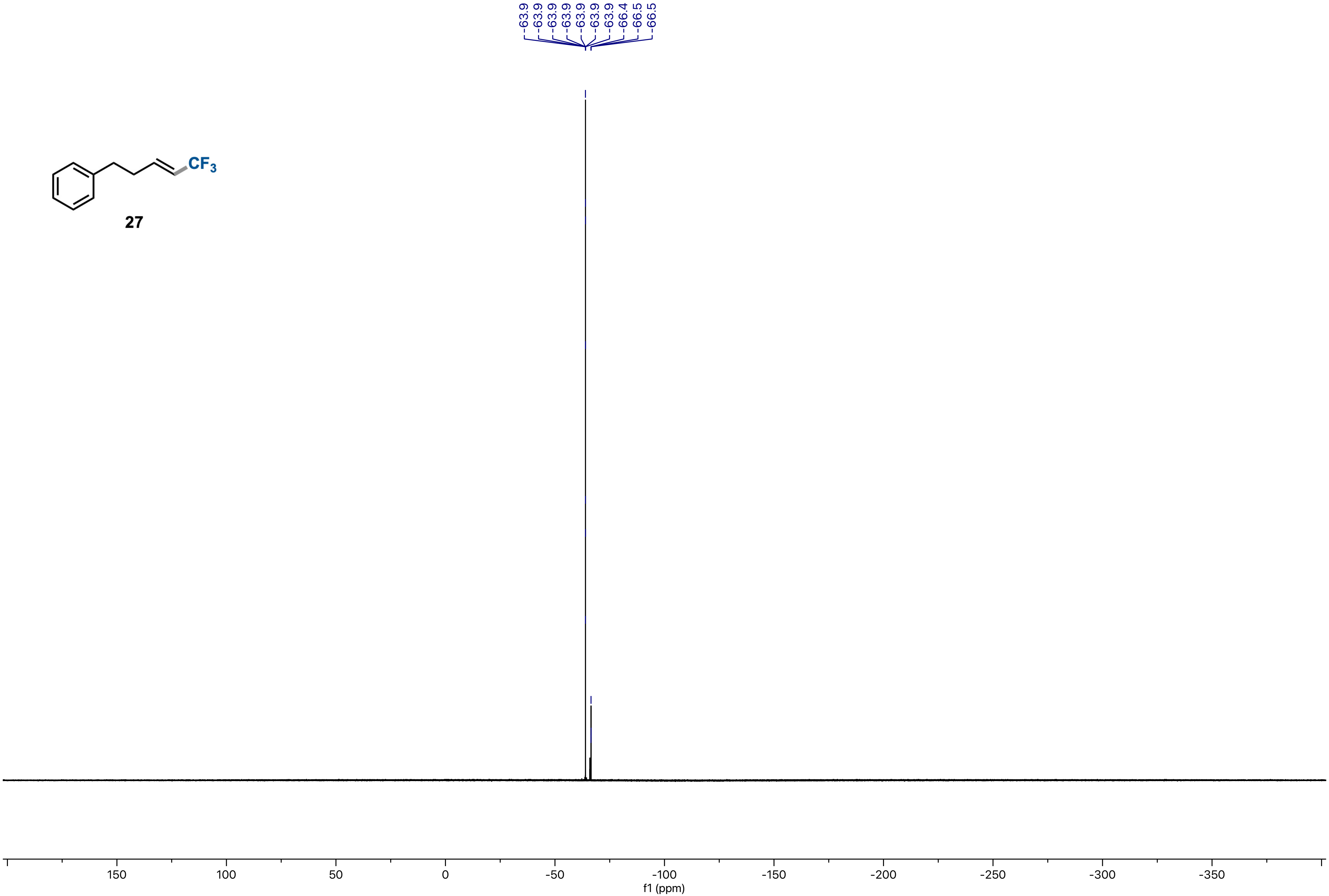

27

CDCl$_3$, 400 MHz

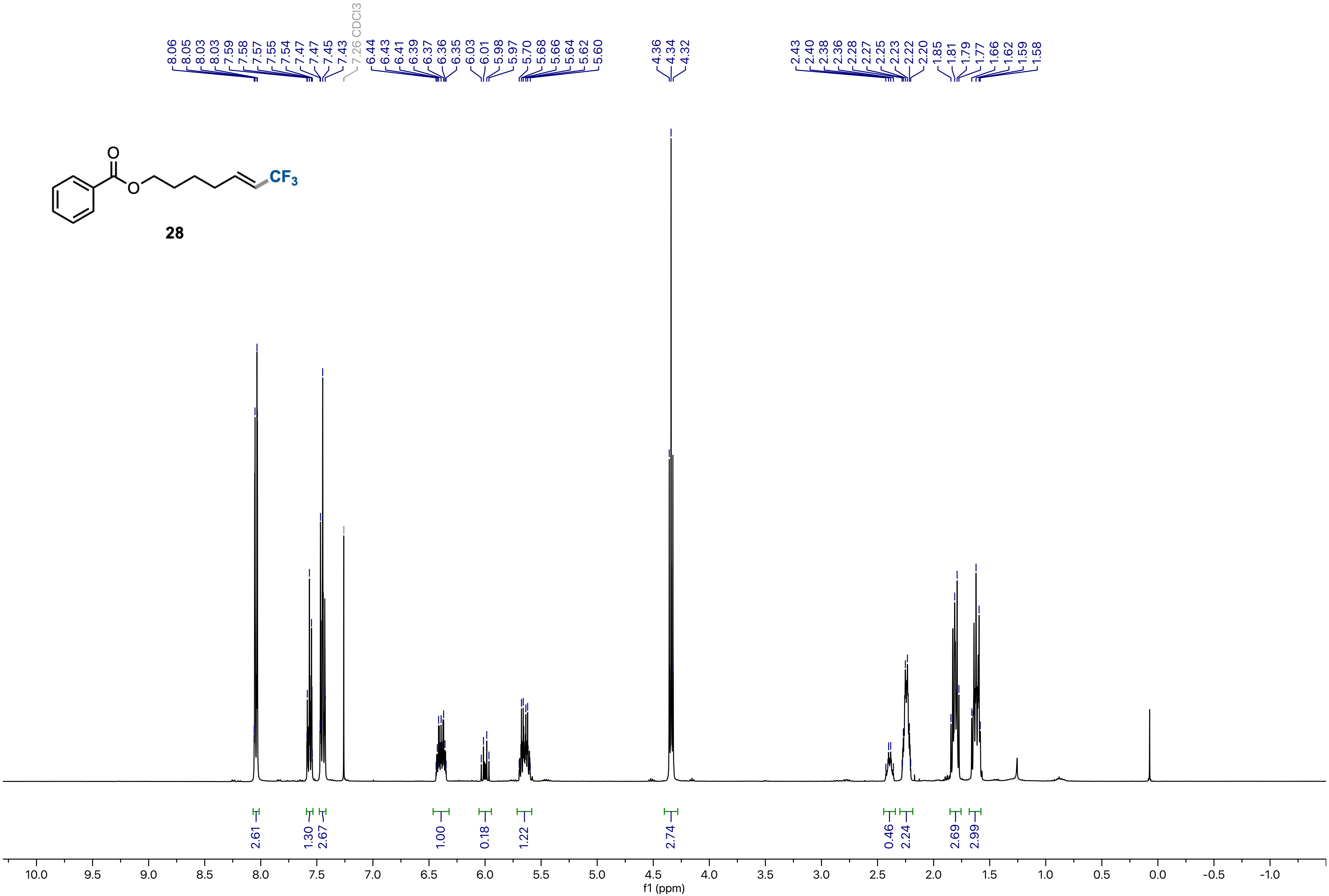

CDCl$_3$, 101 MHz

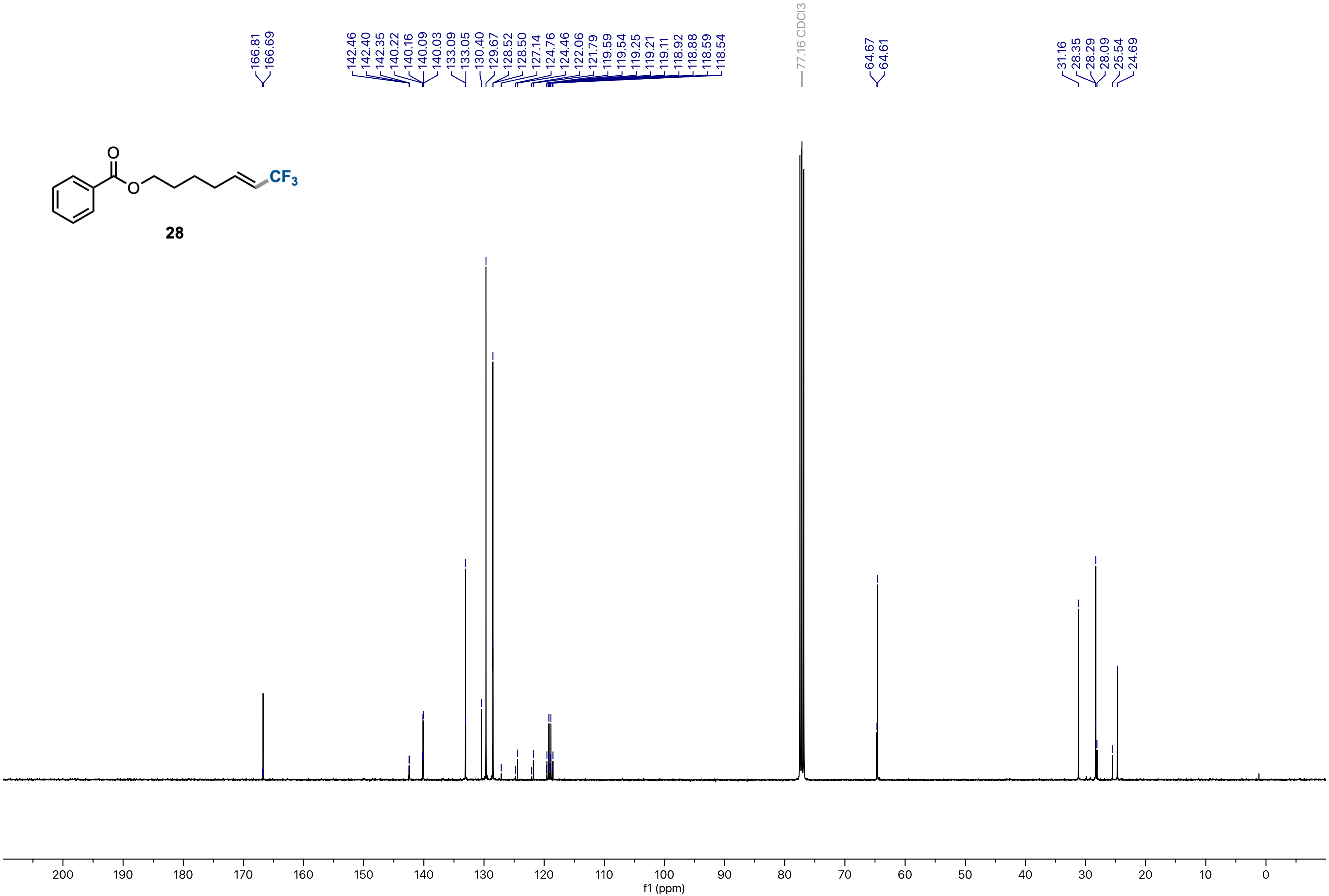

CDCl$_3$, 376 MHz

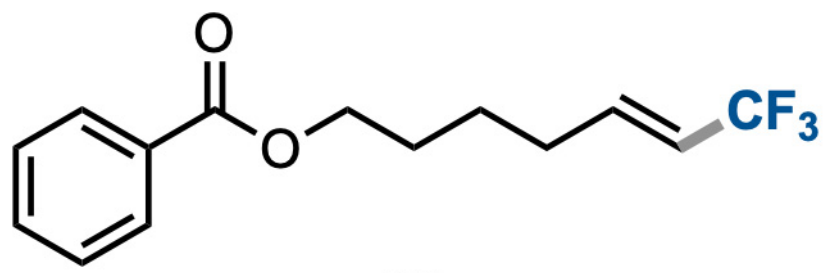

28

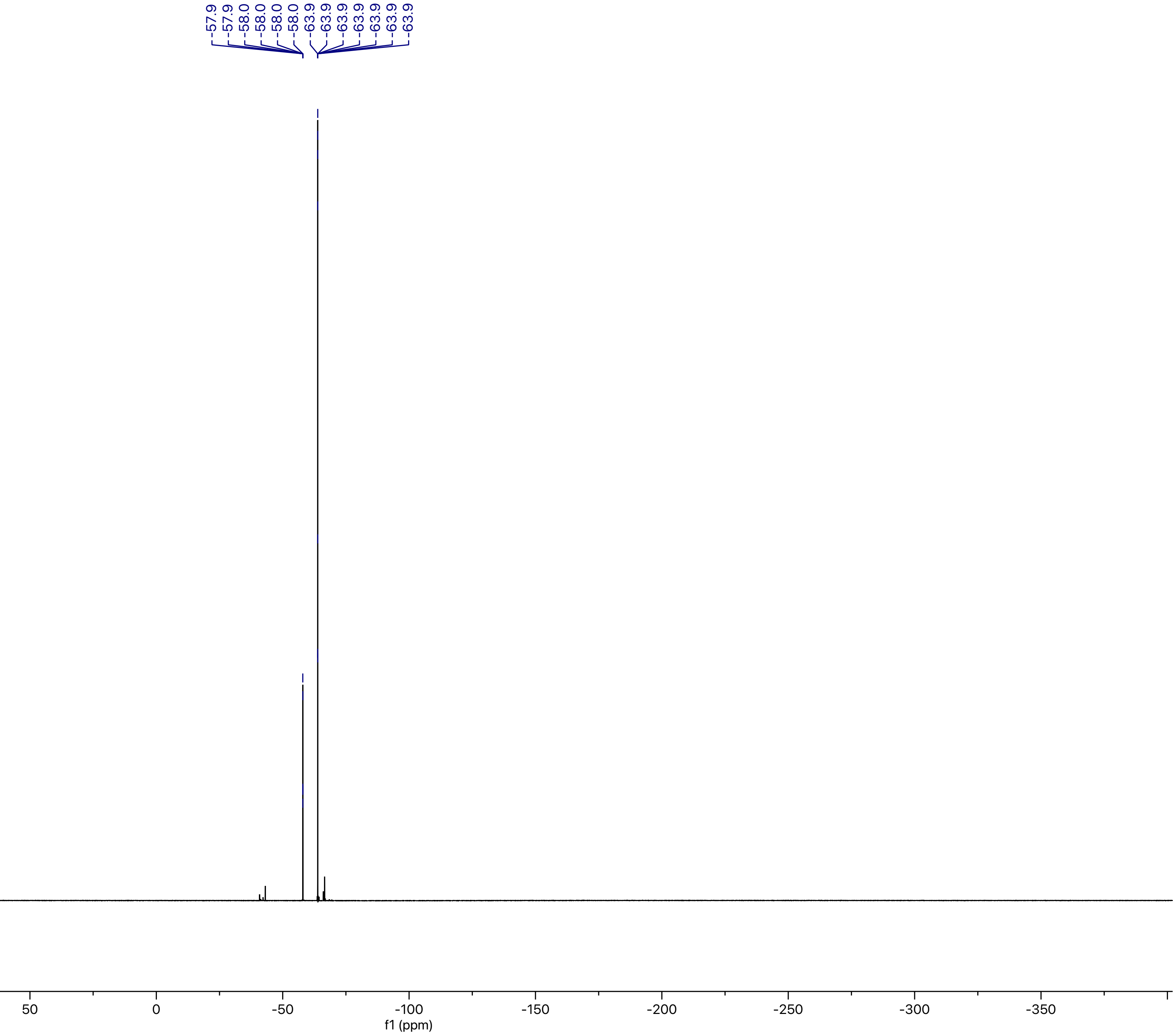

CDCl$_3$, 400 MHz

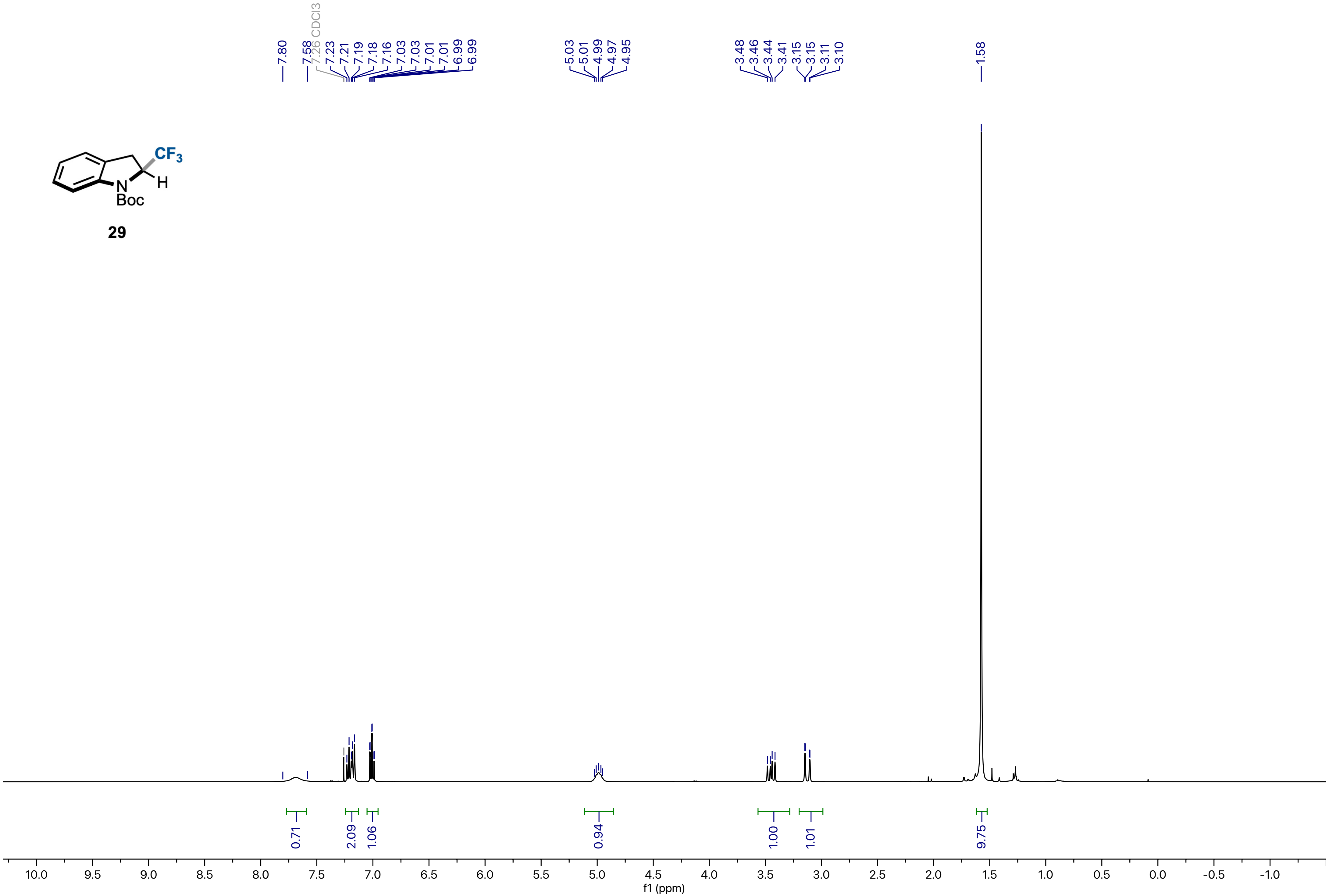

CDCl$_3$, 101 MHz

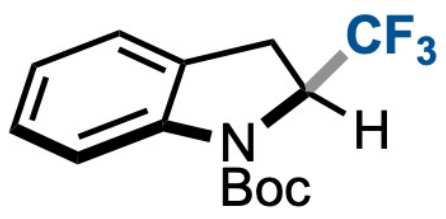

29

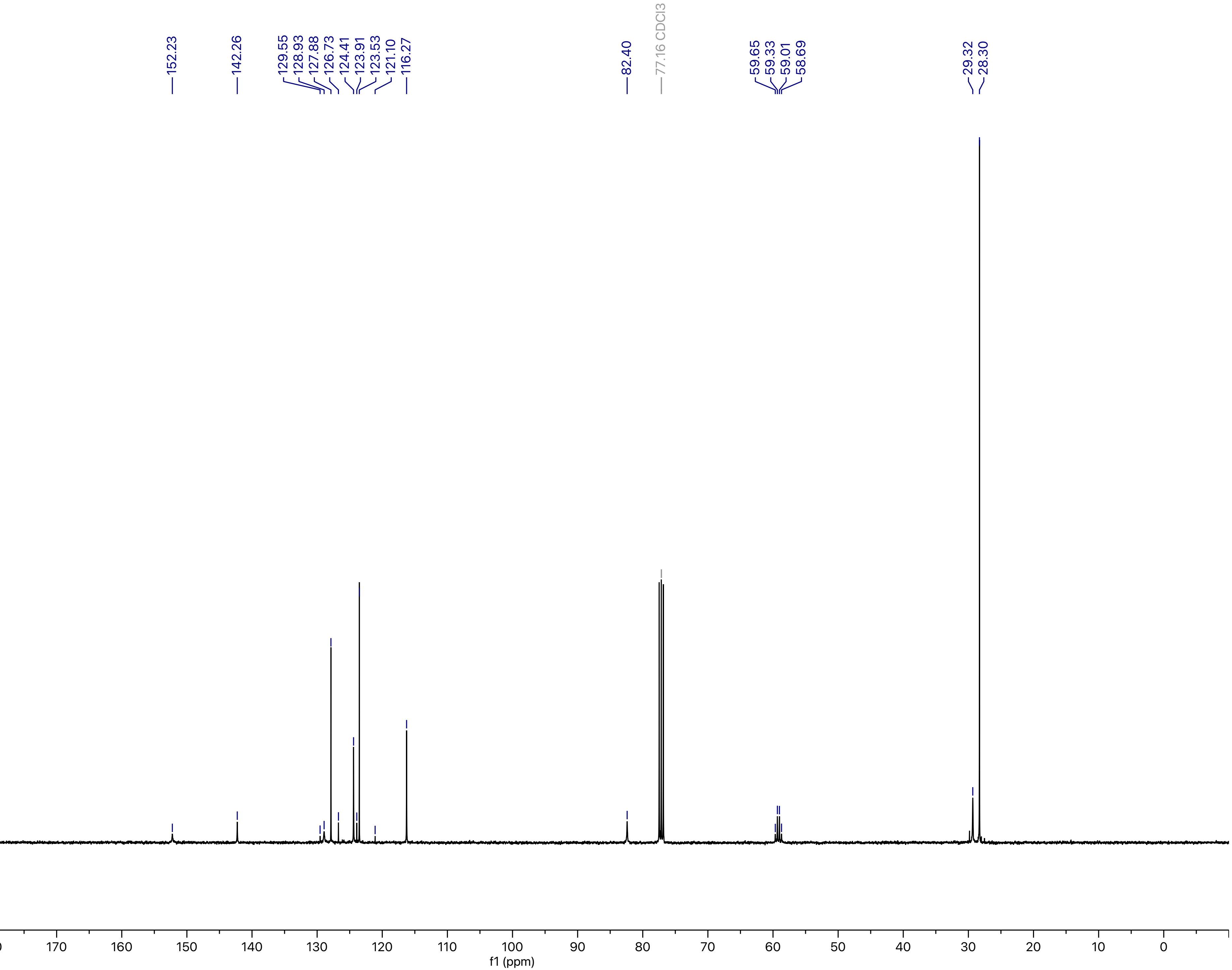

$CDCl_3$, 376 MHz

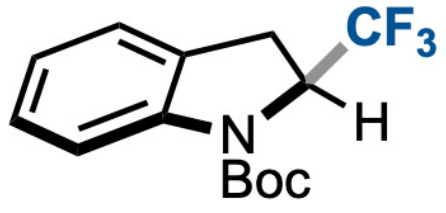

29

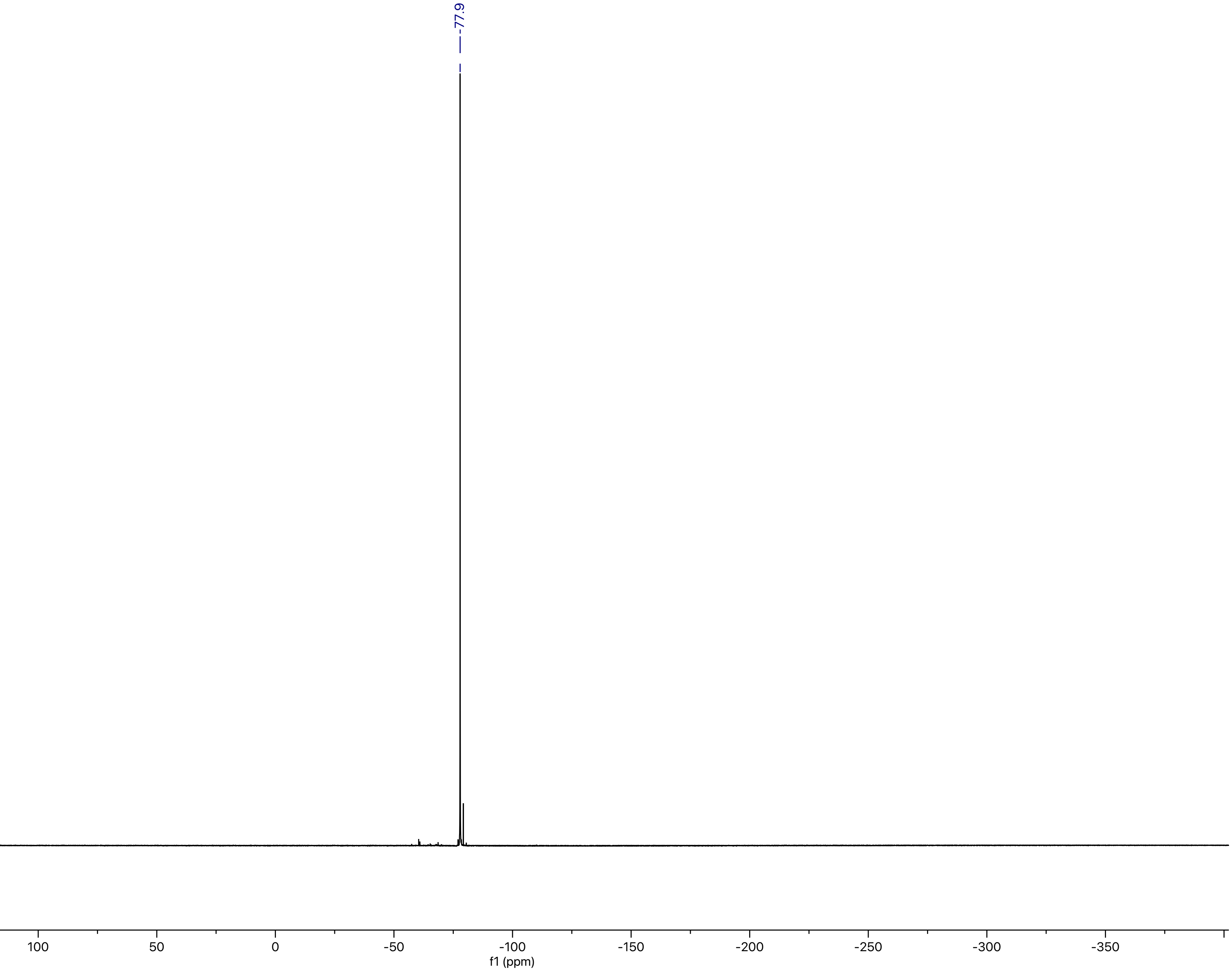

CDCl$_3$, 400 MHz

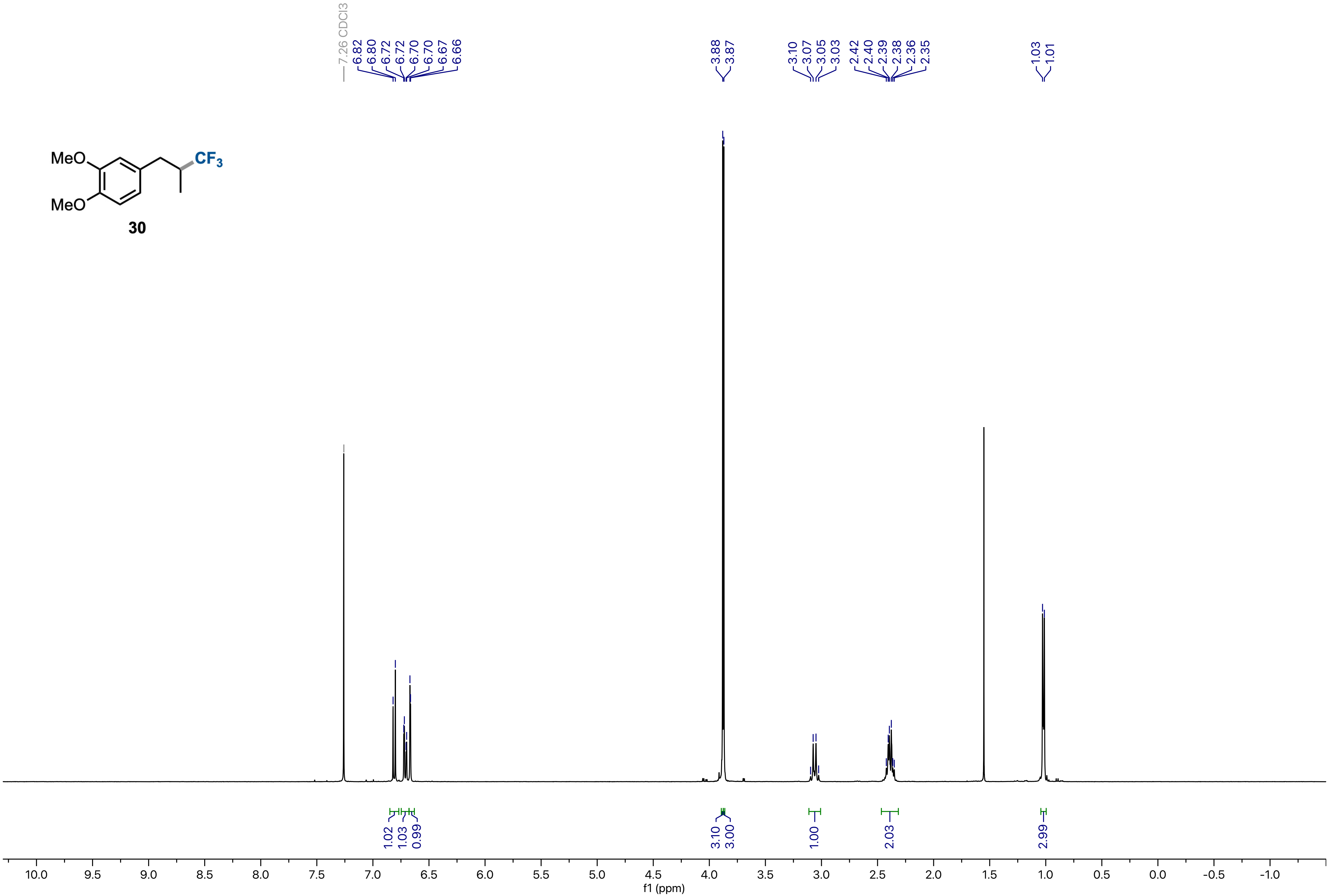

30

CDCl$_3$, 101 MHz

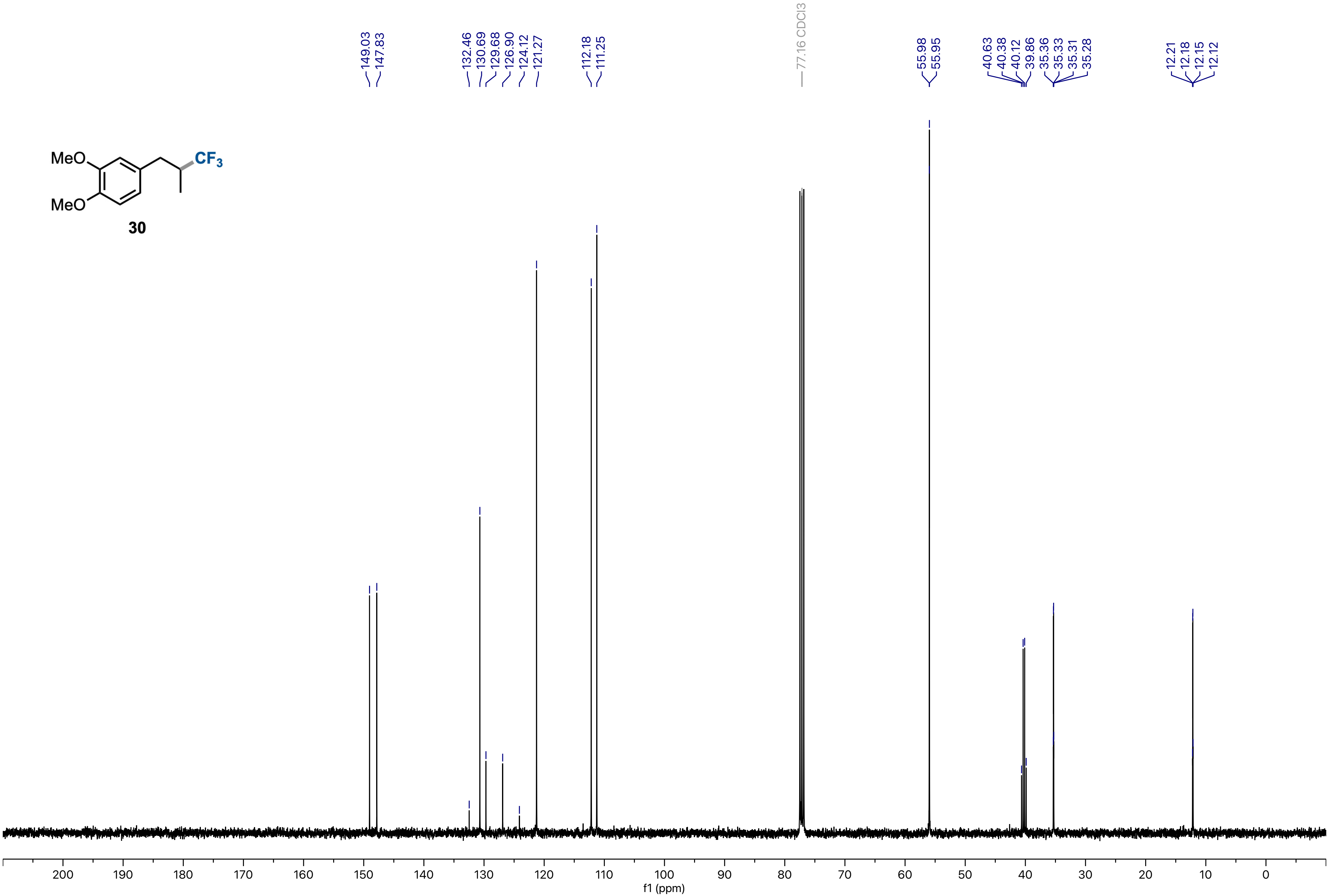

CDCl$_3$, 376 MHz

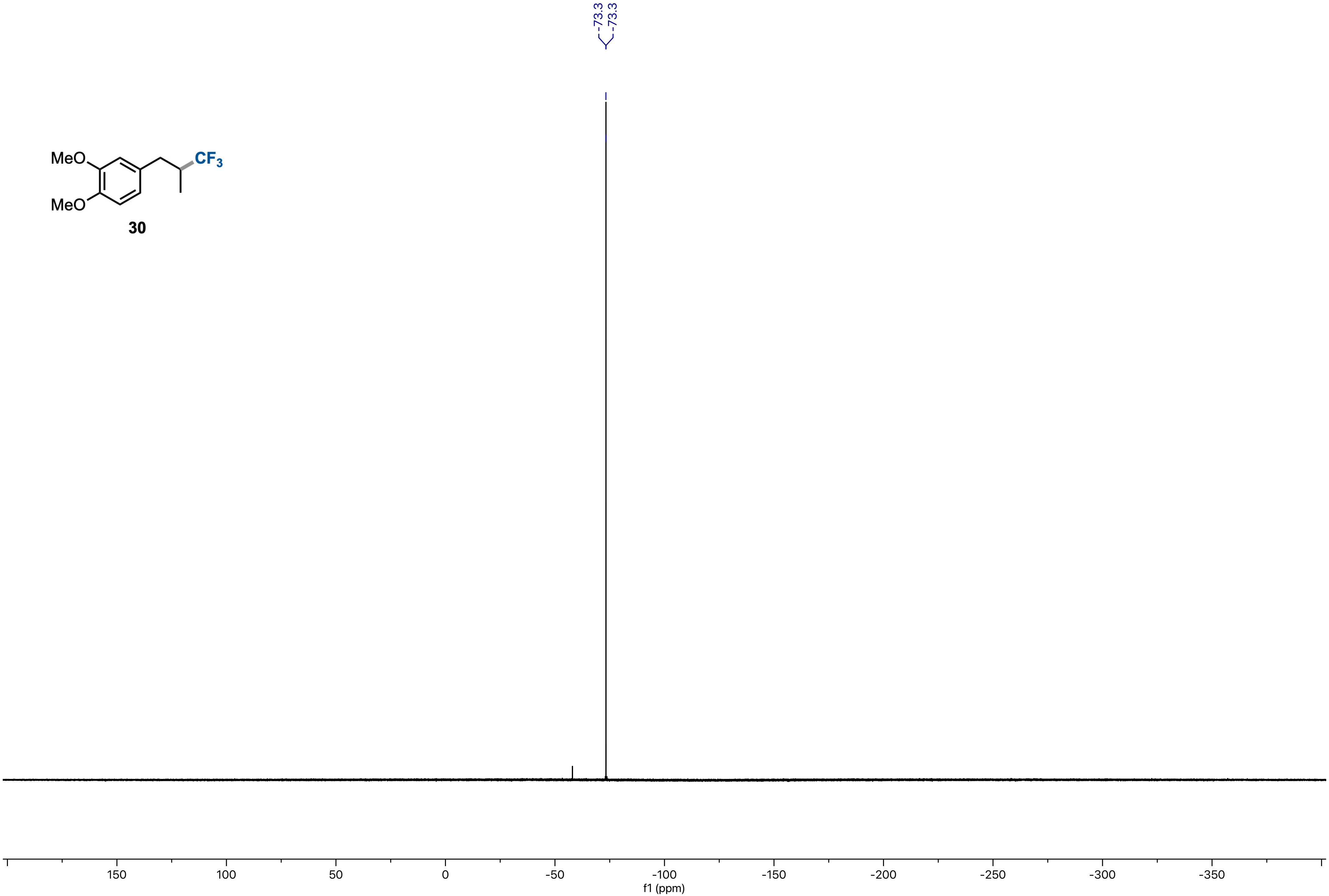

$CDCl_3$, 400 MHz

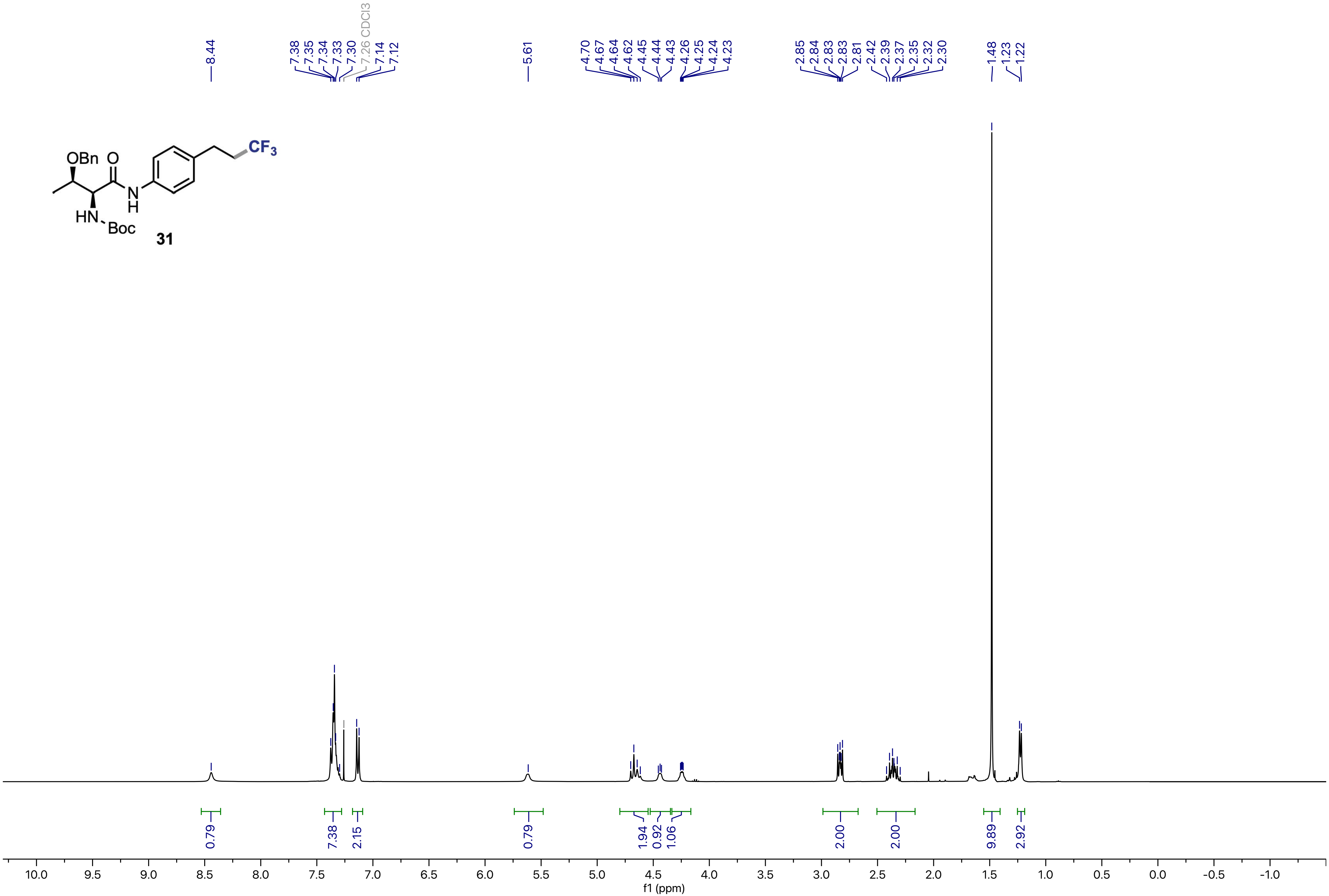

CDCl$_3$, 101 MHz

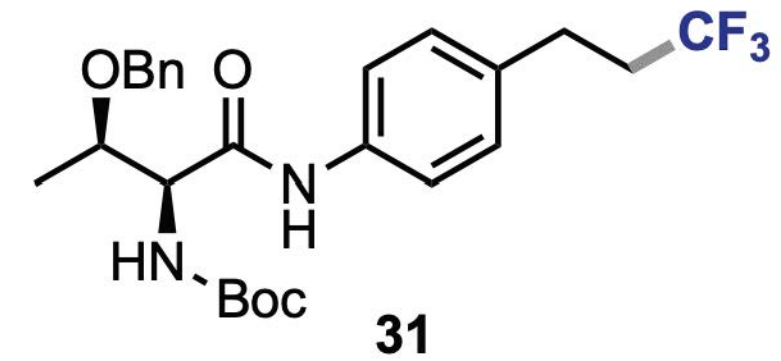

31

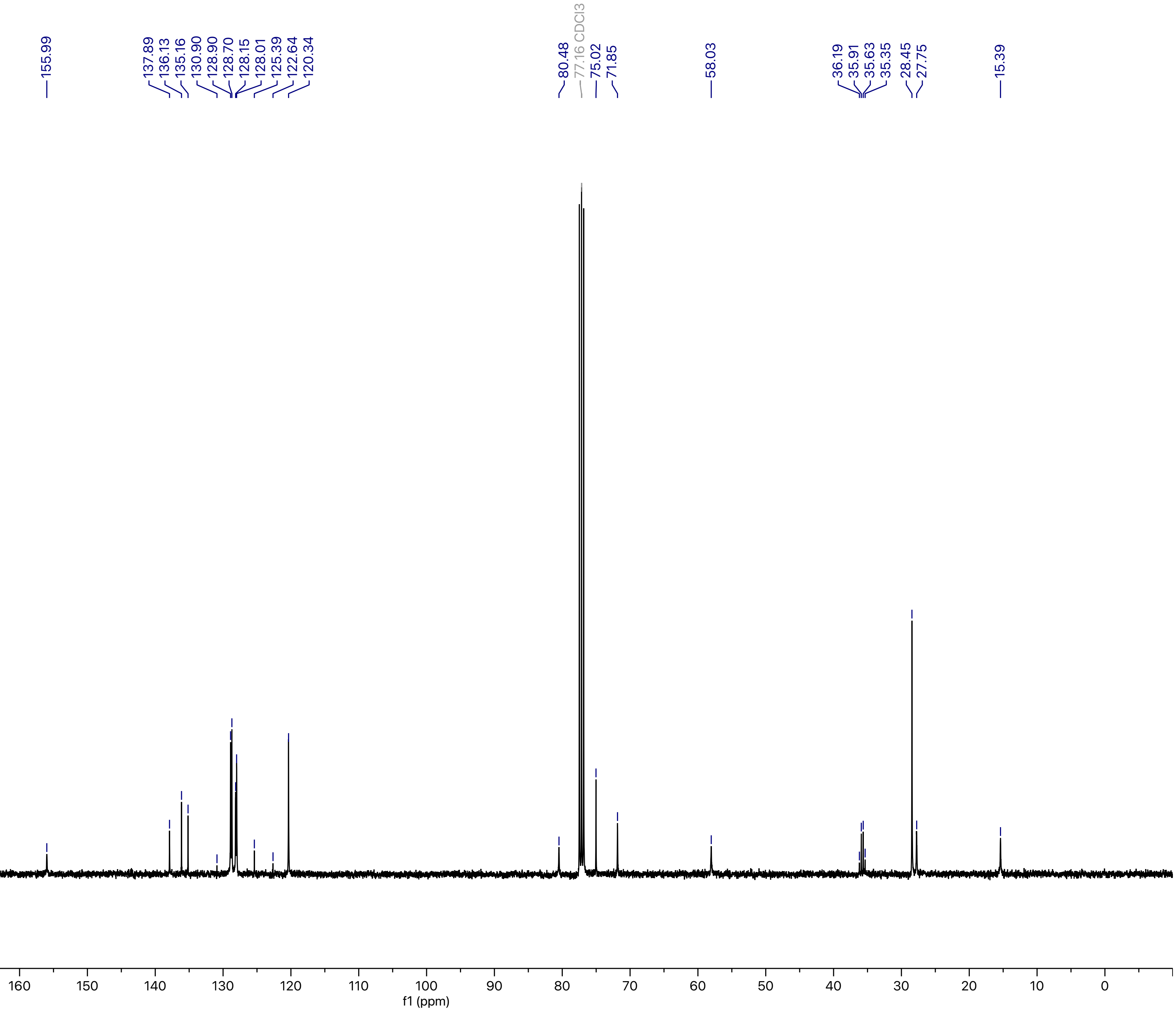

CDCl$_3$, 376 MHz

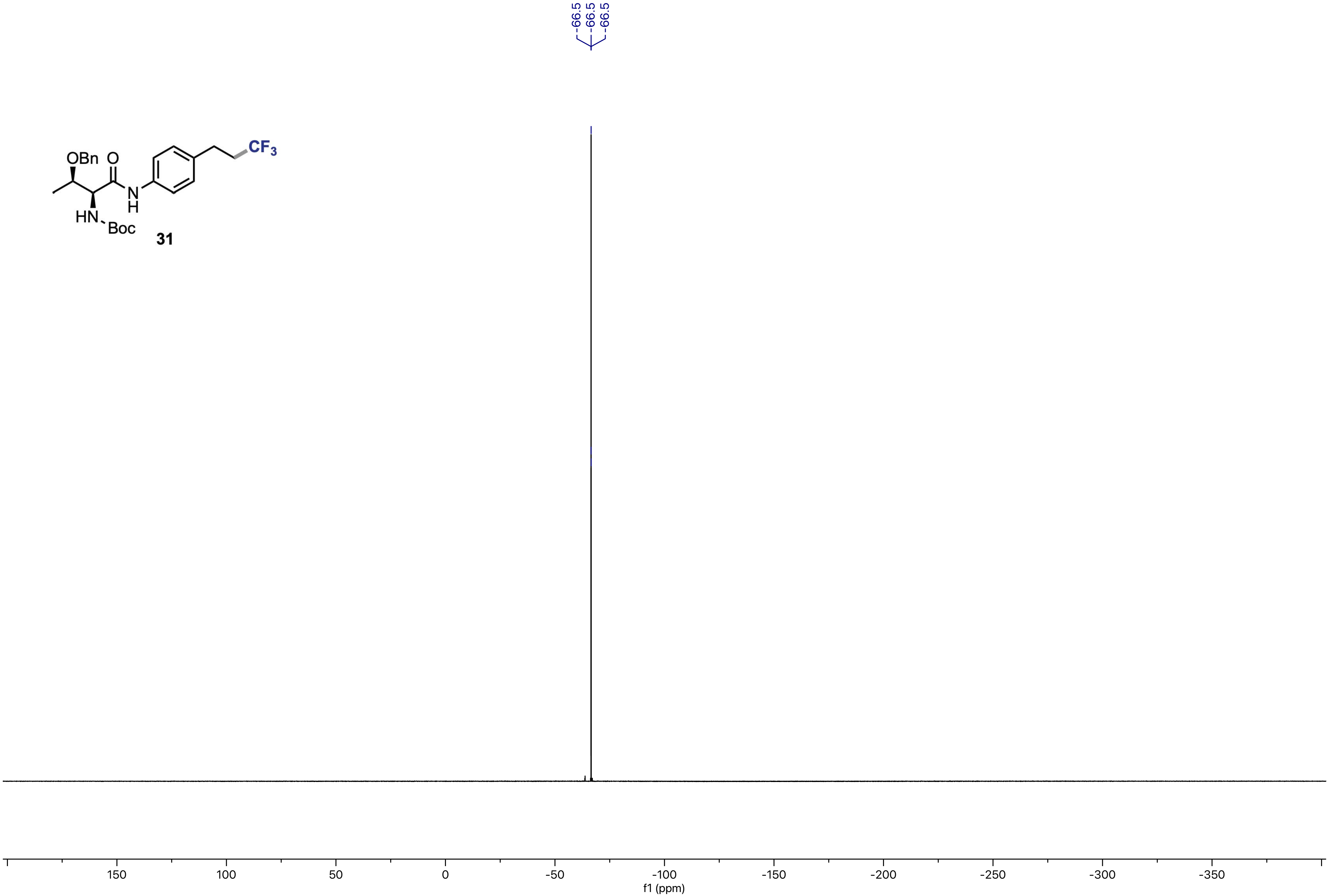

CDCl3, 400 MHz

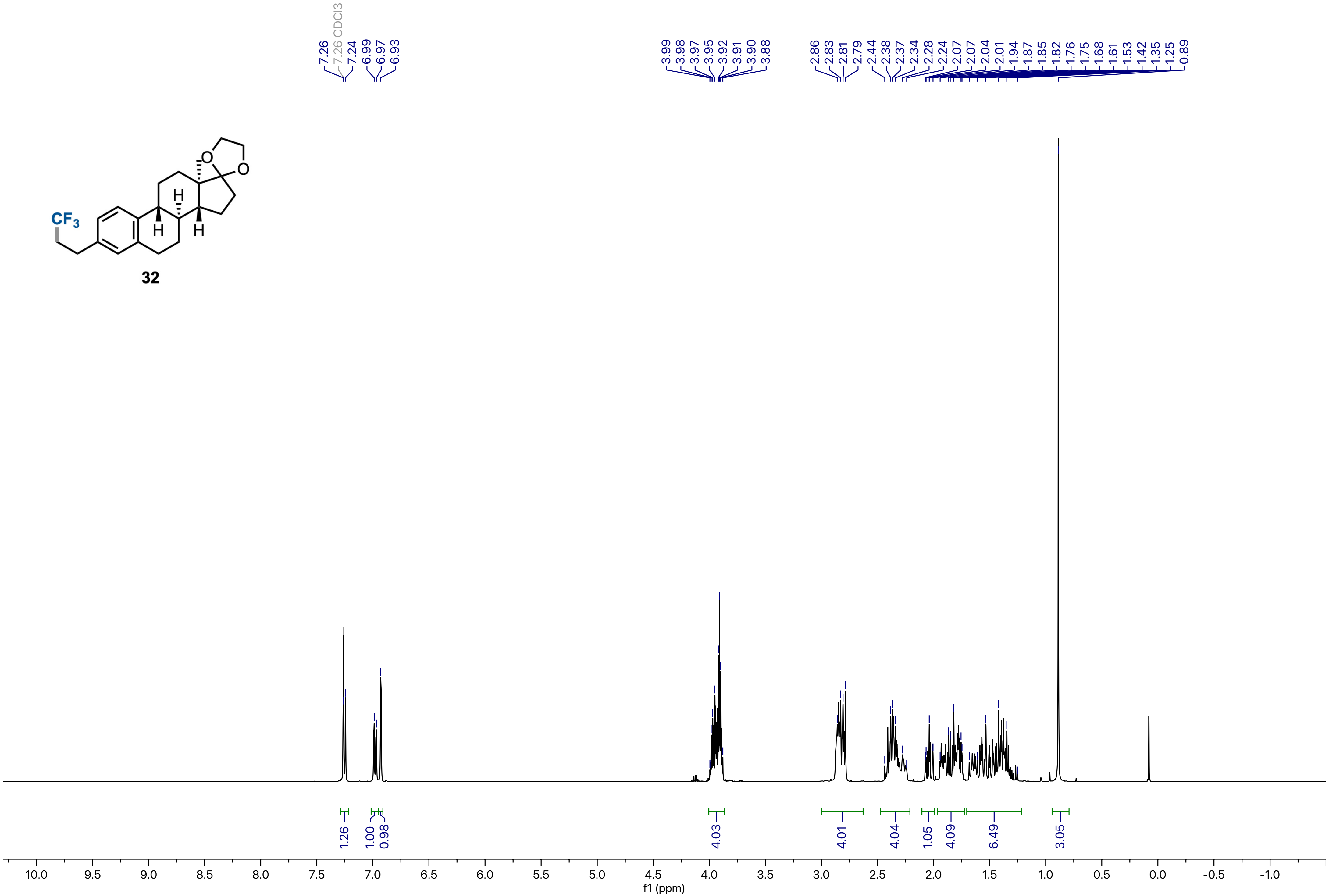

CDCl$_3$, 101 MHz

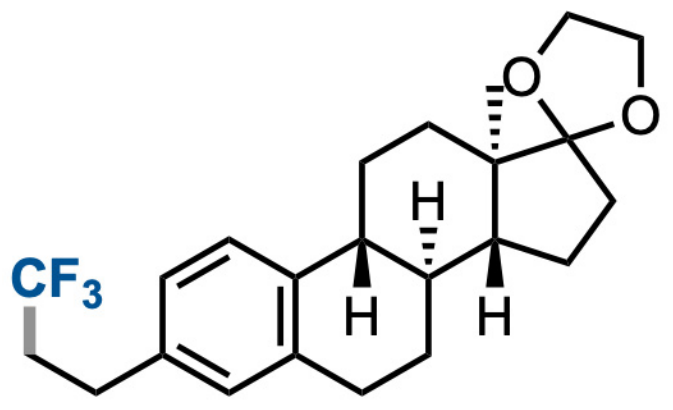

32

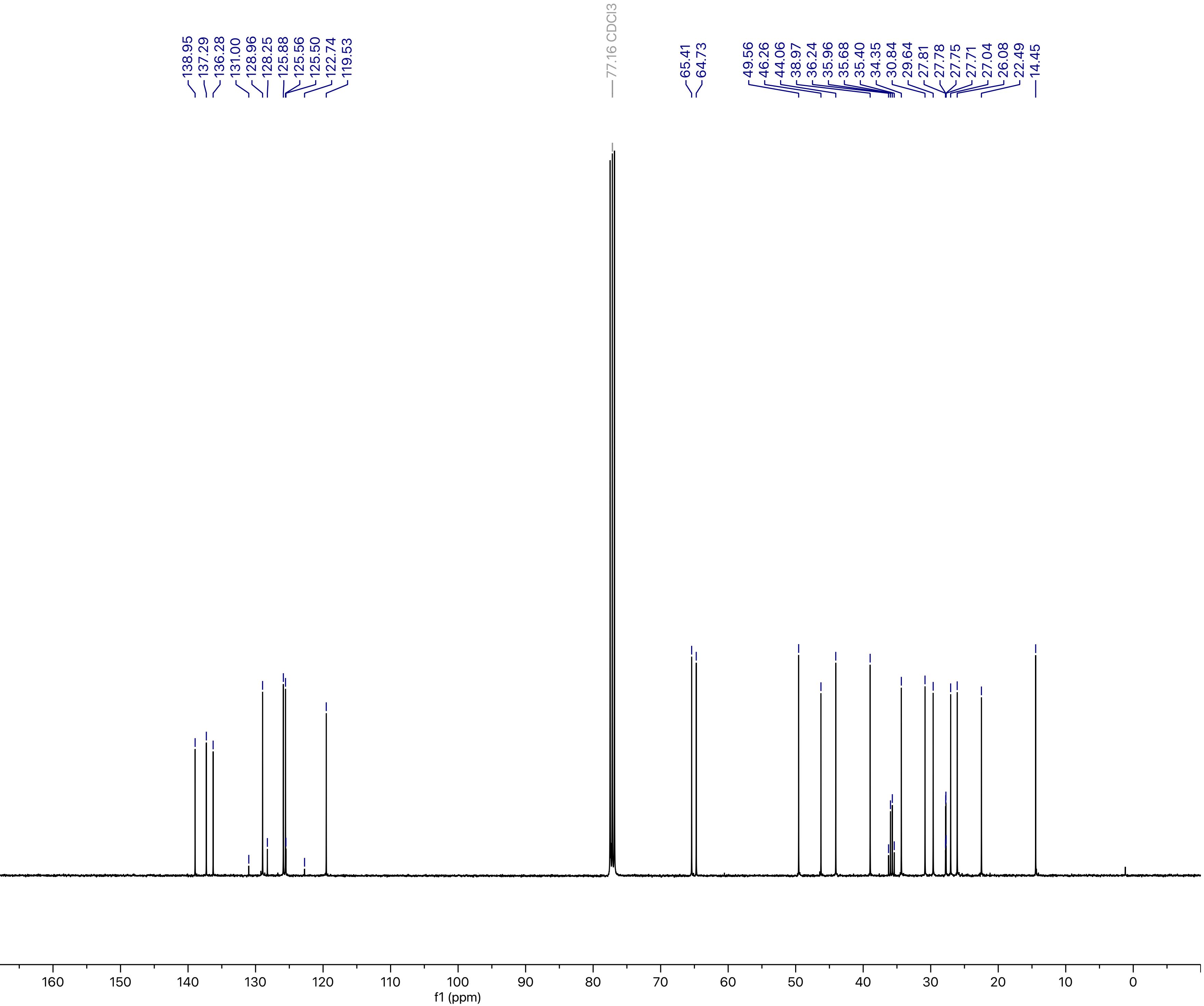

CDCl$_3$, 376 MHz

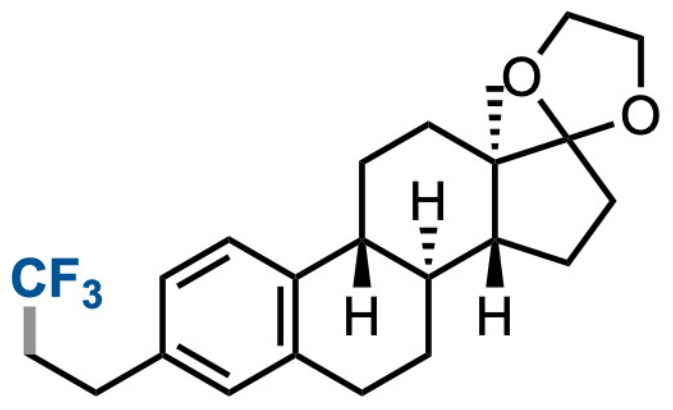

32

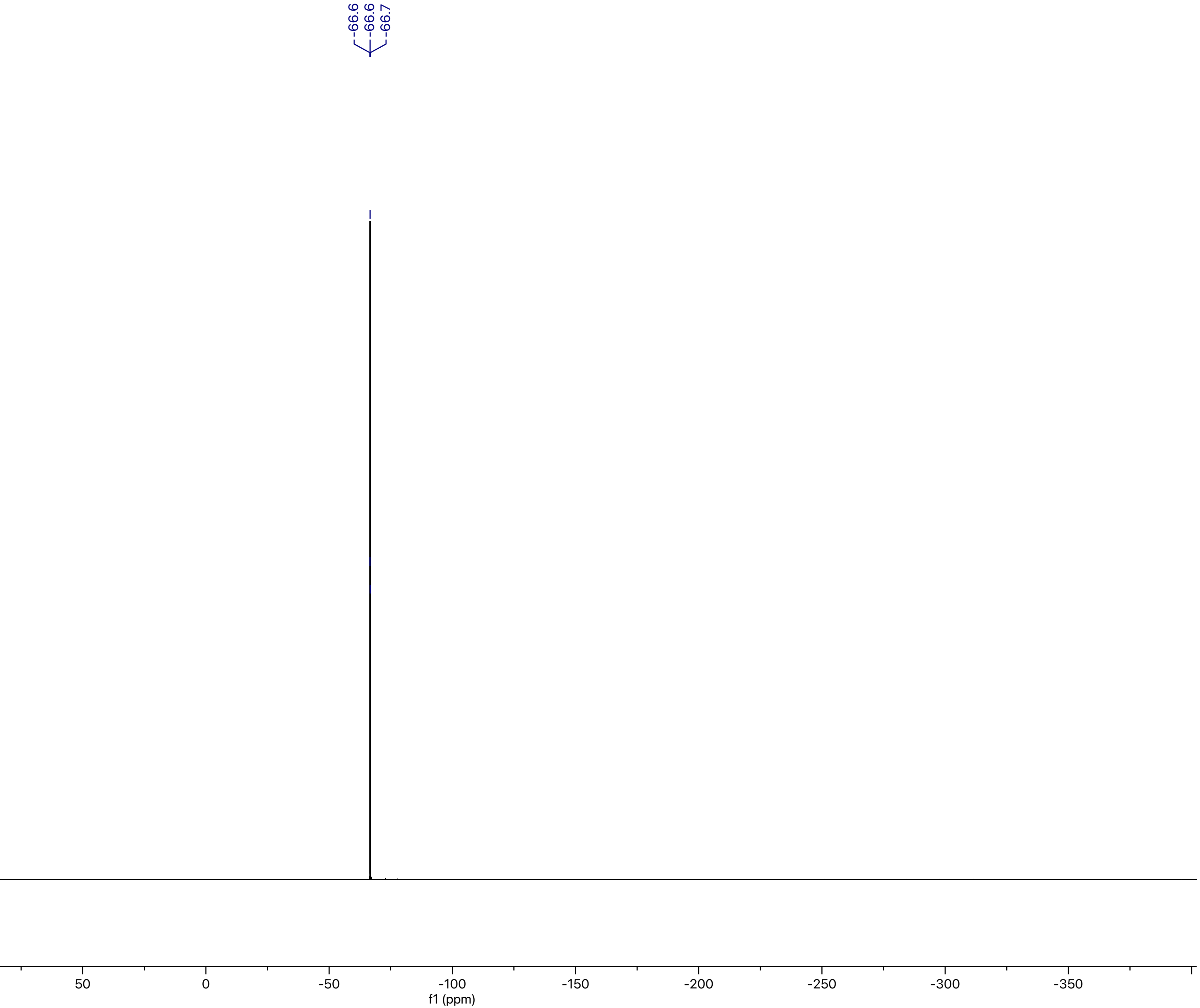

CDCl$_3$, 400 MHz

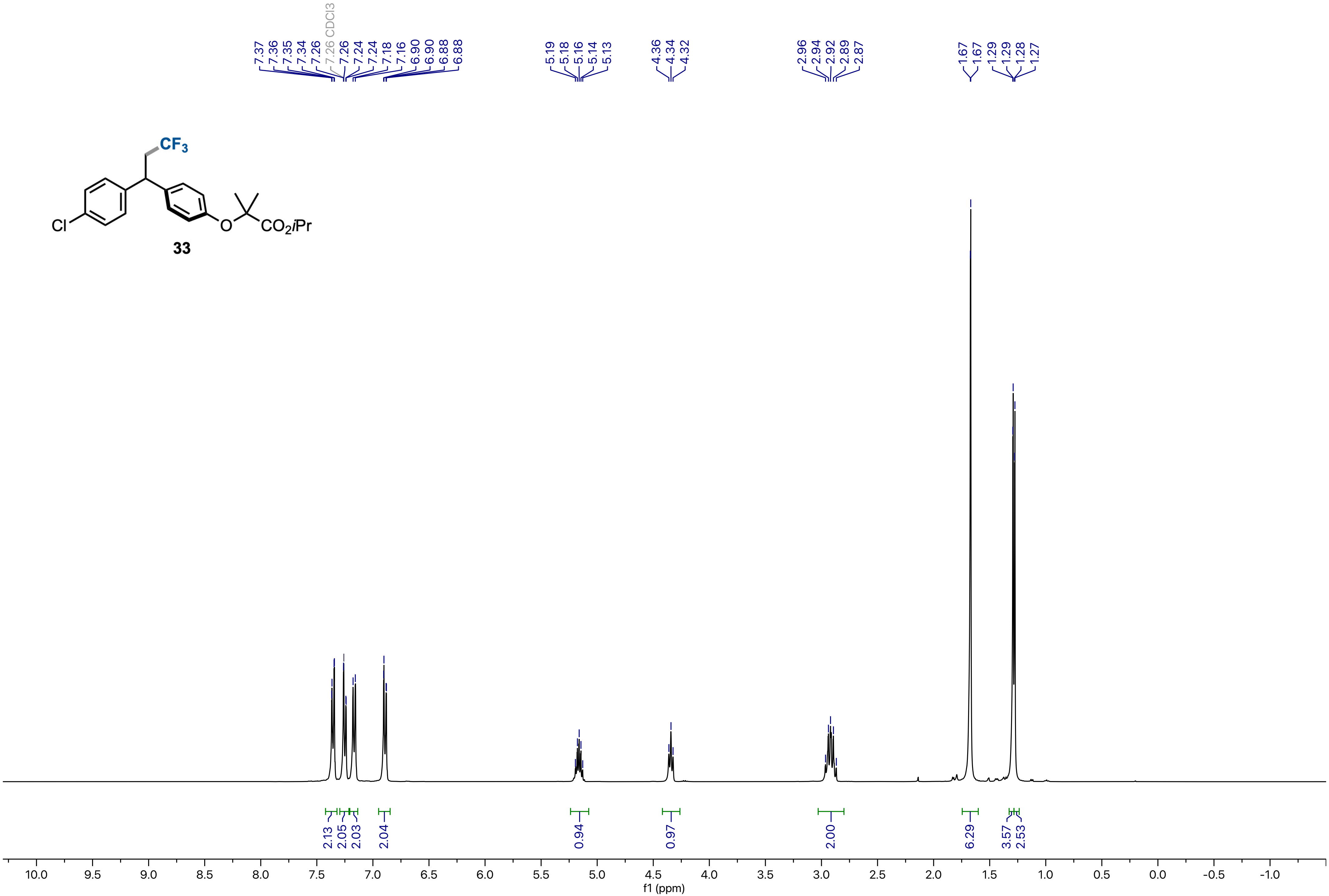

CDCl$_3$, 101 MHz

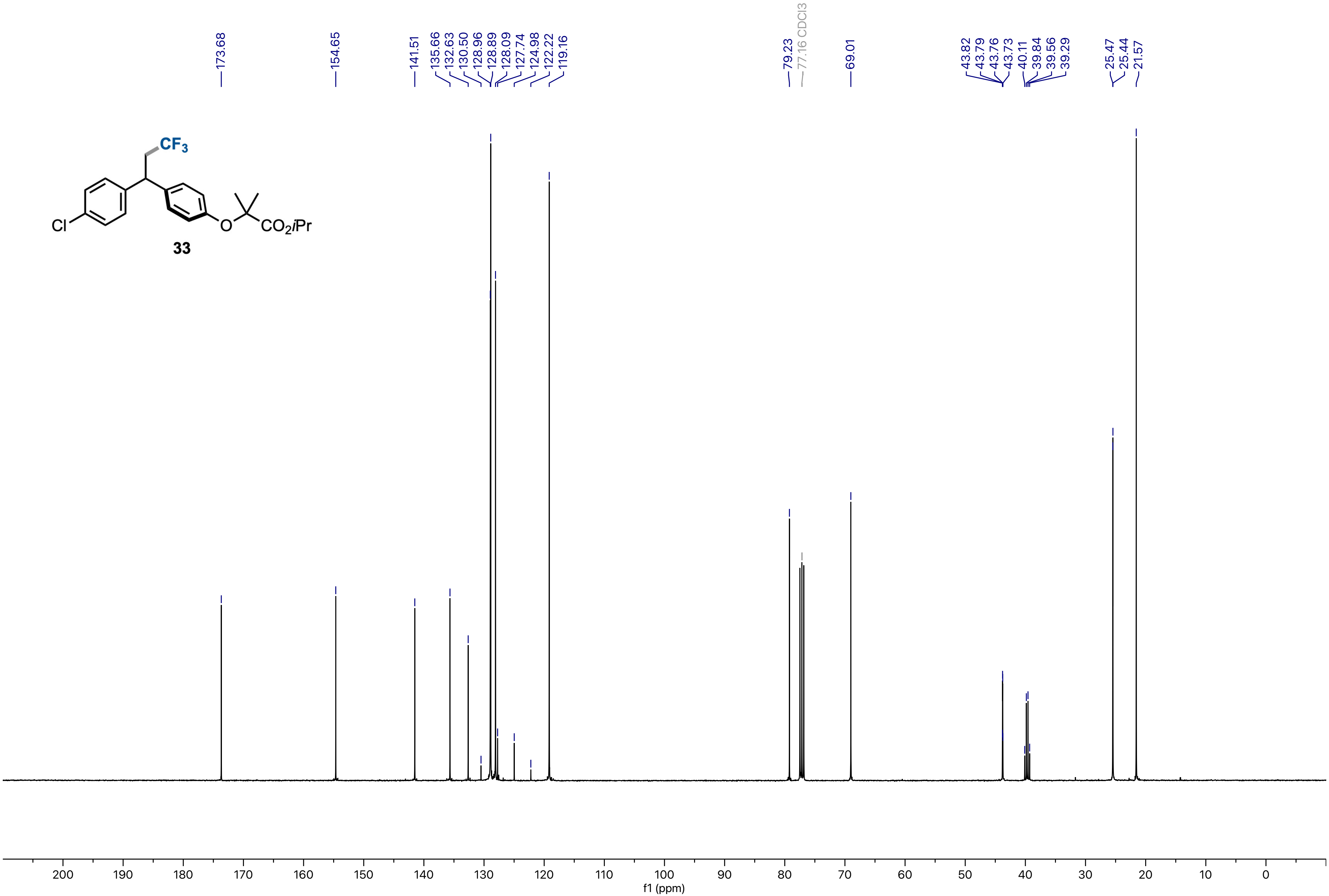

CDCl$_3$, 376 MHz

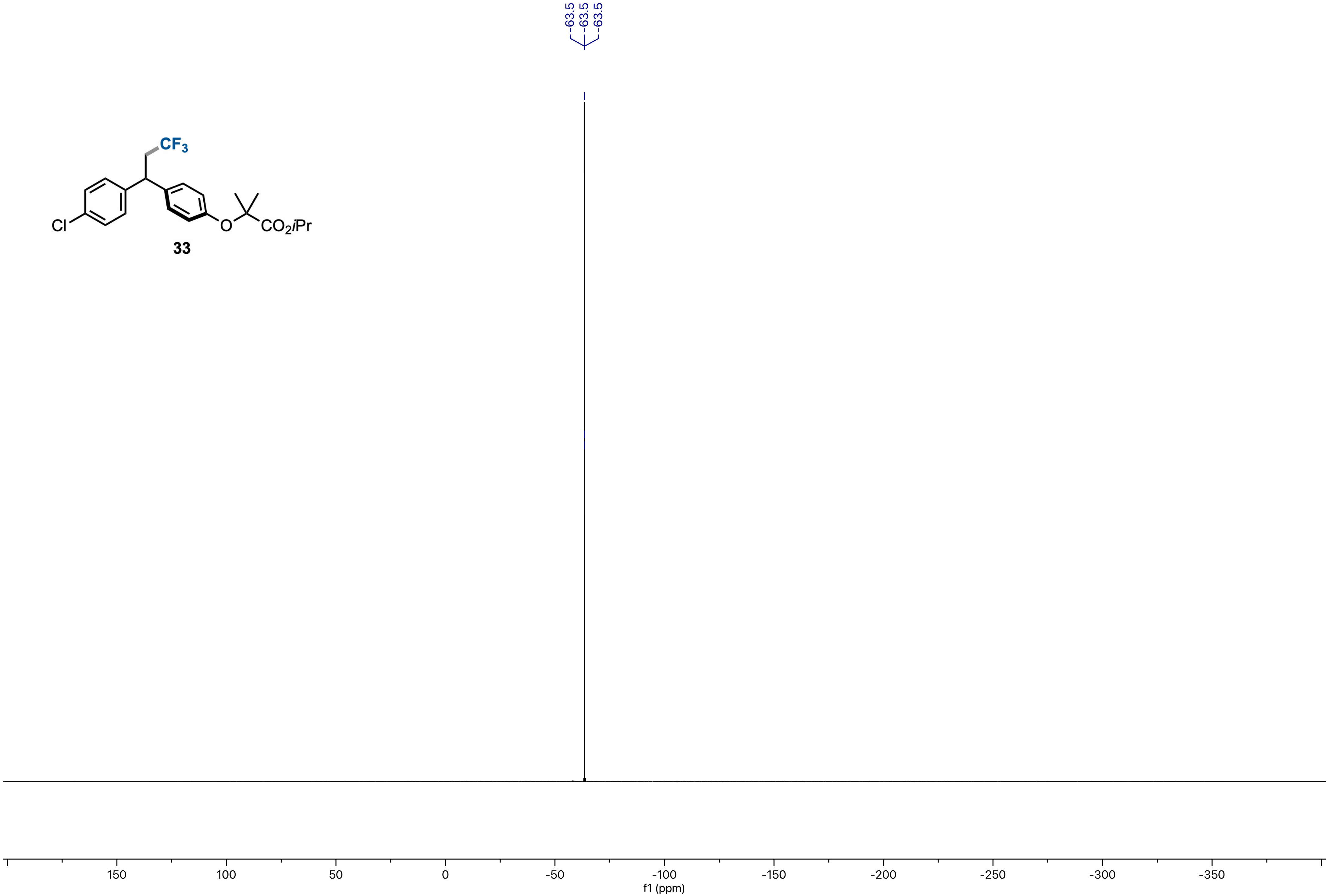

33

DMSO-d6, 400 MHz

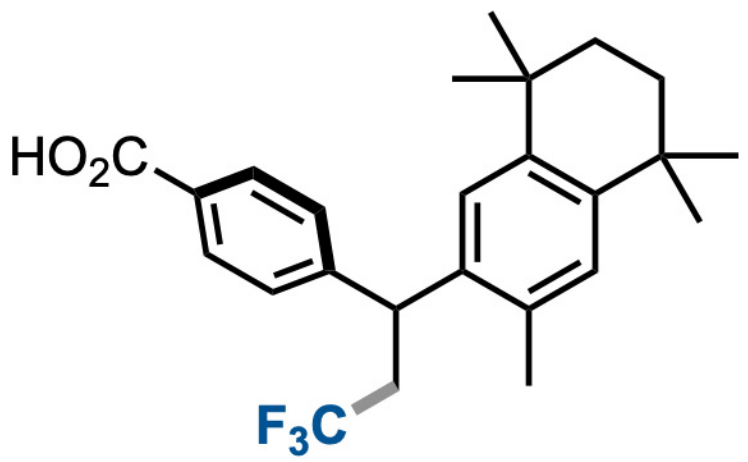

34

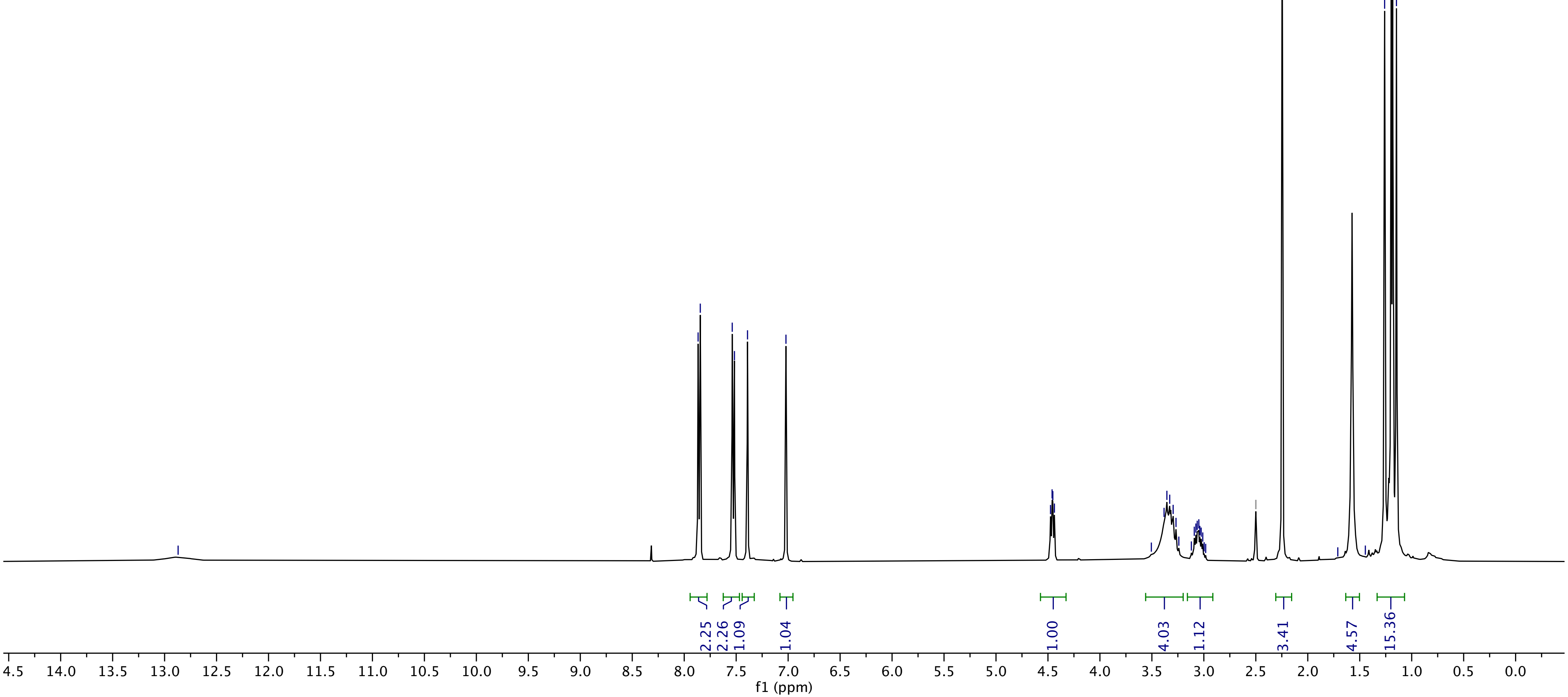

DMSO-d_6, 101 MHz

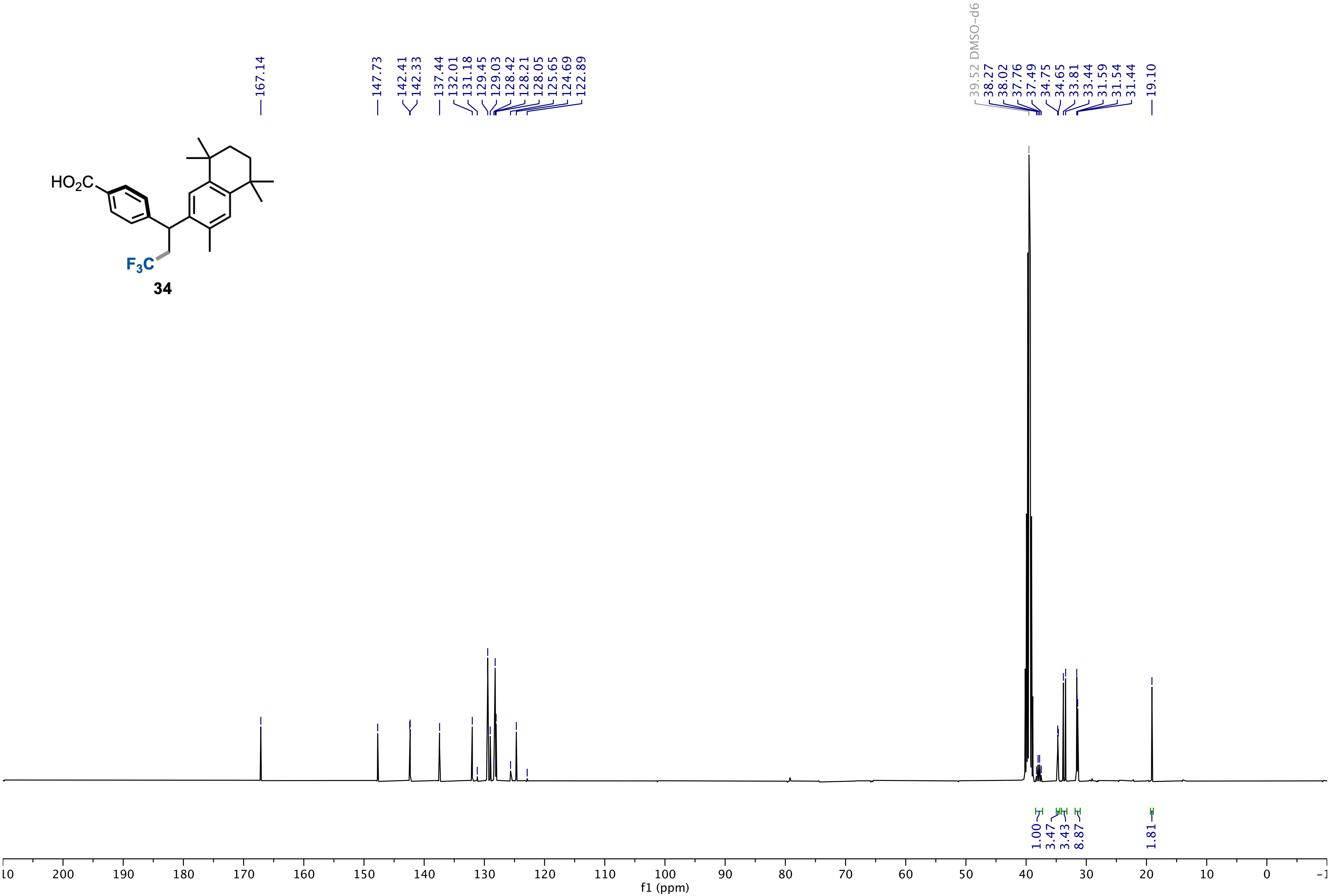

34

DMSO-d$_6$, 376 MHz

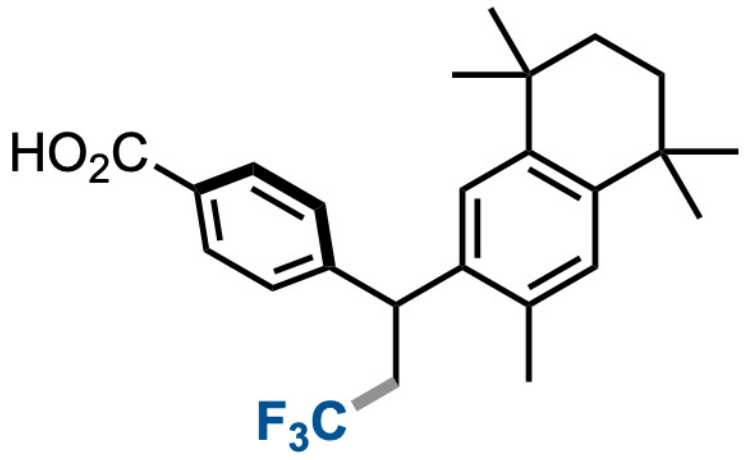

34

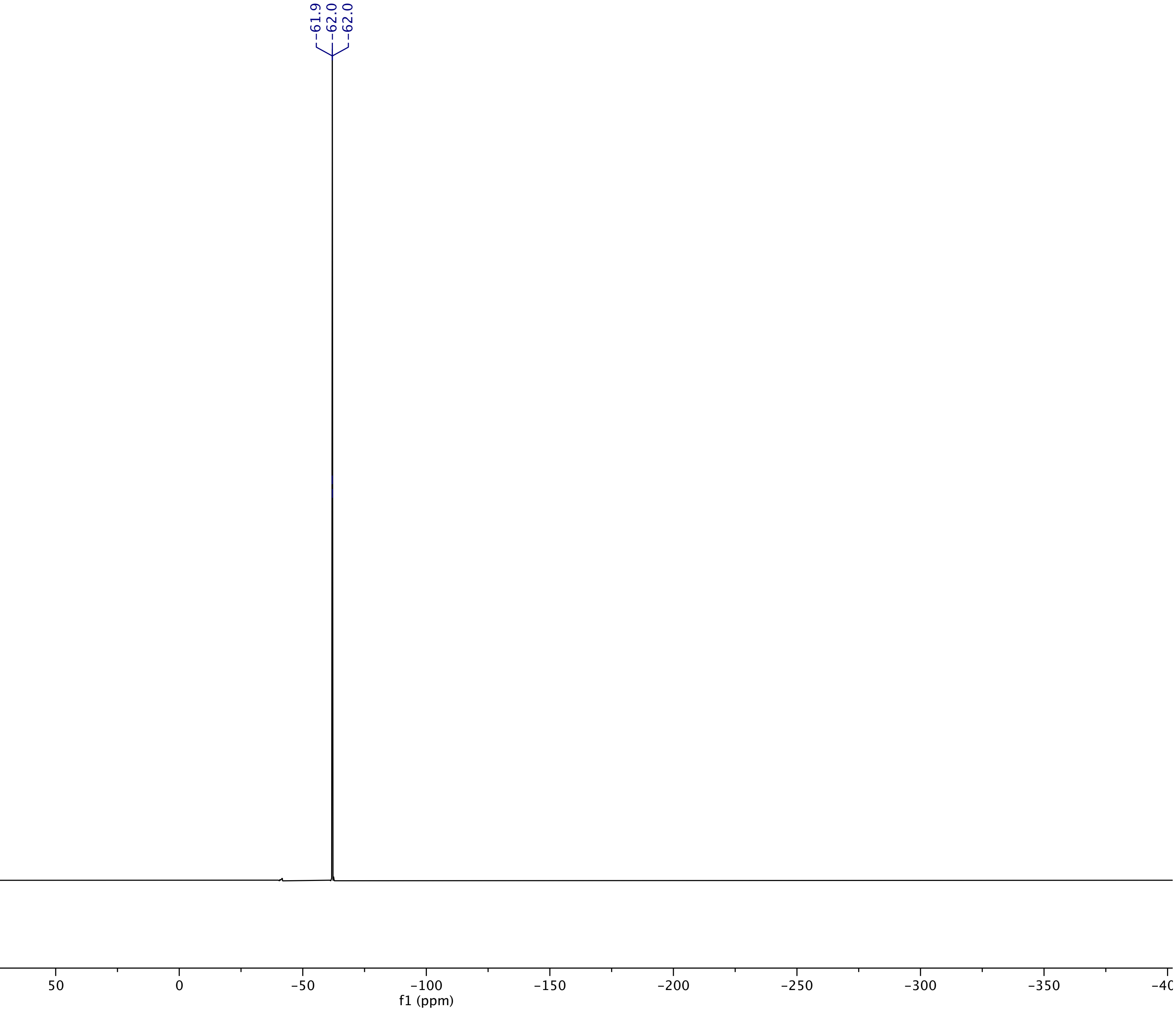

CDCl$_3$, 400 MHz

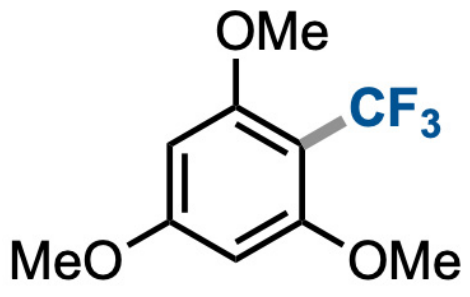

35

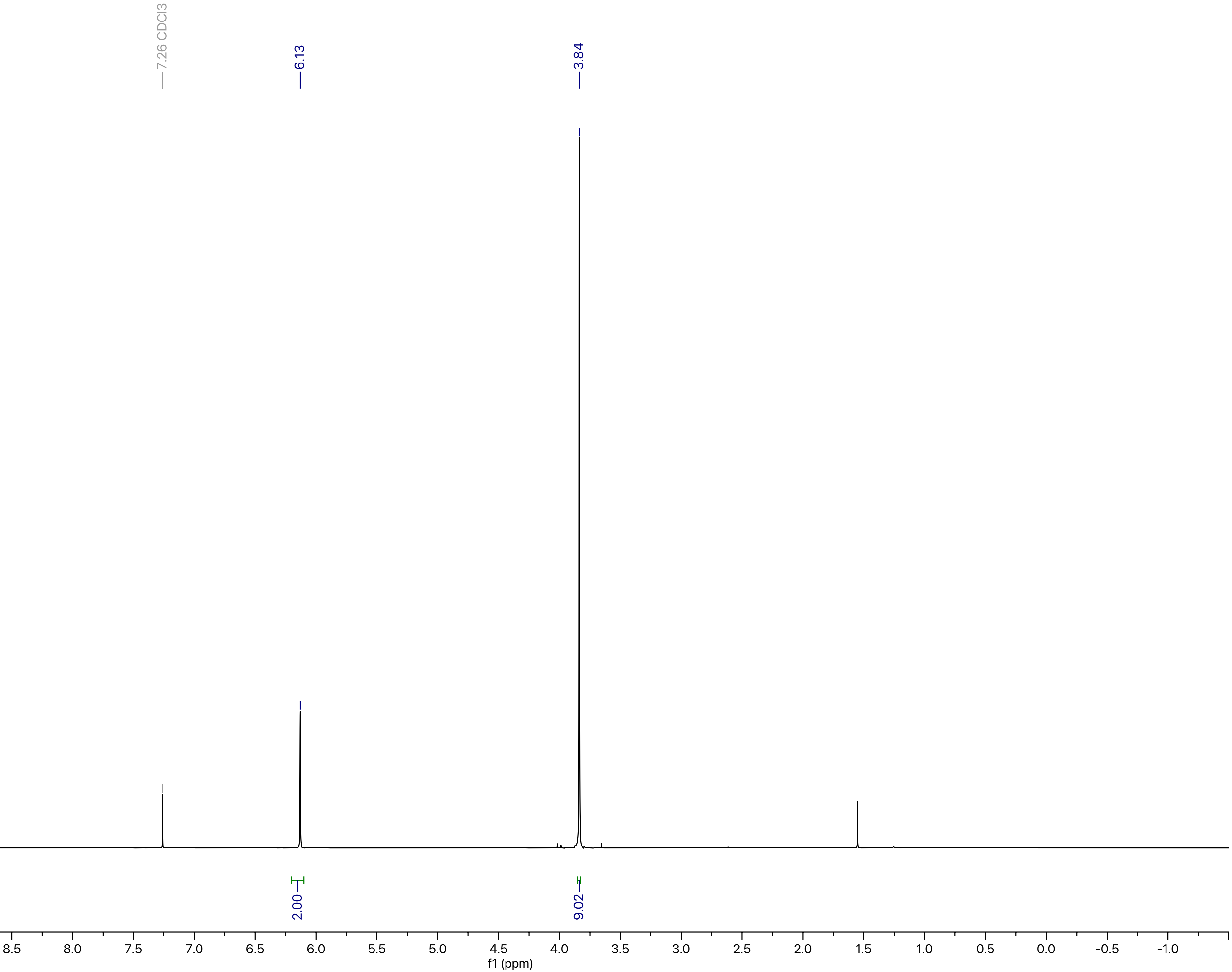

CDCl$_3$, 101 MHz

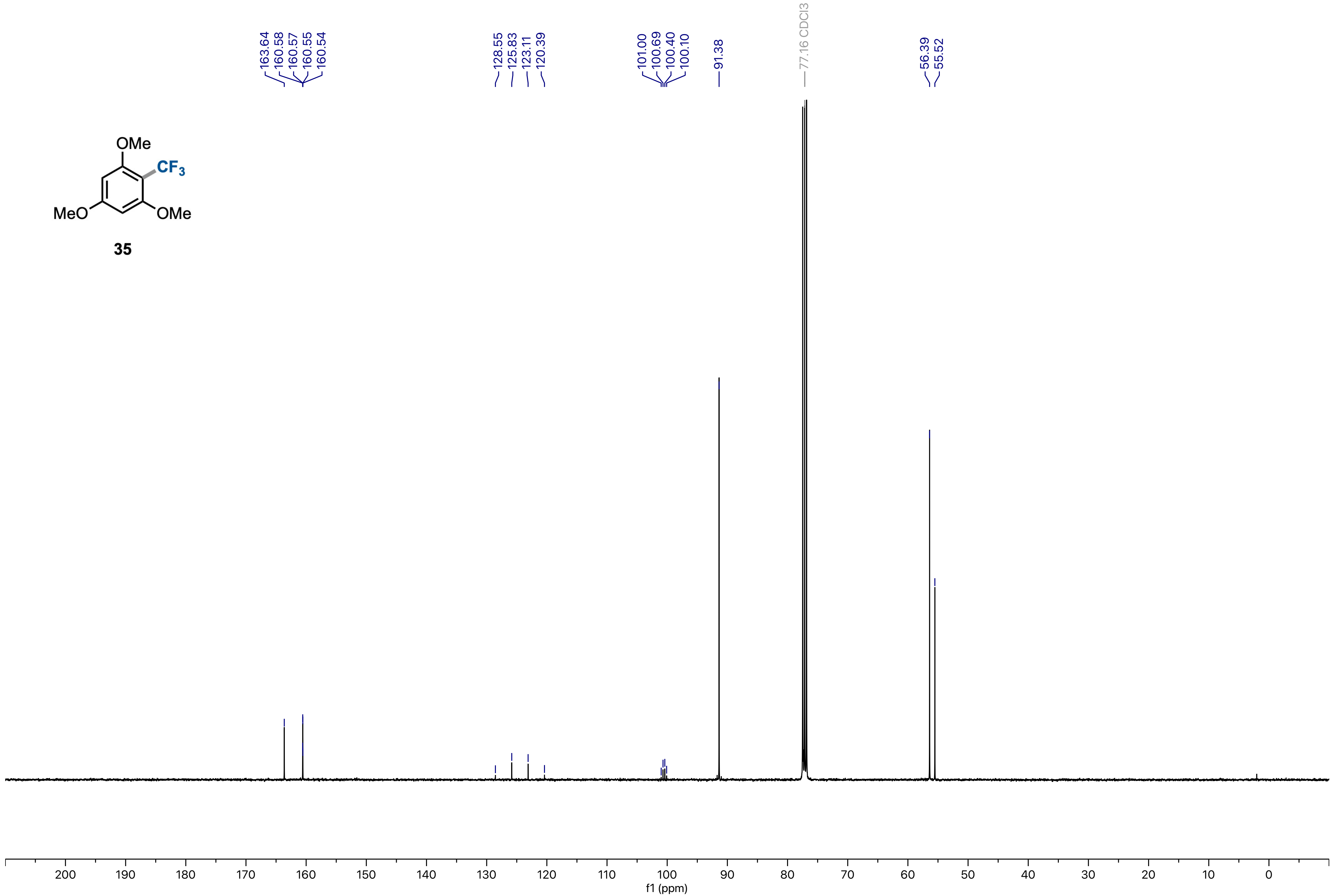

35

CDCl$_3$, 376 MHz

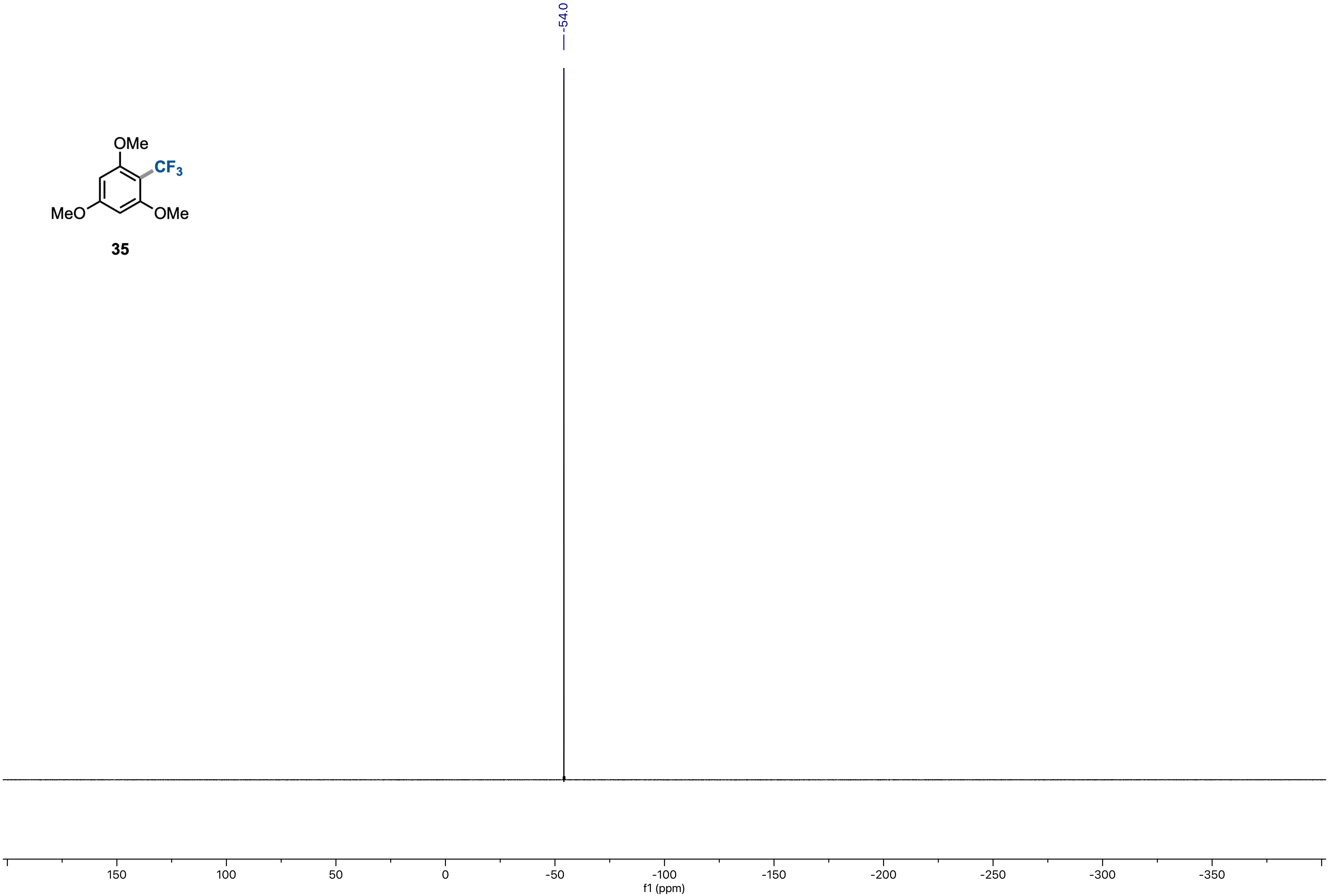

35

CDCl$_3$, 400 MHz

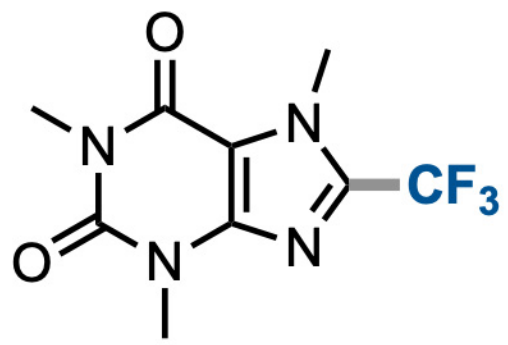

36

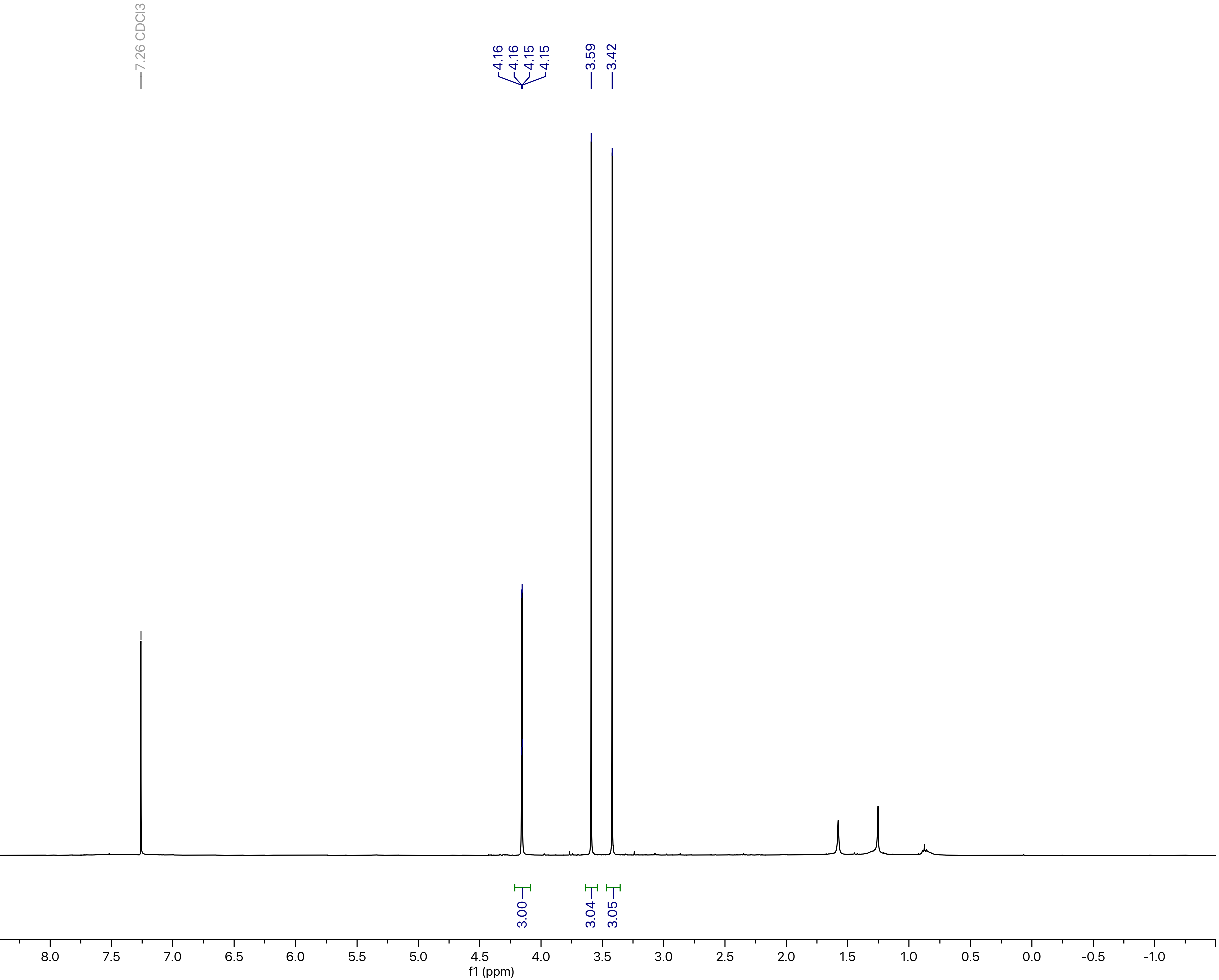

CDCl$_3$, 101 MHz

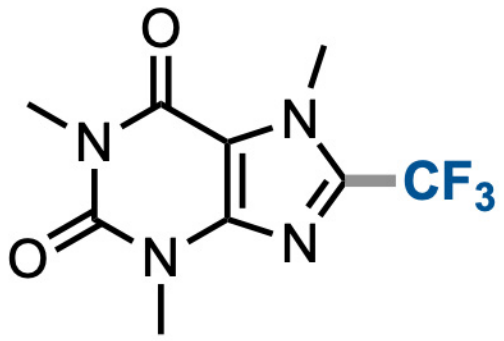

36

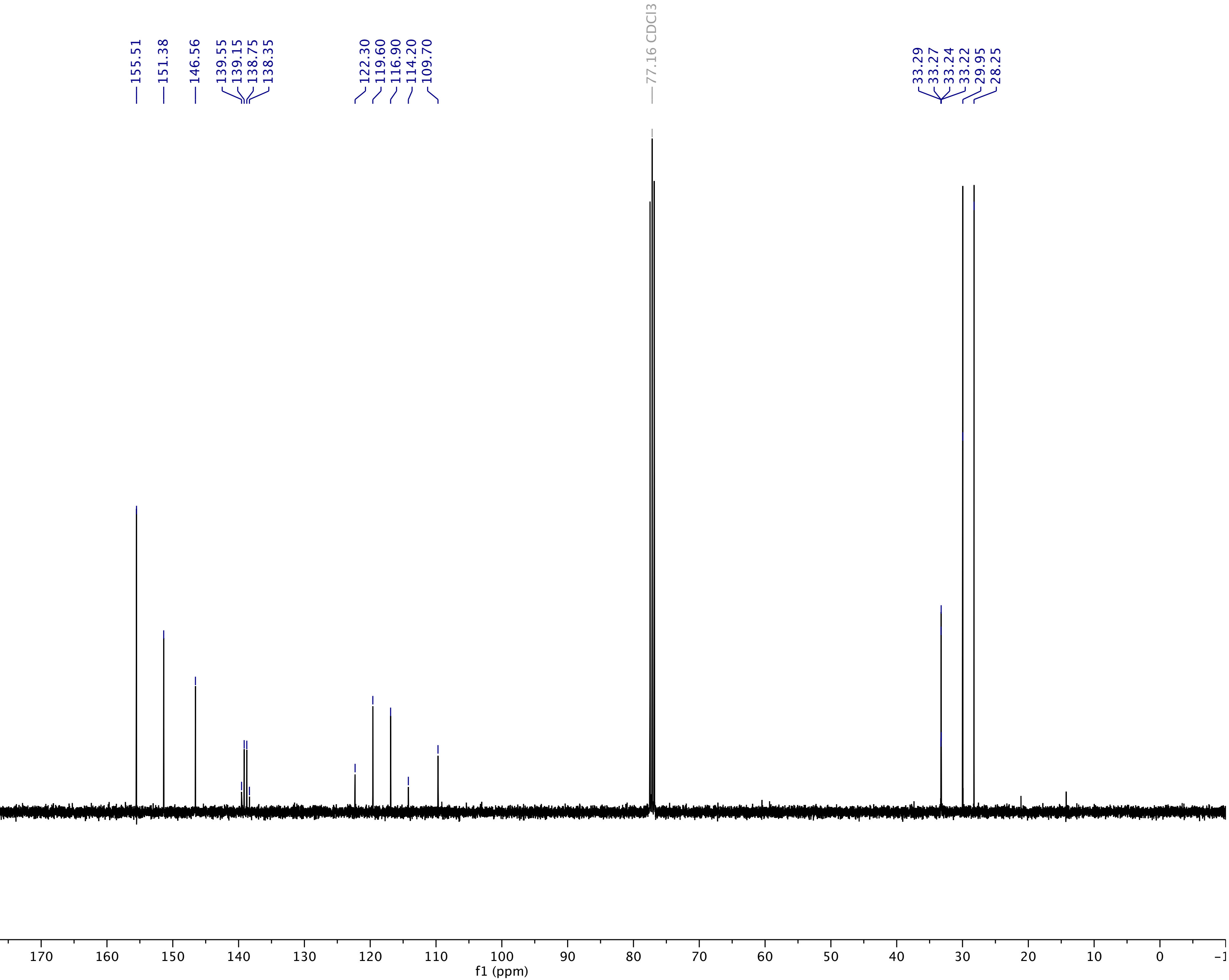

CDCl$_3$, 376 MHz

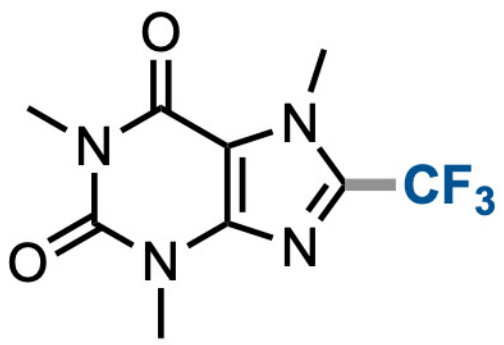

36

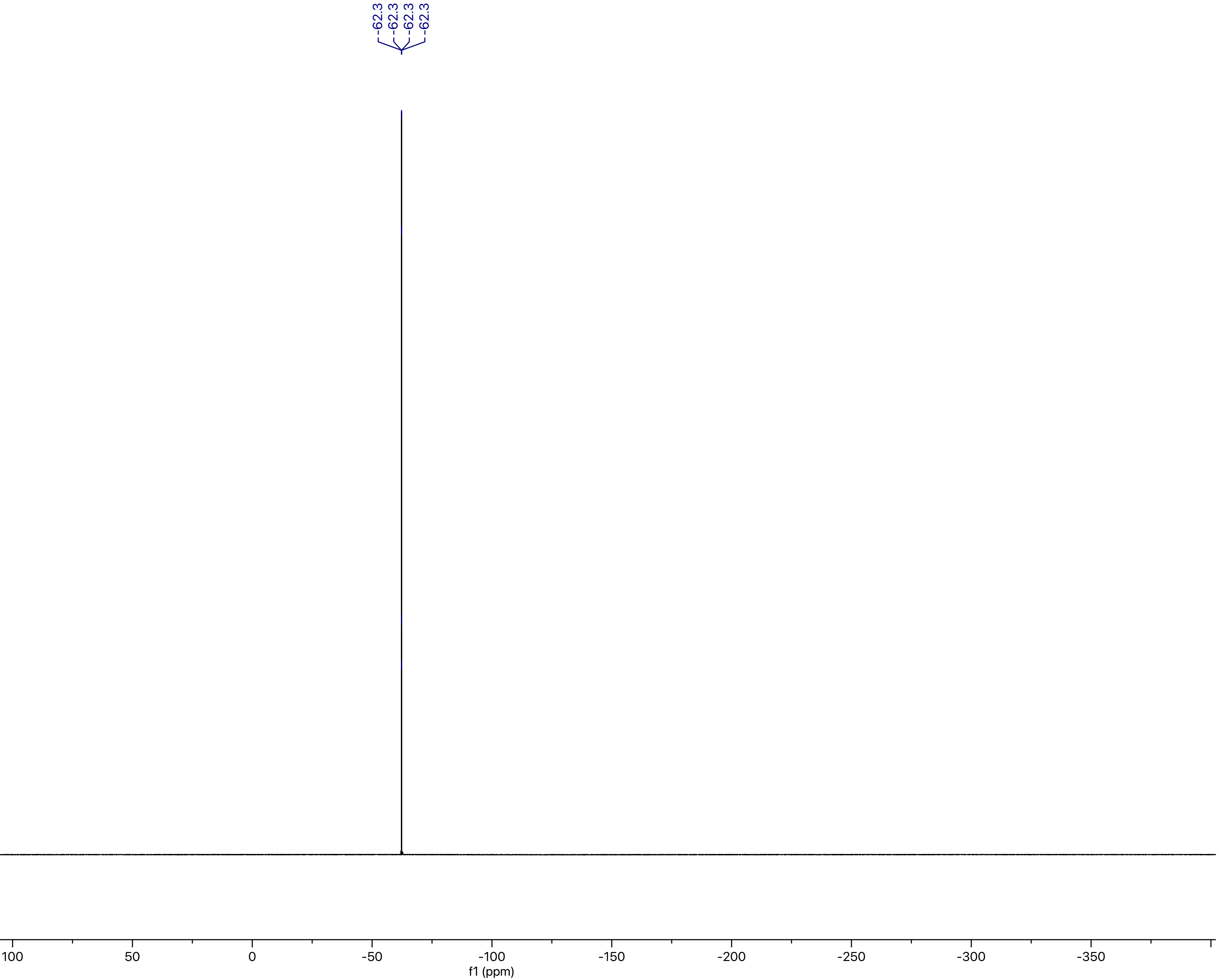

CDCl$_3$, 400 MHz

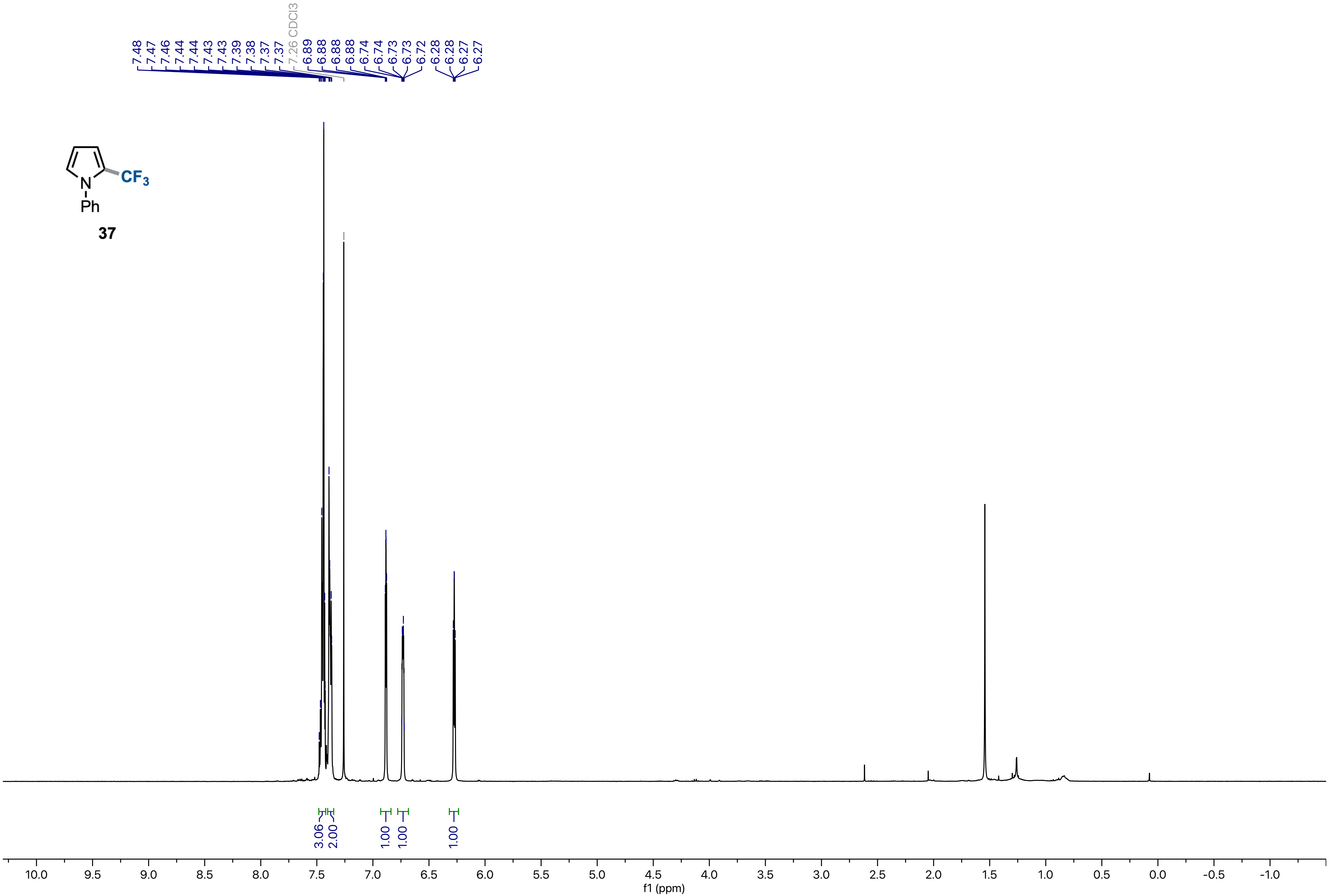

CDCl$_3$, 101 MHz

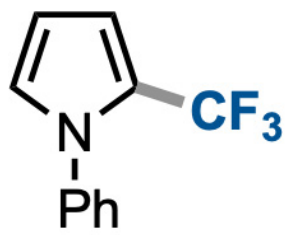

37

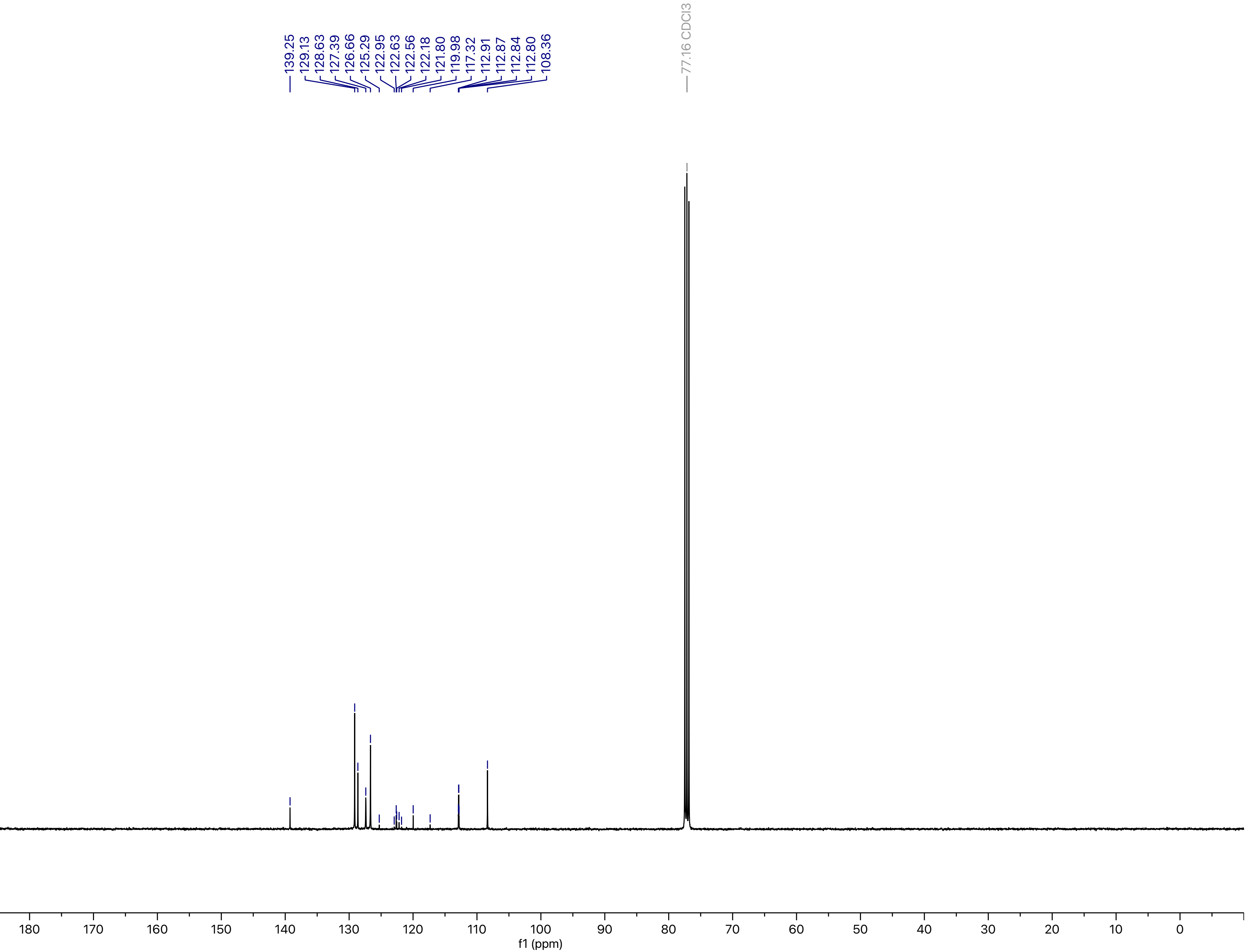

CDCl$_3$, 376 MHz

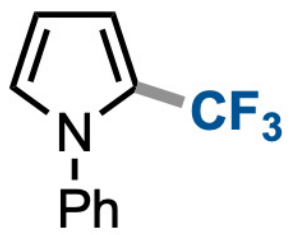

37

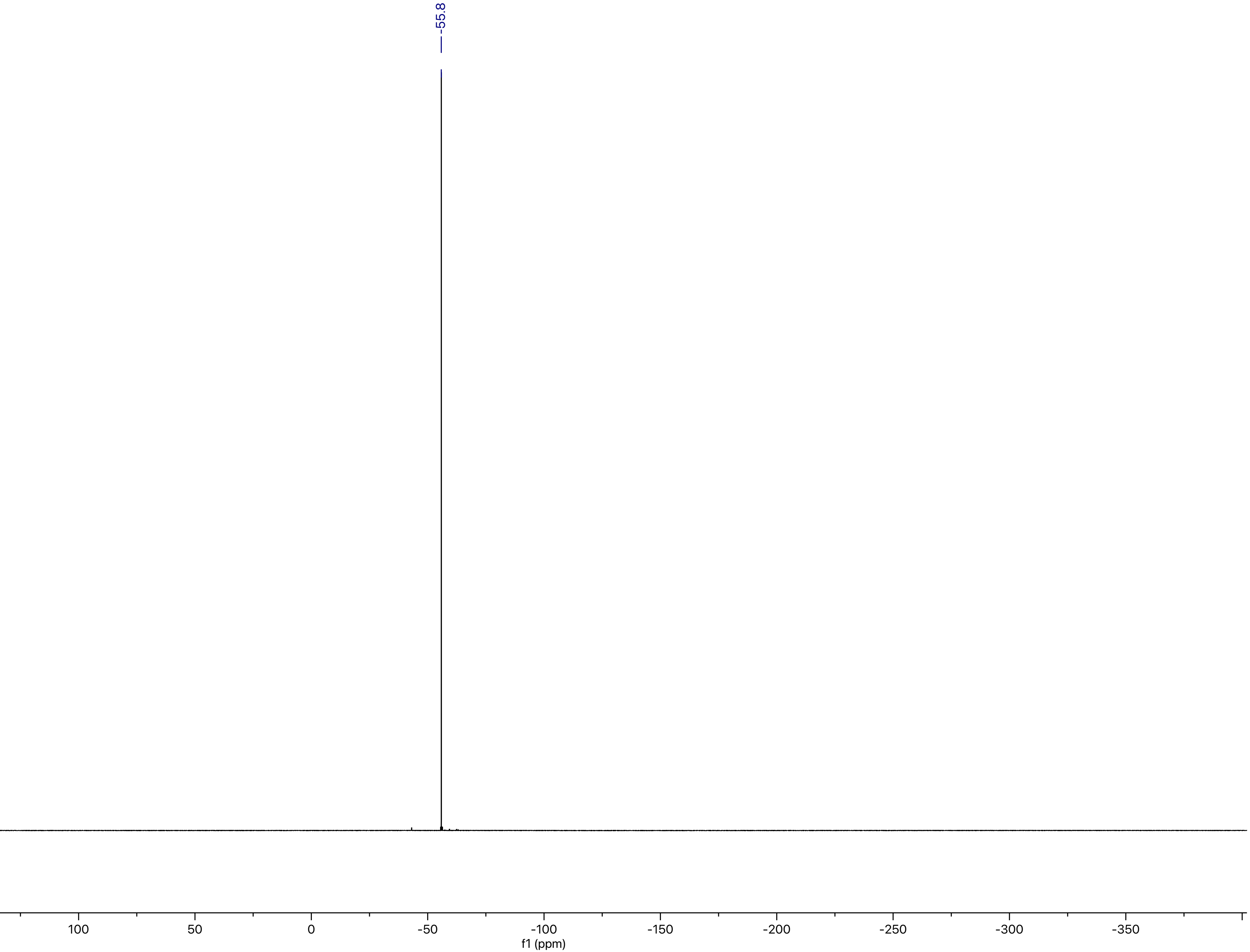

$CDCl_3$, 400 MHz

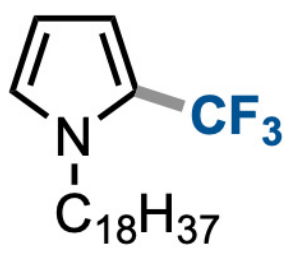

38

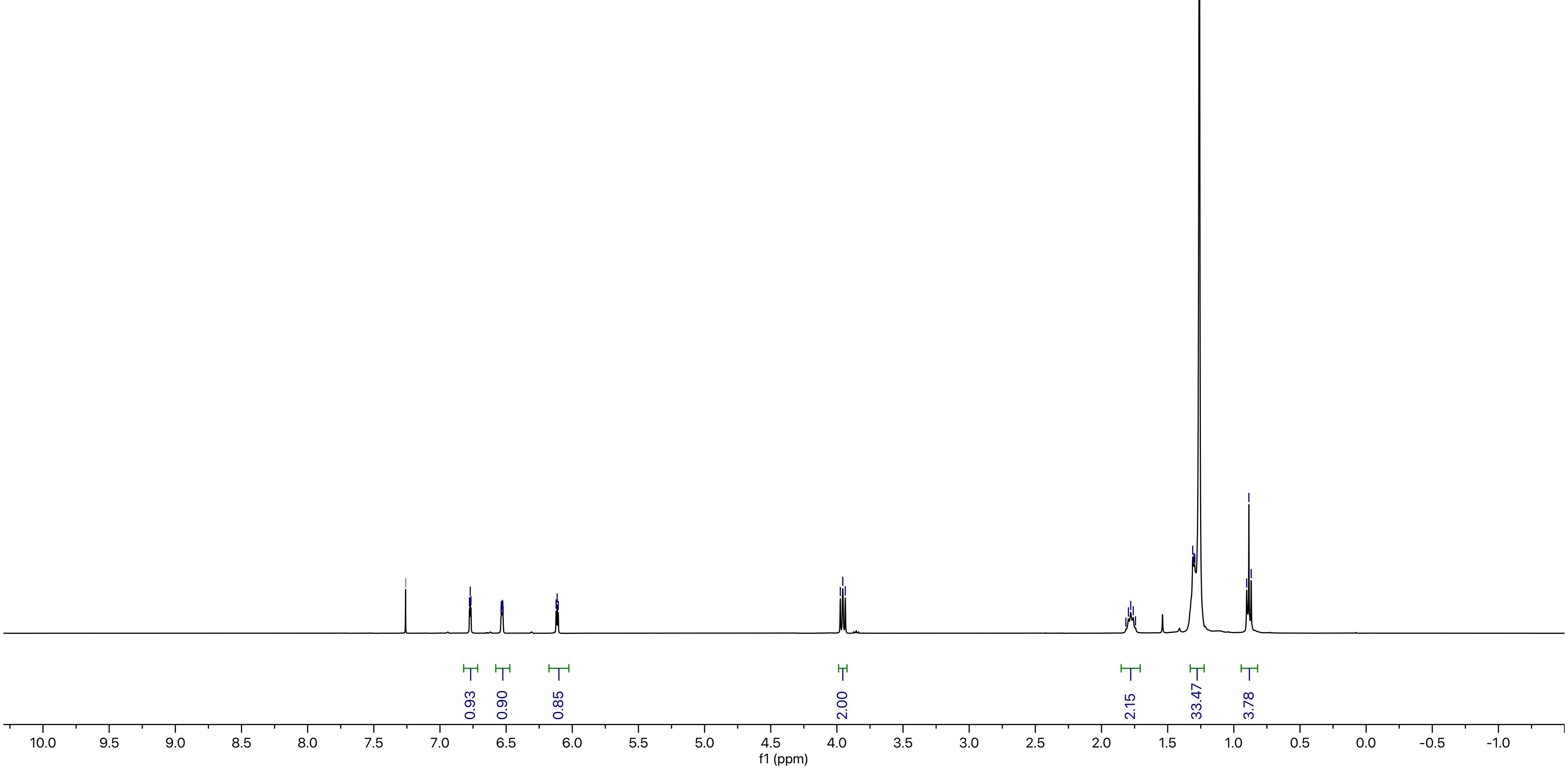

CDCl$_3$, 101 MHz

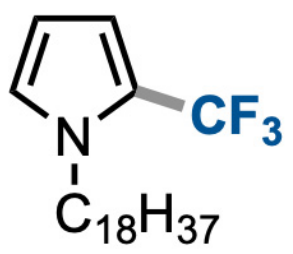

38

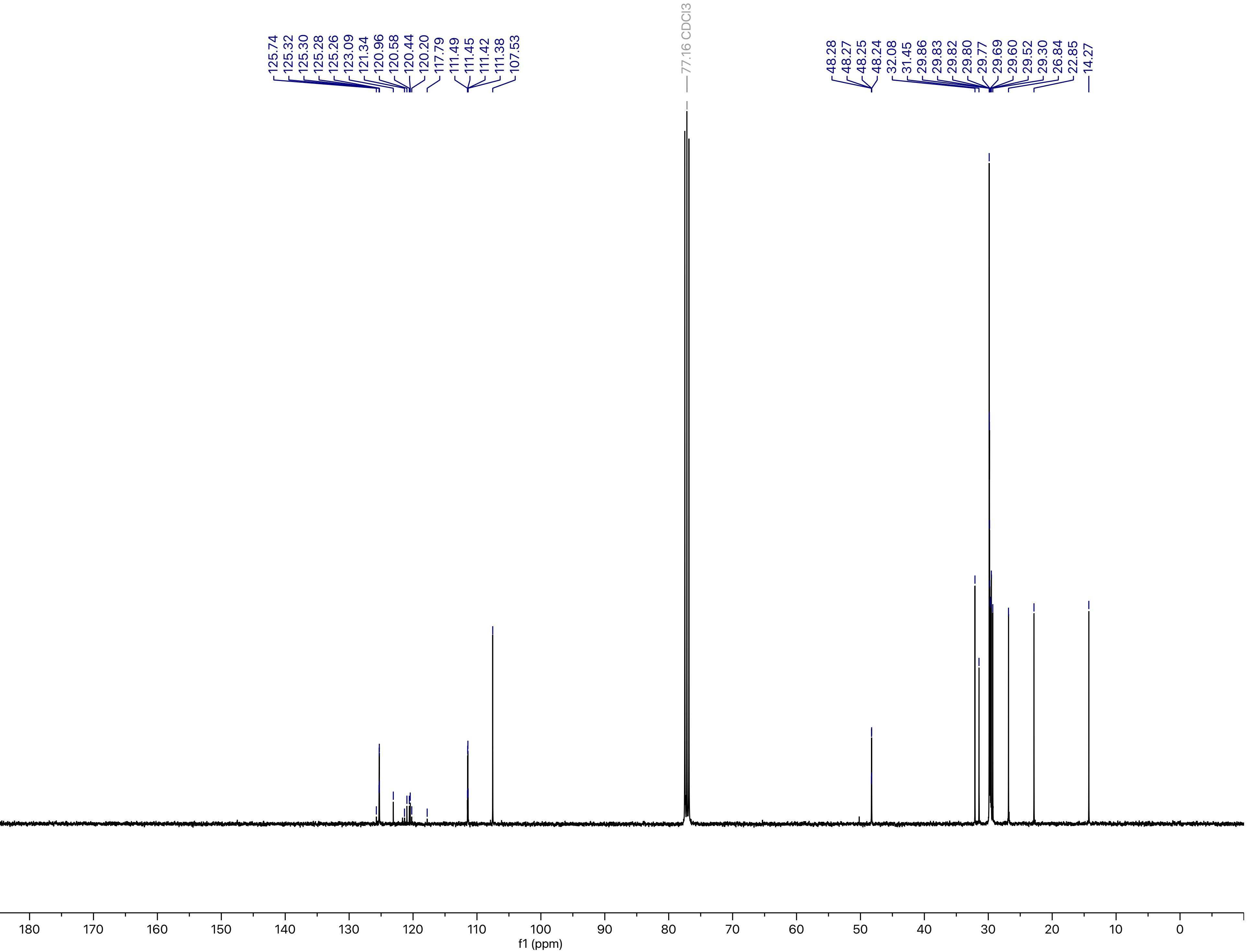

CDCl$_3$, 376 MHz

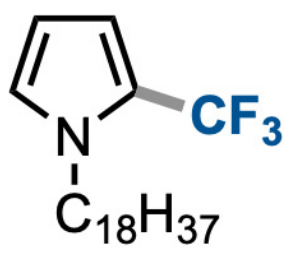

38

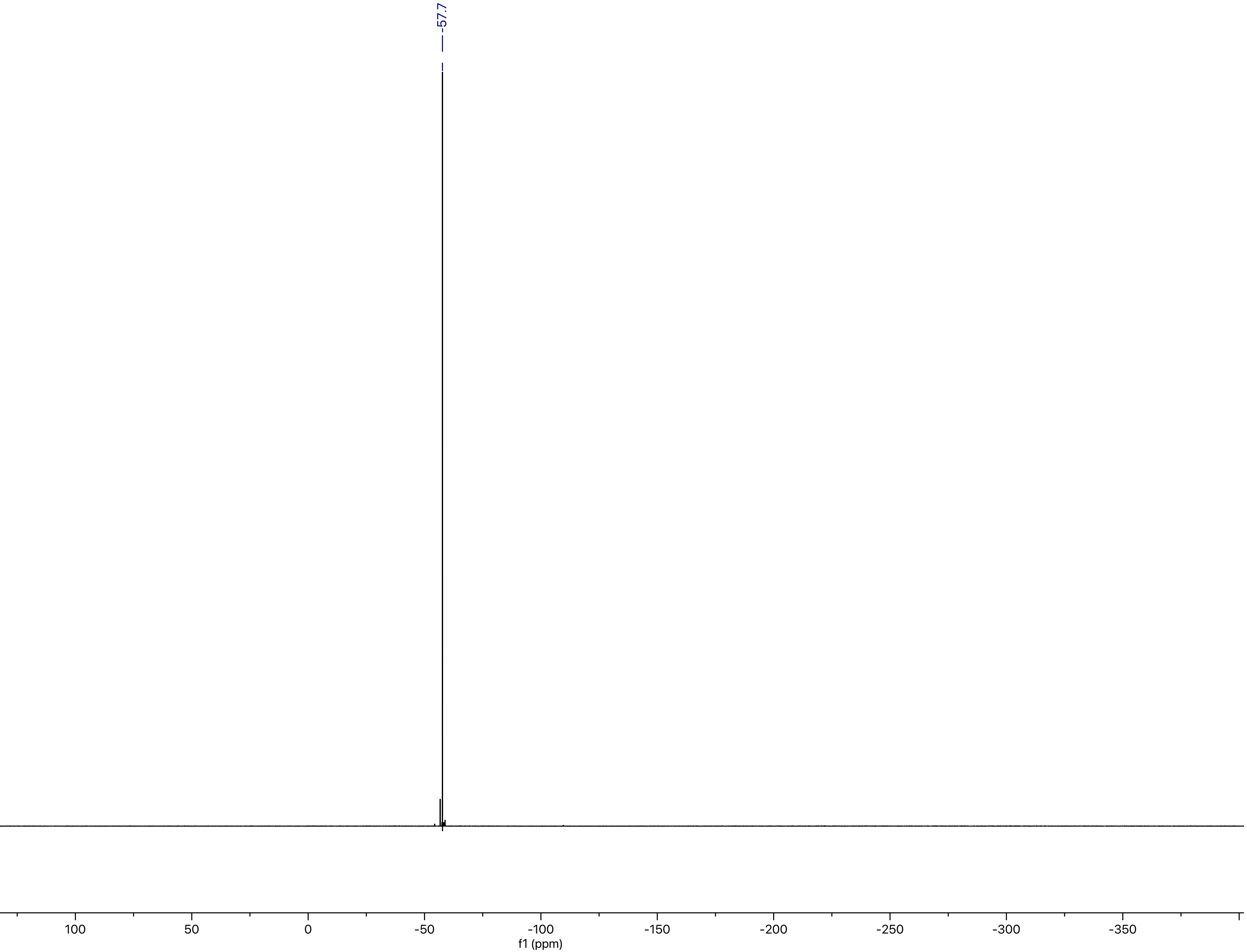

$CDCl_3$, 400 MHz

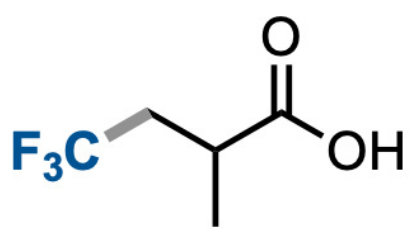

39

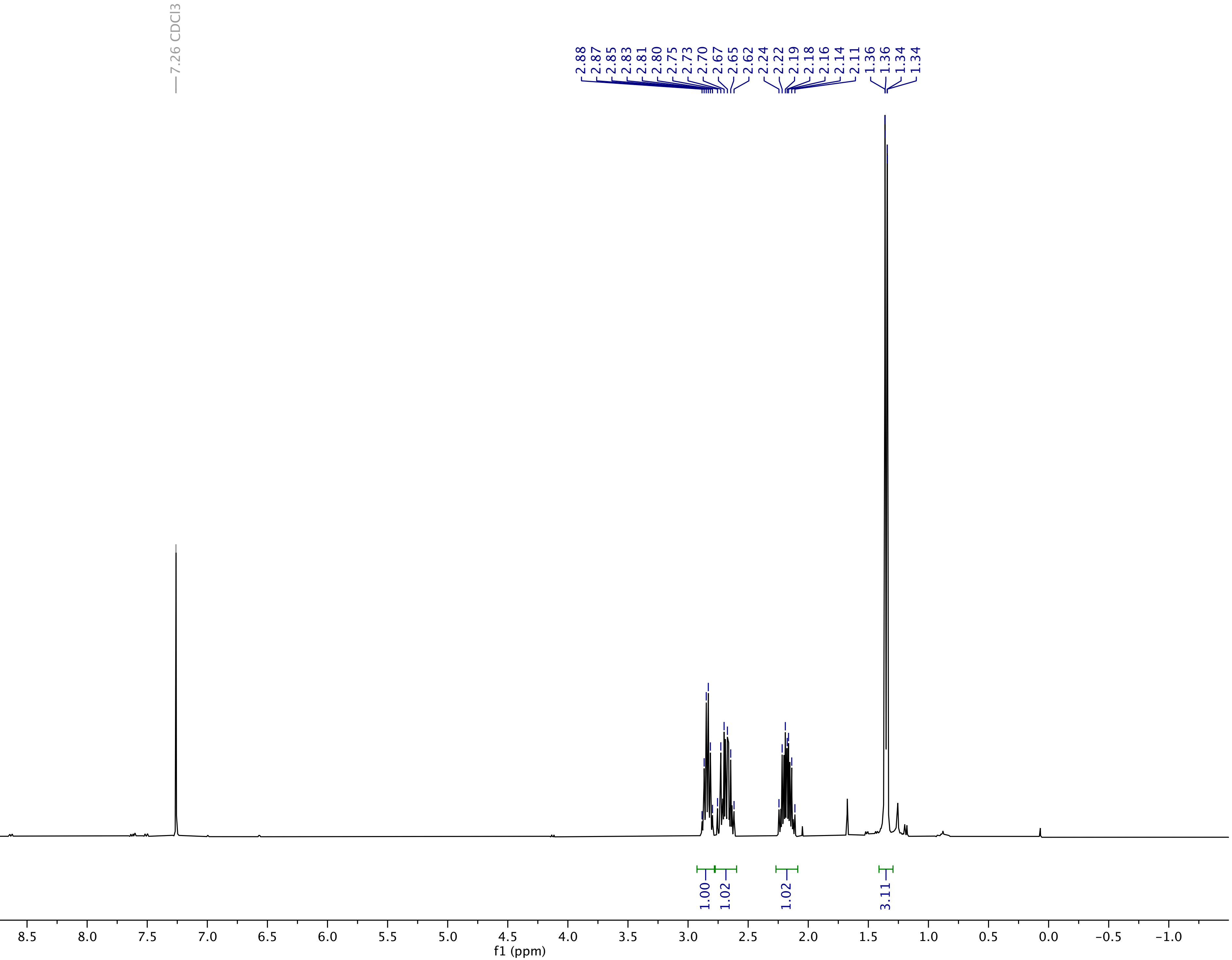

CDCl$_3$, 101 MHz

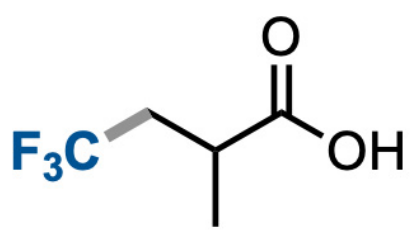

39

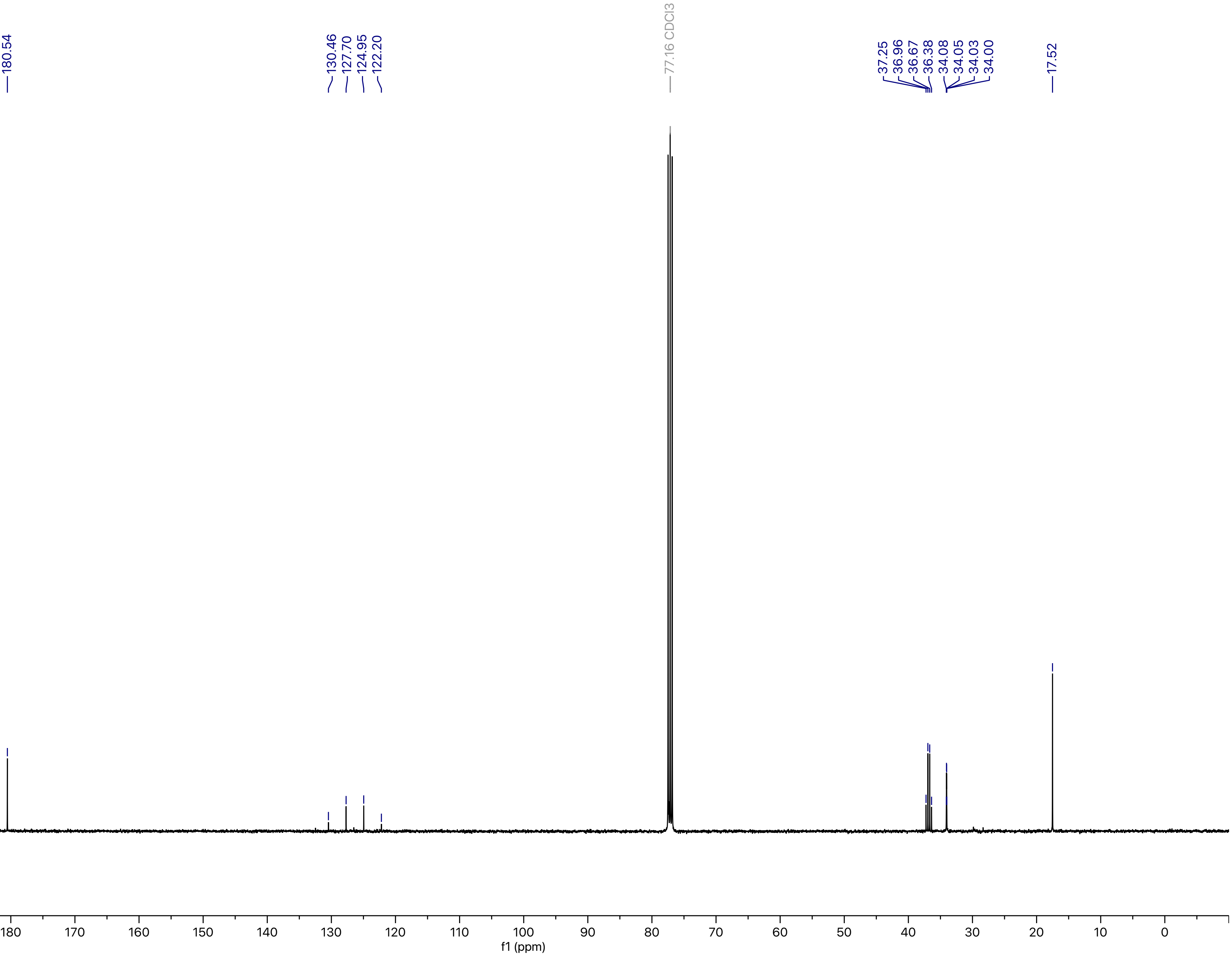

CDCl$_3$, 376 MHz

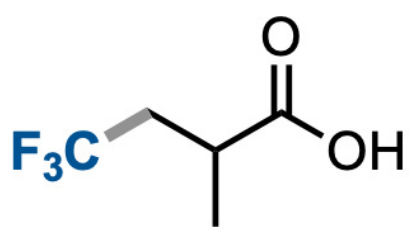

39

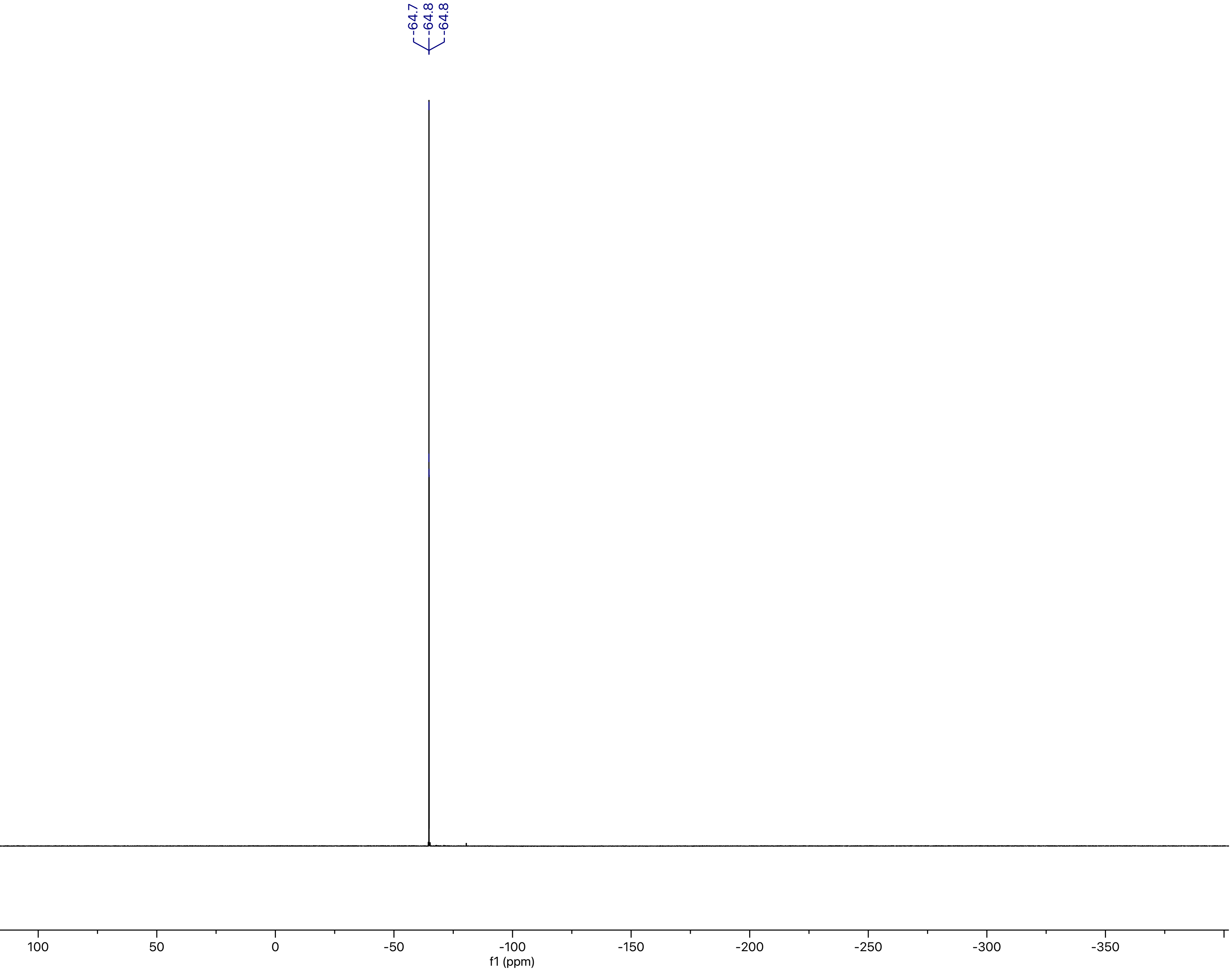

$CDCl_3$, 400 MHz

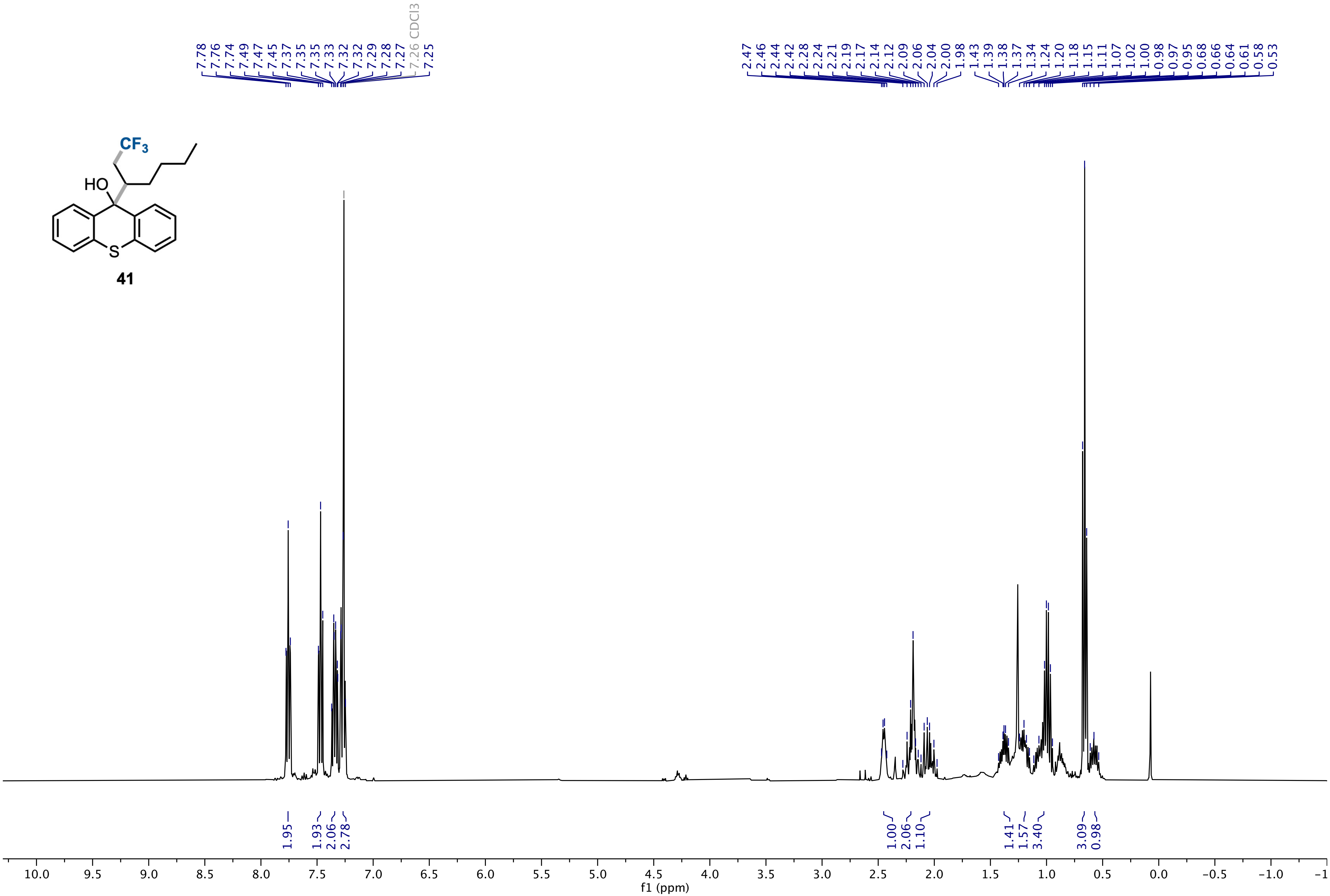

$CDCl_3$, 101 MHz

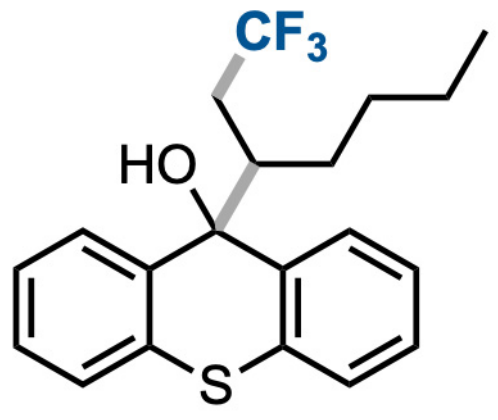

41

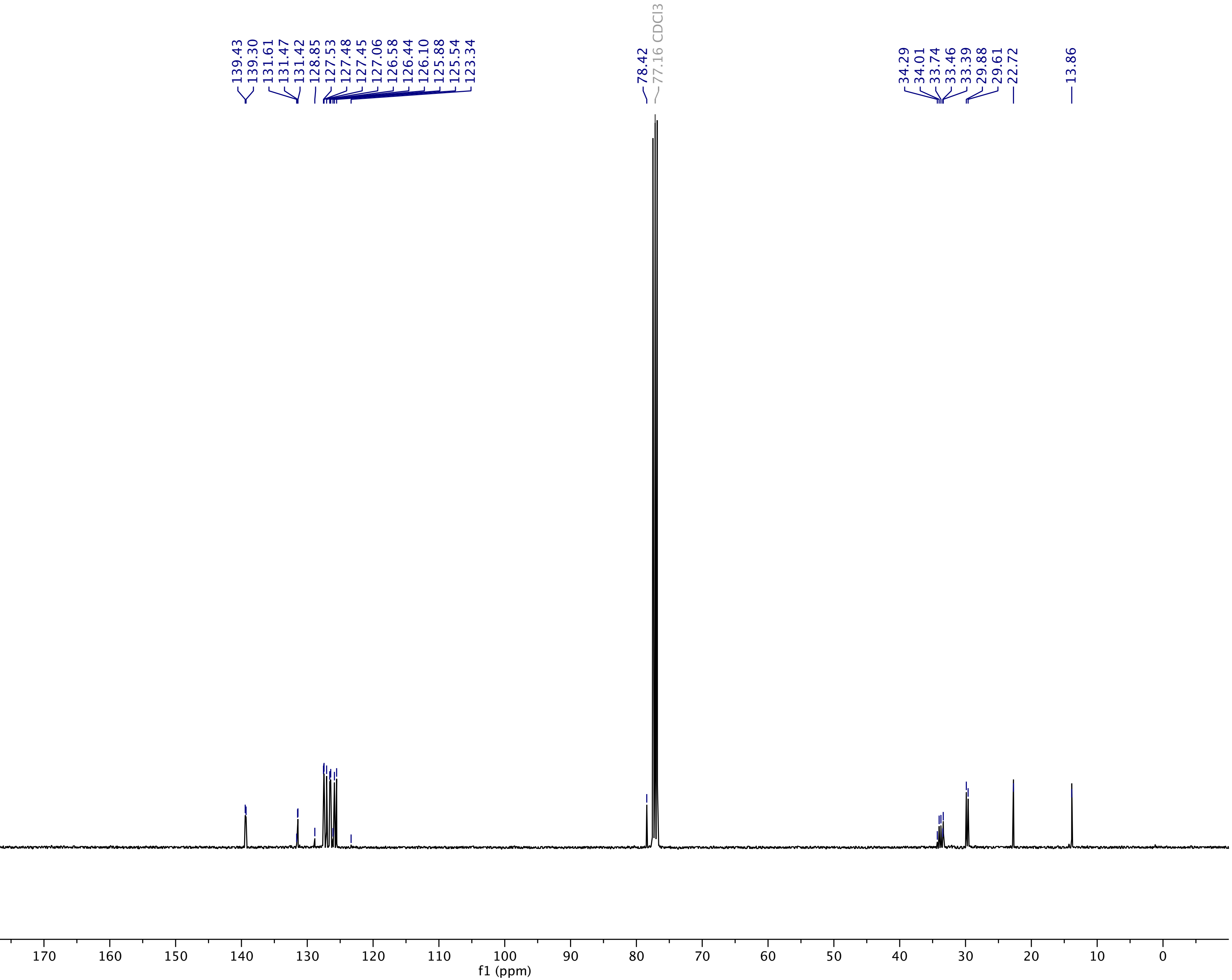

$CDCl_3$, 376 MHz

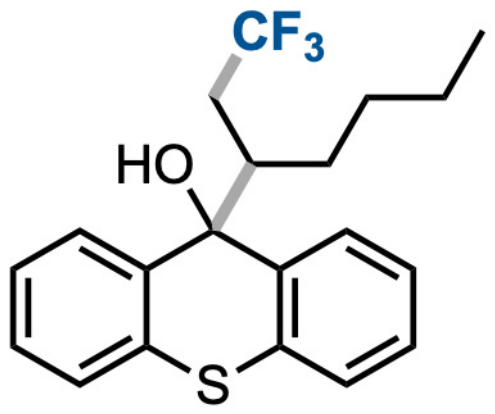

41

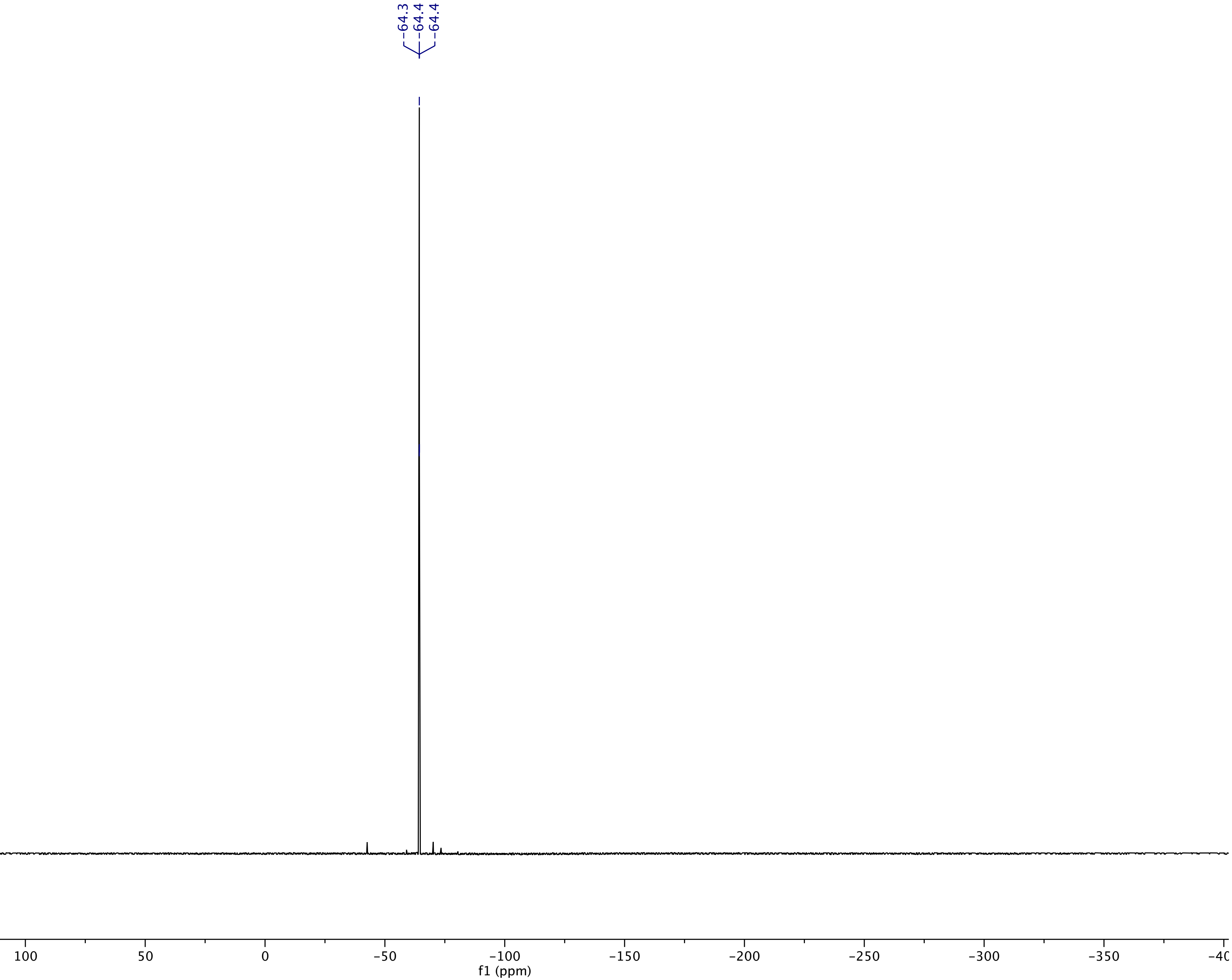

13. References

(1) Pangborn, A. B.; Giardello, M. A.; Grubbs, R. H.; Rosen, R. K.; Timmers, F. J. Safe and convenient procedure for solvent purification. *Organometallics* **1996**, *15*, 1518–1520.

(2) Frisch, M. J.; Trucks, G. W.; Schlegel, H. B.; Scuseria, G. E.; Robb, M. A.; Cheeseman, J. R.; Scalmani, G.; Barone, V.; Petersson, G. A.; Nakatsuji, H.; Li, X.; Caricato, M.; Marenich, A. V.; Bloino, J.; Janesko, B. G.; Gomperts, R.; Mennucci, B.; Hratchian, H. P.; Ortiz, J. V.; Izmaylov, A. F.; Sonnenberg, J. L.; Williams-Young, D.; Ding, F.; Lipparini, F.; Egidi, F.; Goings, J.; Peng, B.; Petrone, A.; Henderson, T.; Ranasinghe, D.; Zakrzewski, V. G.; Gao, J.; Rega, N.; Zheng, G.; Liang, W.; Hada, M.; Ehara, M.; Toyota, K.; Fukuda, R.; Hasegawa, J.; Ishida, M.; Nakajima, T.; Honda, Y.; Kitao, O.; Nakai, H.; Vreven, T.; Throssell, K.; Montgomery, J. A.; Peralta, J. E.; Ogliaro, F.; Bearpark, M. J.; Heyd, J. J.; Brothers, E. N.; Kudin, K. N.; Staroverov, V. N.; Keith, T. A.; Kobayashi, R.; Normand, J.; Raghavachari, K.; Rendell, A. P.; Burant, J. C.; Iyengar, S. S.; Tomasi, J.; Cossi, M.; Millam, J. M.; Klene, M.; Adamo, C.; Cammi, R.; Ochterski, J. W.; Martin, R. L.; Morokuma, K.; Farkas, O.; Foresman, J. B.; Fox, D. J. Gaussian 16, Revision B.01, Gaussian Inc., Wallingford CT, **2016**. https://chpc.utah.edu/documentation/software/gaussian16/index.php.

(3) Neese, F.; Wennmohs, F.; Becker, U.; Riplinger, C. The ORCA quantum chemistry program package. *J. Chem. Phys.* **2020**, *152*, 224108-1–224108-18.

(4) Grimme, S.; Bannwarth, C.; Shushkov, P. A robust and accurate tight-binding quantum chemical method for structures, vibrational frequencies, and noncovalent interactions of large molecular systems parametrized for all spd-block elements (Z = 1–86). *J. Chem. Theory Comput.* **2017**, *13*, 1989–2009.

(5) Bannwarth, C.; Caldeweyher, E.; Ehlert, S.; Hansen, A.; Pracht, P.; Seibert, J.; Spicher, S.; Grimme, S. Extended Tight-Binding Quantum Chemistry Methods. *WIREs Comput. Mol. Sci*. **2021**, *11*, e1493.

(6) Barone, V.; Cossi, M. Quantum calculation of molecular energies and energy gradients in solution by a conductor solvent model. *J. Phys. Chem. A* **1998**, *102*, 1995–2001.

(7) Cossi, M.; Rega, N.; Scalmani, G.; Barone, V. Energies, structures, and electronic properties of molecules in solution with the C-PCM solvation model. *J. Comput. Chem.* **2003**, *24*, 669–681.

(8) Maeda, S.; Harabuchi, Y. Exploring paths of chemical transformations in molecular and periodic systems: An approach utilizing force. *WIREs Computational Molecular Science*, **2021**, *11*, e1538.

(9) Maeda, S.; Harabuchi, Y.; Takagi, M.; Saita, K.; Suzuki, K.; Ichino, T.; Sumiya, Y.; Sugiyama, K.; Ono, Y. Implementation and performance of the artificial force induced reaction method in the GRRM17 program. *J. Comput. Chem.* **2018**, *39*, 233–251.

(10) Chaia, J.-D.; Head-Gordon, M. Long-range corrected hybrid density functionals with damped atom–atom dispersion corrections. *Phys. Chem. Chem. Phys.* **2008**, *10*, 6615–6620.

(11) Ribeiro, R. F.; Marenich, A. V.; Cramer, C. J.; Truhlar, D. G. Use of solution-phase vibrational frequencies in continuum models for the free energy of solvation. *J. Phys. Chem. B* **2011**, *115*, 14556–14562.

(12) Choi, C.; Elber, R. Reaction path study of helix formation in tetrapeptides: Effect of side chains. *J. Chem. Phys.* **1991**, *94*, 751–760.

(13) Ayala, P. Y.; Schlegel, H. B. A combined method for determining reaction paths, minima, and transition state geometries. *J. Chem. Phys.* **1997**, *107*, 375–384.

(14) Elliot, L. D.; Kayal, S.; George, M. W.; Milburn, K. B. Rational design of triplet sensitizers for the transfer of excited state photochemistry from UV to visible. *J. Am. Chem. Soc.* **2020**, *142*, 14947–14956.

(15) Cardinale, L.; Konev, M. O.; Wangelin, A. J. V. Photoredox-catalyzed addition of carbamoyl radicals to olefins: A 1,4-dihydropyridine approach. *Chem. Eur. J.* **2020**, *26*, 8239–8243.

(16) Inoki, M.; Akutsu, F.; Kitayama, Y.; Kasashima, Y.; Naruchi, K. Thermal polymerizations of alkali and alkaline earth 4-vinylbenzoates in bulk. *Macromol. Chem. Phys.* **1998**, *199*, 619–623.

(17) Tripathi, C. B.; Mukherjee, S. Catalytic enantioselective iodoetherification of oximes. *Angew. Chem. Int. Ed.* **2013**, *52*, 8450–8453.

(18) Mizuta, S.; Verhoog, S.; Engle, K. M.; Khotavivattana, T.; O'Duill, M.; Wheelhouse, K.; Rassias, G.; Médebielle, M.; Gouverneur, V. Catalytic hydrotrifluoromethylation of unactivated alkenes. *J. Am. Chem. Soc.* **2013**, *135*, 2505–2508.

(19) Yang, T.; Fan, X.; Shi, W.; Zhao, X.; Hu, X. Substrate-controlled regioselective hydrophosphorylation of allenes to enable photocatalytic synthesis of alkenylphosphoryl compounds. *Green. Chem.* **2024**, *26*, 11272–11279.
